# Zirconium‐Catalyzed Atom‐Economical Synthesis of 1,1‐Diborylalkanes from Terminal and Internal Alkenes

**DOI:** 10.1002/anie.202002642

**Published:** 2020-05-27

**Authors:** Xianjin Wang, Xin Cui, Sida Li, Yue Wang, Chungu Xia, Haijun Jiao, Lipeng Wu

**Affiliations:** ^1^ State Key Laboratory for Oxo Synthesis and Selective Oxidation Suzhou Research Institute of LICP Lanzhou Institute of Chemical Physics (LICP) Chinese Academy of Sciences Lanzhou 730000 P. R. China; ^2^ Leibniz-Institut für Katalyse e. V. Albert-Einstein-Strasse 29a 18059 Rostock Germany; ^3^ University of Chinese Academy of Sciences Beijing 100049 P. R. China

**Keywords:** alkenes, borane, boration, homogeneous catalysis, zirconium

## Abstract

A general and atom‐economical synthesis of 1,1‐diborylalkanes from alkenes and a borane without the need for an additional H_2_ acceptor is reported for the first time. The key to our success is the use of an earth‐abundant zirconium‐based catalyst, which allows a balance of self‐contradictory reactivities (dehydrogenative boration and hydroboration) to be achieved. Our method avoids using an excess amount of another alkene as an H_2_ acceptor, which was required in other reported systems. Furthermore, substrates such as simple long‐chain aliphatic alkenes that did not react before also underwent 1,1‐diboration in our system. Significantly, the unprecedented 1,1‐diboration of internal alkenes enabled the preparation of 1,1‐diborylalkanes.

## Graphical abstract

**An earth‐abundant zirconium‐based** catalyst has been developed for the selective 1,1‐diboration of bulk and inexpensive alkenes with a borane without the need for an additional H_2_ acceptor. Many aryl alkenes as well as long‐chain aliphatic alkenes convert smoothly into their corresponding 1,1‐diborylalkanes and the method also allows the unprecedented remote 1,1‐diboration of internal alkenes.

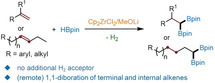

In recent years, 1,1‐diborylalkanes have emerged as versatile building blocks and fundamental intermediates.^1^ Traditionally, they were synthesized from the reaction of bis(boryl)methane with alkylhalides^2^ or the boration of geminal dihalides.^3^ Approaches such as the dihydroboration of alkynes^4^ and the hydroboration of vinyl boronate esters were also known,1a, 1c, 5 but they are relatively less practical because of the accessibility of the substrates. Hence, several other approaches have recently been developed, for example, the insertion of diazoalkanes into diboron compounds,^6^ Ir‐ or Co‐catalyzed benzylic C−H diboration,^7^ direct C−H boration of alkyl boronate esters,^8^ and the deoxygenative diboration of carbonyl compounds,^9^ to expand the synthetic routes to 1,1‐diborylalkanes.

Despite the above progress, the synthesis of 1,1‐diborylalkanes from readily available alkenes has seldom been achieved, with only two systems reported to our knowledge. In 2017, a nickel‐catalyzed 1,1‐diboration of alkenes with bis(pinacolato)diboron (B_2_pin_2_)as the boration reagent was developed.^10^ The direct 1,1‐diboration of alkenes using a relatively inexpensive borane as the boration reagent has proven to be challenging because of the highly reductive nature of boranes which, when reacting with alkenes, would preferably afford the saturated mono‐hydroboration products and the reaction would be terminated.^11^ To achieve the selective 1,1‐diboration of alkenes with a borane, a catalyst should fulfill the following criteria: 1) chemoselective formation of a vinyl boronate ester instead of an alkyl boronate ester (DHB over HB; DHB=dehydrogenative boration, HB=hydroboration);11c, 12 2) proceed by chemoselective HB instead of DHB of the vinyl boronate ester, to produce a diborylalkane rather than a diborylalkene;^10^, ^13^ 3) undergo regioselective HB of the vinyl boronate ester at the α‐position of the bulky Bpin group instead of at the β‐position (1,1‐diboration over 1,2‐diboration).^14^ Thus, besides selectivity issues, a catalyst must also possess self‐contradictory reactivities (DHB and HB), which is difficult to balance (Scheme 1 a). Indeed, it was not until 2018 that a Co(acac)_2_/phosphine system was reported for the synthesis of 1,1‐diborylalkanes from alkenes and HBpin, but 1.2 equiv of norbornene or cyclooctene had to be added as an H_2_ acceptor (Scheme 1 b).^15^ In addition, the substrates were limited to 1,1‐disubstituted alkenes. Moreover, no system was applicable to the bulkier and industrially favored internal alkenes.^16^ Thus, a general and direct 1,1‐diboration of alkenes with a borane for the preparation of 1,1‐diborylalkanes without the need for an H_2_ acceptor remains underdeveloped. Herein, we report our application of a zirconium‐based catalyst that allows the first example of the 1,1‐diboration of alkenes with a borane to be realized without the need for an additional H_2_ acceptor. Our system is operationally simple, cost‐efficient, and suitable for a series of different alkenes, ranging from aryl alkenes to long‐chain aliphatic alkenes. Significantly, the unprecedented remote 1,1‐diboration of internal alkenes can also be achieved (Scheme 1 c).

**Scheme 1:**
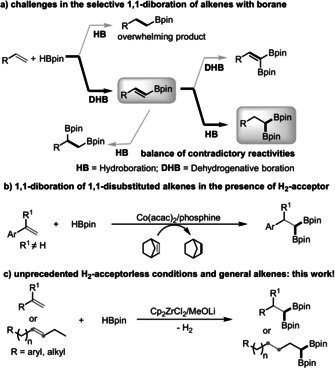
Catalytic 1,1‐diboration of alkenes with a borane: a) challenges and possible side pathways; b) strategy using an excess amount of an H_2_ acceptor; c) our current method without an additional H_2_ acceptor for the (remote) 1,1‐diboration of terminal and internal alkenes.

Early transition metals often have different structures and orthogonal reactivities compared with later ones.^17^ Moreover, several of them are earth‐abundant, for example, zirconium, which is almost as abundant as carbon. In addition, zirconocene complexes have been reported to catalyze the hydroboration of alkynes^18^ and alkenes.^19^ We became interested in exploring the possibility of using zirconocene complexes to achieve the unreported 1,1‐diboration of alkenes without an additional H_2_ acceptor. In a model reaction, 0.2 mmol styrene (**1**) and 0.6 mmol HBpin (**2**) in 1 mL toluene was treated with 5 mol % of different zirconocene complexes at 100 °C. First, the reaction with Cp_2_ZrCl_2_ (Cp=*η* ^5^‐C_5_H_5_) was carried out, but only 6 % of the monohydroboration product **5** was obtained (Table 1, entry 1). The reaction with Cp_2_ZrHCl was then performed and 73 % of the dehydrogenative boration product **4** was observed (entry 2). Interestingly, we found that the addition of MeONa promoted the formation of 1,1‐diborylalkane **3** in 29 % yield (entry 3). Changing the base from MeONa to MeOLi greatly improved the yield of **3** to 88 % (entry 4). The use of other zirconium‐catalysts such as Cp_2_ZrMe_2_ and CpZrCl_3_ gave inferior results (entries 5 and 6). The use of Cp_2_ZrHCl in the presence of MeOLi gave almost similar results as Cp_2_ZrCl_2_ (entry 7). In addition, the reaction temperature and catalyst loading could be further reduced, although slightly lower yields of **3** were obtained (entries 8 and 9). When the reaction was performed with MeOLi alone, only the hydroboration products **5** and **6** were obtained (entry 10). It is also noted that product **5** was not the intermediate of **3**, since treating **5** with HBpin under the catalytic conditions did not lead to any reactions.

**Table 1:** Zirconium‐catalyzed 1,1‐diboration of styrene: optimization of the conditions.^[a]^ 
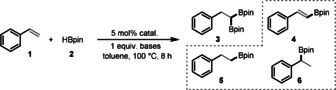

Entry	Catalyst	Base	Yields [%]^[b]^			
			**3**	**4**	**5**	**6**
1	Cp_2_ZrCl_2_	–	0	0	6	0
2	Cp_2_ZrHCl	–	0	73	0	0
3	Cp_2_ZrCl_2_	MeONa	29	47	11	1
4	Cp_2_ZrCl_2_	MeOLi	88	2	6	1
5	Cp_2_ZrMe_2_	MeOLi	63	0	34	2
6	CpZrCl_3_	MeOLi	3	1	84	12
7	Cp_2_ZrHCl	MeOLi	87	1	7	1
8^[c]^	Cp_2_ZrCl_2_	MeOLi	81	4	8	1
9^[d]^	Cp_2_ZrCl_2_	MeOLi	69	1	24	3
10	–	MeOLi	0	0	83	12

[a] Reaction conditions: 0.2 mmol **1**, 0.6 mmol HBpin, 1 equiv of bases, 5 mol % catalyst, 1 mL toluene in a 15 mL pressure tube heated at 100 °C for 8 h. [b] Yields were determined by GC‐MS using *n*‐dodecane as an internal standard. [c] Reaction was performed at 80 °C. [d] Reaction was performed with a catalyst loading of 2 mol %.

We then investigated the generality of different alkenes under the optimized reaction conditions (Table 1, entry 4). We were pleased to find that our method worked well with numerous aryl alkenes with various electronic and steric substituents (Scheme 2). Thus, substrates with ‐Me, ‐^*t*^Bu, ‐Ph, ‐OMe, ‐NMe_2_, and ‐CF_3_ groups at various positions on the phenyl ring reacted well in our system (65–82 % yields, **3**, **7**–**12**, **17**, **18, 22**, **23**). In addition, halogen‐substituted aryl alkenes selectively produced the corresponding 1,1‐diborylalkanes without side reactions from the C−X bonds (X=F, Cl, Br), and yields up to 80 % were obtained (**13**–**15**, **19**–**21**, **24**, **25**). Notably, the ‐NH_2_ group, which was expected to react with HBpin, survived in our system (**16**). Moreover, the reaction also worked well with polysubstituted alkenes; for example, the 1,4‐dimethyl and pentafluoro‐substituted aryl alkenes afforded products **26** and **27** in yields of 80 % and 51 %, respectively. Vinylnaphthalene and α‐methylstyrene yielded **28** and **29** in yields of 83 % and 40 %, respectively. Ferrocenyl‐substituted 1,1‐diborylalkane **30** could also be obtained from vinylferrocene. Further extending the substrate scope to heteroatom‐containing alkenes was also successful: 2‐vinylthiophene, 5‐vinylbenzofuran, and 3‐vinyl‐9*H*‐carbazole underwent efficient 1,1‐diboration in yields up to 85 % (**31**–**33**). Of note was that the ‐NH group on the carbazole moiety remained intact (**33**).

**Scheme 2:**
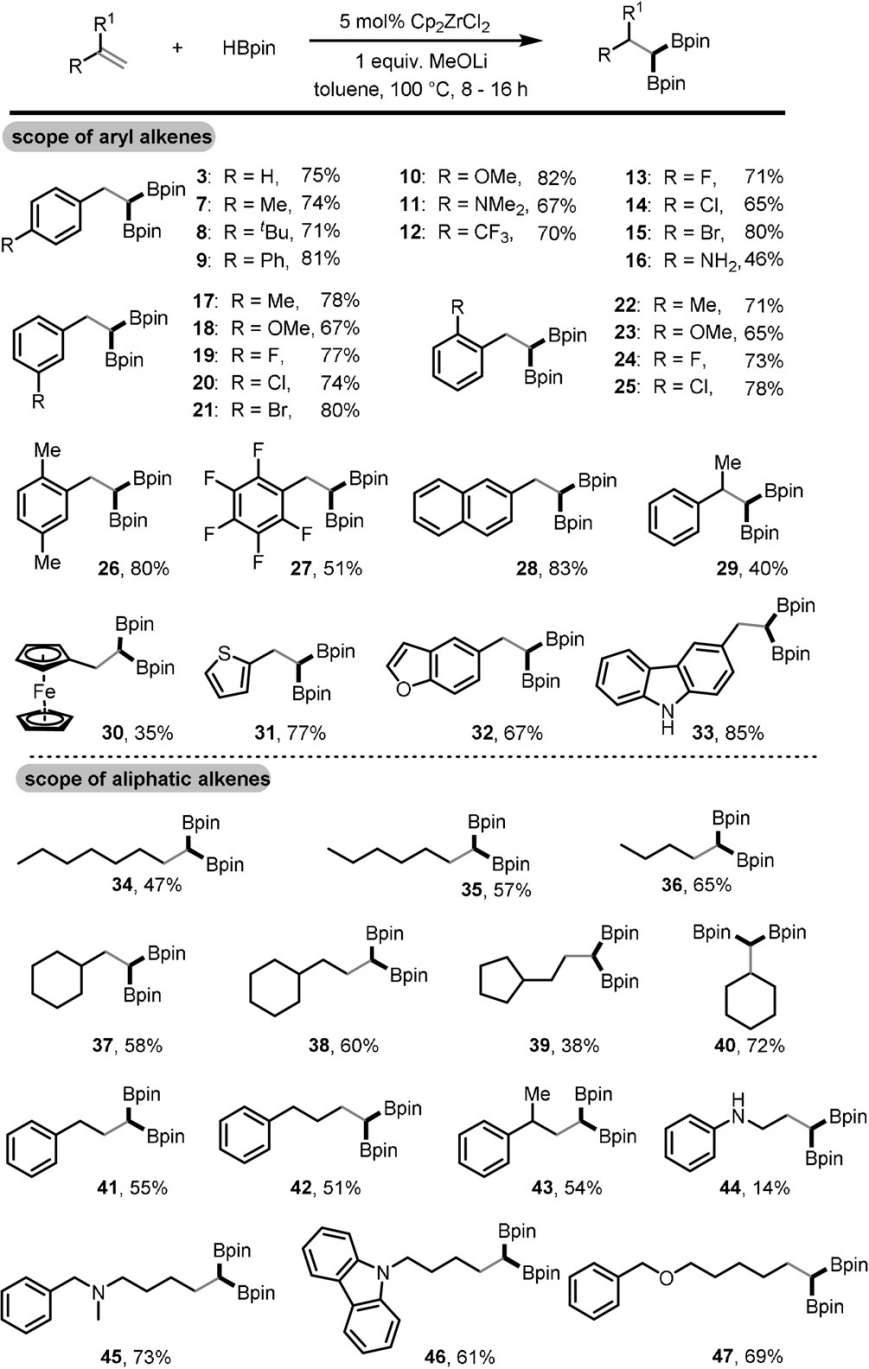
Substrate generality in the zirconium‐catalyzed 1,1‐diboration of various alkenes. Reaction conditions: 0.2 mmol alkenes, 0.6 mmol HBpin, 5 mol % Cp_2_ZrCl_2_, 0.2 mmol MeOLi, and 1 mL toluene in a 15 mL pressure tube, heated at 100 °C for 8–16 h; yields of isolated products are given.

Next, we turned our attention to validate the generality of our method to long‐chain aliphatic alkenes. We were pleased to find that our procedure worked for a series of bulk aliphatic alkenes with different carbon lengths, although with reduced yields because of the presence of alkane and mono‐hydroboration side products: thus **34**–**36** were obtained in yields up to 65 %. In addition, our diboration process was also applicable to aliphatic alkenes with various substituents: cyclohexyl‐, cyclopentyl‐, and phenyl‐substituted aliphatic alkenes all worked well to give the corresponding products in yields up to 72 % (**37**–**43**). *N*‐Allylaniline was one exception, with **44** being obtained in only 14 % yield. Fortunately, other N‐ and O‐containing moieties, such as methylbenzylamine and benzyl ether, all survived the 1,1‐diboration process, with yields ranging from 61 % to 73 % (**45**–**47**).

We then focused on gaining insight into the reaction mechanism. Analysis of the stoichiometric reaction of Cp_2_ZrCl_2_, MeOLi, and HBpin (molar ratio 1:2:2) revealed the formation of MeO‐Bpin, which was also detected in the catalytic reaction with or without styrene (Figures S1–S3). In addition, Zr‐H was detected in the ^1^H NMR spectrum by trapping with acetone (Figure S4). Furthermore, H_2_ and vinyl boronate ester **4** were observed in the ^1^H NMR spectrum during the catalytic reaction with styrene (Figure S5). Moreover, the sequential addition of MeOLi followed by HBpin to the solution of Cp_2_ZrCl_2_ in [D_8_]toluene showed a color change from colorless to dark brown as well as changes in the chemical shifts of the Cp and MeO^−^ signals in the ^1^H NMR spectra (Figure S6). On the basis of those observations as well as literature precedents,1c, 20 we propose the following reaction mechanism: Cp_2_ZrCl_2_ first interacts with 2 equiv MeOLi to yield Cp_2_Zr(OMe)_2_, and then further reacts with HBpin to form Cp_2_ZrH_2_ (**A**) and MeO‐Bpin. Cp_2_ZrH_2_ (**A**) enters into the catalytic cycle by reaction with HBpin and forms Cp_2_Zr(H)Bpin (**B**) with release of H_2_. Then, insertion of alkene into the Zr−B bond forms the zirconium boryl alkyl species (**C**), which gives the vinyl boronate ester intermediate **4** and regenerates Cp_2_ZrH_2_ (**A**) after β‐H elimination. The addition of Cp_2_Zr(H)Bpin (**B**) to vinyl boronate ester **4** affords zirconium diboryl alkyl species (**D**), which then reacts with another HBpin through σ‐bond metathesis to give 1,1‐diborylalkane **3** (Scheme 3).

**Scheme 3:**
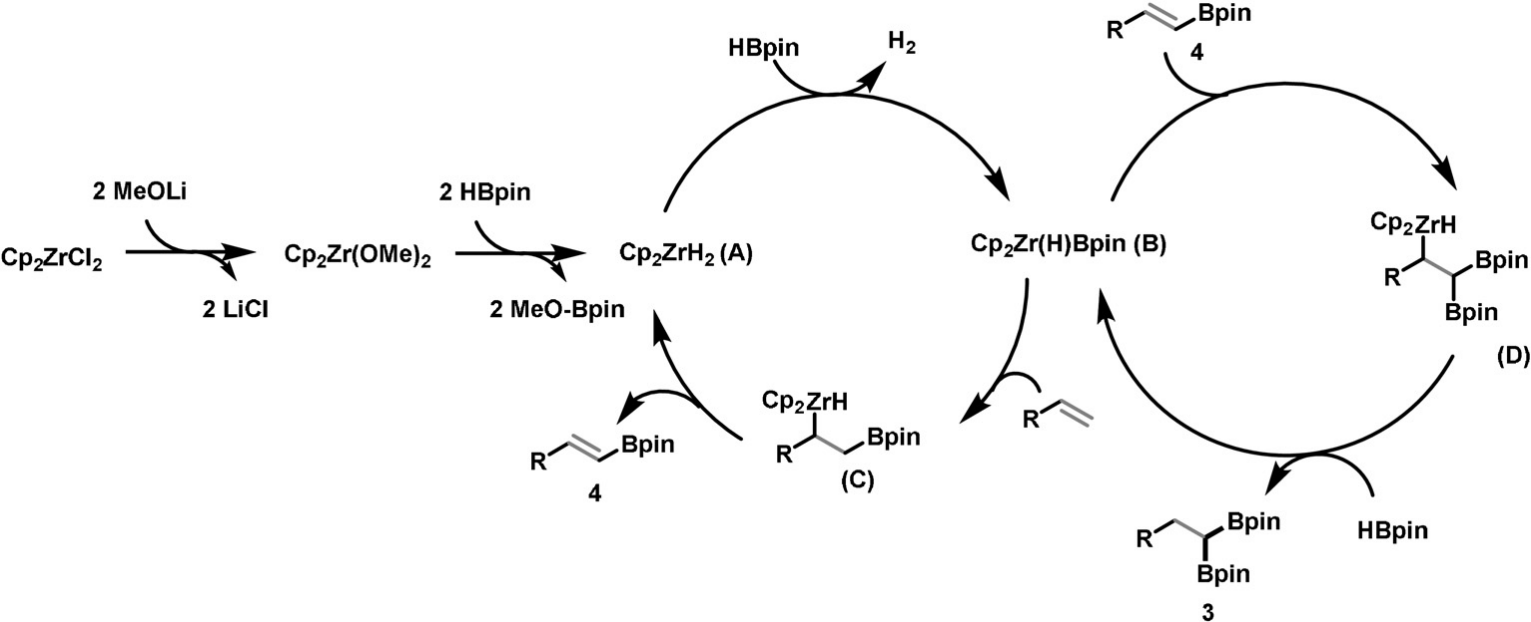
Proposed reaction mechanism.

Mixtures of internal alkenes, commonly obtained from cracking processes, are relatively cheap and easily accessible.^16^, ^21^ However, the selective remote 1,1‐diboration of simple and bulk internal alkenes has not yet been reported. It is well‐known that a stoichiometric amount of a zirconium‐reagent can mediate the isomerization of double bonds,^22^ and one catalytic system has recently been reported.^23^ We thus became interested in exploring whether the isomerization of internal alkenes to terminal alkenes and a subsequent selective 1,1‐diboration could be achieved using our system. To our delight, a variety of internal alkenes with different chain lengths and positions of the double bond worked well in our system (Scheme 4).

**Scheme 4:**
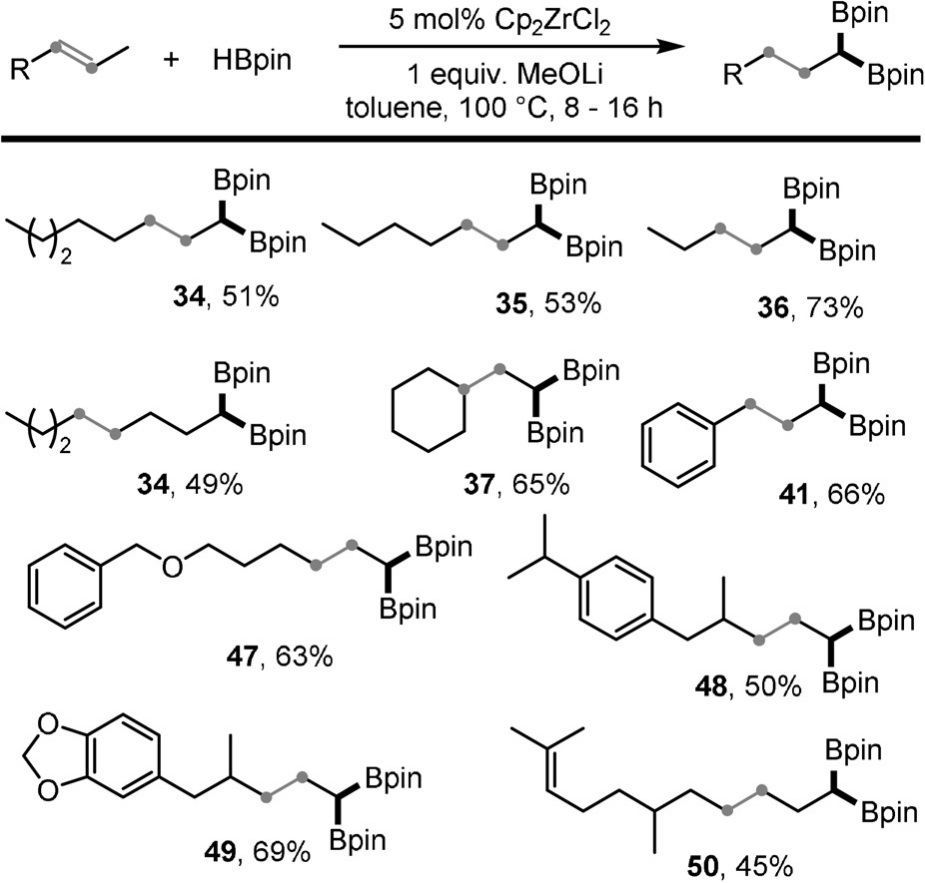
Zirconium‐catalyzed remote 1,1‐diboration of various internal alkenes. Reaction conditions: 0.2 mmol internal alkenes, 0.6 mmol HBpin, 5 mol % Cp_2_ZrCl_2_, 0.2 mmol MeOLi, and 1 mL toluene in a 15 mL pressure tube, heated at 100 °C for 8–16 h; yields of isolated products are given.

2‐Octene, 2‐heptene, and 2‐pentene afforded the corresponding 1,1‐diborylalkanes in 51–73 % yield. A yield comparable to that with 2‐octene was also achieved from 4‐octene, thereby illustrating the efficiency of our catalytic system. Ethylidenecyclohexane afforded **37** in 65 % yield, and β‐methylstyrene, which was reluctant to isomerize, underwent efficient remote 1,1‐diboration and gave the terminal 1,1‐diboration product **41** in 66 % yield. Moreover, an internal alkene with a benzyl ether group underwent the 1,1‐dibroation without problems to afford the corresponding product **47** in 63 % yield. In addition, sterically hindered (*E*)‐1‐isopropyl‐4‐(2‐methylpent‐3‐en‐1‐yl)benzene and an alkene derived from helional produced **48** and **49**, respectively, in yields of 50 % and 69 %. In the case of (*E*)‐2,6‐dimethylundeca‐2,8‐diene, which contains two internal double bonds, selective reaction at the less hindered double bond was achieved, with an acceptable yield of product **50**.

In summary, we have developed an earth‐abundant zirconium‐based catalytic system that enabled the first selective 1,1‐diboration of bulk and inexpensive alkenes with a borane to be realized without the need for an excess amount of another alkene as an H_2_ acceptor. Thus, our strategy is both atom‐economical and cost‐efficient. In contrast to the limited substrate scope of the existing diboration systems with additional acceptors, many aryl alkenes as well as long‐chain aliphatic alkenes converted smoothly into their corresponding 1,1‐diborylalkanes, which are excellent platforms for complex molecules. More importantly, our method also realized the unprecedented remote 1,1‐diboration of internal alkenes.

## Conflict of interest

The authors declare no conflict of interest.

## Supporting information

As a service to our authors and readers, this journal provides supporting information supplied by the authors. Such materials are peer reviewed and may be re‐organized for online delivery, but are not copy‐edited or typeset. Technical support issues arising from supporting information (other than missing files) should be addressed to the authors.

Supplementary
