# Supplementary material for: Zirconium‐Catalyzed Atom‐Economical Synthesis of 1,1‐Diborylalkanes from Terminal and Internal Alkenes
Source: Angew Chem Int Ed Engl. 2020 May 27;59(32):13608–12. doi: 10.1002/anie.202002642 (PMC7496309; doi:10.1002/anie.202002642)
Supplement: Supplementary file 1 — Supplementary [file ANIE-59-13608-s001.pdf]

Angewandte Chemie
Eine Zeitschrift der Gesellschaft Deutscher Chemiker

Supporting Information

Zirconium-Catalyzed Atom-Economical Synthesis of 1,1-Diborylalkanes from Terminal and Internal Alkenes

Xianjin Wang, Xin Cui, Sida Li, Yue Wang, Chungu Xia, Haijun Jiao,* and Lipeng Wu**

anie_202002642_sm_miscellaneous_information.pdf

Table of Contents

General Information........ S2

General Procedure for the Synthesis of 1,1-Diborylalkanes from Alkenes........ S2

Synthetic Procedures........ S3

Initial Condition Optimization Tables........ S6

Mechanism Study Figures........ S13

Synthetic Application of 1,1-Diborylalkanes........ S19

Products Characterization........ S20

NMR Spectra........ S36

References........ S110

General Information

Manipulations were carried out under an atmosphere of dry and deoxygenated N_2 using Schlenk line or in a glovebox (H_2O and O_2 < 0.01 ppm). Glassware was pre-dried in an oven at 150 °C for several hours and cooled prior to use. Solvents were purchased as super dry solvent or purified via standard purification and dry operations. Cp_2ZrCl_2, Cp_2ZrMe_2, Cp_2ZrHCl, $CpZrCl_3$ were purchased from Sigma Aldrich, TCI or Macklin company. Alkenes were purchased from Energy, Adamas, Macklin company or synthesized from the corresponding bromide and potassium vinyltrifluoroborate[1] or from aldehydes and the corresponding alkyl triphenylphosphonium bromide.[2] ^1H NMR, ^{13}C NMR, ^{11}B NMR spectra were recorded on Bruker Advance Neo 400 MHz NMR at room temperature using $CDCl_3$, d_8-Toluene or d_6-DMSO as a solvent. Chemical shifts (δ) are given in parts per million (ppm). Coupling constants (*J*) are given in Hertz (Hz). Thin-layer chromatography (TLC) employed glass 0.25 mm silica gel plates. Flash chromatography columns were packed with 200-300 mesh silica gel in petroleum (bp. 60-90 °C). High-Resolution MS analyses were performed on Agilent 6530 Accurate – Mass Q-TOF LC/MS with ESI mode or Waters Micro Mass GCT Premier with EI mode.

General Procedure for the Synthesis of 1,1-Diborylalkanes from Alkenes

In a nitrogen-filled glovebox, to a 15 mL pressure tube equipped with a magnetic stirrer were added Cp_2ZrCl_2 (2.9 mg, 0.01 mmol), MeOLi (7.6 mg, 0.2 mmol), toluene (1 mL) and HBpin (87 μL, 0.6 mmol) in a sequence manner. After slightly shaking the tube, the corresponding alkene (0.2 mmol) was added. The pressure tube was taken out the glove box and was heated at 100 ºC for 8 – 16 h. Upon completion, methanol was added at room temperature to the reaction and stirred for 5 mins then all the solvent was evaporated, and the crude product was isolated on deactivated silica gel using flash chromatography with petroleum ether/ethyl acetate or dichloromethane/methanol as the eluent to give the corresponding 1,1-diborylalkanes.

Synthetic Procedures

Procedure for the synthesis of 51[3]

In a 25 mL round bottom flask was charged with **3** (0.36 g, 1 mmol), $Pd(dba)_2$ (58 mg, 10 mol%), P*t*Bu_3 (2 mL, 0.1 M hexane solution, 20 mol%), and 5 mL THF. To the above solution was added 4-iodoanisole (0.26 g, 1.1 mmol). Then aqueous KOH solution (1 mL, 3 M, 3 mmol) was added at room temperature. The reaction solution was stirred at room temperature for 12 h then quenched with NH_4Cl, and extracted with Et_2O, the combined ethereal layer was washed with $NaHCO_3$, brine and dried over $MgSO_4$. The solvent was evaporated, and the crude product was isolated on silica gel using flash chromatography (PE: EA = 20:1 as eluent).

Procedure for the synthesis of 52[4]

In a 25 mL round bottom flask was charged with **3** (0.36 g, 1 mmol) and 4 mL MeCN, 4 mL MeOH. To the above mixture, a solution of KF (232 mg, 4 mmol) in 0.6 mL H_2O was added drop wisely, and the mixture was stirred at room temperature for 5 minutes. Then *L*-(+)-tartaric acid (308 mg, 2.04 mmol) in 2 mL THF was added slowly. The reaction mixture was filtered to remove the white precipitate and washed thoroughly with excess amount of MeCN, then the filtrate was concentrated and recrystallized with hexane and Et_2O to give **49** which was further dried under vacuum.

Procedure for the synthesis of 53[5]

Under N_2 atmosphere, a 25 mL schlenk tube was charged with **3** (0.36 g, 1 mmol) and 5 mL THF, the tube was then cooled to 0 ºC. To the above solution was added LTMP (0.15 g, 1 mmol). After 5 minutes, acetophenone (120 µL, 1 mmol) was added and the reaction mixture was stirred at room temperature for 1 h and filtered through a pad of silica gel with diethyl ether (20 mL). The solvent was evaporated, and the crude product was isolated on silica gel using flash chromatography (PE: EA = 40:1 as eluent).

Procedure for the synthesis of 54[6]

Under N_2 atmosphere, a 35 mL pressure tube was charged with **3** (0.72 g, 2 mmol), quinoline-N-oxide (145 mg, 1 mmol) and 10 mL toluene, then MeONa (135 mg, 2.5 mmol) was added. The reaction mixture was stirred at 80 ºC for 16 h then filtered through a plug of celite and washed with Et_2O. The solvent was evaporated, and the crude product was isolated on silica gel using flash chromatography (PE: EA = 10:1 as eluent).

Procedure for the synthesis of 55[7]

Under N_2 atmosphere, a 25 mL schlenk tube was charged with **3** (0.36 g, 1 mmol) and 1-bromo hexane (280 μL, 0.9 mmol) and 5 mL THF, then MeONa (135 mg, 2.5 mmol) was added. The reaction mixture was stirred at room temperature for 16 h and then filtered through a plug of celite and washed with Et_2O. The solvent was evaporated, and the crude product was isolated on silica gel using flash chromatography (PE: EA = 20:1 as eluent). Then the obtained intermediate in a flask was chilled to 0 ºC, to this flask was added 1 mL of degassed mixture of 3M aqueous NaOH and 30% H_2O_2 (1:1). The reaction mixture was allowed to stir at room temperature for 3 h. Then the reaction mixture was extracted with EA three times and the combined organic layers were washed with H_2O, brine and dried over $MgSO_4$. The solvent was evaporated, and the crude product was isolated on silica gel using flash chromatography (PE: EA = 15:1 as eluent).

Procedure for the synthesis of 57[8]

Under N_2 atmosphere, a 25 mL schlenk tube was charged with LTMP (0.4 mmol) and 5 mL THF the tube was then cooled to 0 ºC. A solution of **34** (0.15 g, 0.4 mmol) in THF were added. The reaction mixture was allowed to stir for 5 minutes at 0 ºC. Next, a solution of diiodomethane (2 equiv., 0.8 mmol) in 2 mL THF was added drop wisely at 0 ºC. The reaction vial was allowed to warm to 60 ºC and stir for additional 2 hours. Upon completion, the reaction mixture was concentrated under reduced pressure and the crude product was isolated on silica gel using flash chromatography (PE: EA = 50:1 as eluent).

Procedure for the synthesis of 58[9]

Under N_2 atmosphere, a 25 mL schlenk tube was charged with LTMP (0.55 mmol) and 5 mL THF. The tube was then cooled to 0 ºC and a solution of **41** (0.19 g, 0.5 mmol) in THF were added. The reaction mixture was allowed to stir for 5 minutes at 0 ºC. Next, the reaction mixture was transferred drop wisely via syringe to a second tube containing a solution of 1,3-dibromopropane (191 μL, 1.0 mmol) in 5 mL THF at 0 °C. The reaction mixture was allowed to stir at 0 °C for 15 min, then warmed to room temperature, filtered through a silica gel plug, washed with diethyl ether, and concentrated in vacuo. The crude intermediate was isolated on silica gel using flash chromatography (PE: EA = 50:1 as eluent). Then to the THF solution of isolated intermediate was added 3 equiv. of *t*BuONa and the solution was stirred at room temperature for 14 h. Upon completion, the reaction mixture was diluted with diethyl ether (5 mL), filtered through a silica gel plug, rinsed with diethyl ether, and concentrated in vacuo. The final product was isolated on silica gel using flash chromatography (PE: EA = 80:1 as eluent).

Procedure for the synthesis of 59[9]

Under N_2 atmosphere, a 25 mL schlenk tube was charged with **3** (0.36 g, 1 mmol) and 5 mL THF, the tube was then cooled to 0 ºC. To the above solution was added LTMP (0.18 g, 1.2 mmol). After 5 minutes, 1,4-bis(bromomethyl)benzene (0.35 g, 1.2 mmol) was added and the reaction mixture was refluxed for 5 h and filtered through a pad of silica gel with diethyl ether (20 mL). The solvent was evaporated, and the crude product was isolated on silica gel using flash chromatography (PE: EA = 10:1 as eluent).

Initial Condition Optimization Tables

Table S1. Zr-catalyzed synthesis of 1,1-diborylalkanes from alkene: bases effect[a]

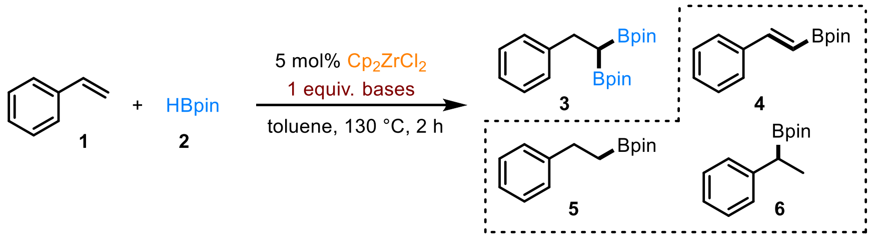

Entry	Bases	Yields [%][b]			
		3	4	5	6
1	^{t}BuONa	52	20	23	3
2	^{t}BuOK	5	59	13	5
3	KOAc	3	89	3	3
4	CsF	24	58	12	3
5	$NaBH_4$	1	57	20	1
6	^{t}BuOLi	87	1	8	1
7	MeONa	60	2	19	11
8	K_2CO_3	1	69	11	<1
9	NaOH	24	5	62	3
10	KOH	5	56	19	4
**11**	**MeOLi**	**92**	**1**	**5**	**1**

[a]Reaction conditions: 0.2 mmol **1**, 0.6 mmol HBpin, 1 equiv. of different bases, 5 mol% Cp_2ZrCl_2, 1 mL toluene in 15 mL pressure tube and heated at 130 ºC for 2 h; [b]yields were determined by GC-MS using *n*-dodecane as internal standard.

Table S2. Zr-catalyzed synthesis of 1,1-diborylalkanes from alkene: MeOLi amount effect[a]

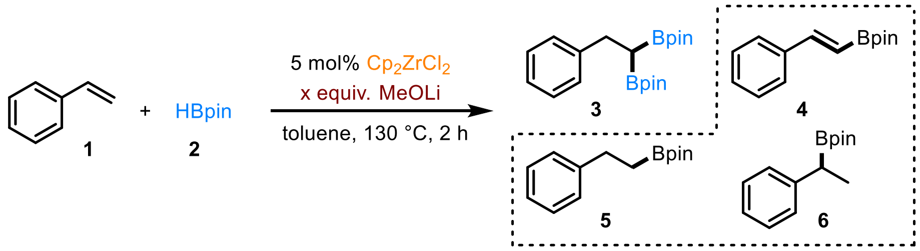

Entry	MeOLi (x equiv.)	Yields [%][b]			
		**3**	**4**	**5**	**6**
1	0	0	0	13	0
2	0.25	70	12	7	1
3	0.5	89	0	2	2
**4**	**1.0**	**92**	**1**	**5**	**1**
5	1.5	84	0	9	2
6	2.0	82	1	10	2
7[c]	1.0	0	0	84	16

[a]Reaction conditions: 0.2 mmol **1**, 0.6 mmol HBpin, 5 mol% Cp_2ZrCl_2, 1 mL toluene in 15 mL pressure tube and heated at 130 ºC for 2 h; [b]yields were determined by GC-MS using *n*-dodecane as internal standard; [c]reaction was performed without Cp_2ZrCl_2.

Table S3. Zr-catalyzed synthesis of 1,1-diborylalkanes from alkene: solvent effect[a]

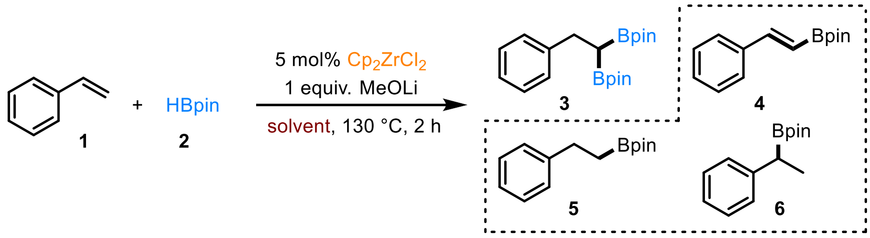

Entry	Solvents	Yields [%][b]			
		**3**	**4**	**5**	**6**
1	Dioxane	48	0	41	3
2	THF	22	0	61	5
3	DMSO	1	0	14	3
4	DCM	10	0	0	87
5	DMF	1	0	0	3
6	MTBE	51	1	42	3
**7**	**Tol**	**92**	**1**	**5**	**1**
8	MeCN	1	0	2	0
9	DGDE	43	0	35	12
10	Hexane	86	1	8	1

[a]Reaction conditions: 0.2 mmol **1**, 0.6 mmol HBpin, 1 equiv. of MeOLi, 5 mol% Cp_2ZrCl_2, 1 mL of different solvents in 15 mL pressure tube and heated at 130 ºC for 2 h; [b]yields were determined by GC-MS using *n*-dodecane as internal standard. DGDE = diethylene glycol diethyl ether.

Table S4. Zr-catalyzed synthesis of 1,1-diborylalkanes from alkene: temperature effect[a]

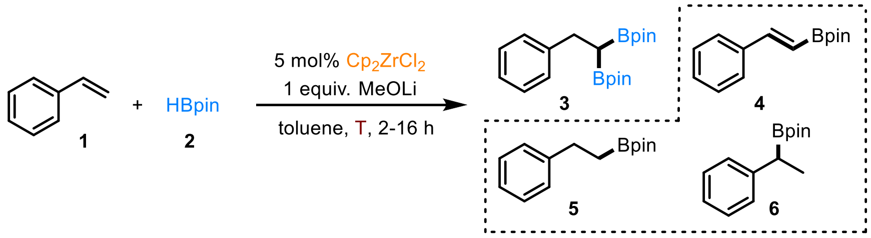

Entry	Temperature [°C]	Yields [%][b]			
		**3**	**4**	**5**	**6**
1	130	92	1	5	1
**2**	**100**	**88**	**2**	**6**	**1**
3	80	81	4	8	1
4	60	10	35	9	1
5	50	0	0	0	0

[a]Reaction conditions: 0.2 mmol **1**, 0.6 mmol HBpin, 1 equiv. of MeOLi, 5 mol% Cp_2ZrCl_2, 1 mL of toluene in 15 mL pressure tube and heated at the temperature shown above for 2-16 h; [b]yields were determined by GC-MS using *n*-dodecane as internal standard.

Table S5. Zr-catalyzed synthesis of 1,1-diborylalkanes from alkene: catalyst loading effect at different temperature[a]

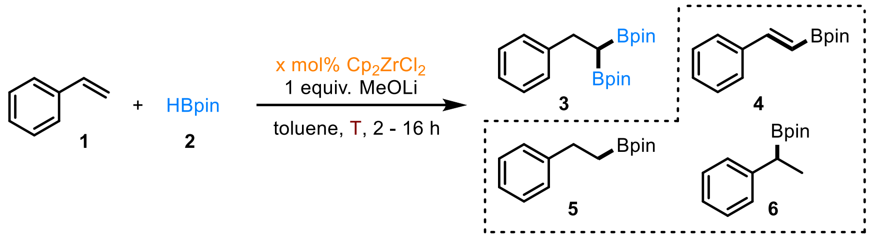

Entry	x	Temperature [°C]	Yields [%][b]			
			**3**	**4**	**5**	**6**
1	5	130	92	1	5	1
2	2	130	74	1	22	2
**3**	**5**	**100**	**88**	**2**	**6**	**1**
4	2	100	69	1	24	3

[a]Reaction conditions: 0.2 mmol **1**, 0.6 mmol HBpin, 1 equiv. of MeOLi, 1 mL of toluene in 15 mL pressure tube and heated at the temperature shown above for 2-16 h; [b]yields were determined by GC-MS using *n*-dodecane as internal standard.

Table S6. Zr-catalyzed synthesis of 1,1-diborylalkanes from alkene: catalyst effect[a]

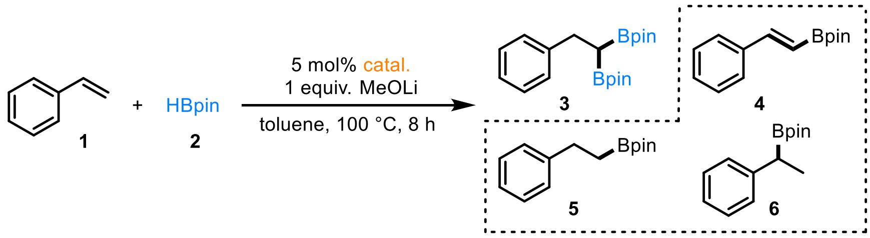

Entry	Catal.	Yields [%][b]			
		3	4	5	6
**1**	**Cp_2ZrCl_2**	**88**	**2**	**6**	**1**
2	$CpZrCl_3$	3	1	84	12
3	Cp_2ZrMe_2	63	0	34	2
4	Cp_2ZrHCl	87	1	7	1

[a]Reaction conditions: 0.2 mmol **1**, 0.6 mmol HBpin, 1 equiv. of MeOLi, 5 mol% cat., 1 mL of toluene in 15 mL pressure tube and heated at 100 ºC for 8 h; [b]yields were determined by GC-MS using *n*-dodecane as internal standard.

Table S7. Zr-catalyzed synthesis of 1,1-diborylalkanes from alkene: different methoxide effect[a]

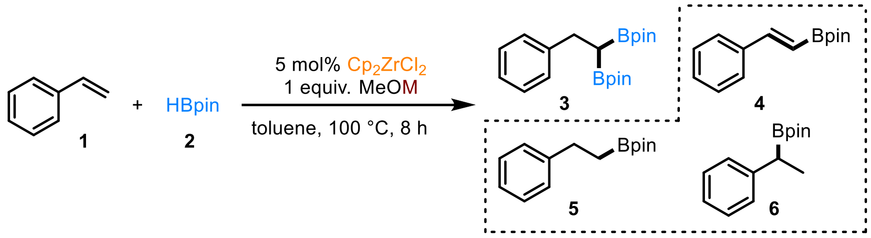

Entry	MeOM	Yields [%][b]			
		3	4	5	6
**1**	**MeOLi**	**88**	**2**	**6**	**1**
2	MeONa	29	47	11	1
3	MeOK	16	26	54	2
4[c]	$Mg(OMe)_2$	7	9	13	0

[a]Reaction conditions: 0.2 mmol **1**, 0.6 mmol HBpin, 1 equiv. of MeOM, 5 mol% Cp_2ZrCl_2, 1 mL of toluene in 15 mL pressure tube and heated at 100 ºC for 8 h; [b]yields were determined by GC-MS using *n*-dodecane as internal standard; [c]0.5 equiv. of $Mg(OMe)_2$ was used.

Mechanism Study Figures

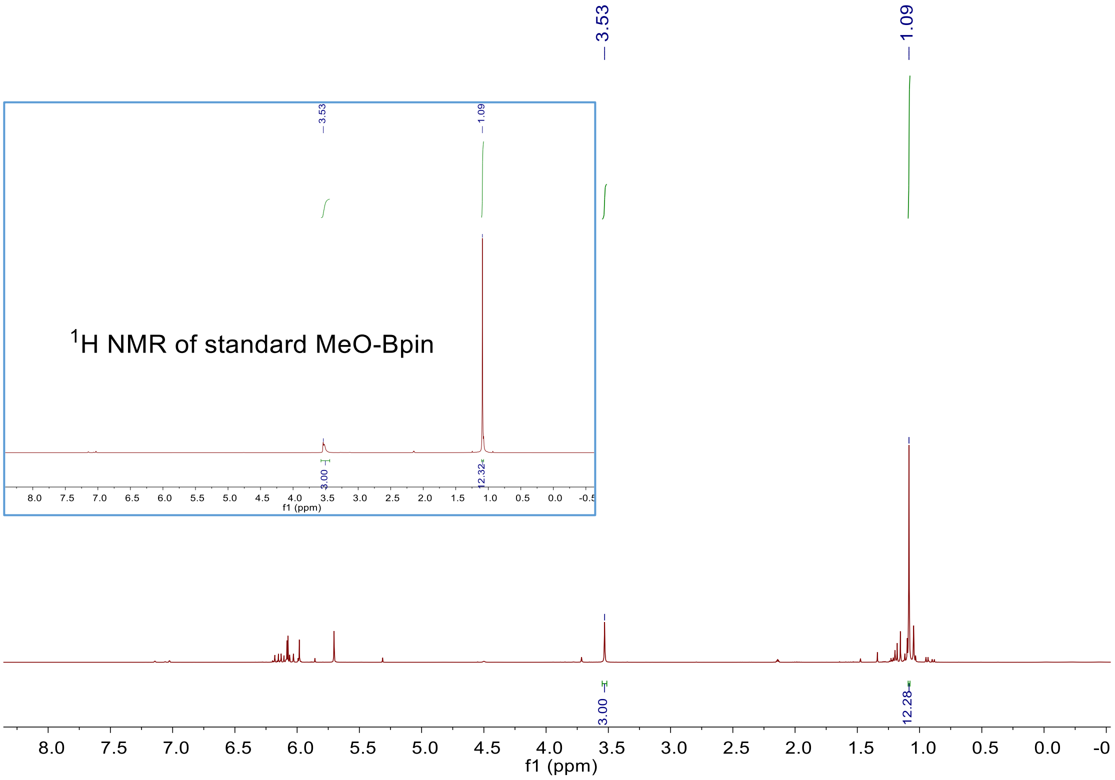

Figure S1. ^{1}H NMR spectrum of 0.1 mmol of Cp_2ZrCl_2, 0.2 mmol of MeOLi and 0.2 mmol of HBpin after heating at 100 °C for 2 h showed the formation of MeO-Bpin. Inserted figure of standard ^{1}H NMR spectrum of MeO-Bpin.

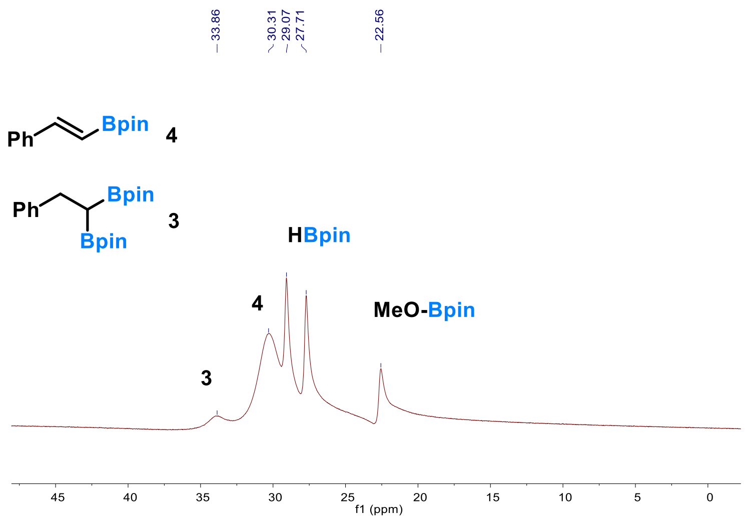

Figure S2. ^{11}B NMR spectrum of the standard catalytic reaction conditions with styrene performed at 0.2 mmol scale in a J-Young NMR tube after heating at 100 °C for 1 h.

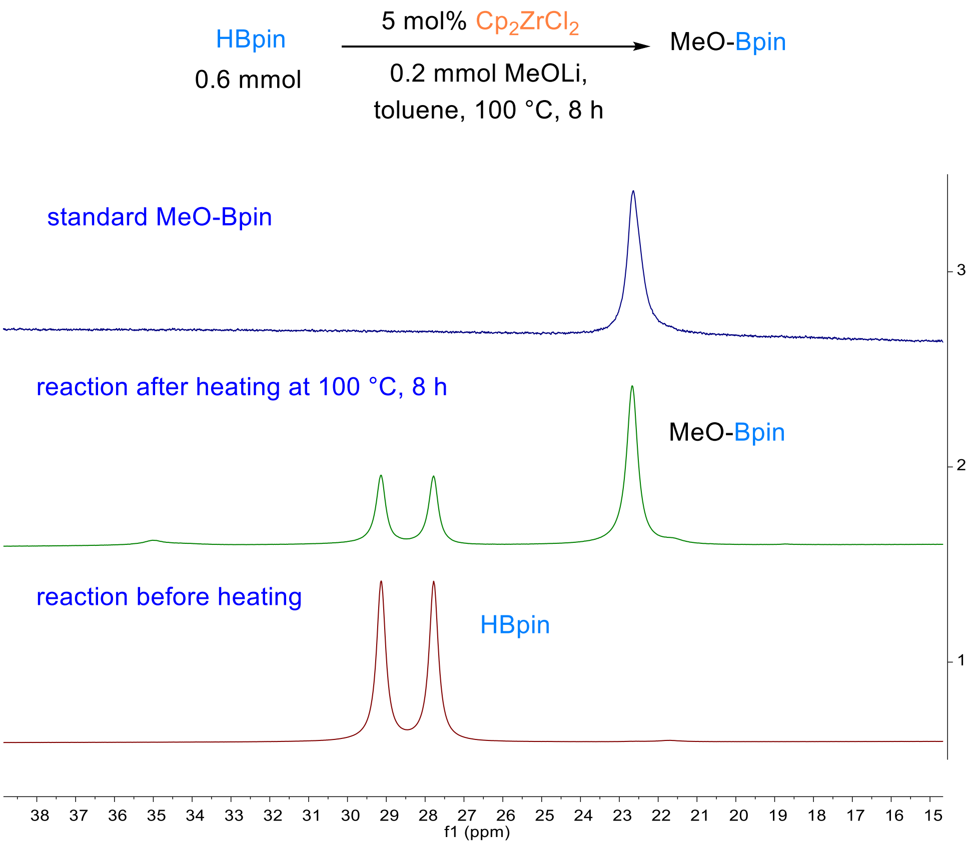

Figure S3. ^{11}B NMR spectrum of standard MeO-Bpin and catalytic reaction without styrene showed the formation of MeO-Bpin.

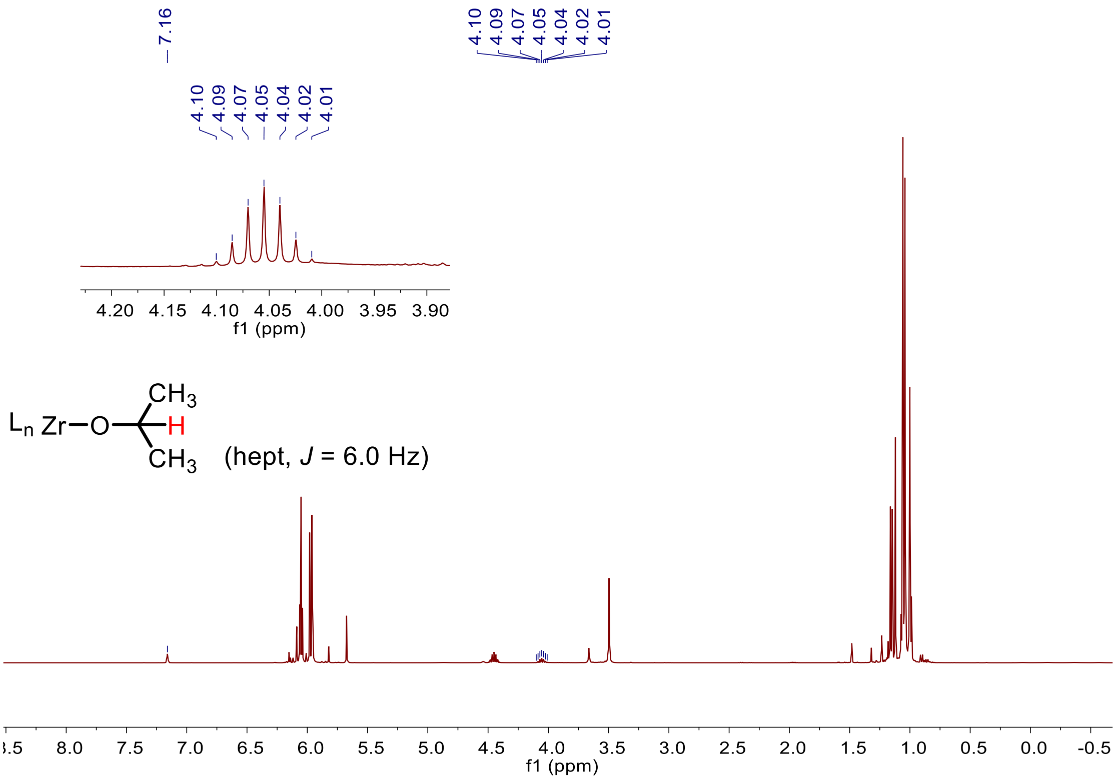

Figure S4. ^{1}H NMR spectrum of 0.1 mmol Cp_2ZrCl_2, 0.2 mmol MeOLi and 0.2 mmol HBpin with 0.2 mmol acetone in *d*$_6$-benzene after heating at 100 °C for 2 h. The hept peaks at 4.05 ppm with J value of 6.0 Hz indicates the formation of Zr-O*i*Pr from Zr-H trapped by acetone.

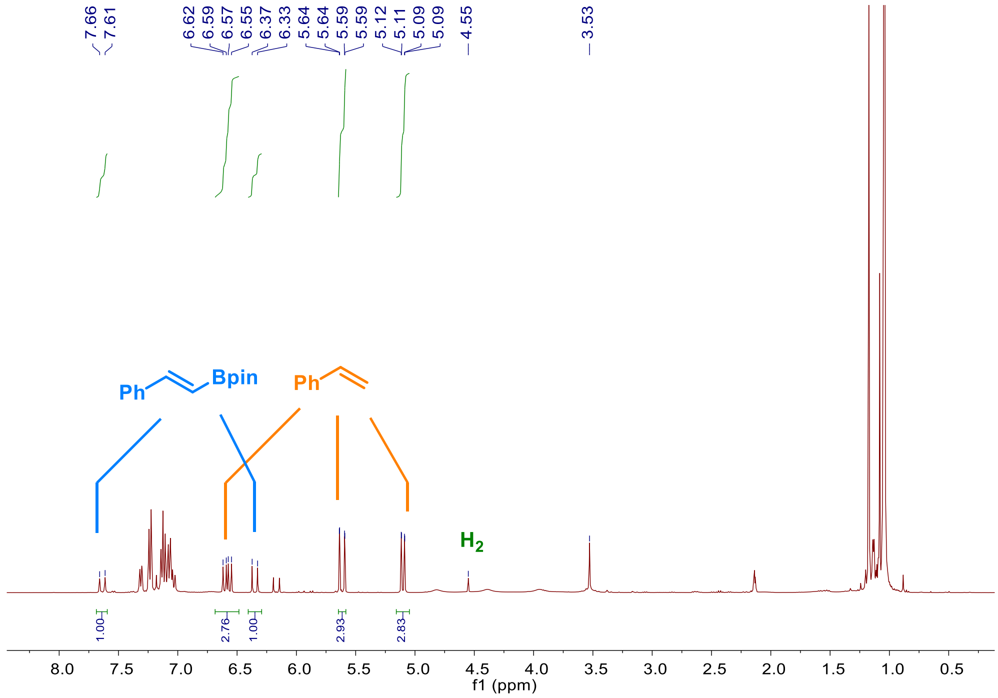

Figure S5. ^{1}H NMR spectrum of standard catalytic reaction conditions performed in d_8-toluene in J-Young NMR tube and monitored after 0.5 h of heat at 100 °C shows clearly the formation of H_2 at 4.55 ppm and the conversion of styrene to intermediate vinyl boronate ester **4**.

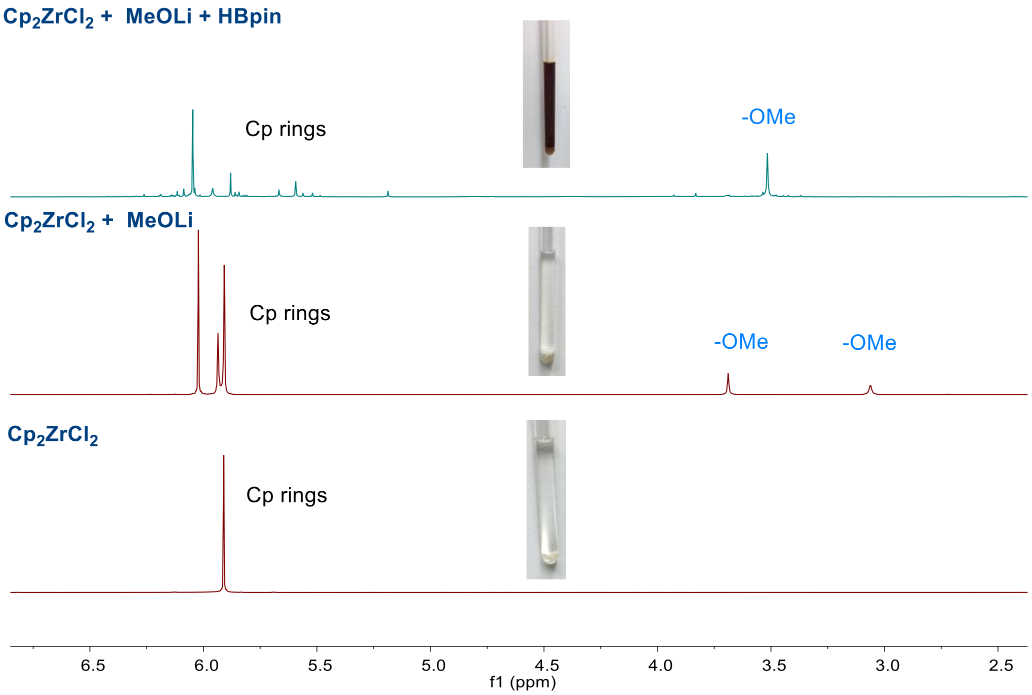

Figure S6. ^{1}H NMR spectra of 0.1 mmol of Cp_2ZrCl_2 in 0.5 mL d_8-toluene solution and sequential adding 0.2 mmol of MeOLi and 0.2 mmol of HBpin to the above solution. We can see that new “Cp” species was formed and the MeO^- also played a role during the transformation which eventually all changed to MeO-Bpin.

Synthetic Application of 1,1-Diborylalkanes

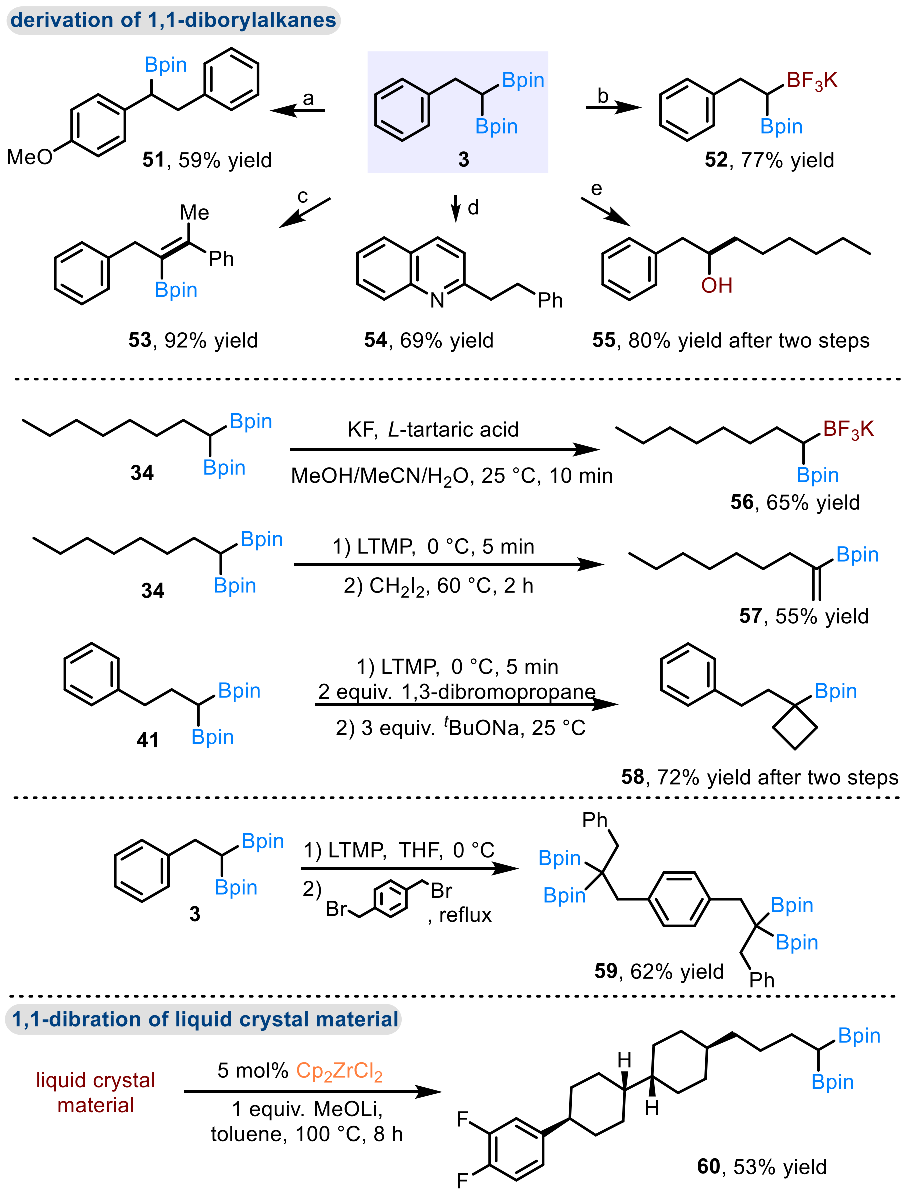

Scheme S1. a) Pd(dba)$_2$, ^{t}Bu$_3$P, KOH, 4-iodoanisole, THF, 25 °C, 12 h; b) KF, *L*-tartaric acid, MeOH/MeCN/H$_2$O, 25 °C, 10 min; c) LTMP, 0 °C, 5 min then acetophenone (LTMP = Lithium Tetramethylpiperidide); d) quinoline-*N*-oxide, MeONa, toluene, 80 °C, 16 h; e) MeONa, 1-bromohexane, 25 °C, 16 h then H$_2$O$_2$/NaOH, 0-25 °C, 3 h.

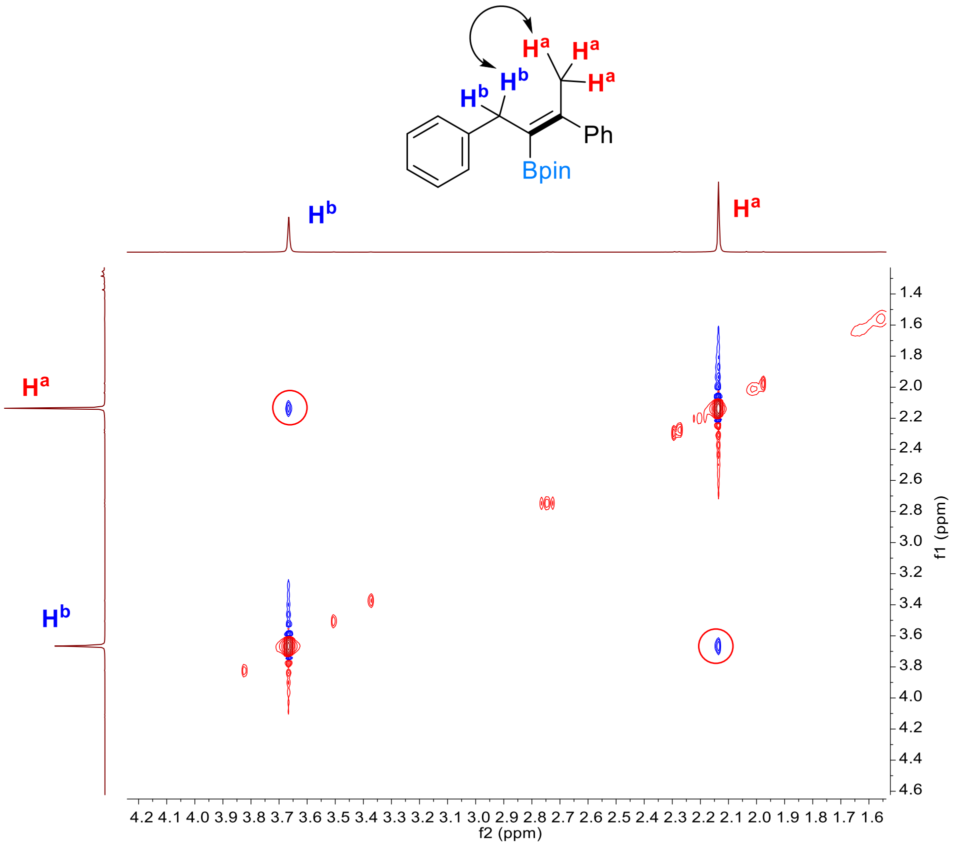

Figure S7. NOESY spectra of **53.** The configuration of **53** was confirmed to be *E*-stereoisomer as H^a at 2.13 ppm has NOESY signals with H^b at 3.66 ppm.

Products Characterization

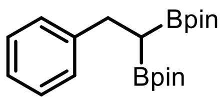

2,2'-(2-phenylethane-1,1-diyl)bis(4,4,5,5-tetramethyl-1,3,2-dioxaborolane) **3**

Eluent: petroleum ether/ethyl acetate (10:1). Colorless oil. **^{1}H NMR** (400 MHz, $CDCl_3$) δ 7.25 – 7.18 (m, 4H), 7.13 – 7.08 (m, 1H), 2.88 (d, *J* = 8.4 Hz, 2H), 1.18 (s, 12H), 1.17 (s, 13H), the "CH" signals were overlapped with the intensive signal of four Me groups. **^{13}C NMR** (101 MHz, $CDCl_3$) δ 144.4, 128.3, 127.9, 125.3, 83.0, 31.2, 24.7, 24.5. **^{11}B NMR** (128 MHz, $CDCl_3$) δ 33.62. Spectroscopic data are in agreement with those previously reported.[10]

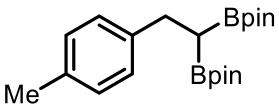

2,2'-(2-(*p*-tolyl)ethane-1,1-diyl)bis(4,4,5,5-tetramethyl-1,3,2-dioxaborolane) **7**

Eluent: petroleum ether/ethyl acetate (10:1). Colorless oil. **^{1}H NMR** (400 MHz, $CDCl_3$) δ 7.13 (d, *J* = 8.0 Hz, 2H), 7.02 (d, *J* = 8.0 Hz, 2H), 2.85 (d, *J* = 8.3 Hz, 2H), 2.28 (s, 3H), 1.19 (s, 12H), 1.18 (s, 12H), 1.12 (d, *J* = 8.3 Hz, 1H). **^{13}C NMR** (101 MHz, $CDCl_3$) δ 141.3, 134.5, 128.6, 128.1, 83.0, 30.8, 24.7, 24.5, 21.0. **^{11}B NMR** (128 MHz, $CDCl_3$) δ 33.45. Spectroscopic data are in agreement with those previously reported.[10]

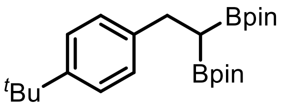

2,2'-(2-(4-(*tert*-butyl)phenyl)ethane-1,1-diyl)bis(4,4,5,5-tetramethyl-1,3,2-dioxaborolane) **8**

Eluent: petroleum ether/ethyl acetate (10:1). Colorless oil. **^{1}H NMR** (400 MHz, $CDCl_3$) δ 7.32 (d, *J* = 8.3 Hz, 2H), 7.16 (d, *J* = 8.3 Hz, 2H), 3.10 (s, 2H), 1.26 (s, 9H), 1.14 (s, 25H), the "CH" signals were overlapped with the intensive signal of eight Me groups. **^{13}C NMR** (101 MHz, $CDCl_3$) δ 147.6, 141.3, 129.2, 124.0, 82.8, 34.1, 32.7, 31.4, 24.6. **^{11}B NMR** (128 MHz, $CDCl_3$) δ 33.25. Spectroscopic data are in agreement with those previously reported.[10]

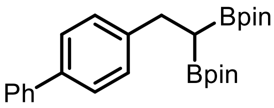

2,2'-(2-([1,1'-biphenyl]-4-yl)ethane-1,1-diyl)bis(4,4,5,5-tetramethyl-1,3,2-dioxaborolane) **9**

Eluent: petroleum ether/ethyl acetate (15:1). Pale yellow solid. **^{1}H NMR** (400 MHz, $CDCl_3$) δ 7.58 – 7.54 (m, 2H), 7.46 (d, *J* = 8.1 Hz, 2H), 7.40 (t, *J* = 7.7 Hz, 2H), 7.31 (d, *J* = 8.4 Hz, 3H), 2.92 (d, *J* = 8.3 Hz, 2H), 1.19 (s, 12H), 1.18 (s, 13H), the "CH" signals were overlapped with the intensive signal of four Me groups. **^{13}C NMR** (101 MHz, $CDCl_3$) δ 143.7, 141.4, 138.3, 128.8, 128.6, 126.9, 126.8, 126.7, 83.1, 30.97, 24.8, 24.5. **^{11}B NMR** (128 MHz, $CDCl_3$) δ 33.27. Spectroscopic data are in agreement with those previously reported.[10]

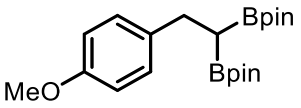

2,2'-(2-(4-methoxyphenyl)ethane-1,1-diyl)bis(4,4,5,5-tetramethyl-1,3,2-dioxaborolane) **10**

Eluent: petroleum ether/ethyl acetate (10:1). Pale yellow oil. **^{1}H NMR** (400 MHz, $CDCl_3$) δ 7.14 (d, *J* = 8.6 Hz, 2H), 6.76 (d, *J* = 8.6 Hz, 2H), 3.75 (s, 3H), 2.82 (d, *J* = 8.3 Hz, 2H), 1.18 (s, 13H), 1.17 (s, 13H), the "CH" signals were overlapped with the intensive signal of four Me groups. **^{13}C NMR** (101 MHz, $CDCl_3$) δ 157.4, 136.7, 129.2, 113.3, 83.0, 55.2, 30.4, 24.8, 24.5. **^{11}B NMR** (128 MHz, $CDCl_3$) δ 33.41. Spectroscopic data are in agreement with those previously reported.[11]

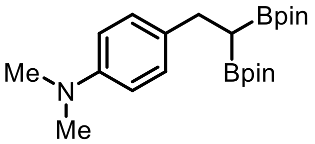

4-(2,2-bis(4,4,5,5-tetramethyl-1,3,2-dioxaborolan-2-yl)ethyl)-*N*,*N*-dimethylaniline **11**

Eluent: petroleum ether/ethyl acetate (5:1). Pale yellow oil. **^{1}H NMR** (400 MHz, $CDCl_3$) δ 7.11 (d, *J* = 8.5 Hz, 2H), 6.66 (d, *J* = 8.6 Hz, 2H), 2.87 (s, 6H), 2.80 (d, *J* = 8.3 Hz, 2H), 1.19 (s, 12H), 1.18 (s, 12H), 1.12 (t, *J* = 8.2 Hz, 1H). **^{13}C NMR** (101 MHz, $CDCl_3$) δ 148.9, 133.2, 128.8, 82.9, 41.1, 30.3, 24.8, 24.5. **^{11}B NMR** (128 MHz, $CDCl_3$) δ 33.20. Spectroscopic data are in agreement with those previously reported.[11]

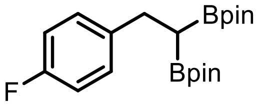

2,2'-(2-(4-fluorophenyl)ethane-1,1-diyl)bis(4,4,5,5-tetramethyl-1,3,2-dioxaborolane) **13**

Eluent: petroleum ether/ethyl acetate (10:1). Pale yellow oil. **^{1}H NMR** (400 MHz, $CDCl_3$) δ 7.22 – 7.12 (m, 2H), 6.90 (t, *J* = 8.8 Hz, 2H), 2.84 (d, *J* = 8.4 Hz, 2H), 1.18 (s, 12H), 1.17 (s, 12H), 1.12 (t, *J* = 8.4 Hz, 1H). **^{13}C NMR** (101 MHz, $CDCl_3$) δ 161.1 (d, *J* = 242.2 Hz), 140.1 (d, *J* = 3.0

Hz), 129.6 (d, J = 7.7 Hz), 114.5 (d, J = 20.9 Hz), 83.1, 30.5, 24.8, 24.5. **^{11}B NMR** (128 MHz, $CDCl_3$) δ 34.00. **^{19}F NMR** (376 MHz, $CDCl_3$) δ -118.65, -118.66, -118.67, -118.68, -118.70, -118.71, -118.72. Spectroscopic data are in agreement with those previously reported.[10]

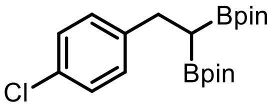

2,2'-(2-(4-chlorophenyl)ethane-1,1-diyl)bis(4,4,5,5-tetramethyl-1,3,2-dioxaborolane) **14**

Eluent: petroleum ether/ethyl acetate (10:1). Colorless oil. **^{1}H NMR** (400 MHz, $CDCl_3$) δ 7.20 – 7.13 (m, 4H), 2.83 (d, J = 8.3 Hz, 2H), 1.18 (s, 12H), 1.17 (s, 12H), 1.11 (t, J = 8.3 Hz, 1H). **^{13}C NMR** (101 MHz, $CDCl_3$) δ 143.0, 131.0, 129.7, 128.0, 83.2, 30.7, 24.8, 24.5. **^{11}B NMR** (128 MHz, $CDCl_3$) δ 33.61. Spectroscopic data are in agreement with those previously reported.[12]

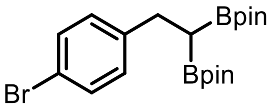

2,2'-(2-(4-bromophenyl)ethane-1,1-diyl)bis(4,4,5,5-tetramethyl-1,3,2-dioxaborolane) **15**

Eluent: petroleum ether/ethyl acetate (10:1). White solid. **^{1}H NMR** (400 MHz, $CDCl_3$) δ 7.32 (d, J = 8.4 Hz, 2H), 7.10 (d, J = 8.4 Hz, 2H), 2.81 (d, J = 8.3 Hz, 2H), 1.17 (s, 12H), 1.16 (s, 12H), 1.10 (t, J = 8.3 Hz, 1H). **^{13}C NMR** (101 MHz, $CDCl_3$) δ143.4, 130.9, 130.1, 119.0, 83.1, 30.7, 24.7, 24.4. **^{11}B NMR** (128 MHz, $CDCl_3$) δ 33.26. Spectroscopic data are in agreement with those previously reported.[13]

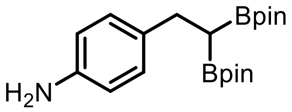

4-(2,2-bis(4,4,5,5-tetramethyl-1,3,2-dioxaborolan-2-yl)ethyl)aniline **16**

Eluent: petroleum ether/ethyl acetate (5:1). Pale yellow oil. **^{1}H NMR** (400 MHz, $CDCl_3$) δ 7.02 (d, J = 8.4 Hz, 2H), 6.57 (d, J = 8.4 Hz, 2H), 3.48 (s, 2H), 2.77 (d, J = 8.3 Hz, 2H), 1.18 (s, 12H), 1.17 (s, 12H), 1.10 (t, J = 8.3 Hz, 1H). **^{13}C NMR** (101 MHz, $CDCl_3$) δ 143.7, 134.9, 129.1, 115.0, 83.0, 30.4, 24.8, 24.5. **^{11}B NMR** (128 MHz, $CDCl_3$) δ 33.26. **HRMS** calcd for $C_{20}H_{34}B_2NO_4$: 374.2668 $[M+H]^+$, found: 374.2671.

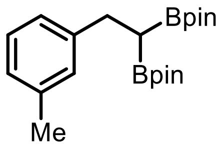

2,2'-(2-(*m*-tolyl)ethane-1,1-diyl)bis(4,4,5,5-tetramethyl-1,3,2-dioxaborolane) **17**

Eluent: petroleum ether/ethyl acetate (10:1). Colorless oil. **^{1}H NMR** (400 MHz, $CDCl_3$) δ 7.09 (d, *J* = 7.5 Hz, 1H), 7.06 (s, 1H), 7.03 (d, *J* = 7.6 Hz, 1H), 6.92 (d, *J* = 7.4 Hz, 1H), 2.85 (d, *J* = 8.3 Hz, 2H), 2.29 (s, 3H), 1.19 (s, 12H), 1.18 (s, 12H), 1.13 (t, *J* = 8.3 Hz, 1H). **^{13}C NMR** (101 MHz, $CDCl_3$) δ 144.4, 137.2, 129.2, 127.8, 126.0, 125.3, 83.0, 31.21, 24.7, 24.5, 21.3. **^{11}B NMR** (128 MHz, $CDCl_3$) δ 32.98. Spectroscopic data are in agreement with those previously reported.[11]

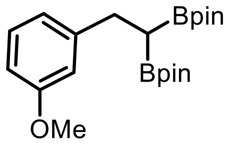

2,2'-(2-(3-methoxyphenyl)ethane-1,1-diyl)bis(4,4,5,5-tetramethyl-1,3,2-dioxaborolane) **18**

Eluent: petroleum ether/ethyl acetate (10:1). Colorless oil. **^{1}H NMR** (400 MHz, $CDCl_3$) δ 7.12 (t, *J* = 7.9 Hz, 1H), 6.87 – 6.76 (m, 2H), 6.66 (dd, *J* = 8.2, 2.6 Hz, 1H), 3.76 (s, 3H), 2.86 (d, *J* = 8.3 Hz, 2H), 1.18 (s, 12H), 1.17 (s, 13H), the "CH" signals were overlapped with the intensive signal of four Me groups. **^{13}C NMR** (101 MHz, $CDCl_3$) δ 159.3, 146.1, 128.8, 120.7, 113.7, 111.1, 83.0, 55.0, 31.3, 24.7, 24.5. **^{11}B NMR** (128 MHz, $CDCl_3$) δ 33.32. Spectroscopic data are in agreement with those previously reported.[13]

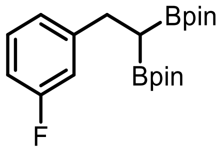

2,2'-(2-(3-fluorophenyl)ethane-1,1-diyl)bis(4,4,5,5-tetramethyl-1,3,2-dioxaborolane) **19**

Eluent: petroleum ether/ethyl acetate (20:1). Colorless oil. **^{1}H NMR** (400 MHz, $CDCl_3$) δ 7.15 (td, *J* = 7.9, 6.1 Hz, 1H), 7.02 – 6.90 (m, 2H), 6.83 – 6.75 (m, 1H), 2.85 (d, *J* = 8.4 Hz, 2H), 1.18 (s, 12H), 1.16 (s, 13H), , the "CH" signals were overlapped with the intensive signal of four Me groups. **^{13}C NMR** (101 MHz, $CDCl_3$) δ 162.7 (d, *J* = 244.2 Hz), 147.1 (d, *J* = 7.1 Hz), 129.3 (d, *J* = 8.3 Hz), 123.9 (d, *J* = 2.6 Hz), 115.2 (d, *J* = 21.0 Hz), 112.1 (d, *J* = 21.0 Hz), 83.1, 31.1 (d,

J = 1.8 Hz), 24.7, 24.4. **^{11}B NMR** (128 MHz, $CDCl_3$) δ 33.27. **^{19}F NMR** (376 MHz, $CDCl_3$) δ -114.52 – -114.66 (m). **HRMS** calcd for $C_{20}H_{31}B_2FNaO_4$: 399.2285 $[M+Na]^+$, found: 399.2294.

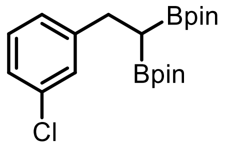

2,2'-(2-(3-chlorophenyl)ethane-1,1-diyl)bis(4,4,5,5-tetramethyl-1,3,2-dioxaborolane) **20**

Eluent: petroleum ether/ethyl acetate (10:1). Colorless oil. **^{1}H NMR** (400 MHz, $CDCl_3$) δ 7.26 – 7.24 (m, 1H), 7.18 – 7.05 (m, 3H), 2.84 (d, J = 8.3 Hz, 2H), 1.19 (s, 12H), 1.18 (s, 12H), 1.10 (t, J = 8.3 Hz, 1H). **^{13}C NMR** (101 MHz, $CDCl_3$) δ 146.6, 133.6, 129.2, 128.6, 126.5, 125.5, 83.2, 31.1, 24.8, 24.5. **^{11}B NMR** (128 MHz, $CDCl_3$) δ 33.14. **HRMS** calcd for $C_{20}H_{32}B_2ClO_4$: 393.2170 $[M+H]^+$, found: 393.2182.

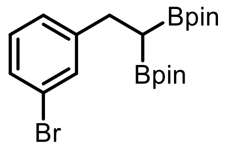

2,2'-(2-(3-bromophenyl)ethane-1,1-diyl)bis(4,4,5,5-tetramethyl-1,3,2-dioxaborolane) **21**

Eluent: petroleum ether/ethyl acetate (10:1). Pale yellow oil. **^{1}H NMR** (400 MHz, $CDCl_3$) δ 7.41 (t, J = 1.9 Hz, 1H), 7.23 (dt, J = 7.8, 1.7 Hz, 1H), 7.15 – 7.11 (m, 1H), 7.07 (t, J = 7.7 Hz, 1H), 2.82 (d, J = 8.3 Hz, 2H), 1.18 (s, 12H), 1.17 (s, 12H), 1.09 (t, J = 8.3 Hz, 1H). **^{13}C NMR** (101 MHz, $CDCl_3$) δ 146.9, 131.5, 129.5, 128.4, 126.9, 122.0, 83.2, 31.0, 24.8, 24.5. **^{11}B NMR** (128 MHz, $CDCl_3$) δ 32.89. **HRMS** calcd for $C_{20}H_{31}B_2BrNaO_4$: 459.1484 $[M+Na]^+$, found: 459.1496.

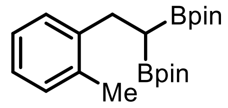

2,2'-(2-(*o*-tolyl)ethane-1,1-diyl)bis(4,4,5,5-tetramethyl-1,3,2-dioxaborolane) **22**

Eluent: petroleum ether/ethyl acetate (10:1). Pale yellow oil. **^{1}H NMR** (400 MHz, $CDCl_3$) δ 7.23 (d, J = 7.1 Hz, 1H), 7.12 – 6.97 (m, 3H), 2.86 (d, J = 8.0 Hz, 2H), 2.31 (s, 3H), 1.19 (d, J = 2.0 Hz, 24H), 1.15 (t, J = 7.9 Hz, 1H). **^{13}C NMR** (101 MHz, $CDCl_3$) δ 142.5, 136.0, 129.8, 128.4, 125.5, 125.4, 83.1, 28.4, 24.8, 24.5, 19.4. **^{11}B NMR** (128 MHz, $CDCl_3$) δ 33.35. Spectroscopic data are in agreement with those previously reported.[13]

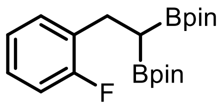

2,2'-(2-(2-fluorophenyl)ethane-1,1-diyl)bis(4,4,5,5-tetramethyl-1,3,2-dioxaborolane) **24**

Eluent: petroleum ether/ethyl acetate (10:1). Colorless oil. **^{1}H NMR** (400 MHz, $CDCl_3$) δ 7.27 (td, *J* = 7.5, 1.8 Hz, 1H), 7.10 (tdd, *J* = 7.4, 5.2, 1.8 Hz, 1H), 7.02 – 6.90 (m, 2H), 2.90 (d, *J* = 8.3 Hz, 2H), 1.18 (s, 13H), the "CH" signals were overlapped with the intensive signal of four Me groups, 1.17 (s, 12H). **^{13}C NMR** (101 MHz, $CDCl_3$) δ 161.1 (d, *J* = 245.2 Hz), 131.1 (d, *J* = 15.9 Hz), 130.5 (d, *J* = 5.3 Hz), 127.0 (d, *J* = 8.1 Hz), 123.4 (d, *J* = 3.6 Hz), 114.9 (d, *J* = 22.2 Hz), 83.1, 24.8, 24.5. **^{11}B NMR** (128 MHz, $CDCl_3$) δ 33.83. **^{19}F NMR** (376 MHz, $CDCl_3$) δ -117.89, -117.91, -117.92, -117.93, -117.94, -117.95. **HRMS** calcd for $C_{20}H_{32}B_2FO_4$: 377.2465 $[M+H]^+$, found: 377.2474.

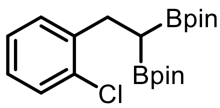

2,2'-(2-(2-chlorophenyl)ethane-1,1-diyl)bis(4,4,5,5-tetramethyl-1,3,2-dioxaborolane) **25**

Eluent: petroleum ether/ethyl acetate (10:1). Colorless oil. **^{1}H NMR** (400 MHz, $CDCl_3$) δ 7.33 (dd, *J* = 7.5, 1.9 Hz, 1H), 7.27 (dd, *J* = 7.8, 1.5 Hz, 1H), 7.13 – 7.03 (m, 2H), 2.98 (d, *J* = 8.0 Hz, 2H), 1.19 (s, 12H), 1.18 (s, 13H), the "CH" signals were overlapped with the intensive signal of four Me groups. **^{13}C NMR** (101 MHz, $CDCl_3$) δ 141.8, 134.0, 130.3, 129.2, 126.8, 126.2, 83.1, 29.1, 24.8, 24.5. **^{11}B NMR** (128 MHz, $CDCl_3$) δ 33.26. **HRMS** calcd for $C_{20}H_{32}B_2ClO_4$: 393.2170 $[M+H]^+$, found: 393.2170.

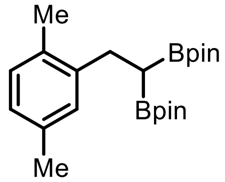

2,2'-(2-(2,5-dimethylphenyl)ethane-1,1-diyl)bis(4,4,5,5-tetramethyl-1,3,2-dioxaborolane) **26**

Eluent: petroleum ether/ethyl acetate (10:1). Pale yellow oil. **^{1}H NMR** (400 MHz, $CDCl_3$) δ 7.07 (s, 1H), 6.96 (d, *J* = 7.6 Hz, 1H), 6.84 (d, *J* = 7.5 Hz, 1H), 2.83 (d, *J* = 8.0 Hz, 2H), 2.27 (s, 3H), 2.25 (s, 3H), 1.20 (s, 24H), 1.12 (t, *J* = 8.0 Hz, 1H). **^{13}C NMR** (101 MHz, $CDCl_3$) δ 142.3, 134.6, 132.7, 129.7, 129.4, 126.0, 83.0, 28.4, 24.8, 24.5, 20.9, 18.8. **^{11}B NMR** (128 MHz, $CDCl_3$) δ 33.62. **HRMS** calcd for $C_{22}H_{37}B_2O_4$: 387.2872 $[M+H]^+$, found: 387.2882.

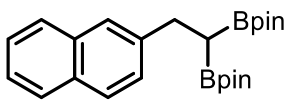

2,2'-(2-(naphthalen-2-yl)ethane-1,1-diyl)bis(4,4,5,5-tetramethyl-1,3,2-dioxaborolane) **28**

Eluent: petroleum ether/ethyl acetate (15:1). Colorless oil. **^{1}H NMR** (400 MHz, $CDCl_3$) δ 7.79 – 7.67 (m, 4H), 7.39 (tt, *J* = 10.0, 3.5 Hz, 3H), 3.06 (d, *J* = 8.3 Hz, 2H), 1.18 (s, 13H), the "CH" signals were overlapped with the intensive signal of four Me groups, 1.18 (s, 12H). **^{13}C NMR** (101 MHz, $CDCl_3$) δ 142.1, 133.5, 131.8, 127.6, 127.49, 127.45, 127.4, 126.0, 125.5, 124.7, 83.1, 31.5, 24.8, 24.5. **^{11}B NMR** (128 MHz, $CDCl_3$) δ 33.38. **HRMS** calcd for $C_{24}H_{35}B_2O_4$: 409.2716 $[M+H]^+$, found: 409.2725.

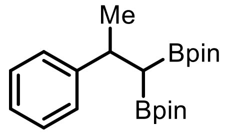

2,2'-(2-phenylpropane-1,1-diyl)bis(4,4,5,5-tetramethyl-1,3,2-dioxaborolane) **29**

Eluent: petroleum ether/ethyl acetate (20:1). Colorless oil. **^{1}H NMR** (400 MHz, $CDCl_3$) δ 7.26 – 7.18 (m, 4H), 7.12 – 7.07 (m, 1H), 3.17 (dq, *J* = 11.8, 6.9 Hz, 1H), 1.26 (dd, *J* = 6.7, 1.8 Hz, 16H), 0.95 (s, 6H), 0.90 (s, 6H). **^{13}C NMR** (101 MHz, $CDCl_3$) δ 149.5, 127.9, 127.0, 125.5, 83.1, 82.7, 37.8, 26.1, 24.9, 24.4, 24.4, 24.2. **^{11}B NMR** (128 MHz, $CDCl_3$) δ 33.80. Spectroscopic data are in agreement with those previously reported.[14]

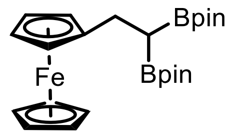

2,2'-(2-ferrocenylethane-1,1-diyl)bis(4,4,5,5-tetramethyl-1,3,2-dioxaborolane) **30**

Eluent: petroleum ether/ethyl acetate (15:1). Yellow solid. **^{1}H NMR** (400 MHz, $CDCl_3$) δ 4.10 (t, *J* = 1.8 Hz, 2H), 4.08 (s, 5H), 3.97 (t, *J* = 1.8 Hz, 2H), 2.59 (d, *J* = 7.8 Hz, 2H), 1.22 (s, 24H), 0.98 (t, *J* = 7.8 Hz, 1H). **^{13}C NMR** (101 MHz, $CDCl_3$) δ 91.7, 83.1, 68.4, 68.3, 66.8, 25.5, 24.8, 24.7. **^{11}B NMR** (128 MHz, $CDCl_3$) δ 34.07. Spectroscopic data are in agreement with those previously reported.[11]

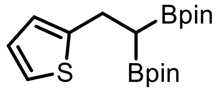

2,2'-(2-(thiophen-2-yl)ethane-1,1-diyl)bis(4,4,5,5-tetramethyl-1,3,2-dioxaborolane) **31**

Eluent: petroleum ether/ethyl acetate (10:1). Pale yellow solid. **^{1}H NMR** (400 MHz, $CDCl_3$) δ 7.03 (d, *J* = 4.2 Hz, 1H), 6.88 – 6.75 (m, 2H), 3.08 (d, *J* = 8.2 Hz, 2H), 1.20 (s, 12H), 1.19 (s, 13H). **^{13}C NMR** (101 MHz, $CDCl_3$) δ 148.0, 126.3, 123.7, 122.4, 83.2, 25.8, 24.8, 24.5. **^{11}B NMR** (128 MHz, $CDCl_3$) δ 33.59. **HRMS** calcd for $C_{18}H_{30}B_2NaO_4S$: 387.1943 $[M+Na]^+$, found: 387.1951.

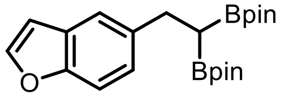

2,2'-(2-(benzofuran-5-yl)ethane-1,1-diyl)bis(4,4,5,5-tetramethyl-1,3,2-dioxaborolane) **32**

Eluent: petroleum ether/ethyl acetate (15:1). Pale yellow oil. **^{1}H NMR** (400 MHz, $CDCl_3$) δ 7.54 (d, *J* = 2.1 Hz, 1H), 7.45 (s, 1H), 7.34 (d, *J* = 8.5 Hz, 1H), 7.20 – 7.16 (m, 1H), 6.68 – 6.65 (m, 1H), 2.97 (d, *J* = 8.3 Hz, 2H), 1.17 – 1.12 (m, 25H), the "CH" signals were overlapped with the intensive signal of eight Me groups. **^{13}C NMR** (101 MHz, $CDCl_3$) δ 153.4, 144.7, 139.0, 127.1, 125.0, 120.3, 110.5, 106.3, 83.0, 31.2, 24.7, 24.4. **^{11}B NMR** (128 MHz, $CDCl_3$) δ 33.25. **HRMS** calcd for $C_{22}H_{36}B_2NO_5$: 416.2774 $[M+NH_4]^+$, found: 416.2782.

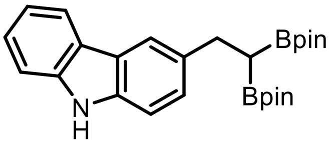

3-(2,2-bis(4,4,5,5-tetramethyl-1,3,2-dioxaborolan-2-yl)ethyl)-9*H*-carbazole **33**

Eluent: petroleum ether/ethyl acetate (5:1). Pale yellow oil. **^{1}H NMR** (400 MHz, $CDCl_3$) δ 8.06 (s, 1H), 8.00 – 7.93 (m, 2H), 7.33 (dd, *J* = 6.3, 1.2 Hz, 2H), 7.25 (dd, *J* = 8.3, 1.6 Hz, 1H), 7.21 – 7.15 (m, 2H), 3.07 (d, *J* = 8.3 Hz, 2H), 1.17 (s, 25H), the "CH" signals were overlapped with the intensive signal of eight Me groups. **^{13}C NMR** (101 MHz, $CDCl_3$) δ 139.8, 137.8, 135.5, 126.6, 125.2, 123.4, 123.1, 120.0, 119.5, 118.9, 110.5, 110.1, 83.0, 31.4, 24.8, 24.5. **^{11}B NMR** (128 MHz, $CDCl_3$) δ 32.92. **HRMS** calcd for $C_{26}H_{36}B_2NO_4$: 448.2825 $[M+H]^+$, found: 448.2841.

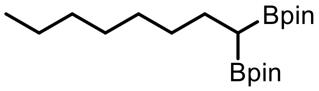

2,2'-(octane-1,1-diyl)bis(4,4,5,5-tetramethyl-1,3,2-dioxaborolane) **34**

Eluent: petroleum ether/ethyl acetate (10:1). Colorless oil. **^{1}H NMR** (400 MHz, $CDCl_3$) δ 1.53 (q, *J* = 7.4 Hz, 2H), 1.29 – 1.23 (m, 10H), 1.22 (s, 12H), 1.21 (s, 12H), 0.86 (t, *J* = 6.8 Hz, 3H), 0.70 (t, *J* = 7.9 Hz, 1H). **^{13}C NMR** (101 MHz, $CDCl_3$) δ 82.8, 32.6, 31.8, 29.6, 29.2, 25.7, 24.8,

24.5, 22.7, 14.1. **^{11}B NMR** (128 MHz, $CDCl_3$) δ 33.92. Spectroscopic data are in agreement with those previously reported.[10-11]

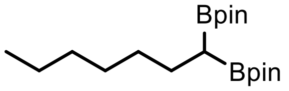

2,2'-(heptane-1,1-diyl)bis(4,4,5,5-tetramethyl-1,3,2-dioxaborolane) **35**

Eluent: petroleum ether/ethyl acetate (10:1). Colorless oil. **^{1}H NMR** (400 MHz, $CDCl_3$) δ 1.52 (q, J = 7.4 Hz, 2H), 1.27 – 1.23 (m, 8H), 1.21 (s, 12H), 1.20 (s, 12H), 0.88 – 0.80 (m, 3H), 0.69 (t, J = 7.9 Hz, 1H). **^{13}C NMR** (101 MHz, $CDCl_3$) δ 82.8, 32.5, 31.7, 29.3, 25.7, 24.8, 24.5, 22.6, 14.1. **^{11}B NMR** (128 MHz, $CDCl_3$) δ 34.59. Spectroscopic data are in agreement with those previously reported.[15]

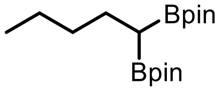

2,2'-(pentane-1,1-diyl)bis(4,4,5,5-tetramethyl-1,3,2-dioxaborolane) **36**

Eluent: petroleum ether/ethyl acetate (10:1). Colorless oil. **^{1}H NMR** (400 MHz, $CDCl_3$) δ 1.58 – 1.51 (m, 2H), 1.27 – 1.25 (m, 4H), 1.22 (s, 12H), 1.22 (s, 12H), 0.86 (t, J = 6.9 Hz, 3H), 0.70 (t, J = 7.9 Hz, 1H). **^{13}C NMR** (101 MHz, $CDCl_3$) δ 82.9, 34.9, 29.7, 25.4, 24.9, 24.5, 22.7, 14.1. **^{11}B NMR** (128 MHz, $CDCl_3$) δ 34.03. **HRMS** calcd for $C_{17}H_{35}B_2O_4$: 325.2716 $[M+H]^+$, found: 325.2724.

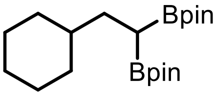

2,2'-(2-cyclohexylethane-1,1-diyl)bis(4,4,5,5-tetramethyl-1,3,2-dioxaborolane) **37**

Eluent: petroleum ether/ethyl acetate (15:1). White solid. **^{1}H NMR** (400 MHz, $CDCl_3$) δ 1.74 – 1.62 (m, 4H), 1.48 – 1.41 (m, 2H), 1.22 (s, 12H), 1.21 (s, 13H), 1.13 (dt, J = 9.6, 3.4 Hz, 4H), 0.82 – 0.75 (m, 3H). **^{13}C NMR** (101 MHz, $CDCl_3$) δ 82.9, 39.8, 33.1, 33.0, 26.8, 26.5, 24.8, 24.6. **^{11}B NMR** (128 MHz, $CDCl_3$) δ 34.13. Spectroscopic data are in agreement with those previously reported.[11]

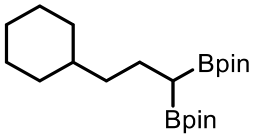

2,2'-(3-cyclohexylpropane-1,1-diyl)bis(4,4,5,5-tetramethyl-1,3,2-dioxaborolane) **38**

Eluent: petroleum ether/ethyl acetate (10:1). Colorless oil. **^{1}H NMR** (400 MHz, $CDCl_3$) δ 1.67 (t,

J = 15.0 Hz, 6H), 1.58 – 1.50 (m, 2H), 1.23 (s, 12H), 1.22 (s, 12H), 1.19 – 1.11 (m, 5H), 0.84 (q, J = 10.2, 9.6 Hz, 2H), 0.66 (t, J = 7.9 Hz, 1H). **^{13}C NMR** (101 MHz, $CDCl_3$) δ 82.8, 40.5, 37.7, 33.3, 26.7, 26.3, 24.8, 24.8, 24.4, 23.0. **^{11}B NMR** (128 MHz, $CDCl_3$) δ 33.57. Spectroscopic data are in agreement with those previously reported.[16]

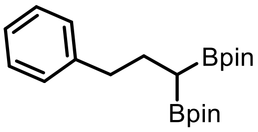

2,2'-(3-phenylpropane-1,1-diyl)bis(4,4,5,5-tetramethyl-1,3,2-dioxaborolane) **41**

Eluent: petroleum ether/ethyl acetate (10:1). Colorless oil. **^{1}H NMR** (400 MHz, $CDCl_3$) δ 7.13 – 7.05 (m, 2H), 7.06 – 6.94 (m, 3H), 2.49 – 2.40 (m, 2H), 1.76 – 1.66 (m, 2H), 1.09 (s, 12H), 1.08 (s, 12H), 0.66 (t, J = 7.9 Hz, 1H). **^{13}C NMR** (101 MHz, $CDCl_3$) δ 143.0, 128.6, 128.1, 125.5, 83.0, 38.7, 29.7, 28.0, 24.9, 24.5. **^{11}B NMR** (128 MHz, $CDCl_3$) δ 33.95. Spectroscopic data are in agreement with those previously reported.[17]

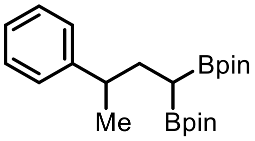

2,2'-(3-phenylbutane-1,1-diyl)bis(4,4,5,5-tetramethyl-1,3,2-dioxaborolane) **43**

Eluent: petroleum ether/ethyl acetate (20:1). Colorless oil. **^{1}H NMR** (400 MHz, $CDCl_3$) δ 7.27 – 7.22 (m, 2H), 7.14 (dd, J = 16.6, 7.5 Hz, 3H), 2.68 – 2.60 (m, 1H), 1.81 (t, J = 7.5 Hz, 2H), 1.23 (s, 12H), 1.22 (s, 3H), 1.19 (s, 12H), 0.71 (t, J = 7.8 Hz, 1H). **^{13}C NMR** (101 MHz, $CDCl_3$) δ 147.6, 128.1, 127.2, 125.6, 82.87, 82.85, 41.8, 34.4, 24.82, 24.80, 24.57, 24.51, 21.6. **^{11}B NMR** (128 MHz, $CDCl_3$) δ 34.27. **HRMS** calcd for $C_{22}H_{37}B_2O_4$: 387.2872 $[M+H]^+$, found: 387.2882.

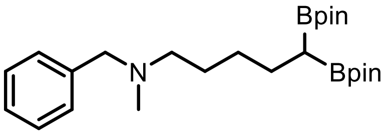

N-benzyl-*N*-methyl-5,5-bis(4,4,5,5-tetramethyl-1,3,2-dioxaborolan-2-yl)pentan-1-amine **45**

Eluent: dichloromethane/methanol (40:1). Colorless oil. **^{1}H NMR** (400 MHz, $CDCl_3$) δ 7.34 – 7.27 (m, 5H), 3.52 (s, 2H), 2.41 – 7.27 (m, 2H), 2.21 (s, 3H), 1.57 – 1.52 (m, 2H), 1.30 – 1.27 (m, 4H), 1.23 (s, 25H). **^{13}C NMR** (101 MHz, $CDCl_3$) δ 129.3, 128.3, 128.2, 127.1, 82.9, 75.1,

62.0, 57.4, 42.0, 29.6, 24.9, 24.5, 22.6, 14.1. **^{11}B NMR** (128 MHz, $CDCl_3$) δ 33.32. **HRMS** calcd for $C_{25}H_{43}B_2NNaO_4^+$ [M+Na$^+$]: 466.3270, Found: 466.3275.

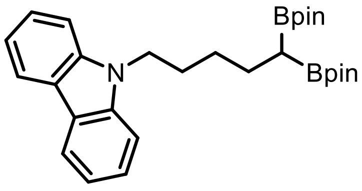

9-(5,5-bis(4,4,5,5-tetramethyl-1,3,2-dioxaborolan-2-yl)pentyl)-9*H*-carbazole **46**

Eluent: petroleum ether/ethyl acetate (10:1). Colorless oil. **^{1}H NMR** (400 MHz, $CDCl_3$) δ 8.06 (d, *J* = 8.5 Hz, 2H), 7.47 – 7.35 (m, 4H), 7.23 – 7.15 (m, 2H), 4.27 (t, *J* = 7.2 Hz, 2H), 1.93 – 1.80 (m, 2H), 1.59 (q, *J* = 7.9 Hz, 2H), 1.43 – 1.31 (m, 2H), 1.16 (s, 12H), 1.13 (s, 12H), 0.70 (t, *J* = 7.9 Hz, 1H). **^{13}C NMR** (101 MHz, $CDCl_3$) δ 140.4, 125.5, 122.8, 120.2, 118.6, 108.7, 82.9, 43.0, 30.0, 29.0, 25.4, 24.7, 24.4. **^{11}B NMR** (128 MHz, $CDCl_3$) δ 33.34. **HRMS** calcd for $C_{29}H_{42}B_2NO_4^+$ [M+H$^+$]: 490.3294, Found: 490.3305.

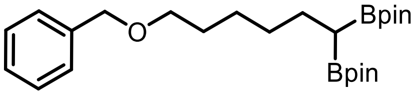

2,2'-(6-(benzyloxy)hexane-1,1-diyl)bis(4,4,5,5-tetramethyl-1,3,2-dioxaborolane) **47**

Eluent: petroleum ether/ethyl acetate (10:1). Colorless oil. **^{1}H NMR** (400 MHz, $CDCl_3$) δ 7.40 – 7.29 (m, 4H), 7.30 – 7.21 (m, 1H), 4.49 (d, *J* = 4.1 Hz, 2H), 3.51 – 3.39 (m, 2H), 1.71 – 1.58 (m, 4H), 1.49 – 1.39 (m, 2H), 1.36 (d, *J* = 7.7 Hz, 2H), 1.20 (s, 25H) the "CH" signals were overlapped with the intensive signal of Me groups. **^{13}C NMR** (101 MHz, $CDCl_3$) δ 138.7, 128.2, 127.5, 127.3, 82.8, 72.8, 70.5, 32.3, 29.6, 26.1, 25.6, 24.8, 24.5, 24.5. **^{11}B NMR** (128 MHz, $CDCl_3$) δ 33.62. **HRMS** calcd for $C_{25}H_{42}B_2NaO_5^+$ [M+Na$^+$]: 467.3111, Found: 467.3120.

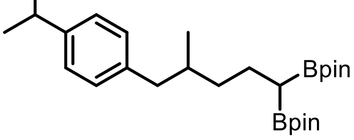

2,2'-(5-(4-isopropylphenyl)-4-methylpentane-1,1-diyl)bis(4,4,5,5-tetramethyl-1,3,2-dioxaborolane) **48**

Eluent: petroleum ether/ethyl acetate (10:1). Colourless oil. **^{1}H NMR** (400 MHz, $CDCl_3$) δ 7.23 – 6.93 (m, 4H), 2.86 (dt, *J* = 13.8, 6.9 Hz, 1H), 2.58 (dd, *J* = 13.4, 6.7 Hz, 1H), 2.37 (dd, *J* = 13.4, 7.5 Hz, 1H), 1.85 – 1.73 (m, 2H), 1.66 (dt, *J* = 12.3, 6.1 Hz, 2H), 1.53 – 1.44 (m, 2H), 1.23 (d, *J* = 6.9 Hz, 6H), 1.19 (d, *J* = 2.0 Hz, 24H), 0.89 (d, *J* = 6.6 Hz, 3H). **^{13}C NMR** (101 MHz,

CDCl3) δ 145.5, 139.4, 129.1, 125.8, 82.5, 43.6, 37.6, 35.9, 33.6, 25.9, 24.6, 24.5, 24.0, 19.7. **^{11}B NMR** (128 MHz, $CDCl_3$) δ 33.33. **HRMS** calcd for $C_{27}H_{46}B_2NaO_4^+$ [M+Na$^+$]: 479.3474, Found: 479.3481.

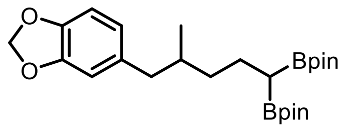

2,2'-(5-(benzo[*d*][1,3]dioxol-5-yl)-4-methylpentane-1,1-diyl)bis(4,4,5,5-tetramethyl-1,3,2-dioxaborolane) **49**

Eluent: petroleum ether/ethyl acetate (10:1). Colourless oil. **^{1}H NMR** (400 MHz, $CDCl_3$) δ 6.69 (d, *J* = 7.9 Hz, 1H), 6.63 (s, 1H), 6.57 (d, *J* = 7.9 Hz, 1H), 5.89 (s, 2H), 2.50 (dd, *J* = 13.5, 6.5 Hz, 1H), 2.28 (dd, *J* = 13.5, 7.8 Hz, 1H), 1.63 (s, 2H), 1.22 – 1.18 (m, 27H), 0.83 (d, *J* = 6.6 Hz, 3H), 0.66 (t, *J* = 7.9 Hz, 1H). **^{13}C NMR** (101 MHz, $CDCl_3$) δ 147.2, 145.3, 135.6, 121.9, 109.6, 107.8, 100.6, 82.9, 43.4, 39.5, 35.2, 24.9, 24.8, 24.5, 23.3, 19.5. **^{11}B NMR** (128 MHz, $CDCl_3$) δ 33.61. **HRMS** calcd for $C_{25}H_{40}B_2NaO_6^+$ [M+Na$^+$]: 481.2903, Found: 481.2894.

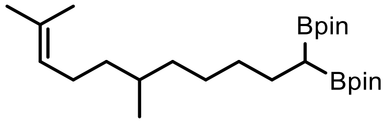

2,2'-(6,10-dimethylundec-9-ene-1,1-diyl)bis(4,4,5,5-tetramethyl-1,3,2-dioxaborolane) **50**

Eluent: petroleum ether/ethyl acetate (10:1). Colourless oil. **^{1}H NMR** (400 MHz, $CDCl_3$) δ 5.09 (s, 1H), 2.04 – 1.83 (m, 3H), 1.67 (s, 3H), 1.60 (s, 3H), 1.54 (q, *J* = 6.9, 5.5 Hz, 4H), 1.22 (d, *J* = 3.4 Hz, 24H), 1.15 – 1.02 (m, 6H), 0.83 (d, *J* = 6.4 Hz, 3H), 0.70 (d, *J* = 7.9 Hz, 1H). **^{13}C NMR** (101 MHz, $CDCl_3$) δ 130.8, 125.2, 82.9, 37.2, 36.9, 33.0, 32.4, 27.1, 25.6, 24.9, 24.5, 19.6, 17.6, 17.5, 14.1. **^{11}B NMR** (128 MHz, $CDCl_3$) δ 34.20. **HRMS** calcd for $C_{25}H_{49}B_2O_4^+$ [M+H$^+$]: 435.3811, Found: 435.3805.

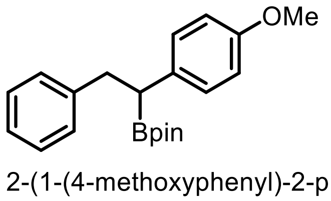

2-(1-(4-methoxyphenyl)-2-phenylethyl)-4,4,5,5-tetramethyl-1,3,2-dioxaborolane **51**

Eluent: petroleum ether/ethyl acetate (20:1). Colourless oil. **^{1}H NMR** (400 MHz, $CDCl_3$) δ 7.23 – 7.09 (m, 7H), 6.83 – 6.76 (m, 2H), 3.76 (s, 3H), 3.10 (dd, *J* = 13.5, 9.6 Hz, 1H), 2.91 (dd, *J* = 13.5, 7.0 Hz, 1H), 2.61 (dd, *J* = 9.6, 7.0 Hz, 1H), 1.11 (s, 6H), 1.10 (s, 6H). **^{13}C NMR** (101 MHz, $CDCl_3$) δ 157.5, 141.8, 134.6, 129.3, 128.9, 128.0, 125.7, 113.8, 83.3, 55.1, 39.1, 24.6, 24.5.

^{11}**B NMR** (128 MHz, $CDCl_3$) δ 32.95. Spectroscopic data are in agreement with those previously reported.

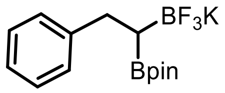

4,4,5,5-tetramethyl-2-(2-phenyl-1-(trifluoro-λ^4-boraneyl)ethyl)-1,3,2-dioxaborolane, potassium salt **52**

Isolated after recrystallization. White solid. ^{1}**H NMR** (400 MHz, DMSO-*d*$_6$) δ 7.12 (h, *J* = 5.8 Hz, 4H), 7.00 (td, *J* = 6.4, 2.8 Hz, 1H), 2.55 (d, *J* = 8.1 Hz, 2H), 1.01 (s, 6H), 0.98 (s, 6H), 0.12 (h, *J* = 6.9 Hz, 1H). ^{13}**C NMR** (101 MHz, DMSO-*d*$_6$) δ 148.7, 128.5, 127.9, 124.6, 81.2, 34.0, 25.2, 24.9. ^{11}**B NMR** (128 MHz, DMSO-*d*$_6$) δ 37.18, 4.21. ^{19}**F NMR** (376 MHz, DMSO) δ -135.45. Spectroscopic data are in agreement with those previously reported.[4]

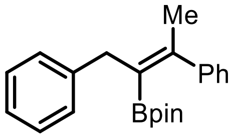

(*E*)-2-(1,3-diphenylbut-2-en-2-yl)-4,4,5,5-tetramethyl-1,3,2-dioxaborolane **53**

Eluent: petroleum ether/ethyl acetate (30:1). Colourless oil. ^{1}**H NMR** (400 MHz, $CDCl_3$) δ 7.34 – 7.16 (m, 9H), 7.17 – 7.10 (m, 1H), 3.66 (s, 2H), 2.13 (s, 3H), 0.88 (s, 12H). ^{13}**C NMR** (101 MHz, $CDCl_3$) δ 147.7, 146.1, 141.0, 128.9, 128.1, 127.9, 127.7, 126.7, 125.6, 82.9, 37.6, 24.4, 20.5. ^{11}**B NMR** (128 MHz, $CDCl_3$) δ 30.45. Spectroscopic data are in agreement with those previously reported.[5]

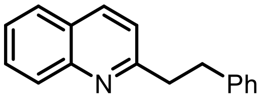

2-phenethylquinoline **54**

Eluent: petroleum ether/ethyl acetate (10:1). Yellow oil. ^{1}**H NMR** (400 MHz, $CDCl_3$) δ 8.08 (d, *J* = 8.5 Hz, 1H), 7.93 (d, *J* = 8.5 Hz, 1H), 7.69 (dd, *J* = 8.1, 1.5 Hz, 1H), 7.64 (ddd, *J* = 8.5, 6.9, 1.5 Hz, 1H), 7.42 (ddd, *J* = 8.1, 6.9, 1.2 Hz, 1H), 7.29 – 7.19 (m, 4H), 7.19 – 7.11 (m, 2H), 3.30 – 3.22 (m, 2H), 3.17 – 3.10 (m, 2H). ^{13}**C NMR** (101 MHz, $CDCl_3$) δ 161.6, 147.9, 141.4, 135.9, 129.2, 128.7, 128.3, 128.2, 127.3, 126.6, 125.8, 125.6, 121.3, 40.7, 35.7. Spectroscopic data are in agreement with those previously reported.[18]

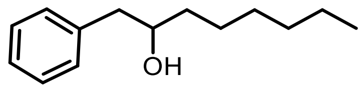

1-phenyloctan-2-ol **55**

Eluent: petroleum ether/ethyl acetate (15:1). Colourless oil. **^{1}H NMR** (400 MHz, $CDCl_3$) δ 7.30 (dd, *J* = 8.4, 6.2 Hz, 2H), 7.25 – 7.18 (m, 3H), 3.80 (dq, *J* = 8.1, 3.7 Hz, 1H), 2.82 (dd, *J* = 13.6, 4.3 Hz, 1H), 2.64 (dd, *J* = 13.5, 8.3 Hz, 1H), 1.51 (dt, *J* = 13.1, 4.6 Hz, 4H), 1.37 – 1.23 (m, 7H), 0.94 – 0.83 (m, 3H). **^{13}C NMR** (101 MHz, $CDCl_3$) δ 138.7, 129.4, 128.5, 126.4, 72.7, 44.1, 36.9, 31.8, 29.3, 25.7, 22.6, 14.0. Spectroscopic data are in agreement with those previously reported.[19]

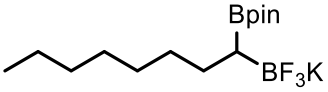

Potassium trifluoro(1-(4,4,5,5-tetramethyl-1,3,2-dioxaborolan-2-yl)octyl)borate **56**

Isolated after recrystallization. White solid. **^{1}H NMR** (400 MHz, DMSO-*d*$_6$) δ 1.58 – 1.36 (m, 12H), 1.34 (s, 6H), 1.32 (s, 6H), 1.09 (t, *J* = 6.9 Hz, 3H), -0.02 (m, 1H). **^{13}C NMR** (101 MHz, DMSO-*d*$_6$) δ 81.00, 33.5, 31.8, 30.1, 29.3, 27.8, 25.3, 24.8, 22.6, 14.4. **^{11}B NMR** (128 MHz, DMSO-*d*$_6$) δ 36.46, 4.77. **^{19}F NMR** (376 MHz, DMSO) δ -134.71. **HRMS** calcd for $C_{14}H_{28}B_2F_3O_2^-$: 307.2233 $[M]^-$, found: 307.2232.

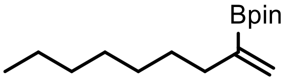

4,4,5,5-tetramethyl-2-(non-1-en-2-yl)-1,3,2-dioxaborolane **57**

Eluent: petroleum ether/ethyl acetate (50:1). Colourless oil. **^{1}H NMR** (400 MHz, $CDCl_3$) δ 5.74 (d, *J* = 3.5 Hz, 1H), 5.58 (brs, 1H), 2.13 (tt, *J* = 7.5, 1.2 Hz, 2H), 1.47 – 1.33 (m, 4H), 1.26 (s, 20H), 0.89 – 0.84 (m, 3H). **^{13}C NMR** (101 MHz, $CDCl_3$) δ 128.6, 83.2, 35.3, 31.8, 29.2, 24.7, 22.7, 14.1. **^{11}B NMR** (128 MHz, $CDCl_3$) δ 29.90. Spectroscopic data are in agreement with those previously reported.[20]

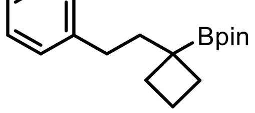

4,4,5,5-tetramethyl-2-(1-phenethylcyclobutyl)-1,3,2-dioxaborolane **58**

Eluent: petroleum ether/ethyl acetate (80:1). Colourless oil. **^{1}H NMR** (400 MHz, Chloroform-*d*) δ 7.27 – 7.23 (m, 2H), 7.20 – 7.13 (m, 3H), 2.54 – 2.44 (m, 2H), 2.15 (ddt, *J* = 12.3, 4.6, 3.0 Hz,

2H), 2.00 – 1.88 (m, 2H), 1.88 – 1.81 (m, 2H), 1.73 (dt, J = 10.9, 8.9 Hz, 2H), 1.28 (s, 12H). **^{13}C NMR** (101 MHz, $CDCl_3$) δ 143.2, 128.3, 128.2, 125.5, 83.0, 42.0, 33.3, 30.2, 24.7, 18.2. **^{11}B NMR** (128 MHz, $CDCl_3$) δ 34.56. Spectroscopic data are in agreement with those previously reported.[9]

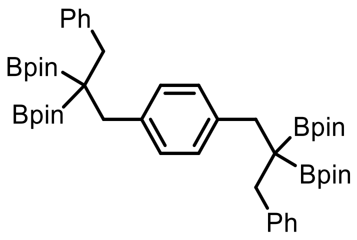

1,4-bis(3-phenyl-2,2-bis(4,4,5,5-tetramethyl-1,3,2-dioxaborolan-2-yl)propyl)benzene **59**

Eluent: petroleum ether/ethyl acetate (10:1). White solid. **^{1}H NMR** (400 MHz, $CDCl_3$) δ 7.19 (d, J = 6.7 Hz, 8H), 7.12 (d, J = 6.4 Hz, 2H), 7.08 (s, 4H), 2.98 (d, J = 14.5 Hz, 8H), 1.20 (d, J = 2.1 Hz, 48H). **^{13}C NMR** (101 MHz, $CDCl_3$) δ 141.8, 138.5, 129.8, 129.1, 127.6, 125.3, 83.4, 34.5, 34.2, 25.0. **^{11}B NMR** (128 MHz, $CDCl_3$) δ 38.61. **HRMS** calcd for $C_{48}H_{71}B_4O_8$: 819.5516 $[M+H]^+$, found: 819.5538.

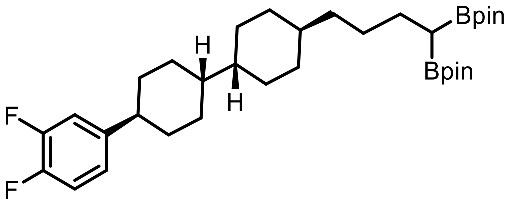

2,2'-(4-((1*s*,1'*r*,4*R*,4'*R*)-4'-(3,4-difluorophenyl)-[1,1'-bi(cyclohexan)]-4-yl)butane-1,1-diyl)bis(4,4,5,5-tetramethyl-1,3,2-dioxaborolane) **60**

Eluent: petroleum ether/ethyl acetate (10:1). White solid. **^{1}H NMR** (400 MHz, $CDCl_3$) δ 7.07 – 6.94 (m, 2H), 6.88 (ddt, J = 8.2, 3.8, 1.7 Hz, 1H), 2.40 (t, J = 12.2 Hz, 1H), 1.79 (dt, J = 54.2, 14.1 Hz, 10H), 1.55 – 1.48 (m, 2H), 1.23 (d, J = 3.8 Hz, 24H), 1.20 – 1.07 (m, 7H), 0.98 (dd, J = 22.2, 9.7 Hz, 3H), 0.92 – 0.81 (m, 3H), 0.71 (t, J = 7.8 Hz, 1H). **^{13}C NMR** (101 MHz, $CDCl_3$) δ 151.4 (d, J = 12.6 Hz), 149.7 (d, J = 12.6 Hz), 148.9 (d, J = 12.5 Hz), 147.3 (d, J = 12.7 Hz), 145.4 – 144.5 (m), 82.6, 43.9, 43.4, 42.9, 38.3, 37.3, 34.6, 33.6, 30.2 (d, J = 3.9 Hz), 28.3, 27.7, 24.6. **^{11}B NMR** (128 MHz, $CDCl_3$) δ 34.47. **^{19}F NMR** (376 MHz, $CDCl_3$) δ -138.72, -138.74, -138.75, -138.77, -138.79, -138.80, -138.83, -142.65, -142.66, -142.67, -142.68, -142.69, -142.70, -142.71, -142.72, -142.73, -142.74. **HRMS** calcd for $C_{34}H_{58}B_2F_2NO_4$: 604.4515 $[M+NH_4]^+$, found: 604.4527.

NMR Spectra

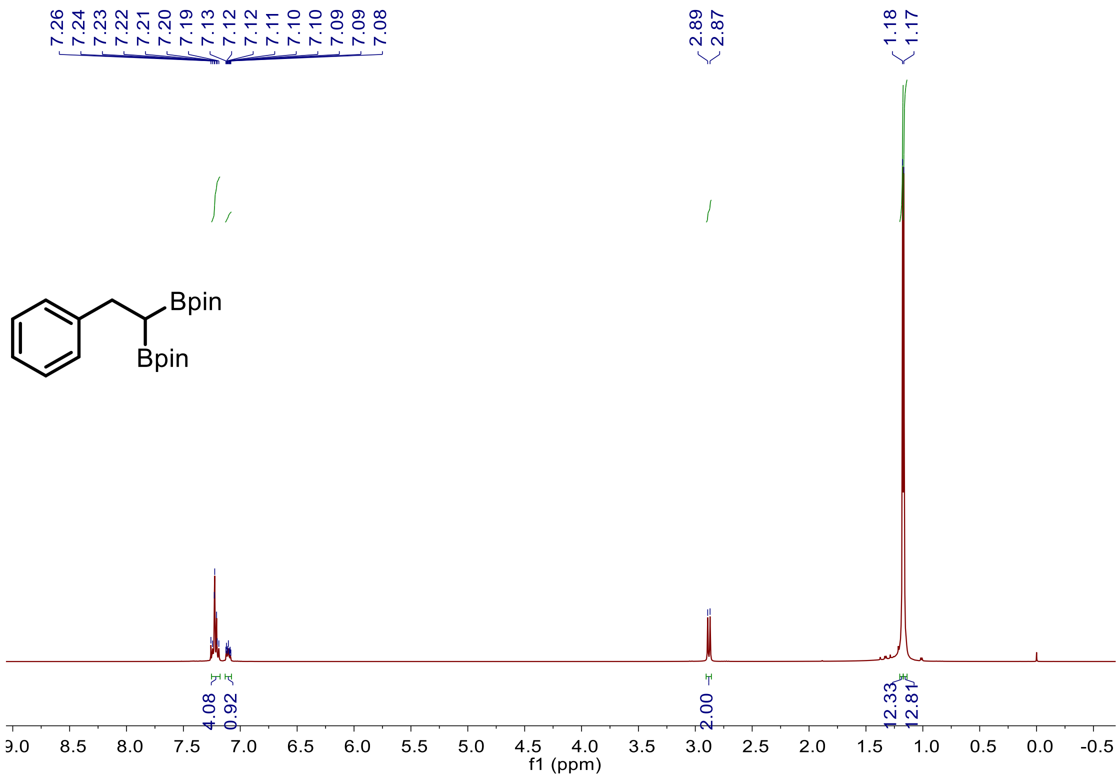

^{1}H NMR spectrum of **3** (Chloroform-*d*)

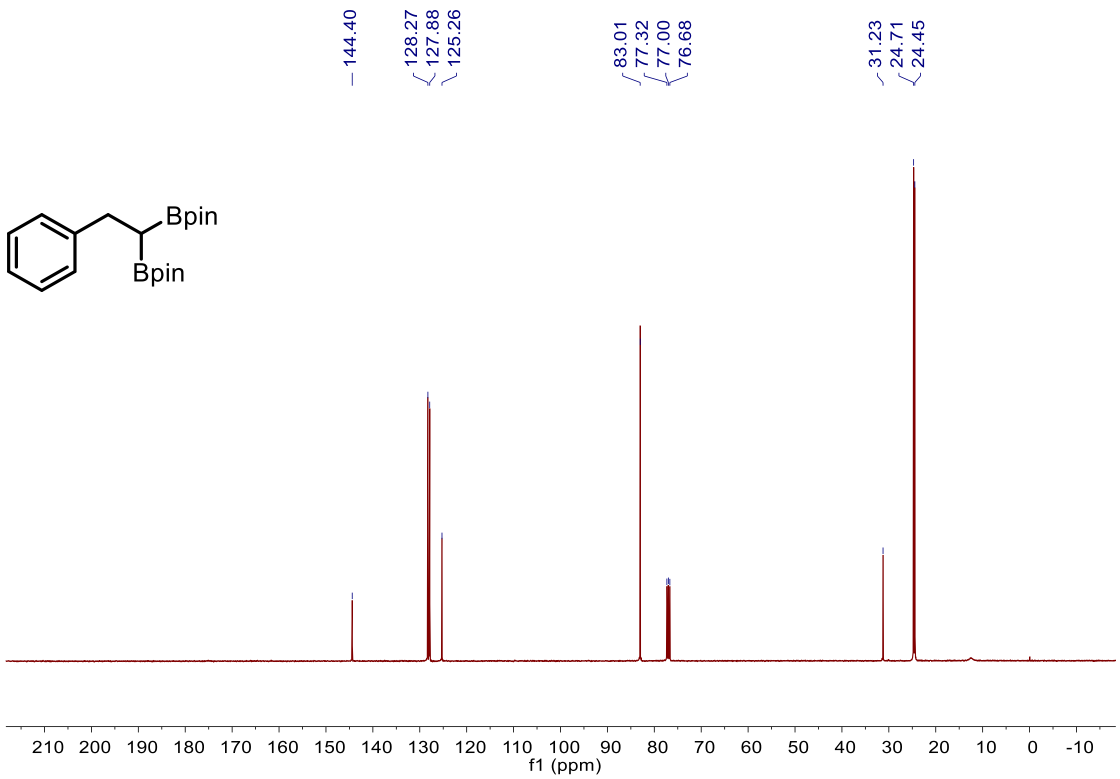

^{13}C NMR spectrum of **3** (Chloroform-*d*)

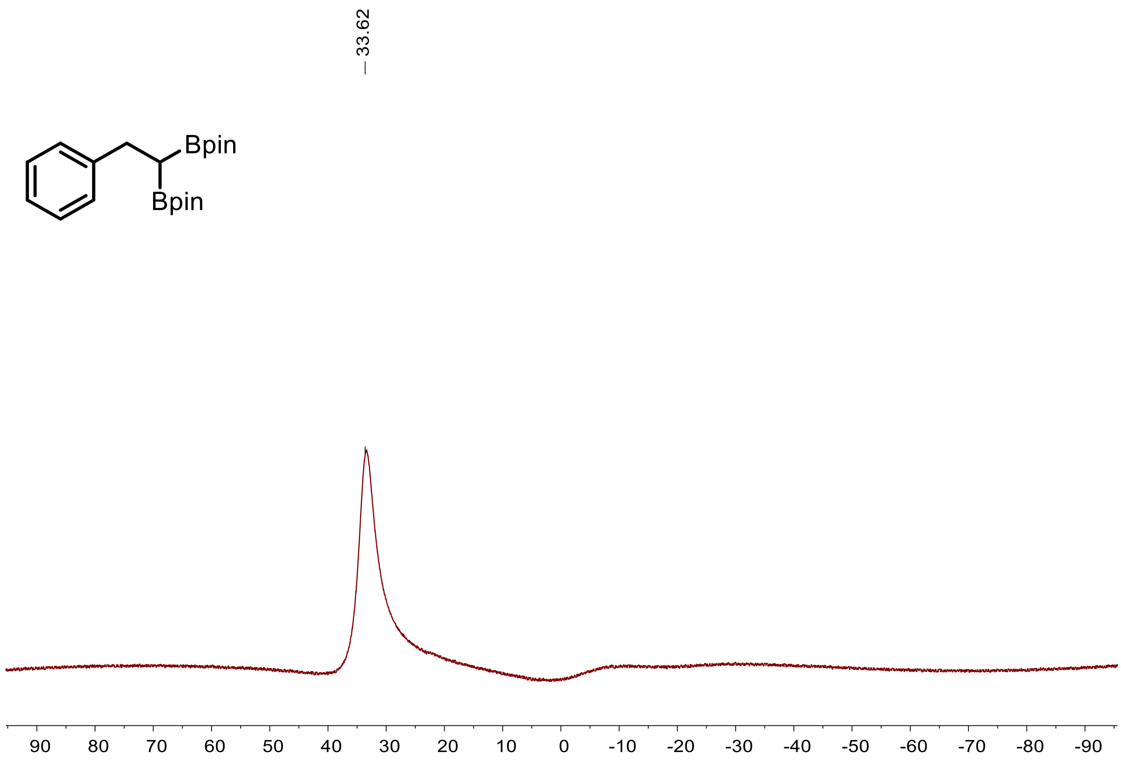

^{11}B NMR spectrum of **3** (Chloroform-*d*)

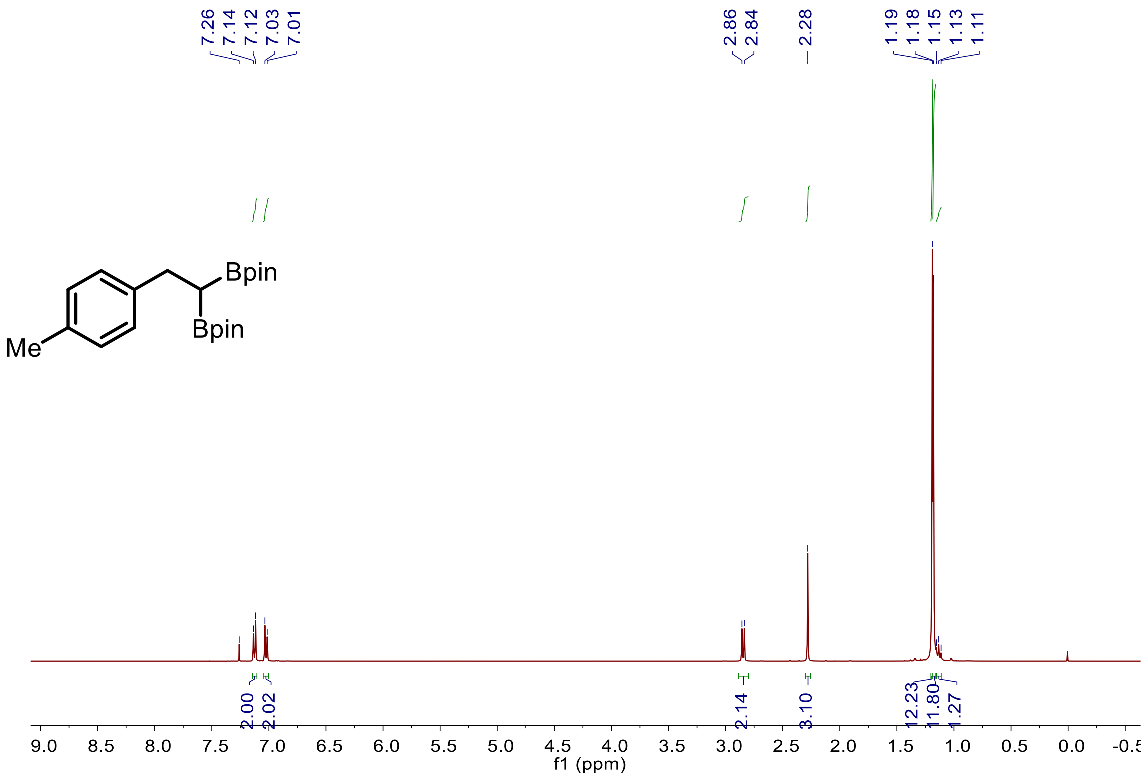

^{1}H NMR spectrum of **7** (Chloroform-*d*)

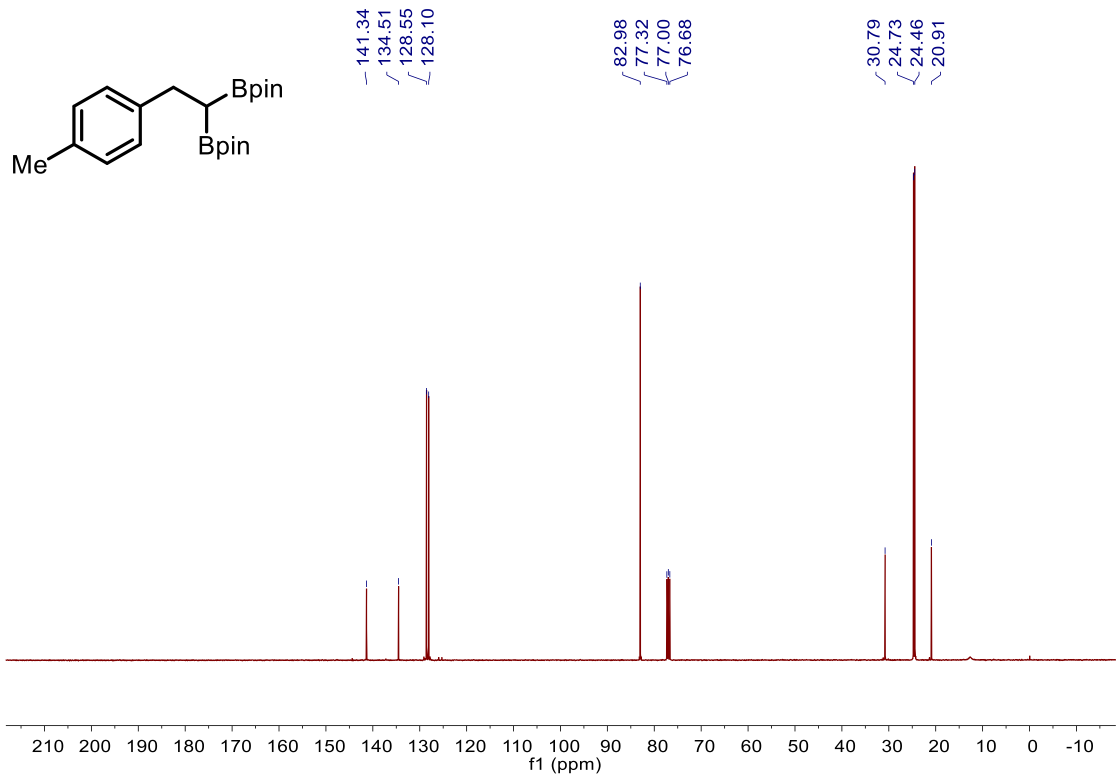

^{13}C NMR spectrum of **7** (Chloroform-*d*)

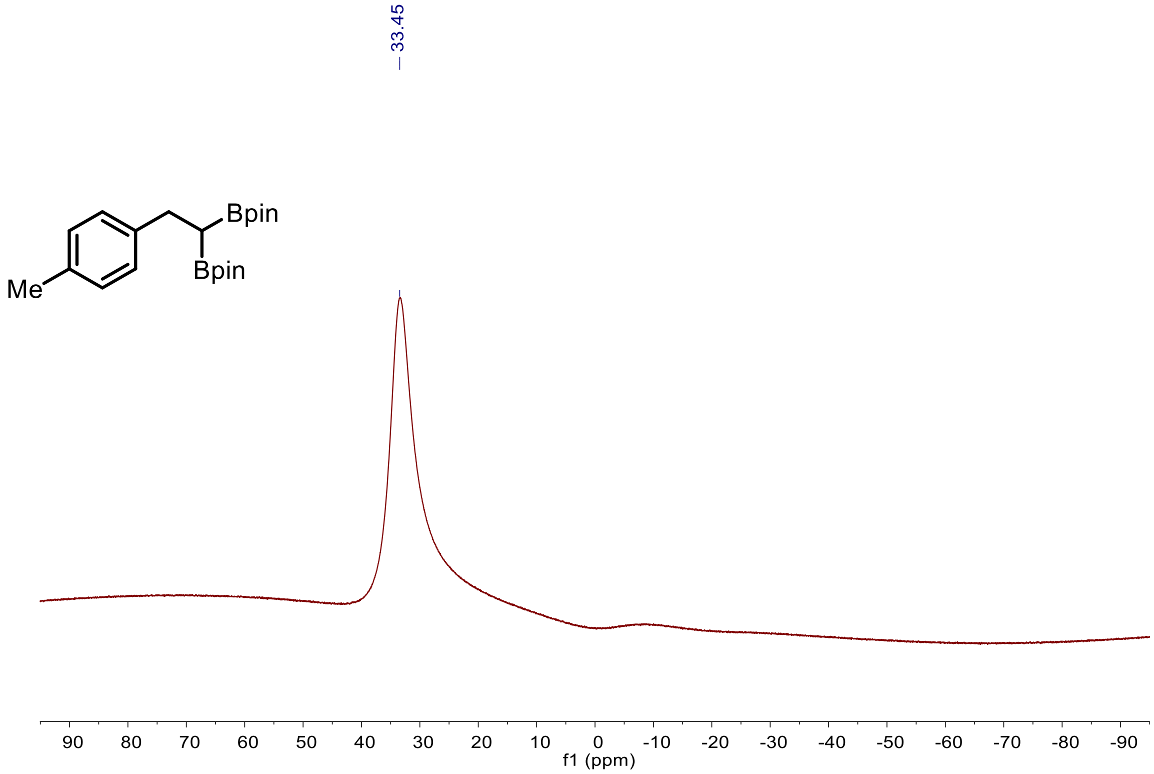

^{11}B NMR spectrum of **7** (Chloroform-*d*)

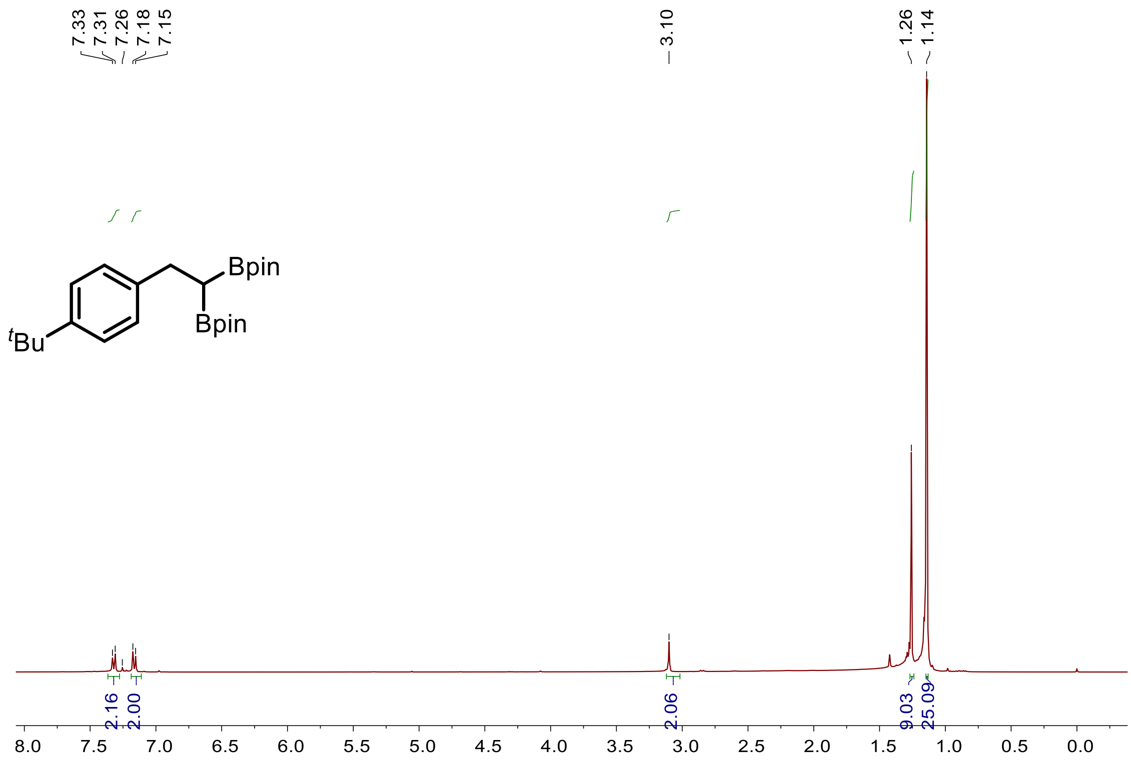

^{1}H NMR spectrum of **8** (Chloroform-*d*)

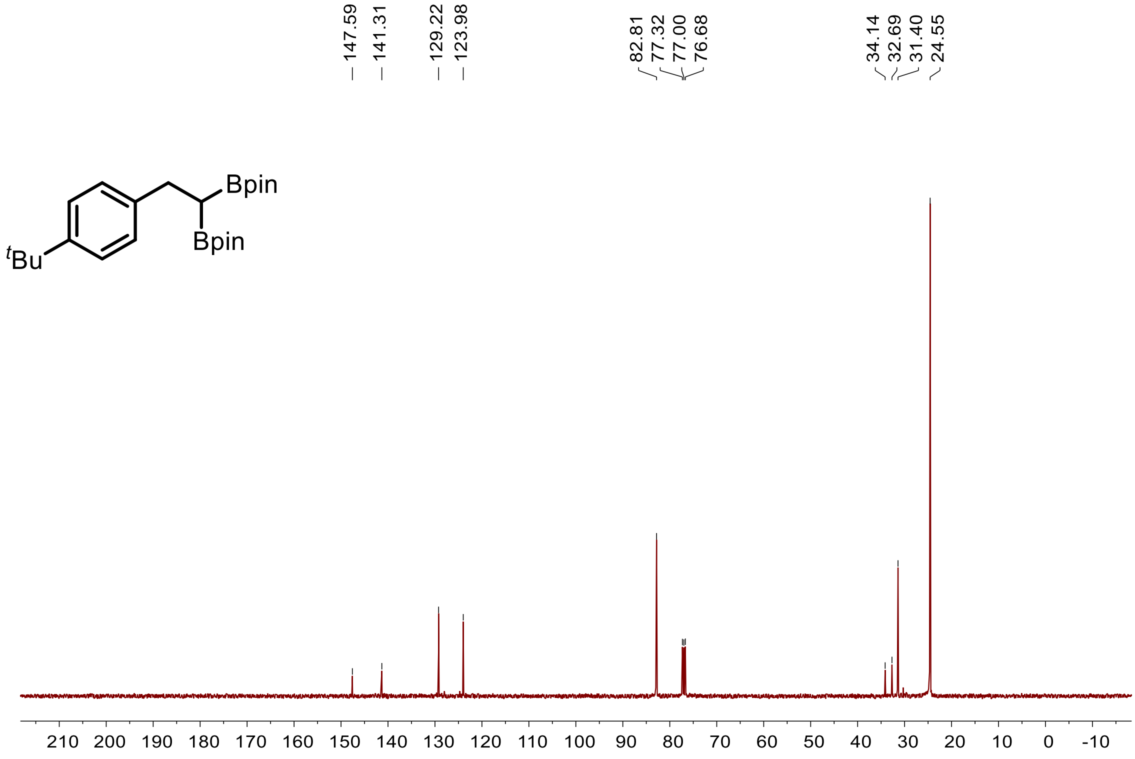

^{13}C NMR spectrum of **8** (Chloroform-*d*)

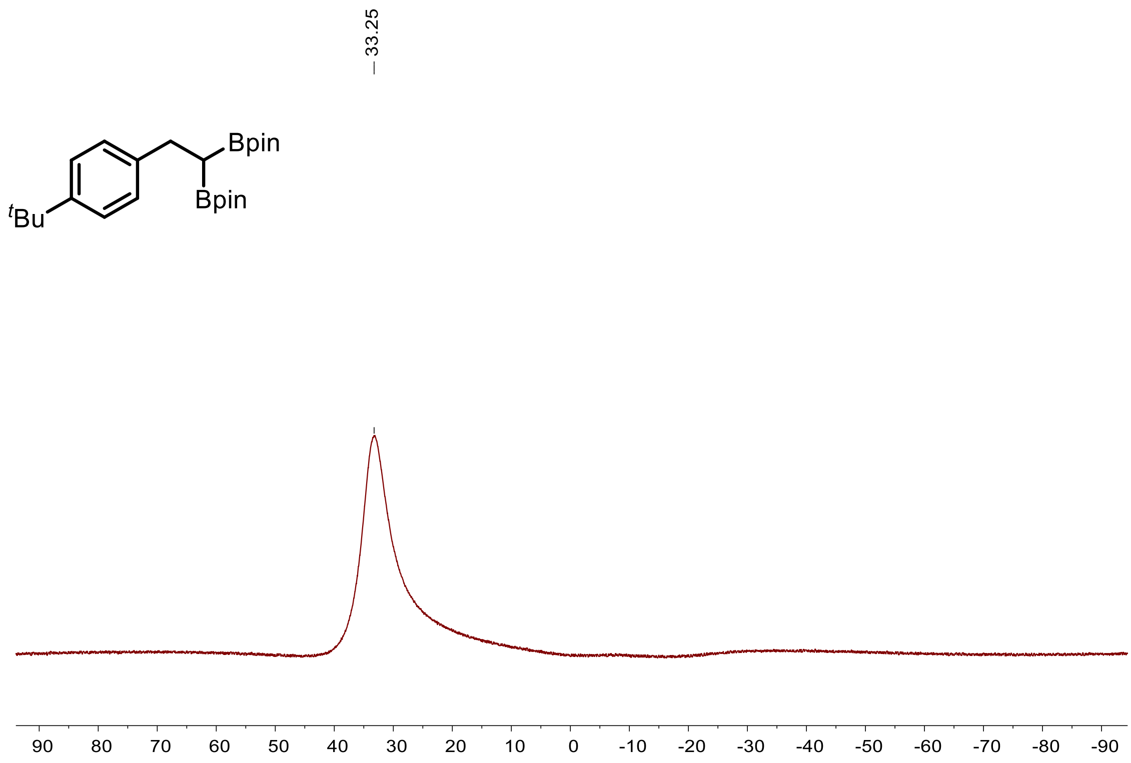

^{11}B NMR spectrum of **8** (Chloroform-*d*)

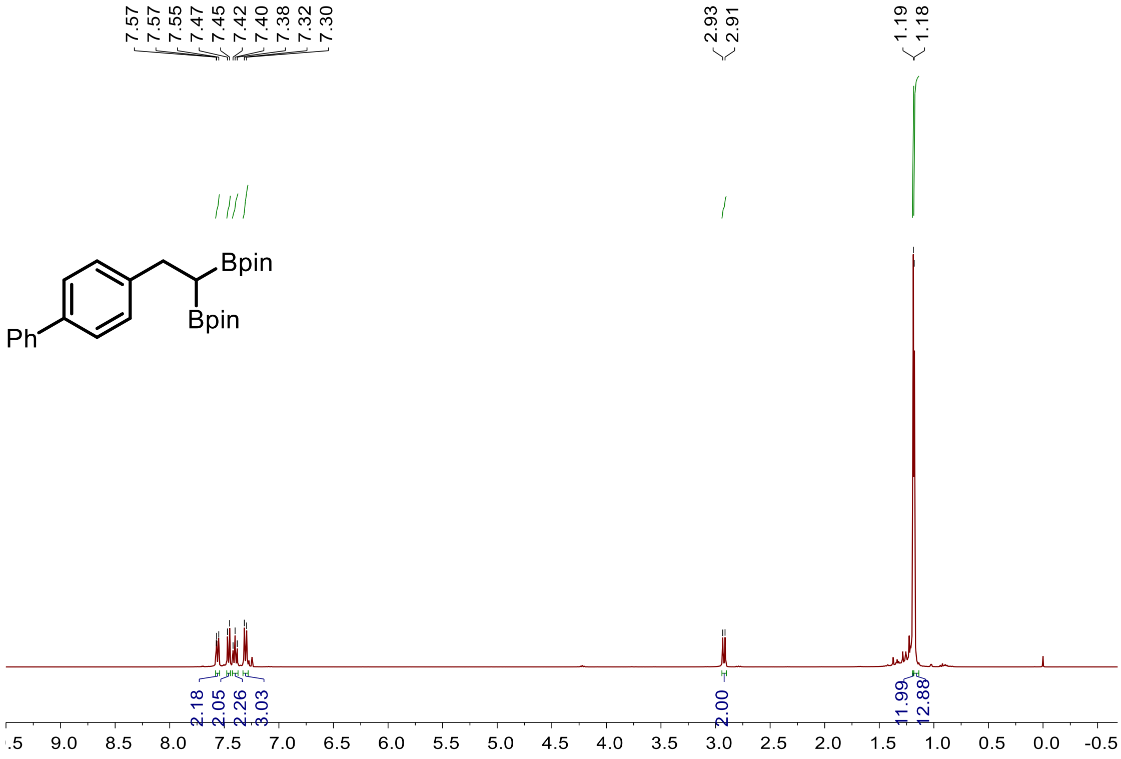

^{1}H NMR spectrum of **9** (Chloroform-*d*)

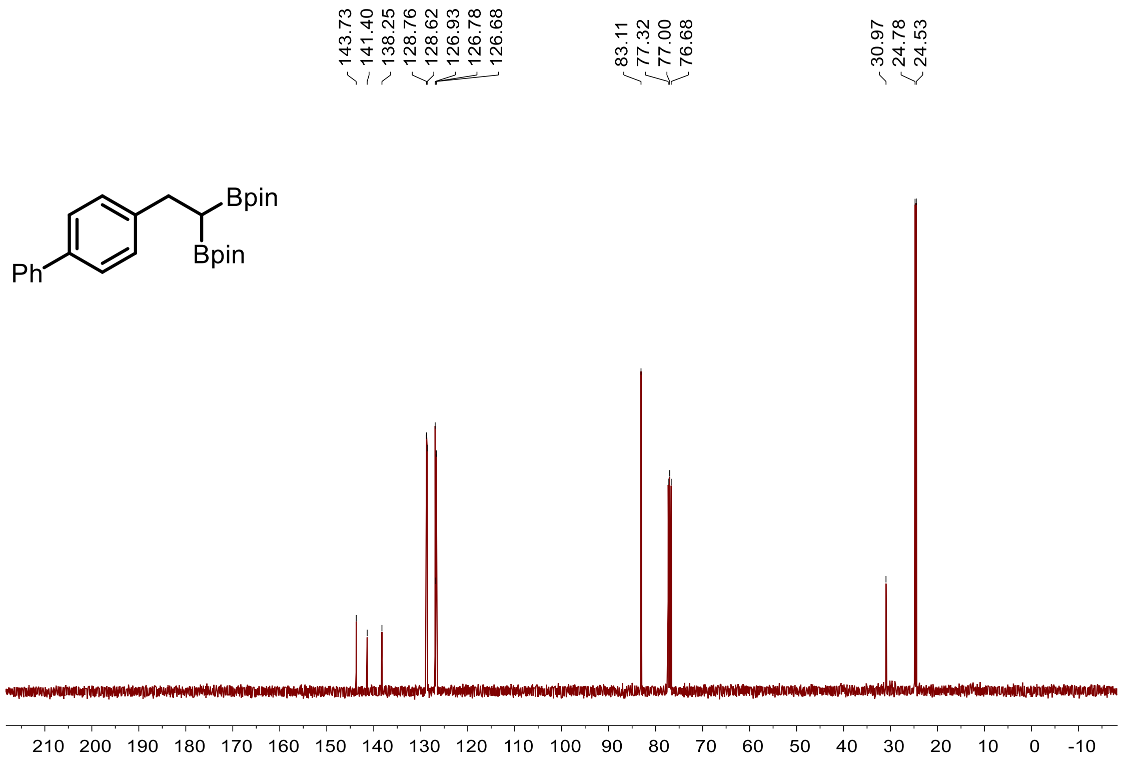

^{13}C NMR spectrum of **9** (Chloroform-*d*)

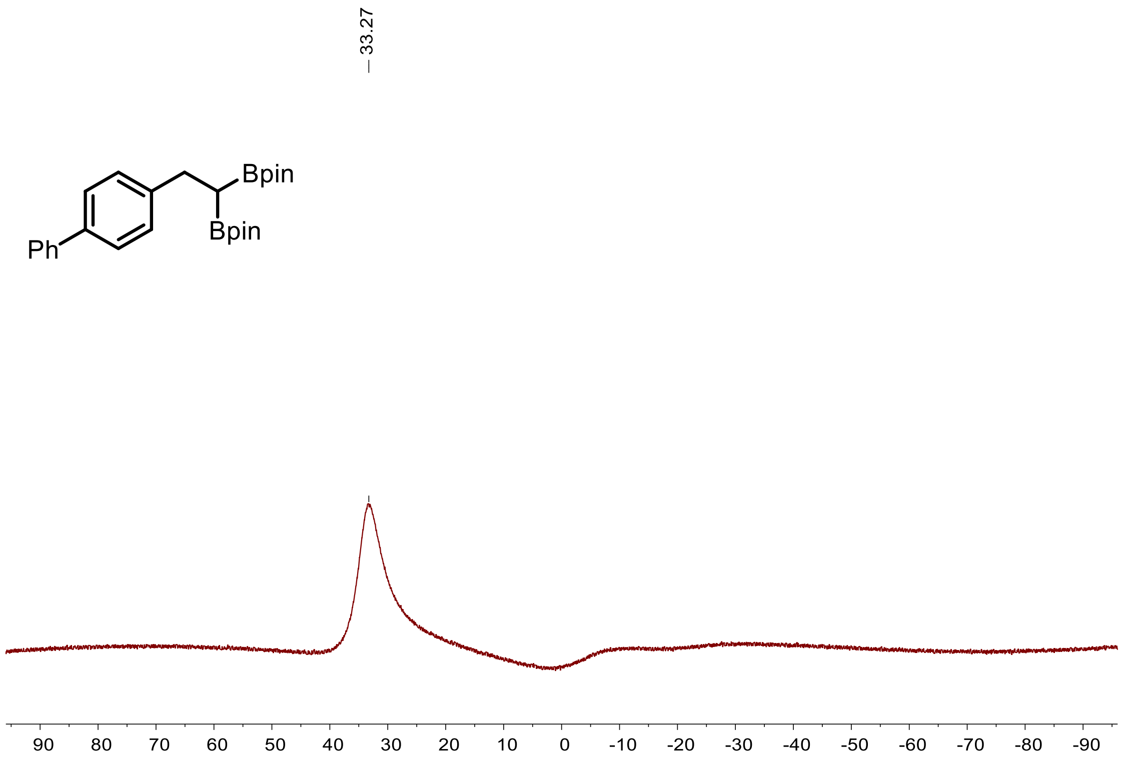

^{11}B NMR spectrum of **9** (Chloroform-*d*)

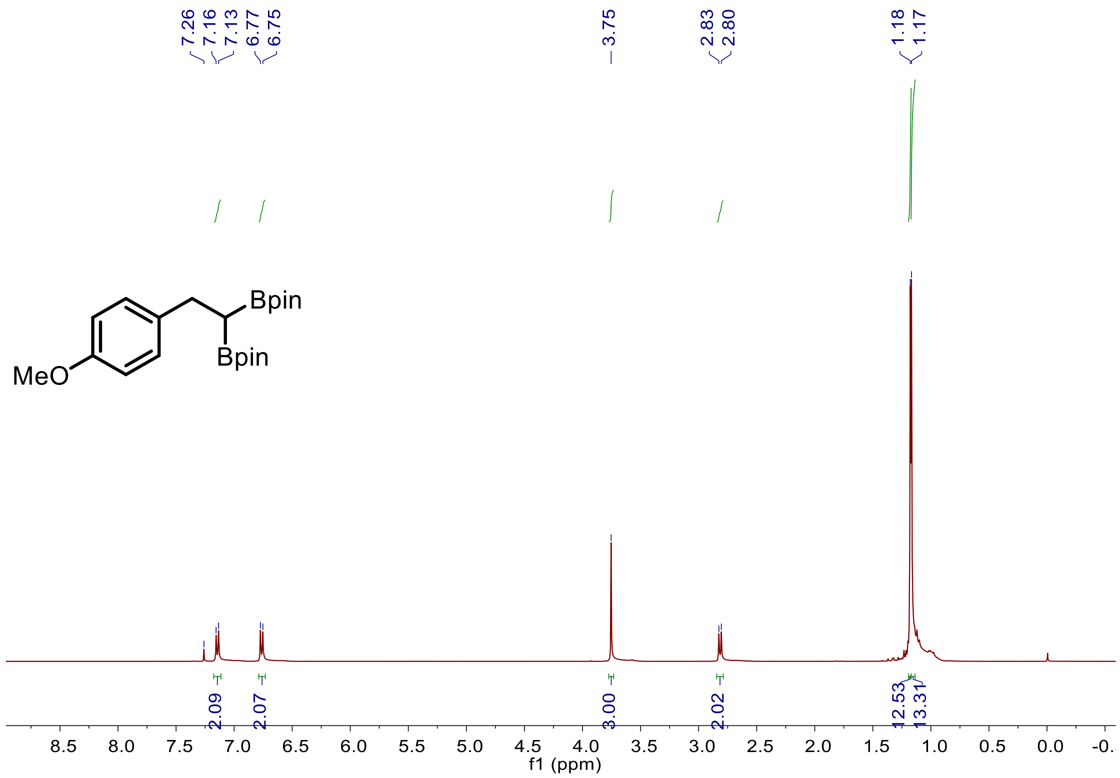

^{1}H NMR spectrum of **10** (Chloroform-*d*)

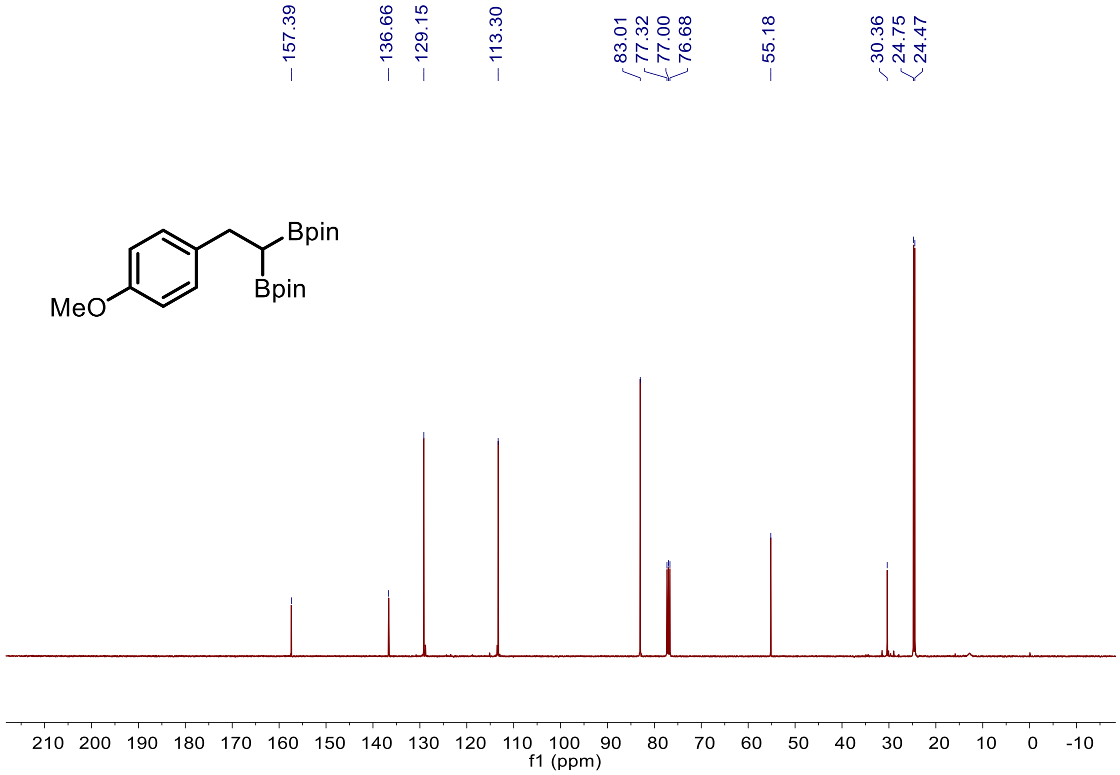

^{13}C NMR spectrum of **10** (Chloroform-*d*)

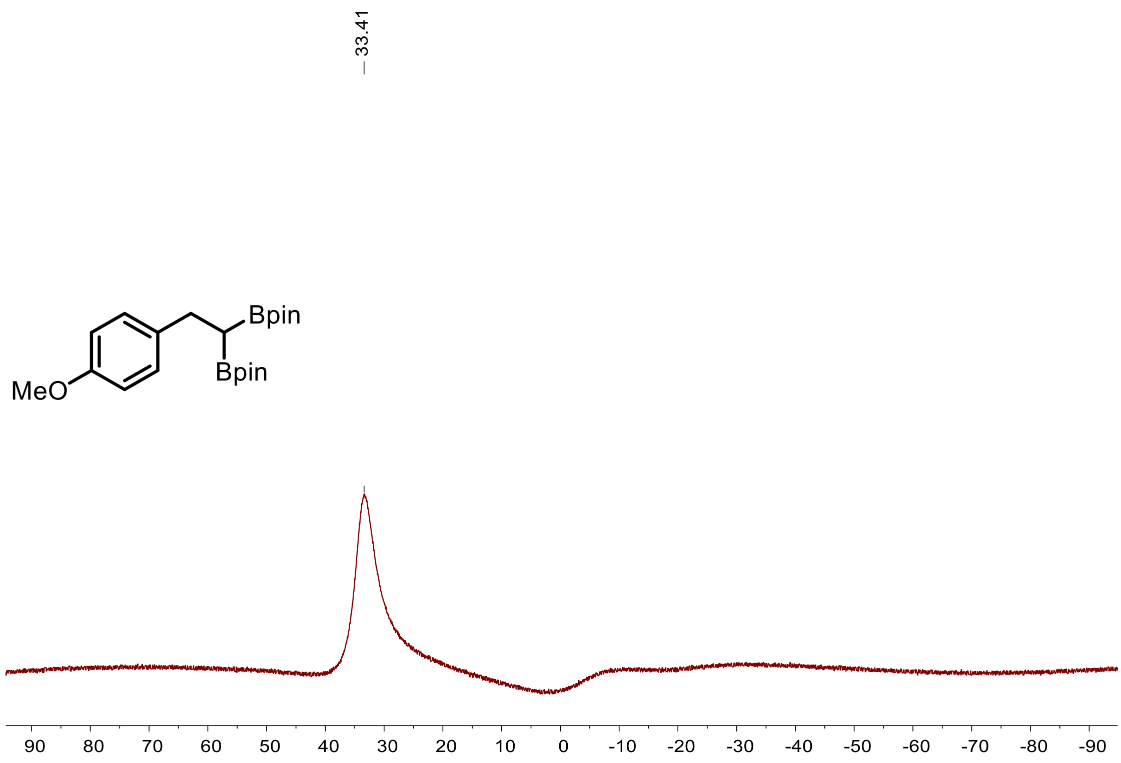

^{11}B NMR spectrum of **10** (Chloroform-*d*)

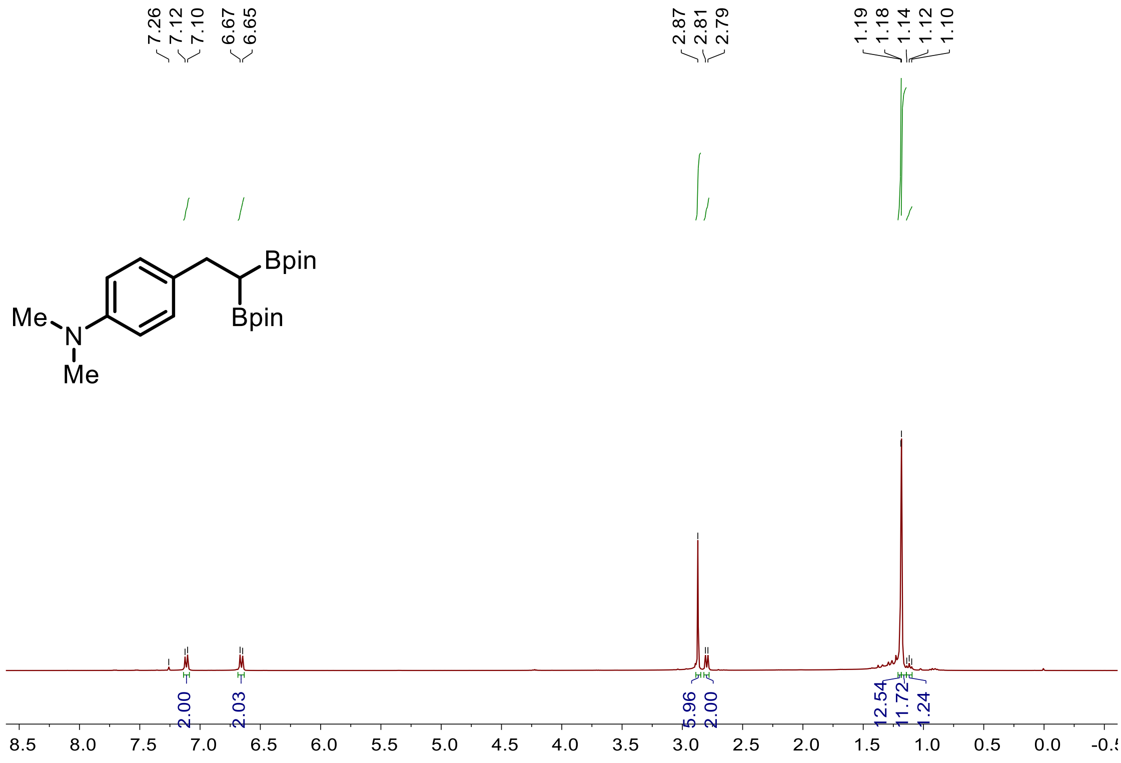

^{1}H NMR spectrum of **11** (Chloroform-*d*)

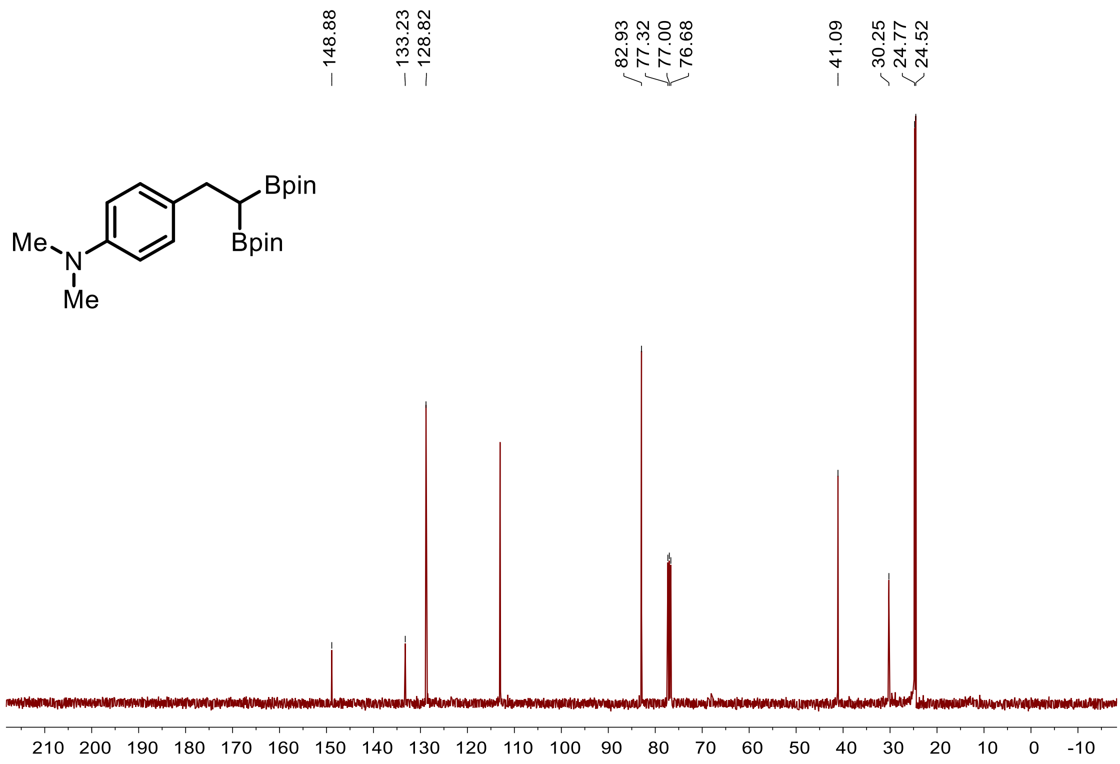

^{13}C NMR spectrum of **11** (Chloroform-*d*)

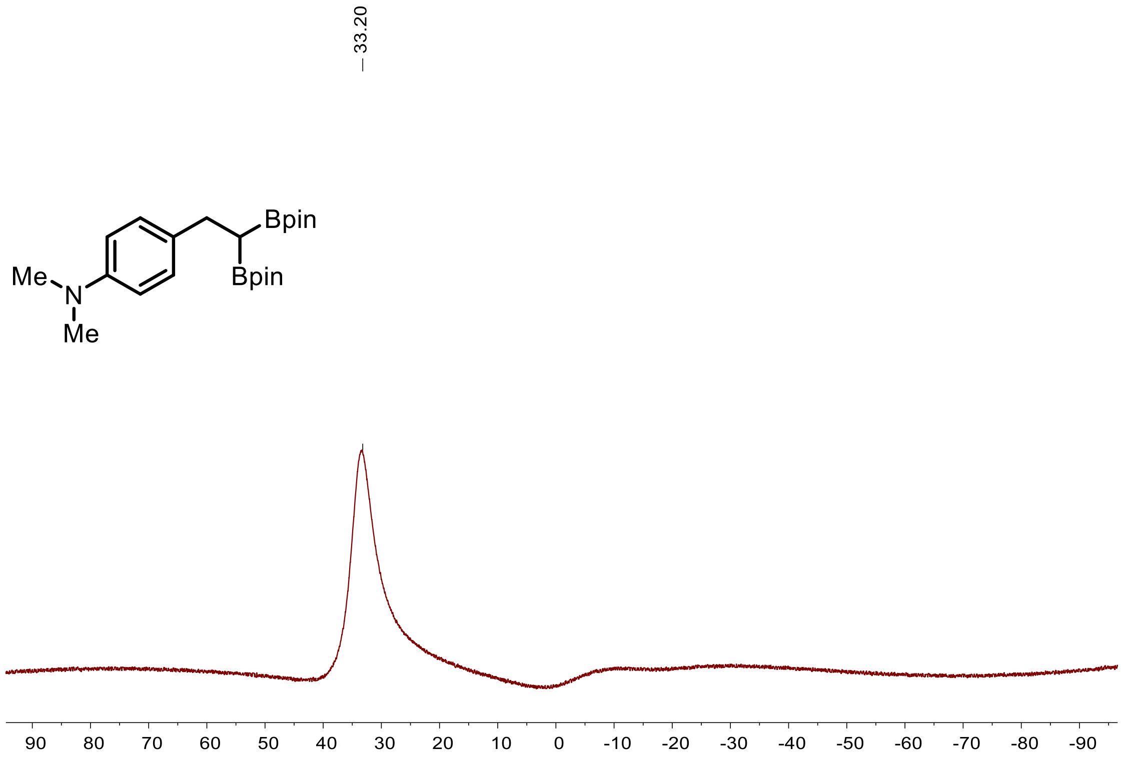

^{11}B NMR spectrum of **11** (Chloroform-*d*)

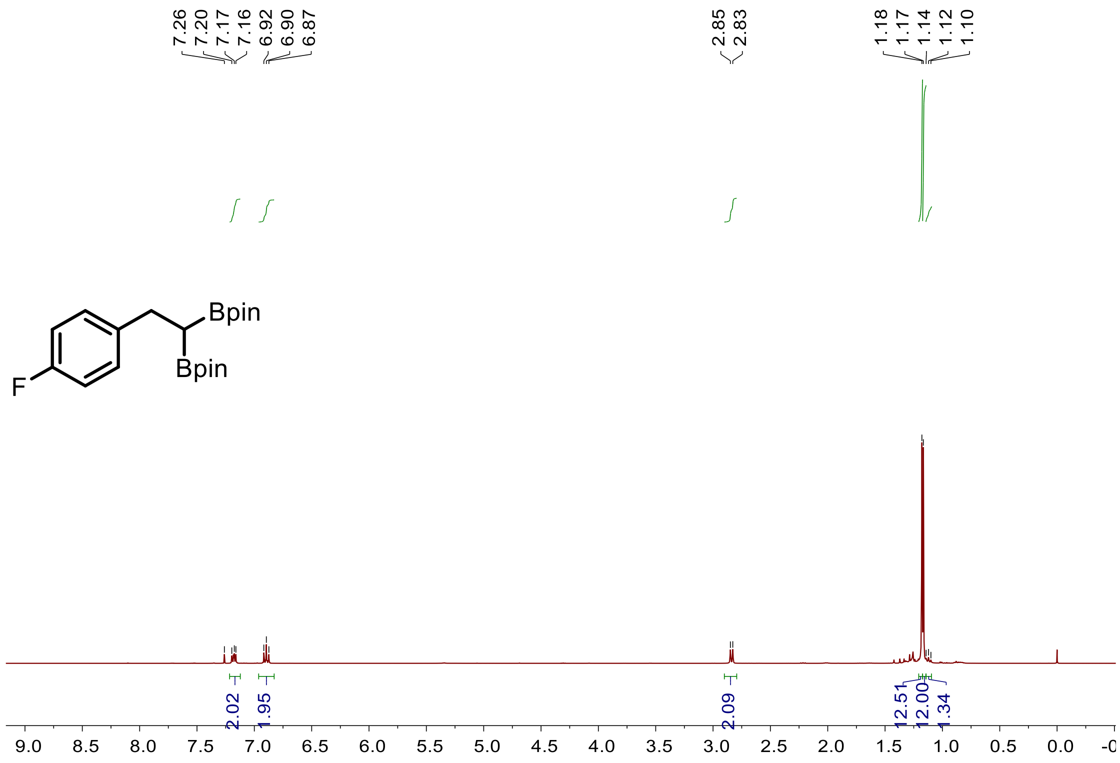

^{1}H NMR spectrum of **13** (Chloroform-*d*)

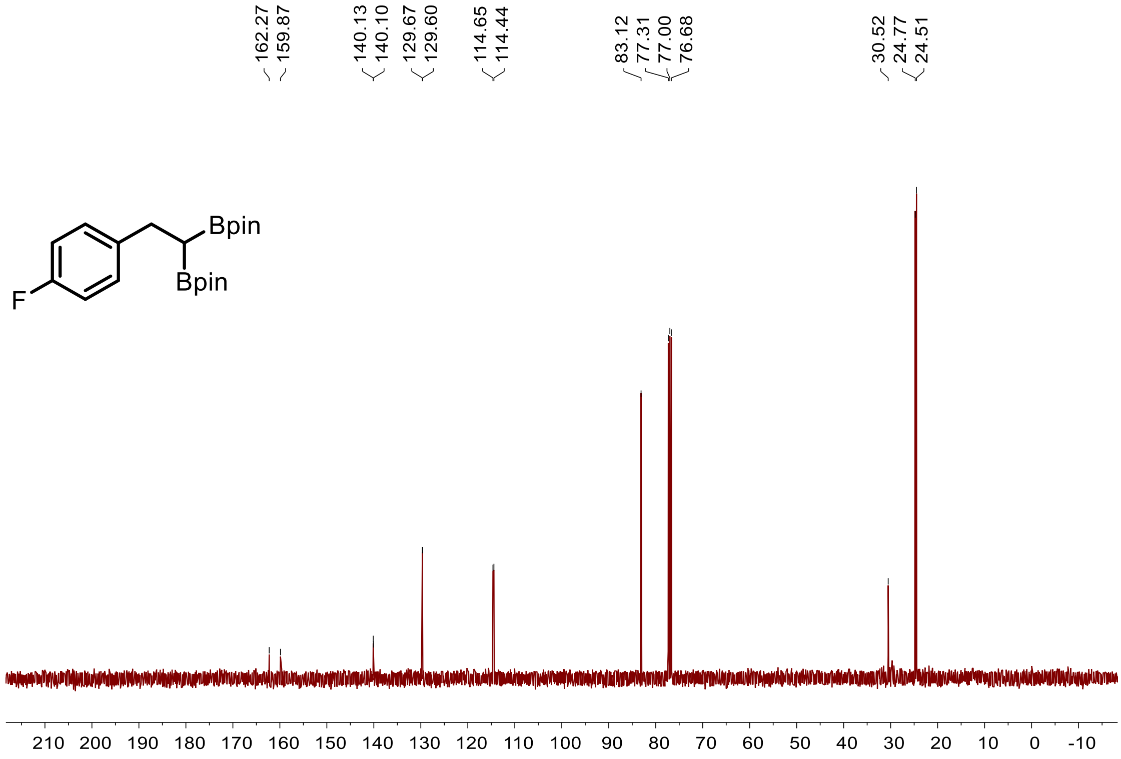

^{13}C NMR spectrum of **13** (Chloroform-*d*)

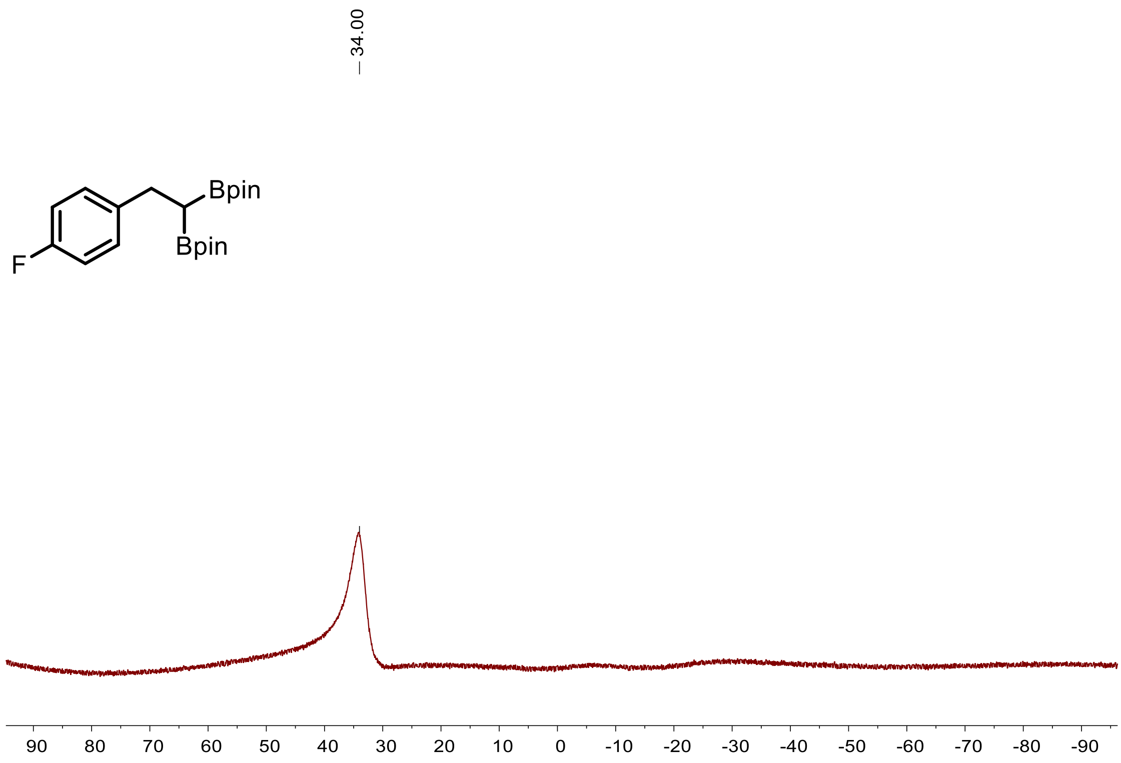

^{11}B NMR spectrum of **13** (Chloroform-*d*)

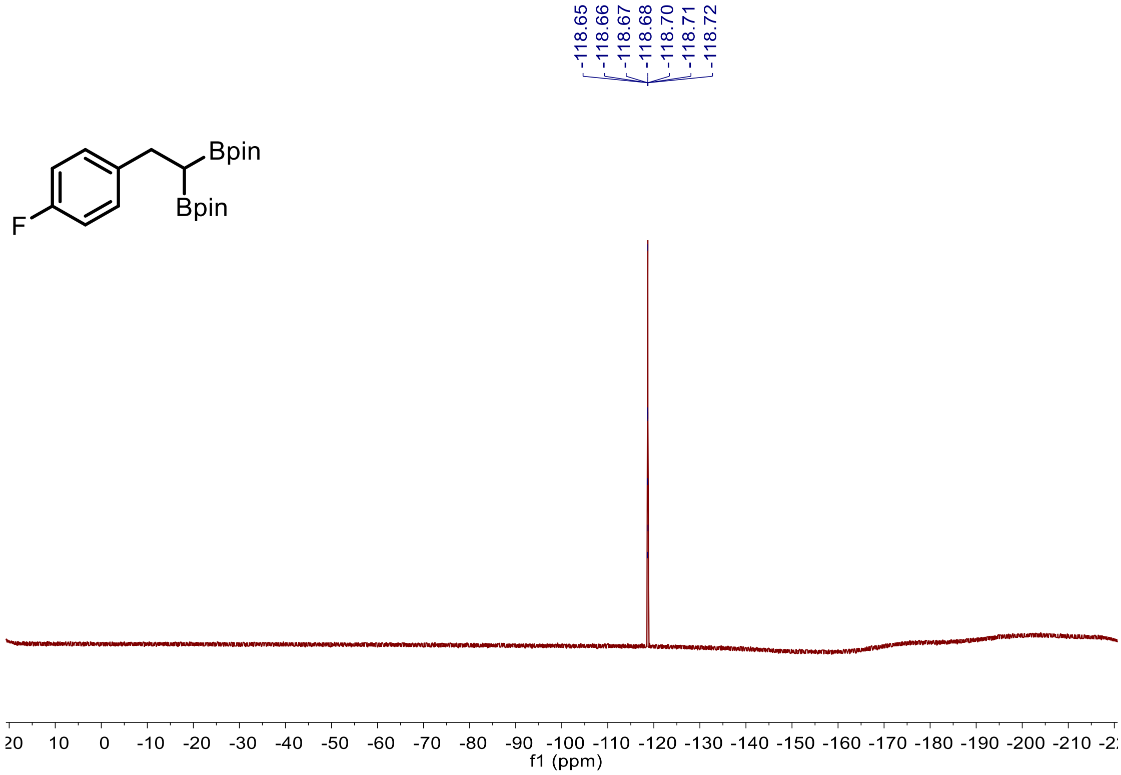

^{19}F NMR spectrum of **13** (Chloroform-*d*)

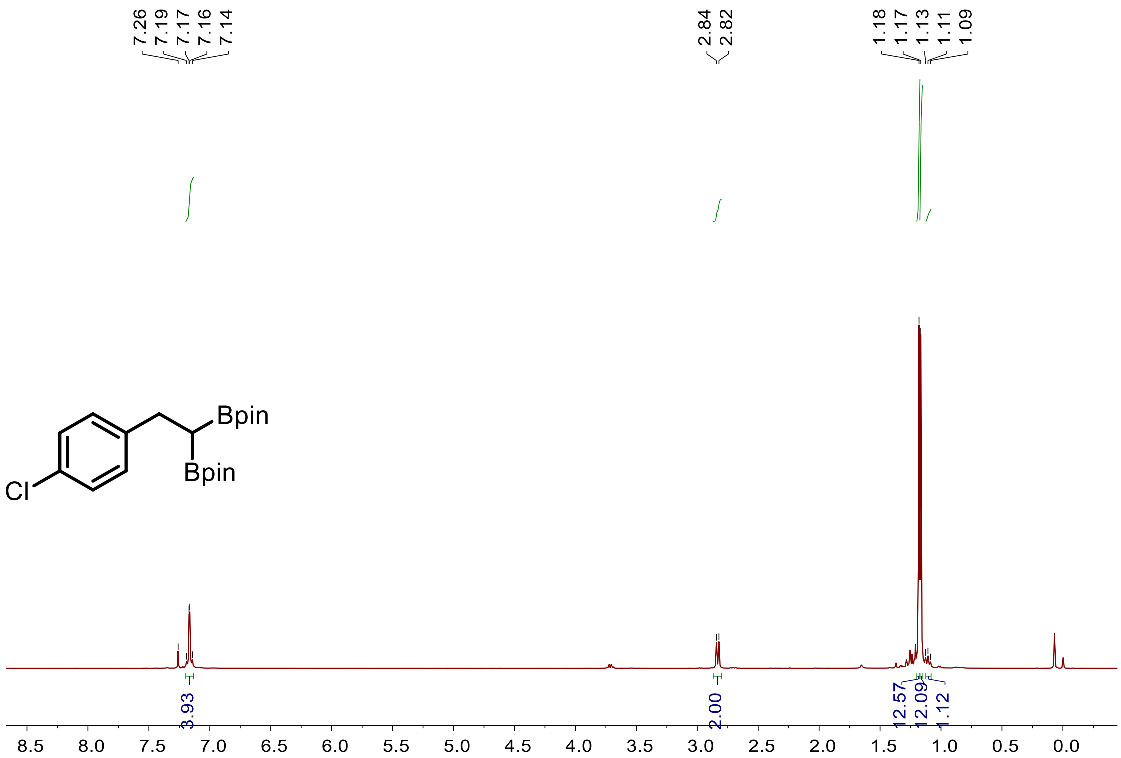

^{1}H NMR spectrum of **14** (Chloroform-*d*)

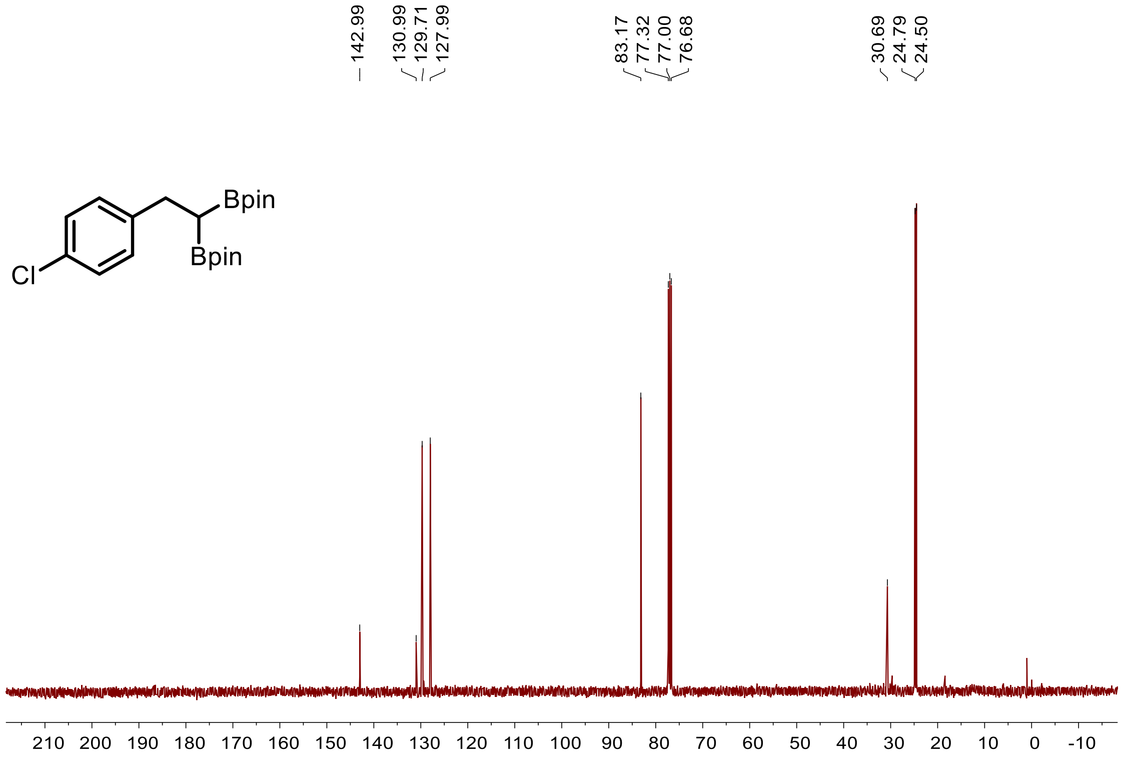

^{13}C NMR spectrum of **14** (Chloroform-*d*)

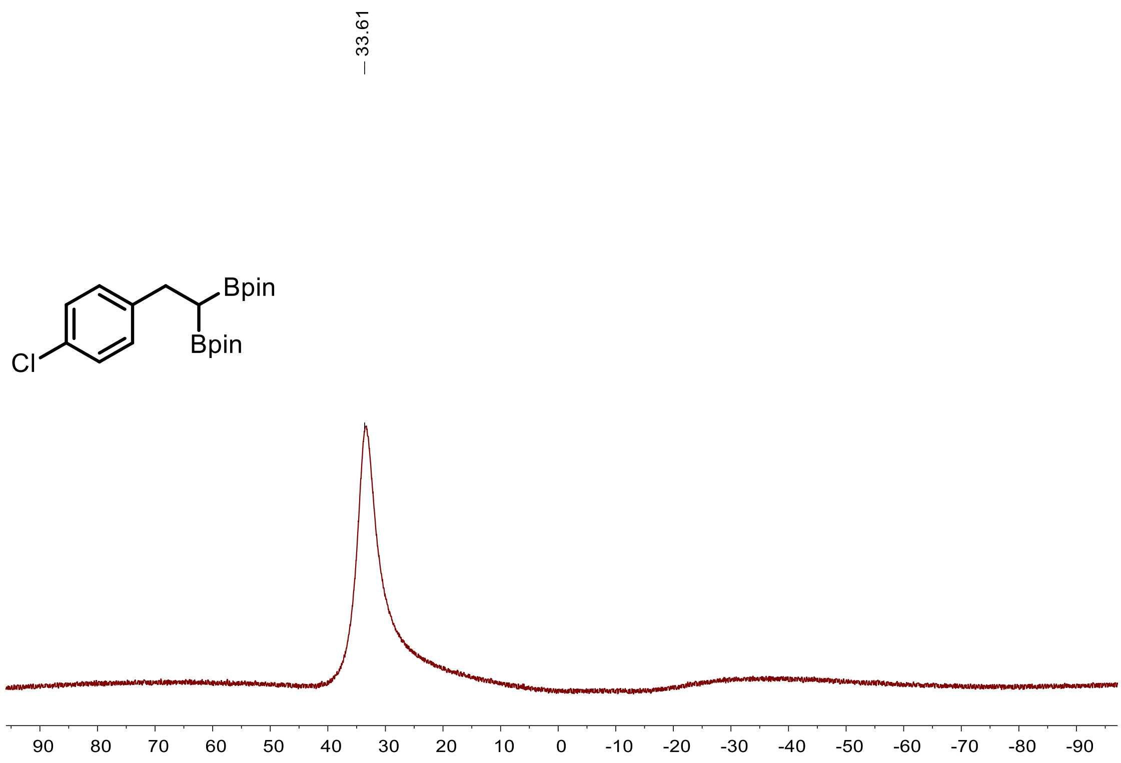

^{11}B NMR spectrum of **14** (Chloroform-*d*)

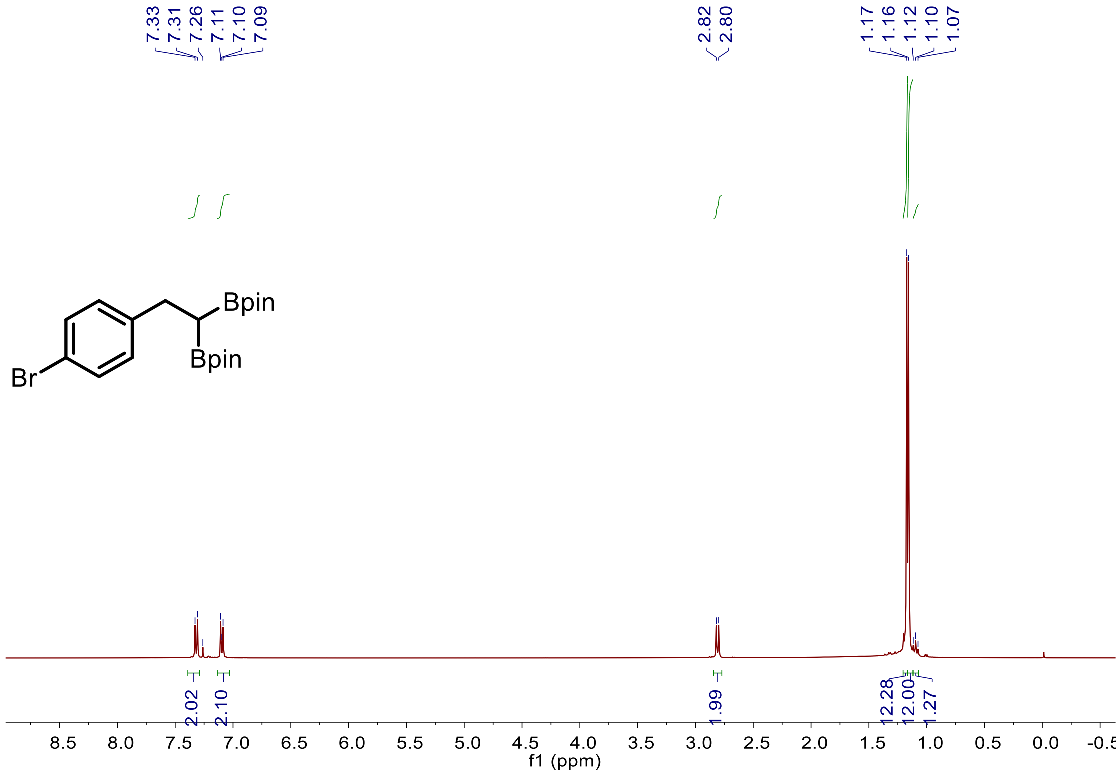

^{1}H NMR spectrum of **15** (Chloroform-*d*)

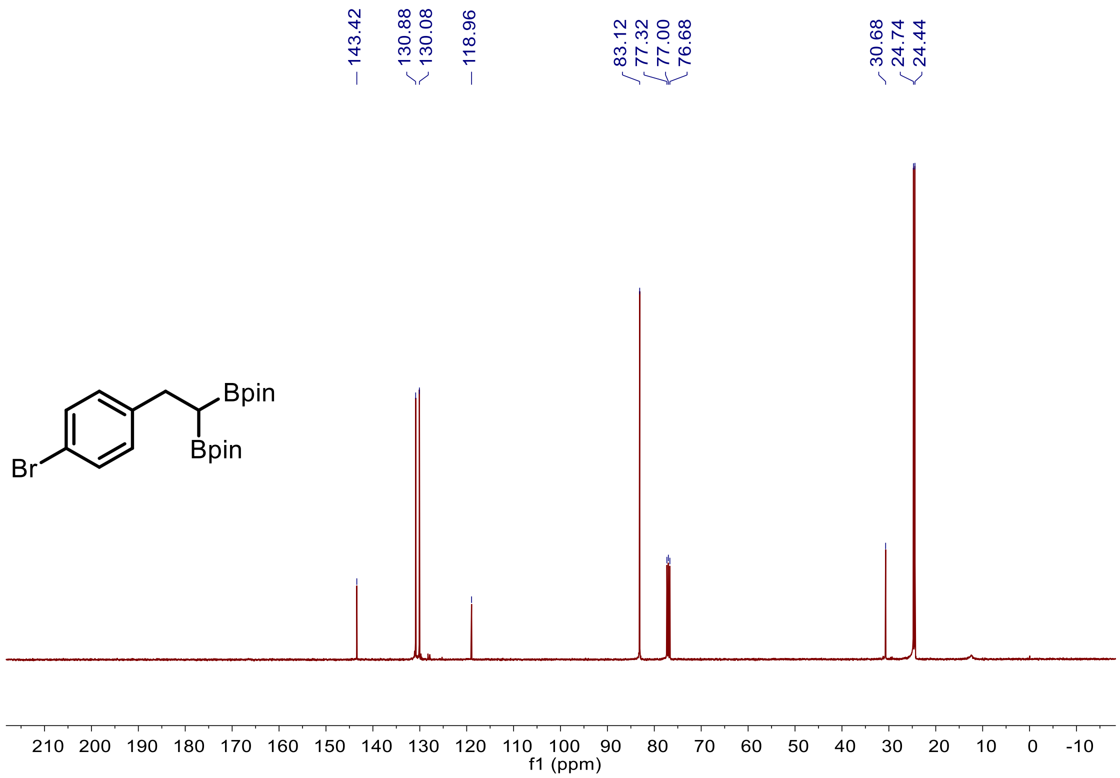

^{13}C NMR spectrum of **15** (Chloroform-*d*)

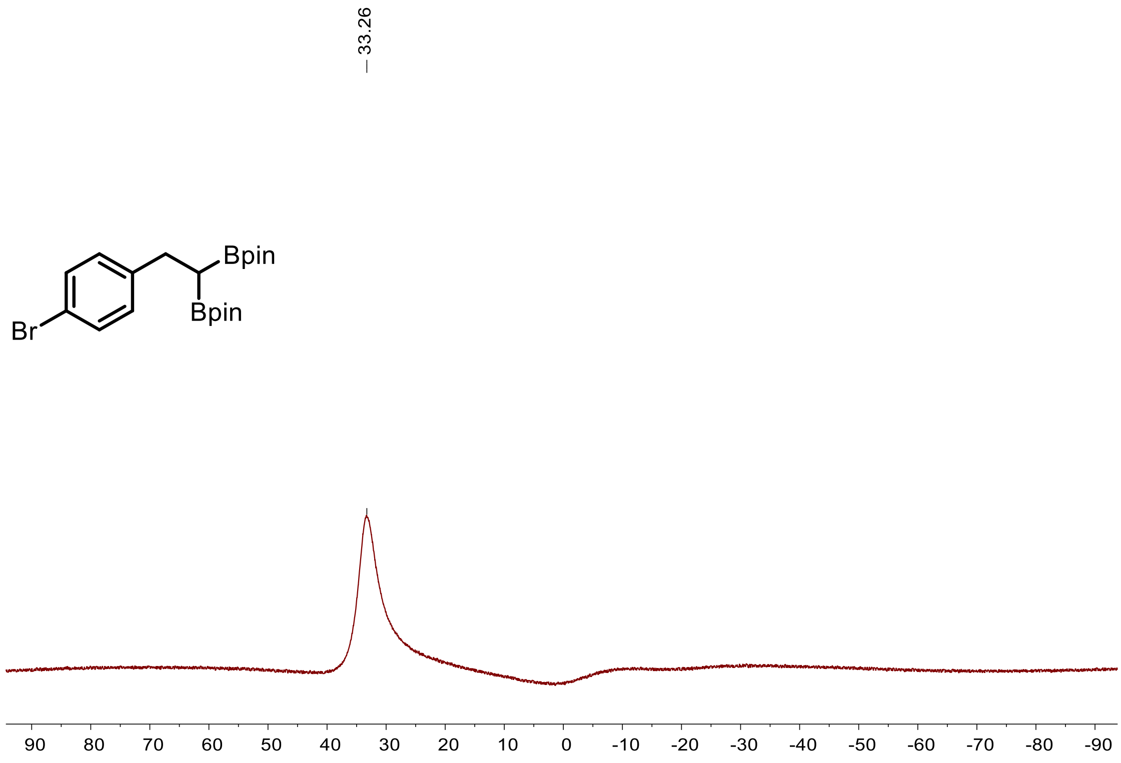

^{11}B NMR spectrum of **15** (Chloroform-*d*)

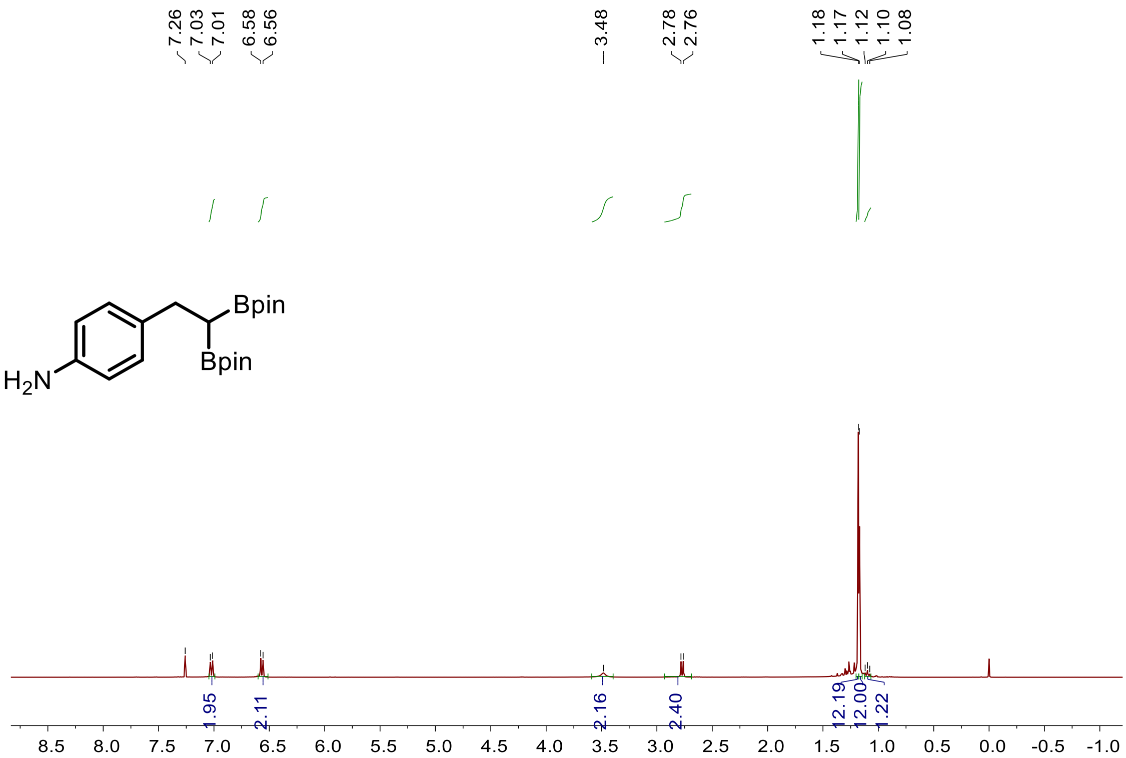

^{1}H NMR spectrum of **16** (Chloroform-*d*)

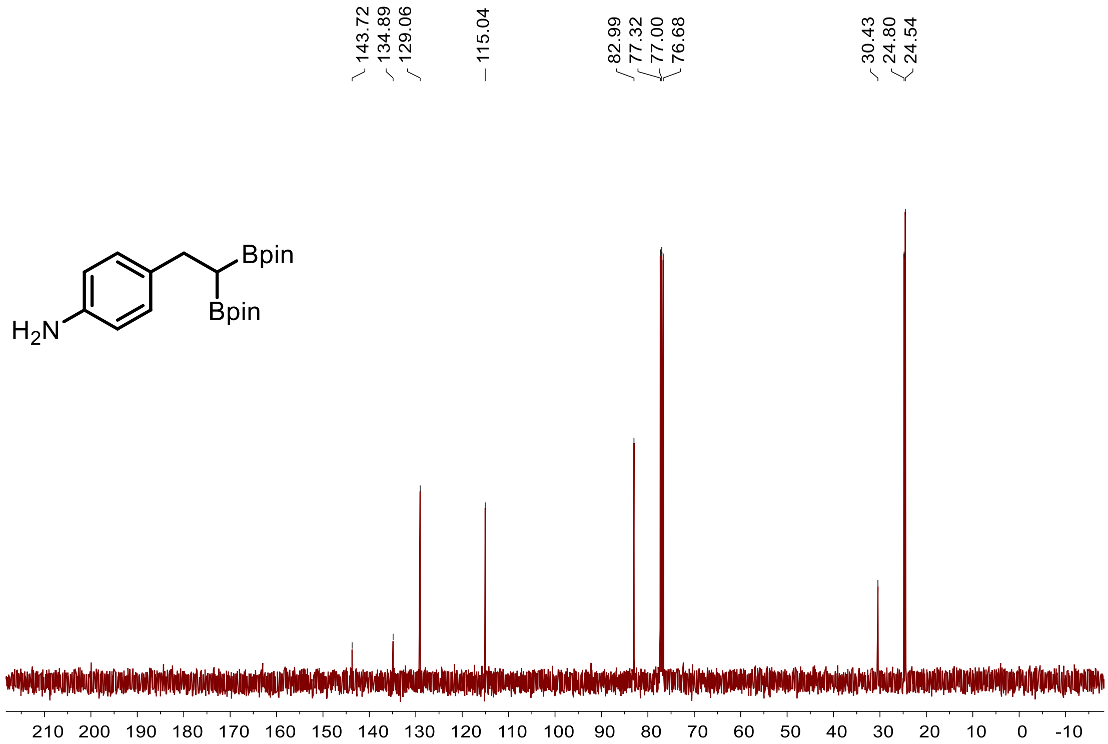

^{13}C NMR spectrum of **16** (Chloroform-*d*)

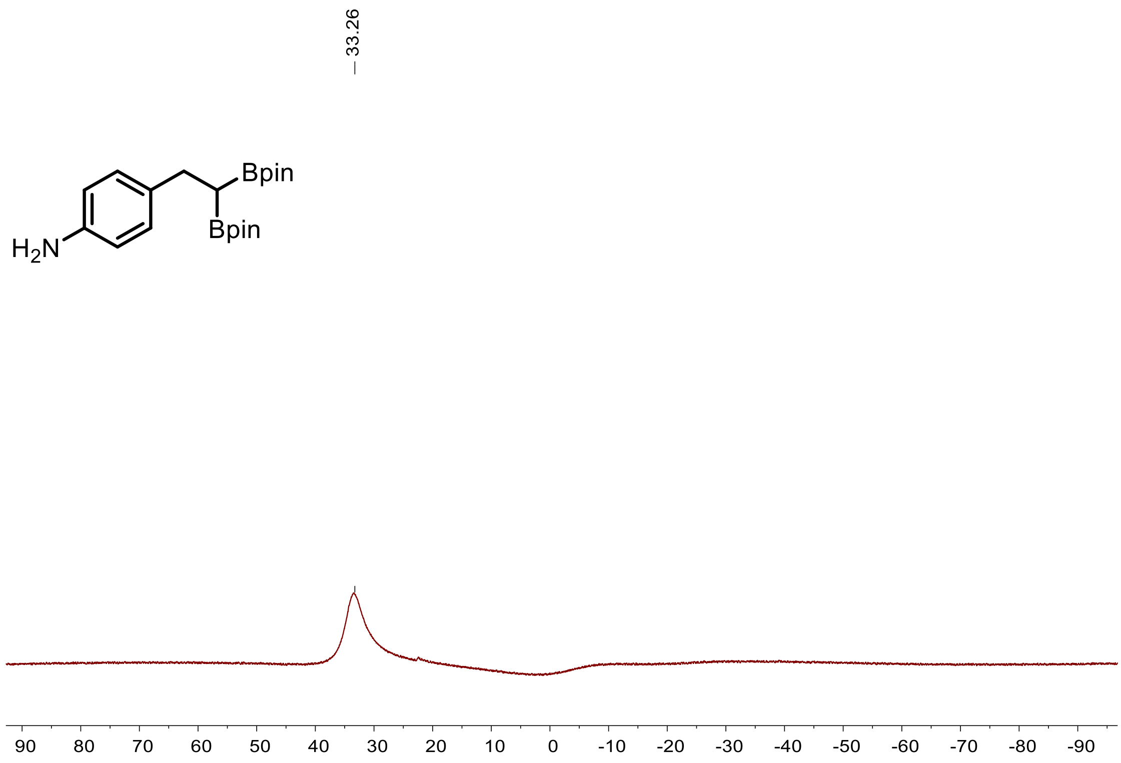

^{11}B NMR spectrum of **16** (Chloroform-*d*)

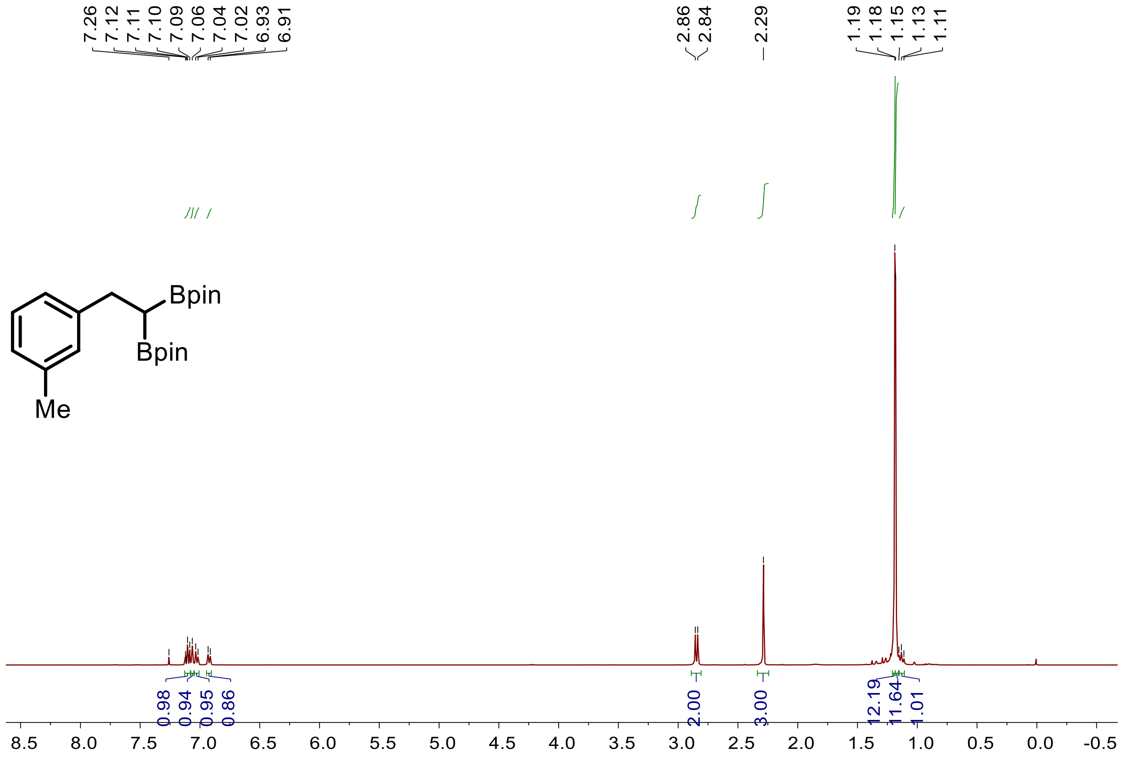

^{1}H NMR spectrum of **17** (Chloroform-*d*)

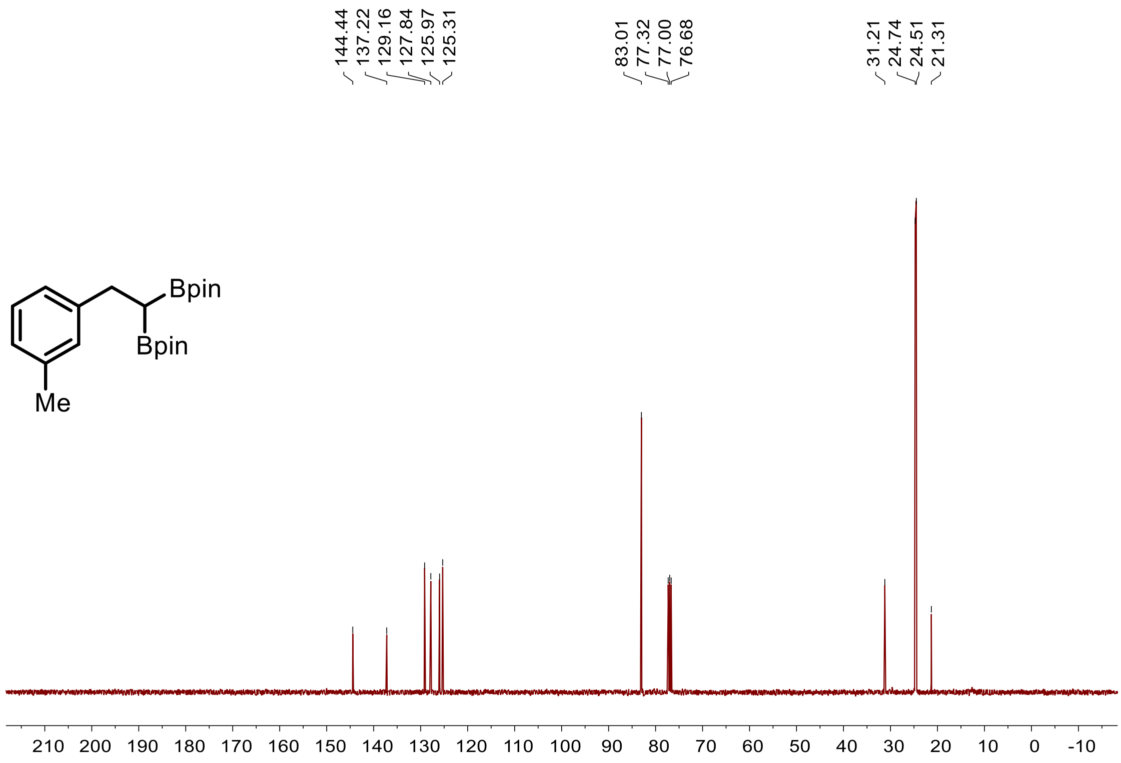

^{13}C NMR spectrum of **17** (Chloroform-*d*)

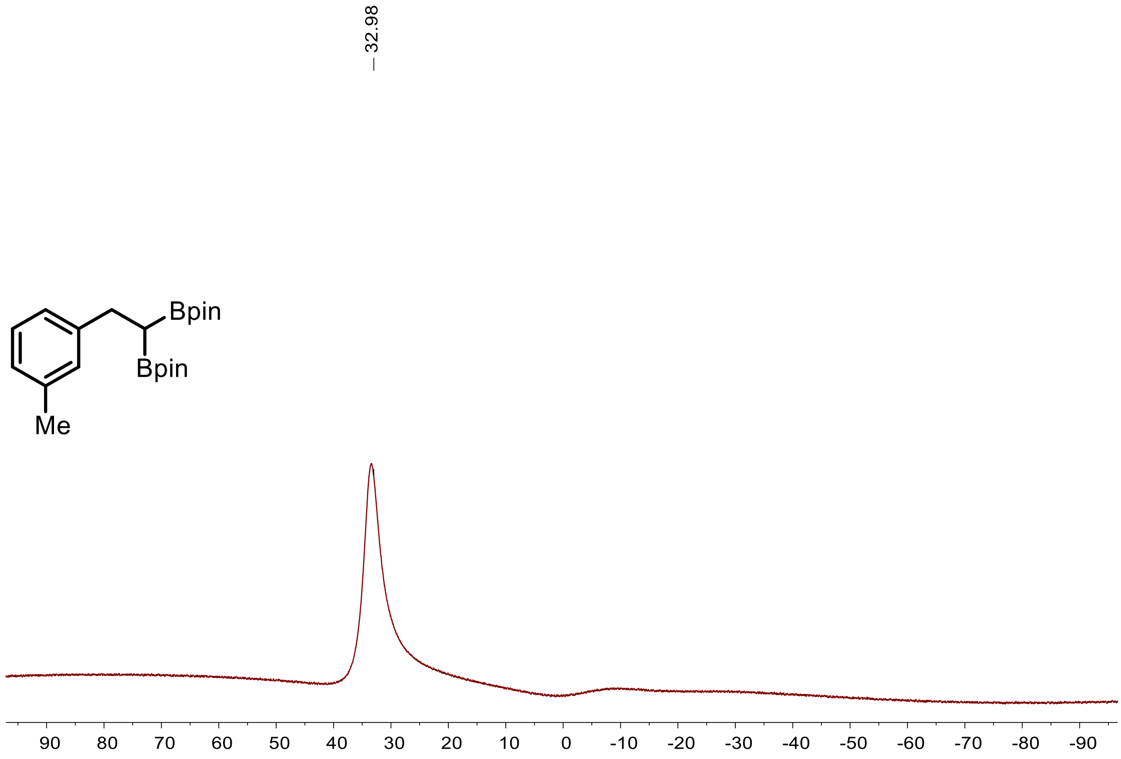

^{11}B NMR spectrum of **17** (Chloroform-*d*)

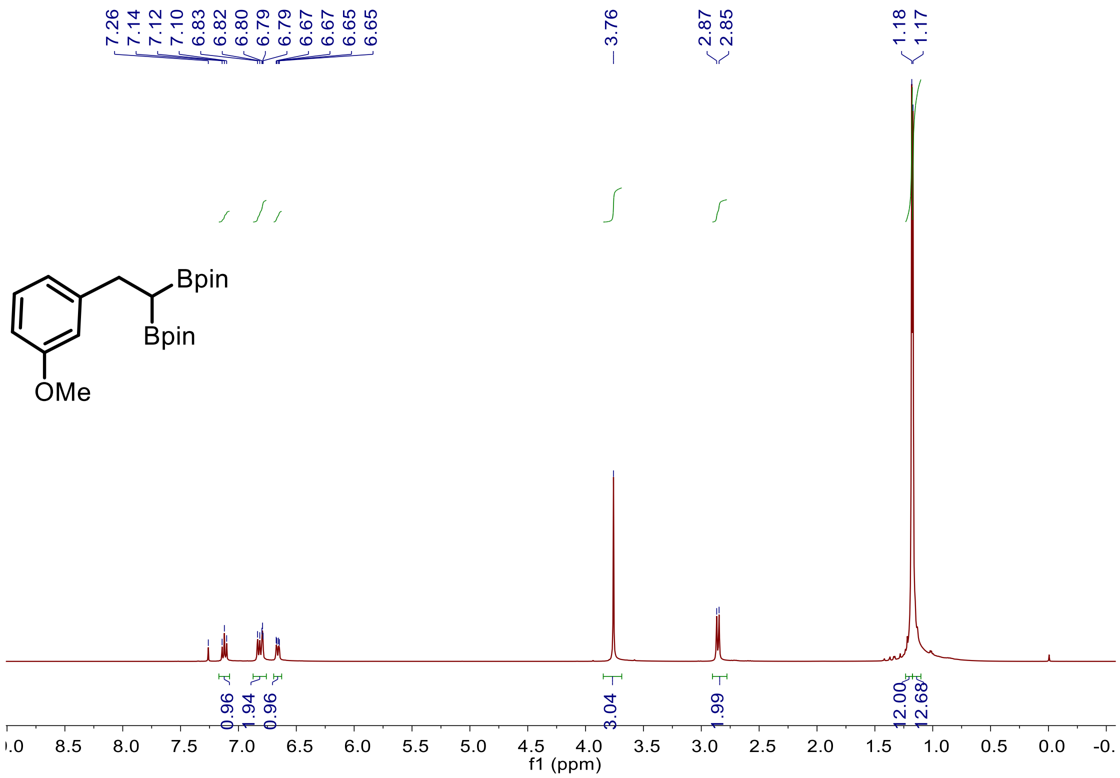

^{1}H NMR spectrum of **18** (Chloroform-*d*)

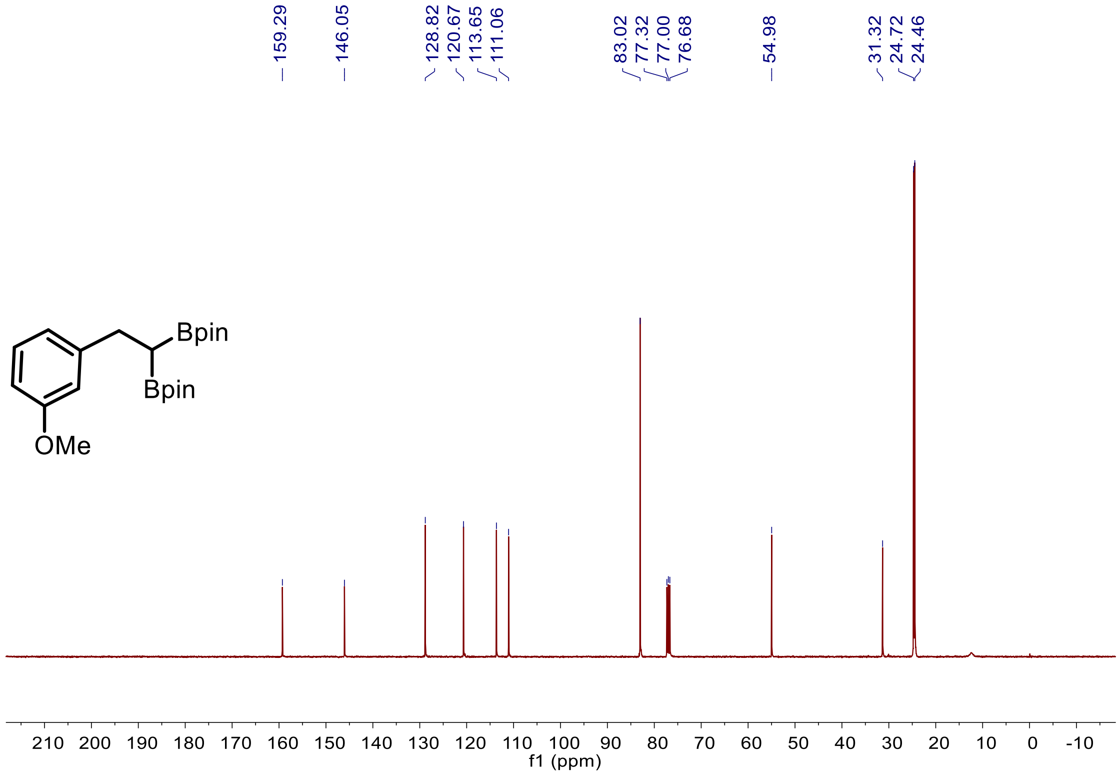

^{13}C NMR spectrum of **18** (Chloroform-*d*)

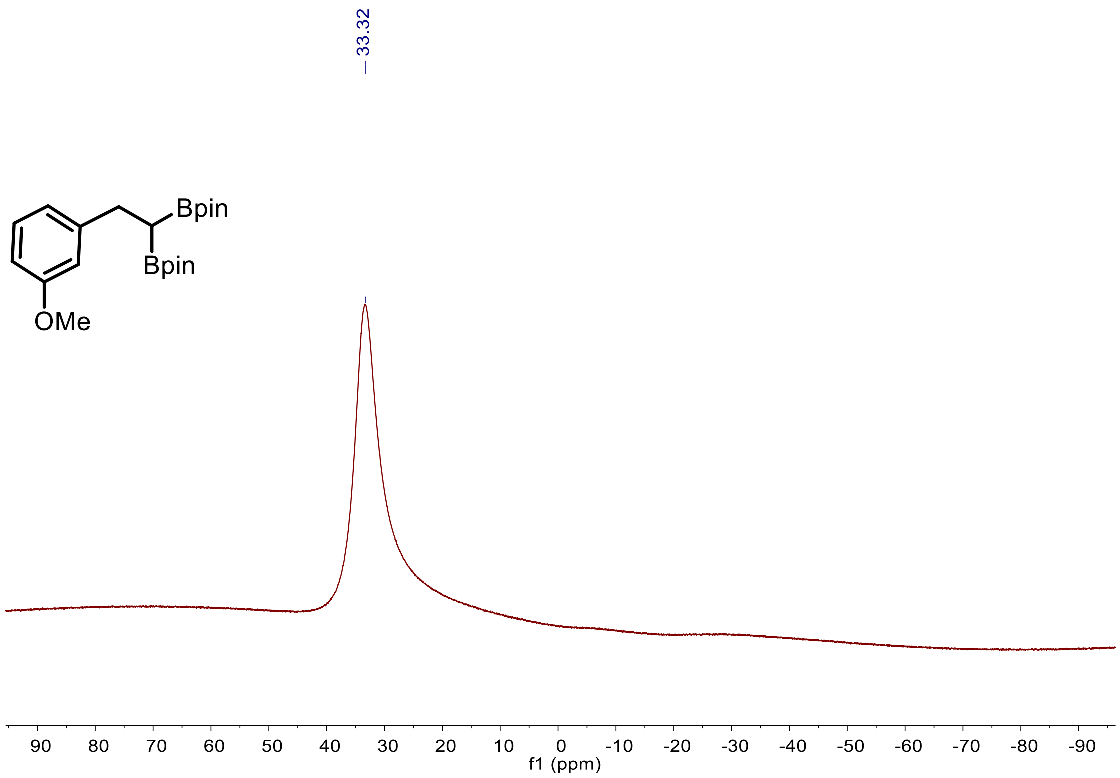

^{11}B NMR spectrum of **18** (Chloroform-*d*)

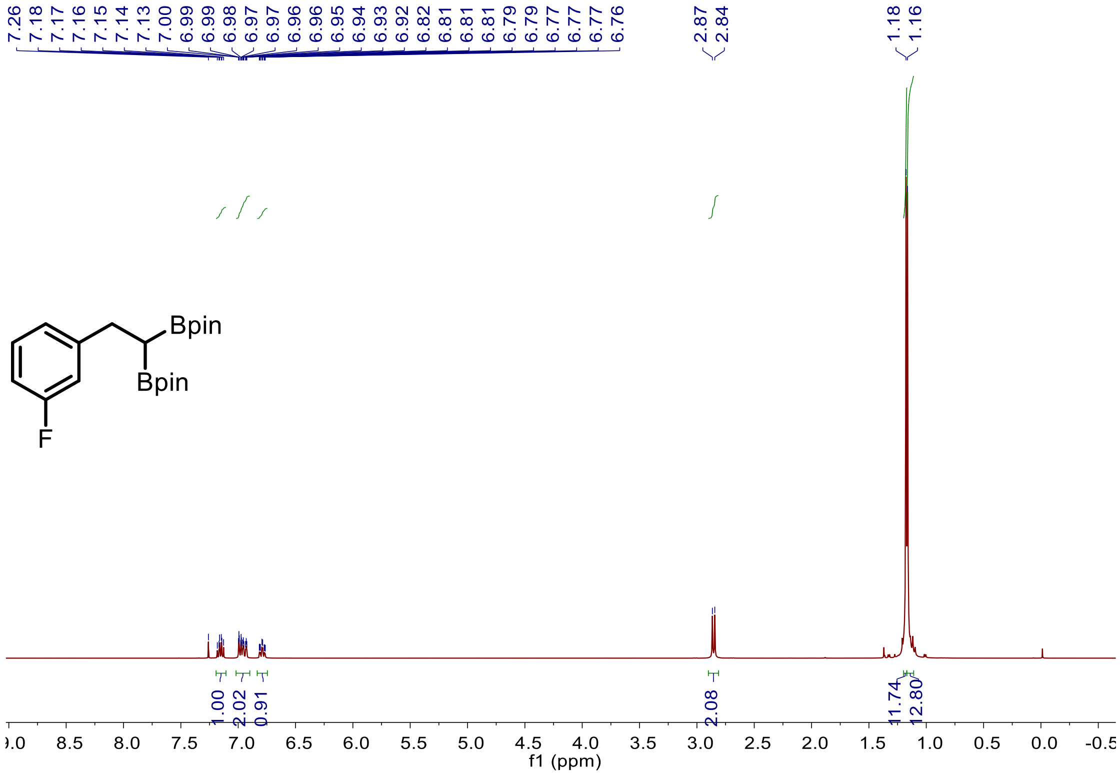

^{1}H NMR spectrum of **19** (Chloroform-*d*)

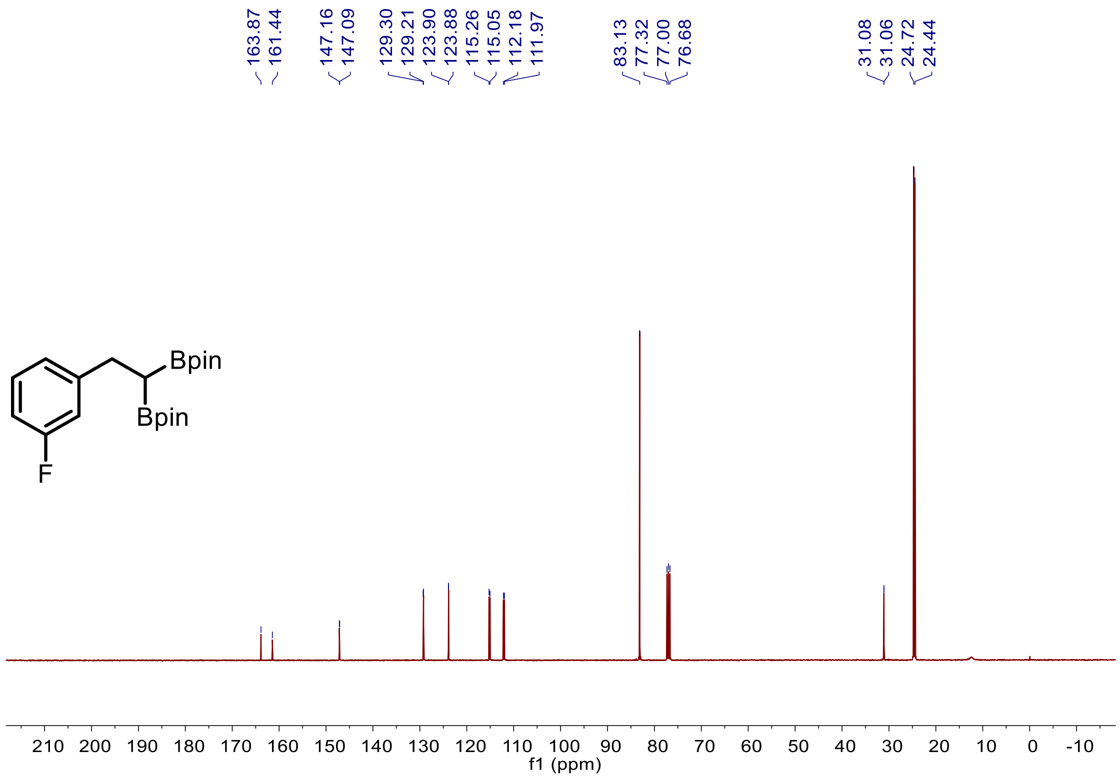

^{13}C NMR spectrum of **19** (Chloroform-*d*)

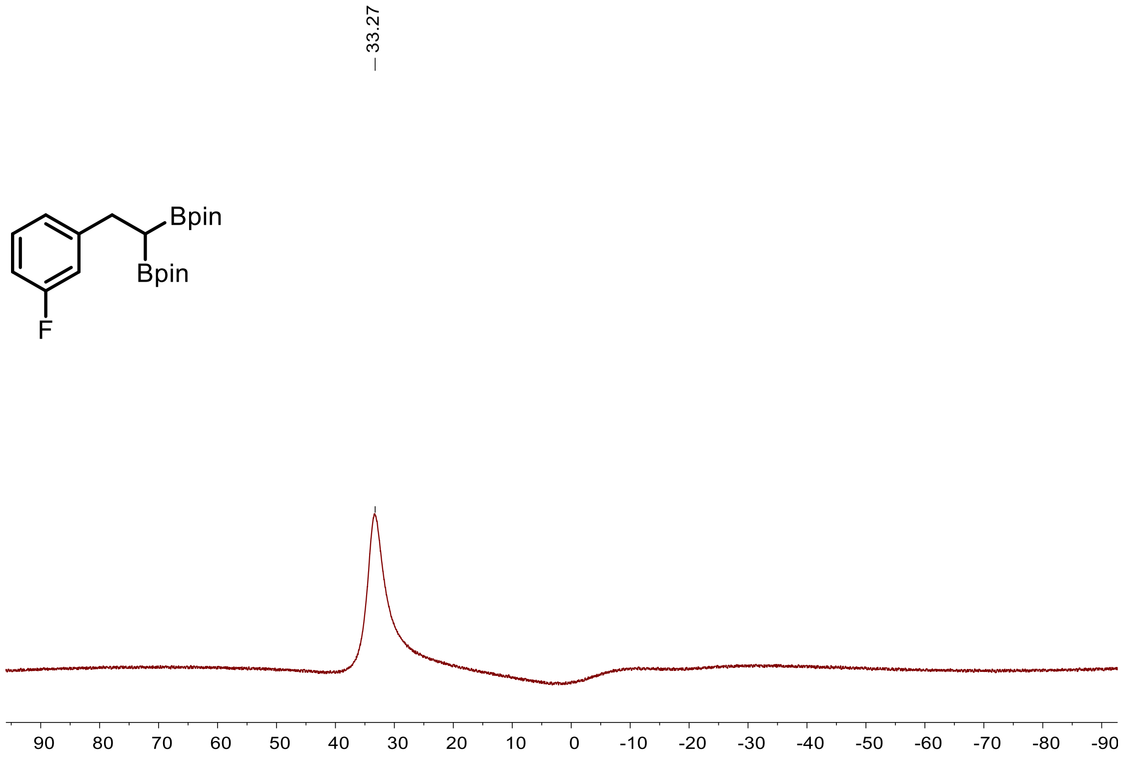

^{11}B NMR spectrum of **19** (Chloroform-*d*)

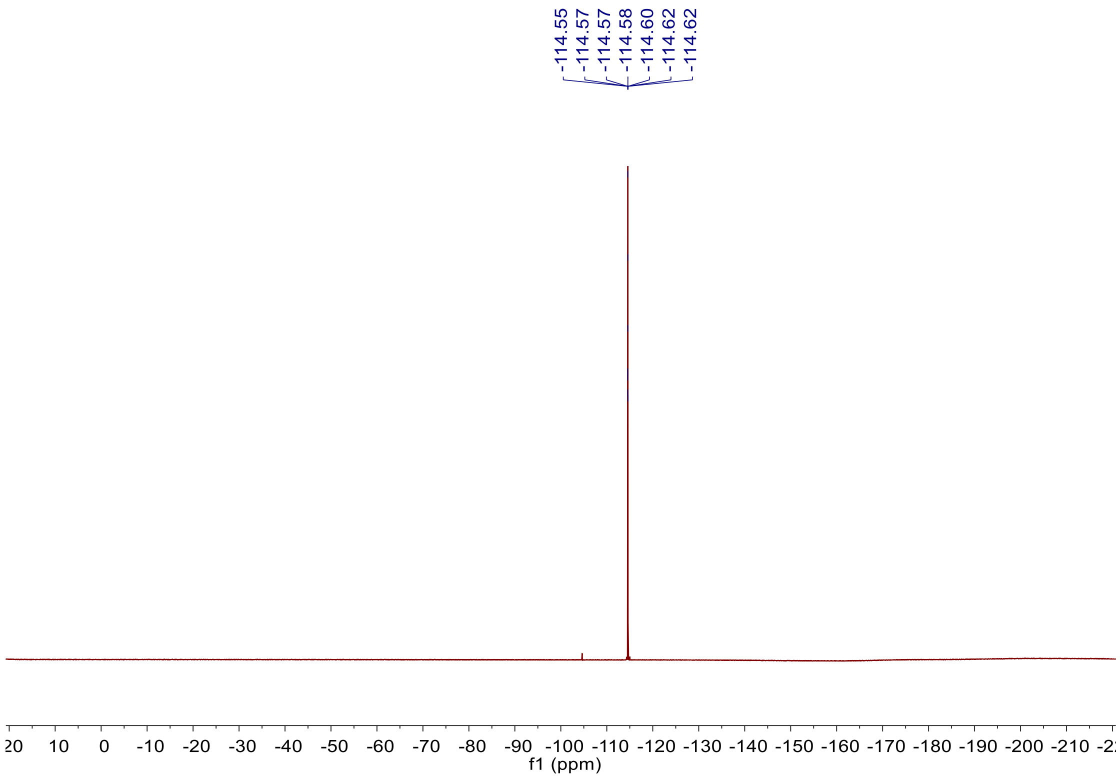

^{19}F NMR spectrum of **19** (Chloroform-*d*)

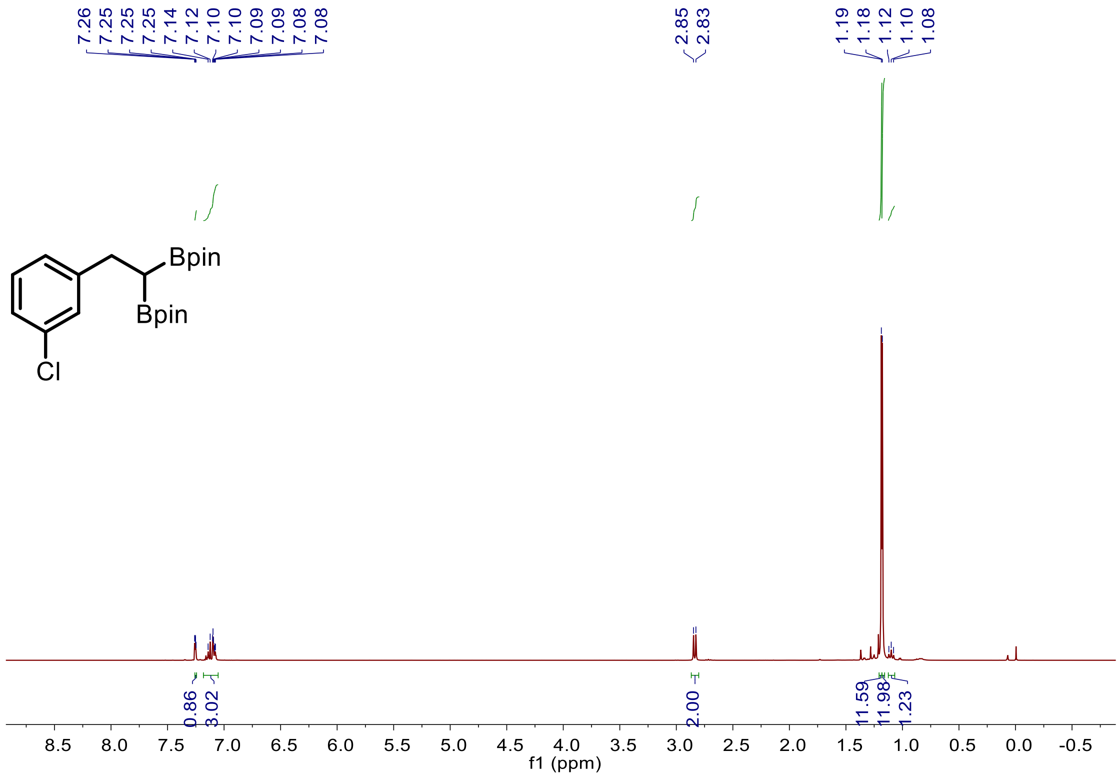

^{1}H NMR spectrum of **20** (Chloroform-*d*)

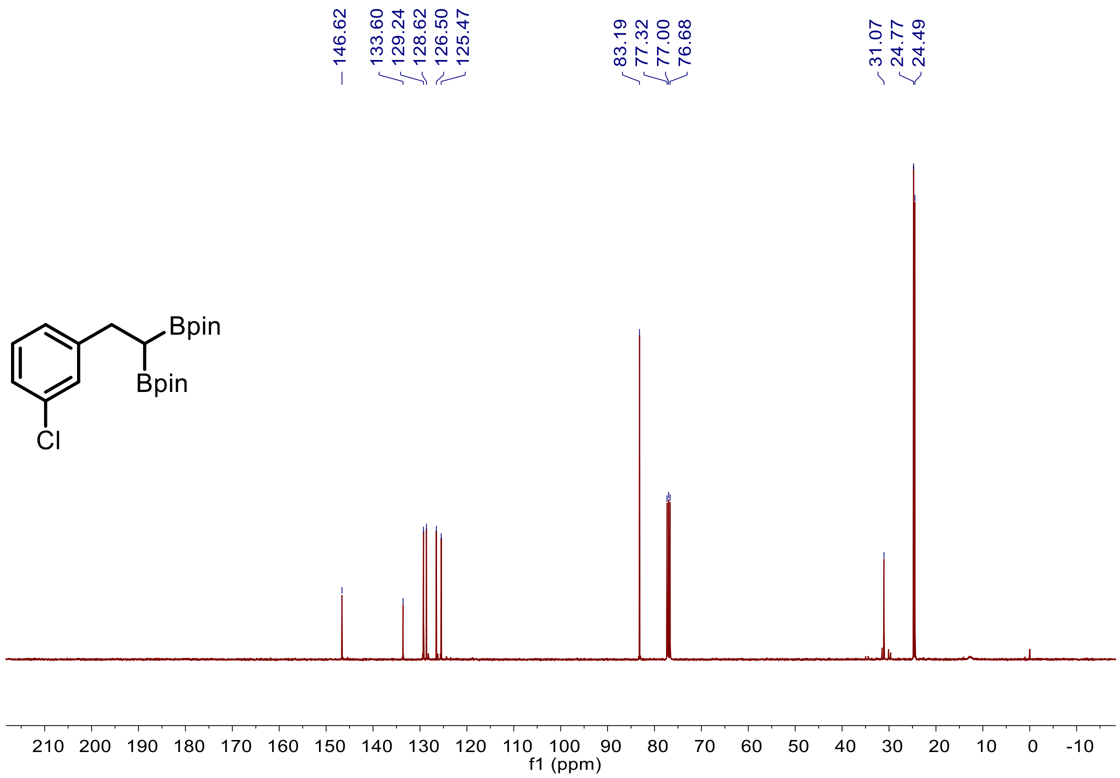

^{13}C NMR spectrum of **20** (Chloroform-*d*)

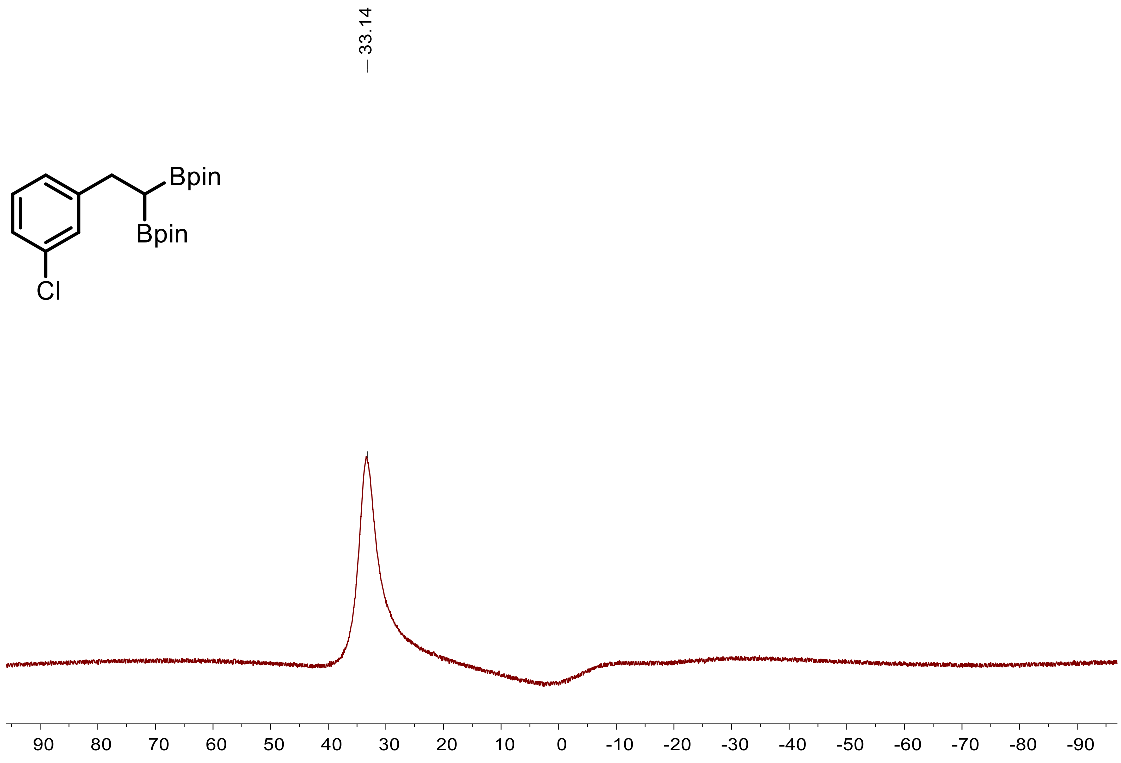

^{11}B NMR spectrum of **20** (Chloroform-*d*)

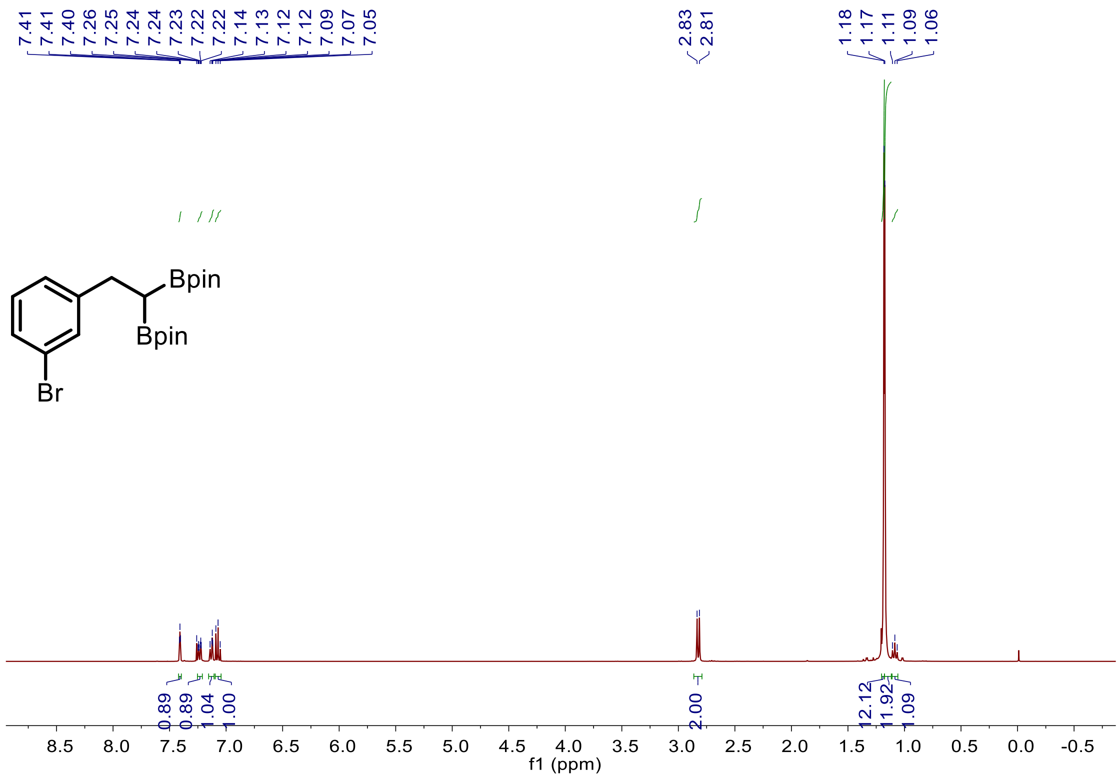

^{1}H NMR spectrum of **21** (Chloroform-*d*)

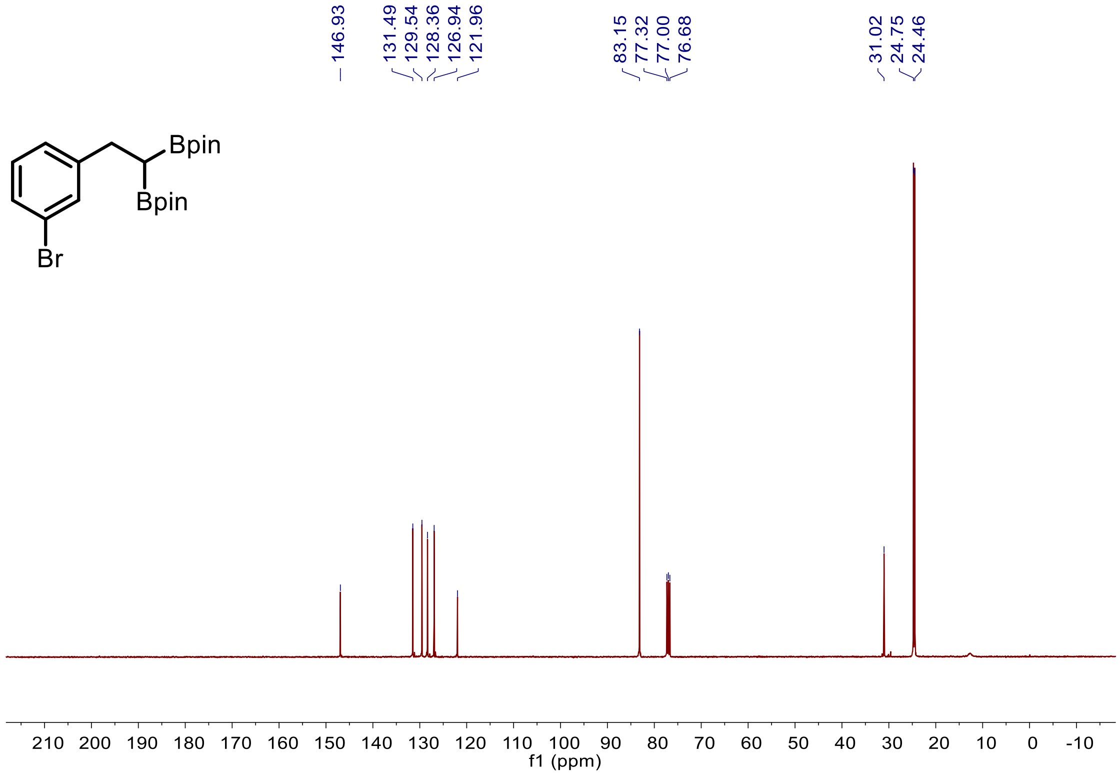

^{13}C NMR spectrum of **21** (Chloroform-*d*)

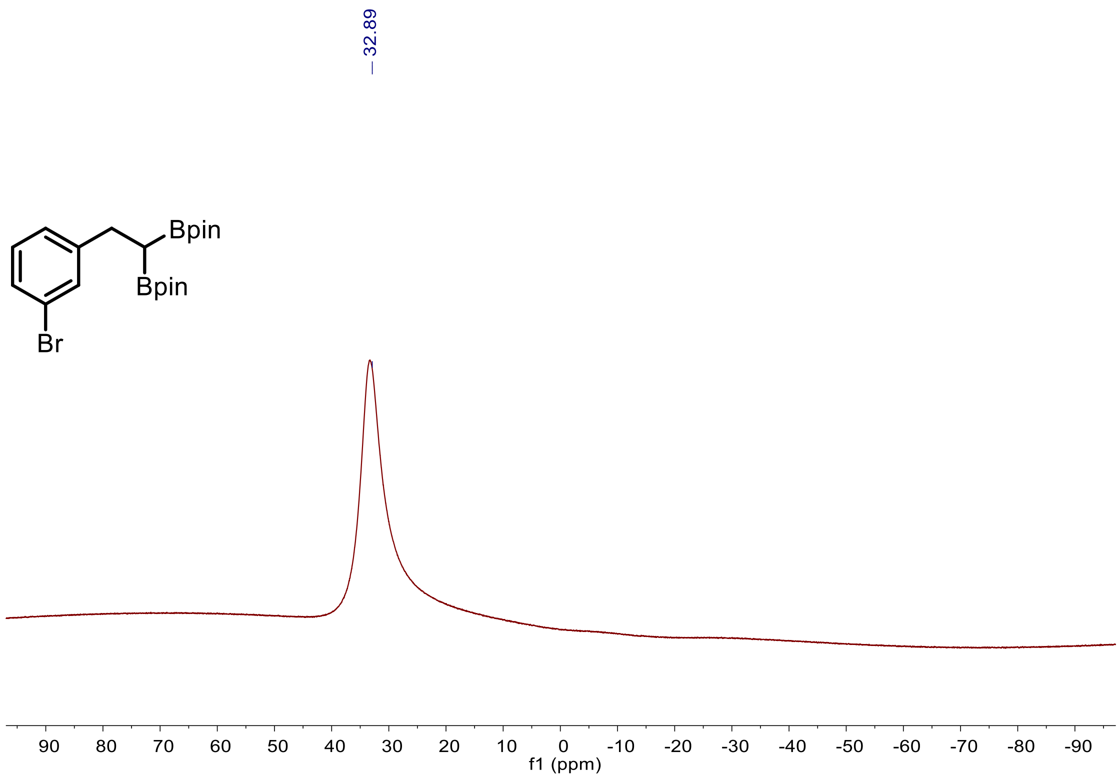

^{11}B NMR spectrum of **21** (Chloroform-*d*)

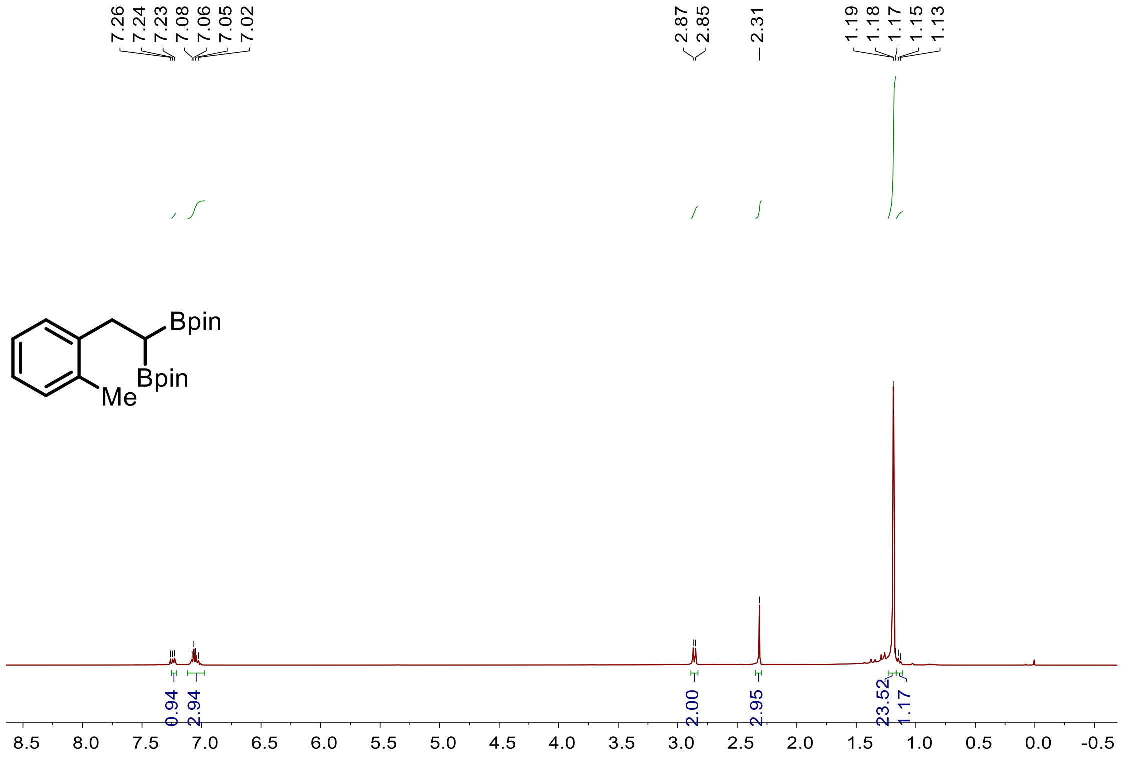

^{1}H NMR spectrum of **22** (Chloroform-*d*)

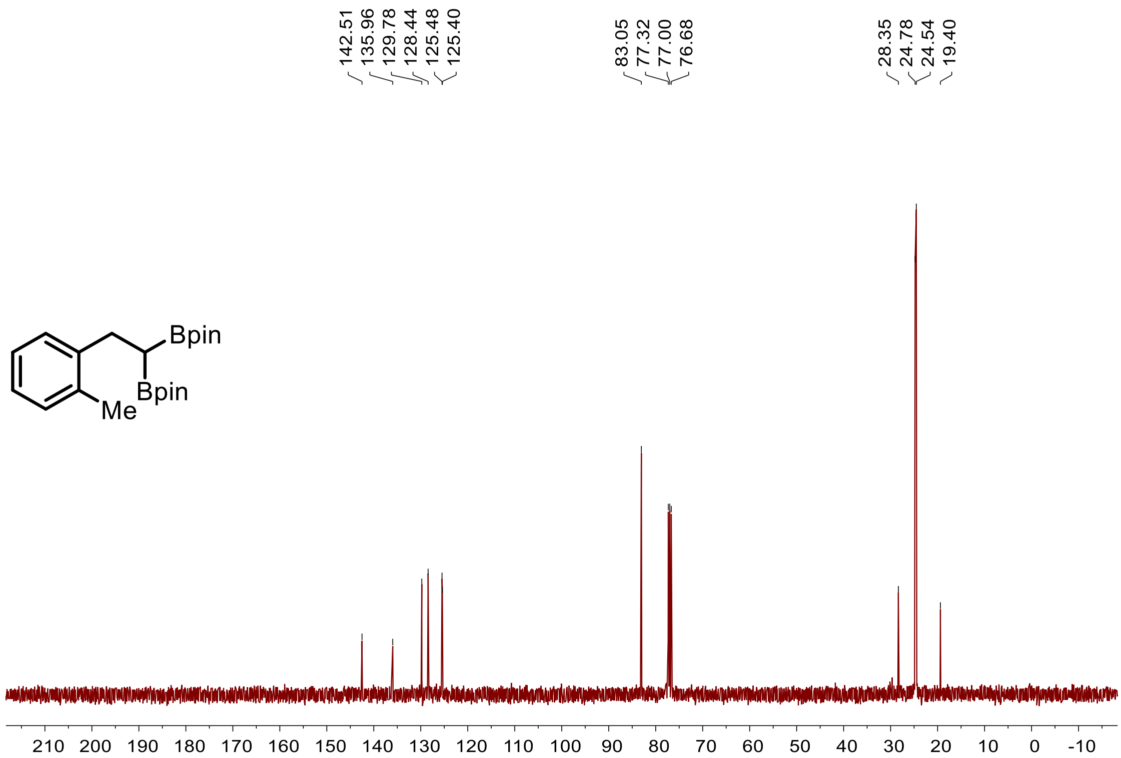

^{13}C NMR spectrum of **22** (Chloroform-*d*)

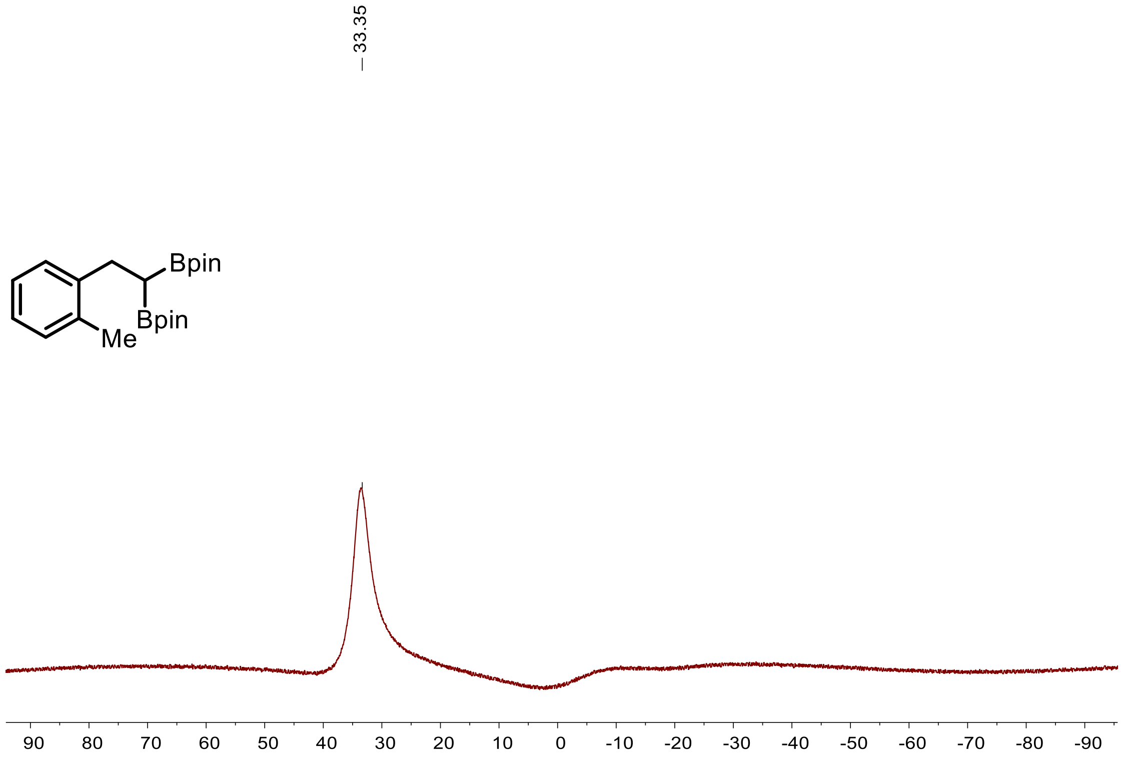

^{11}B NMR spectrum of **22** (Chloroform-*d*)

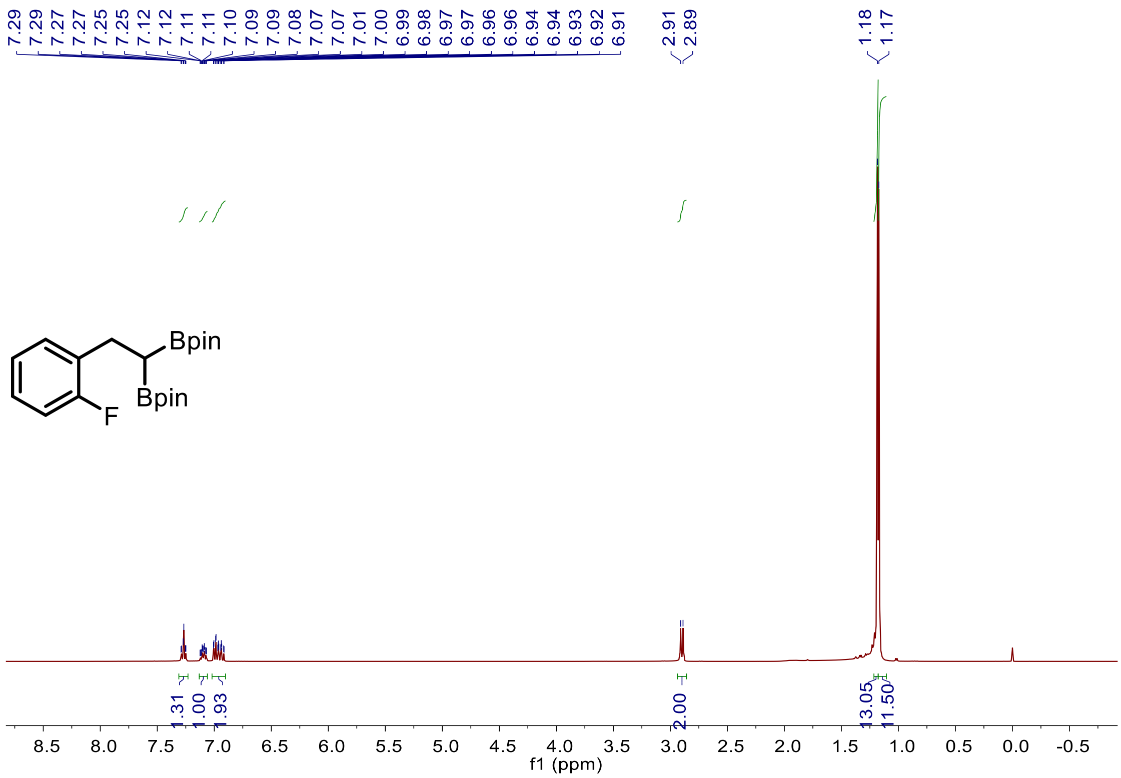

^{1}H NMR spectrum of **24** (Chloroform-*d*)

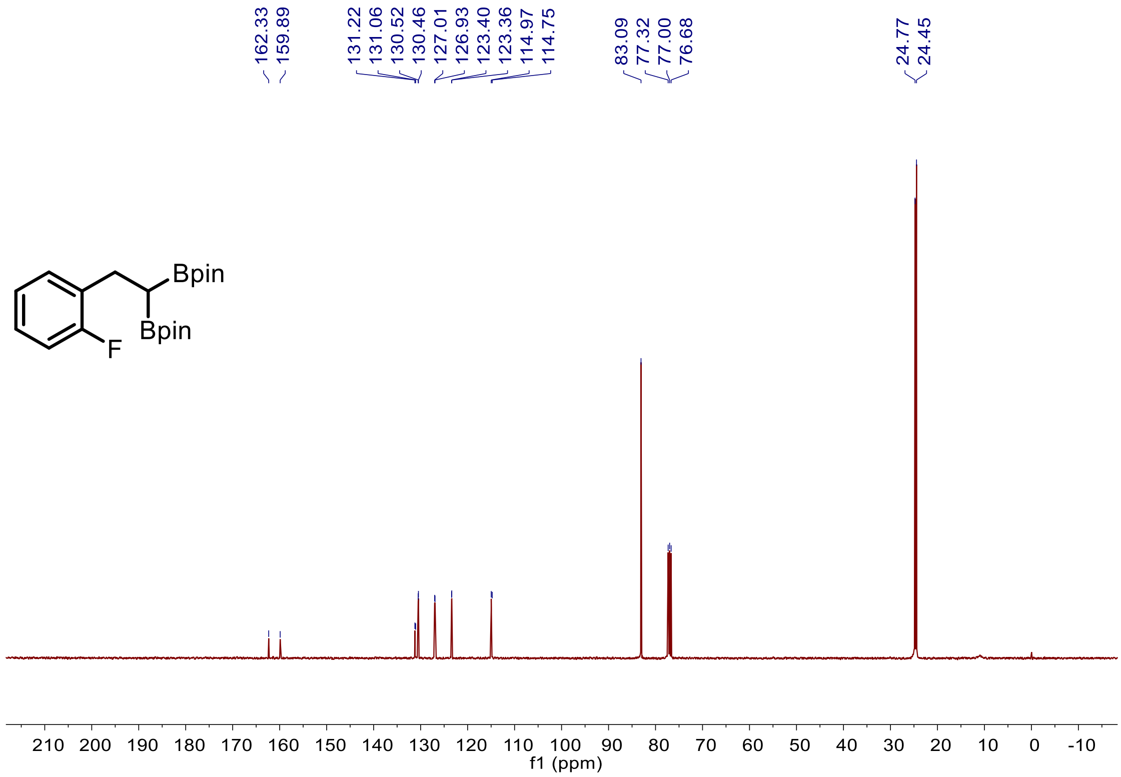

^{13}C NMR spectrum of **24** (Chloroform-*d*)

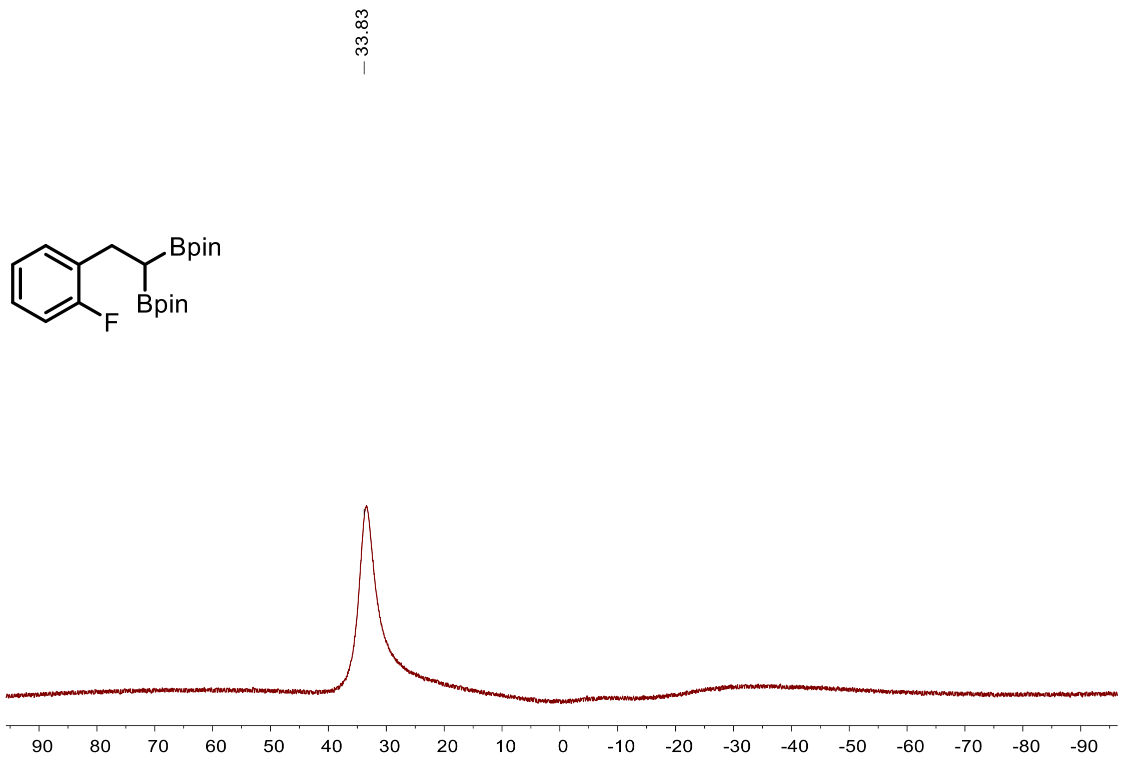

^{11}B NMR spectrum of **24** (Chloroform-*d*)

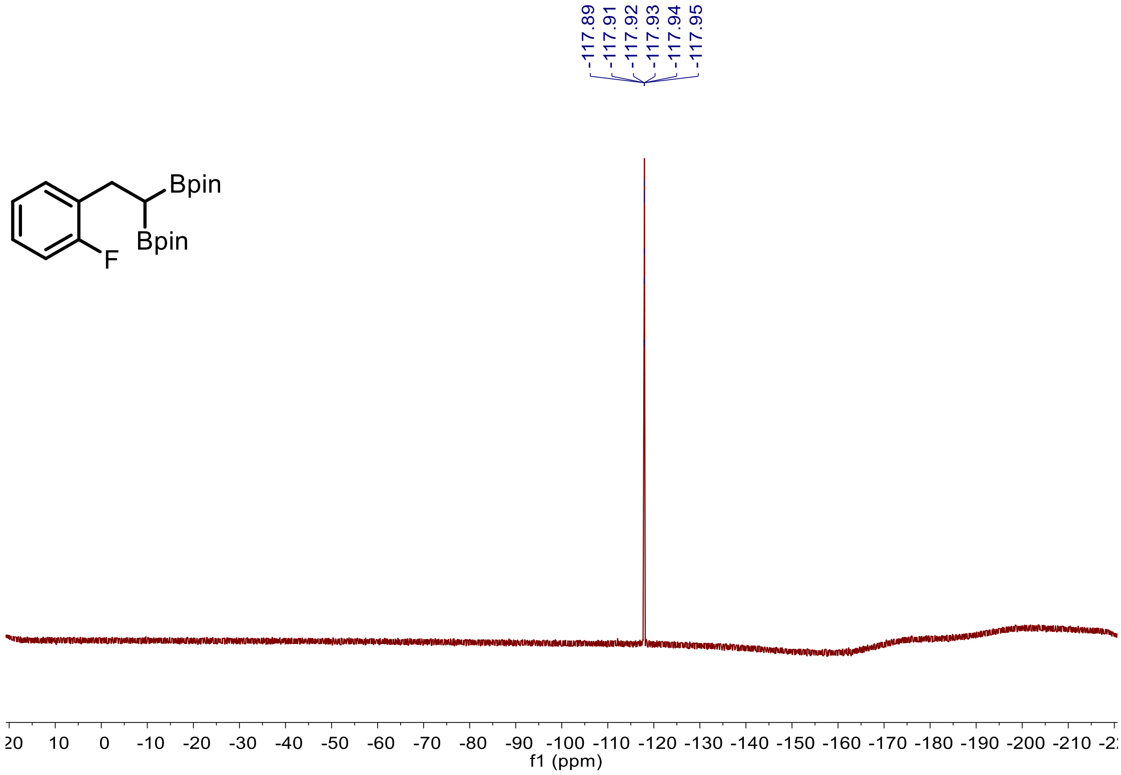

^{19}F NMR spectrum of **24** (Chloroform-*d*)

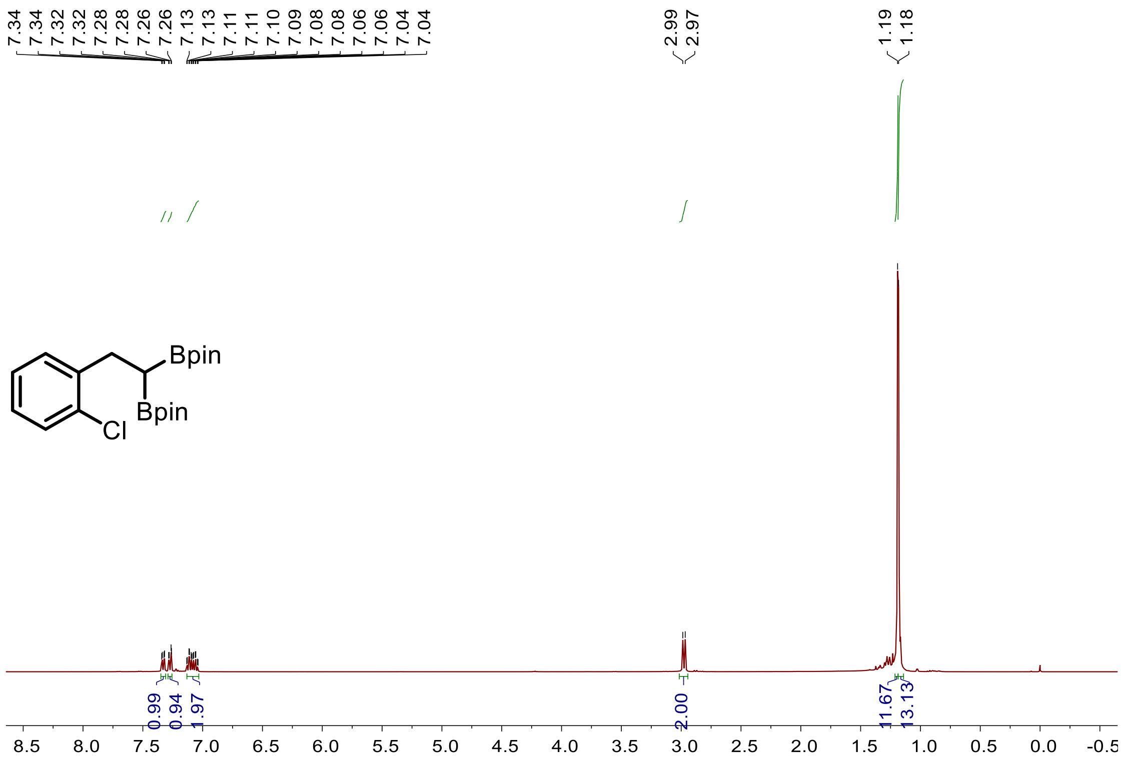

^{1}H NMR spectrum of **25** (Chloroform-*d*)

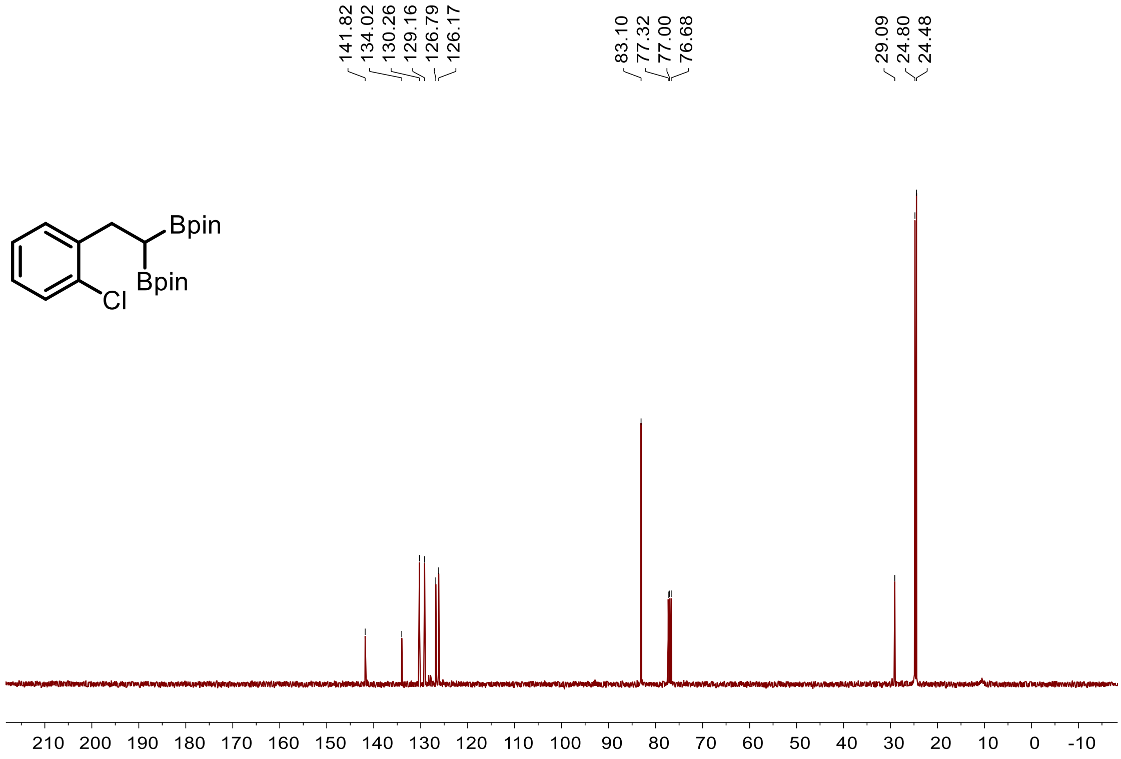

^{13}C NMR spectrum of **25** (Chloroform-*d*)

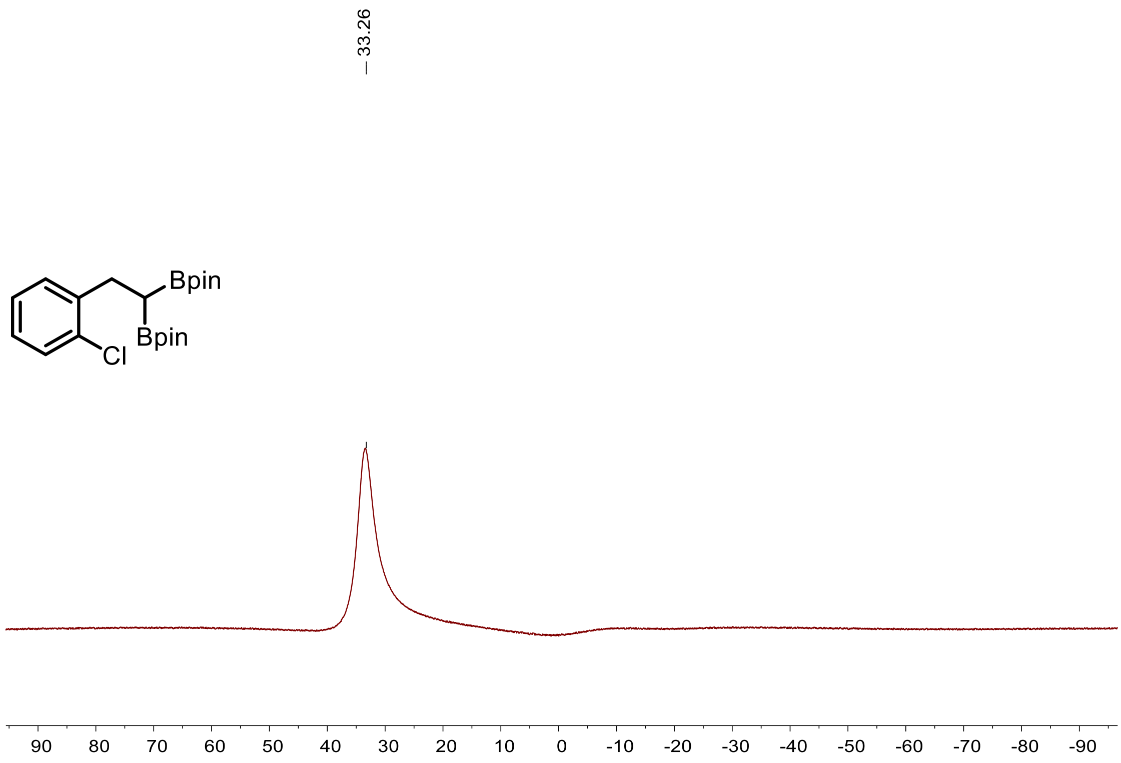

^{11}B NMR spectrum of **25** (Chloroform-*d*)

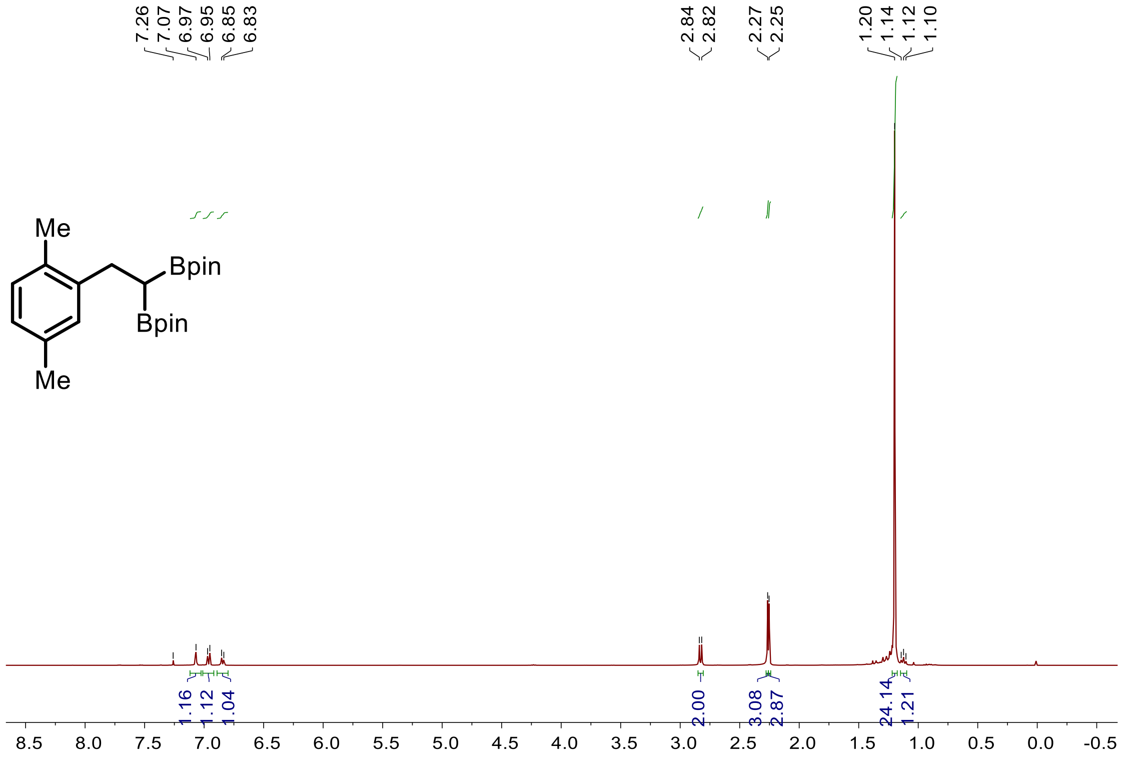

^{1}H NMR spectrum of **26** (Chloroform-*d*)

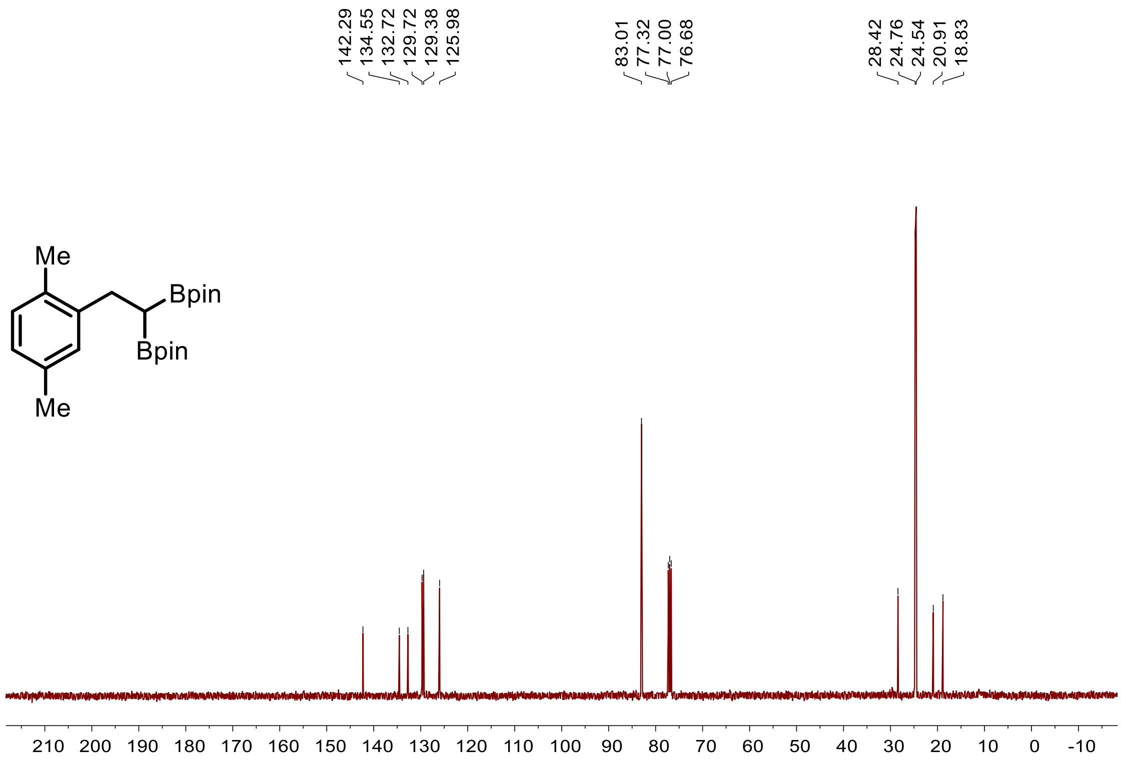

^{13}C NMR spectrum of **26** (Chloroform-*d*)

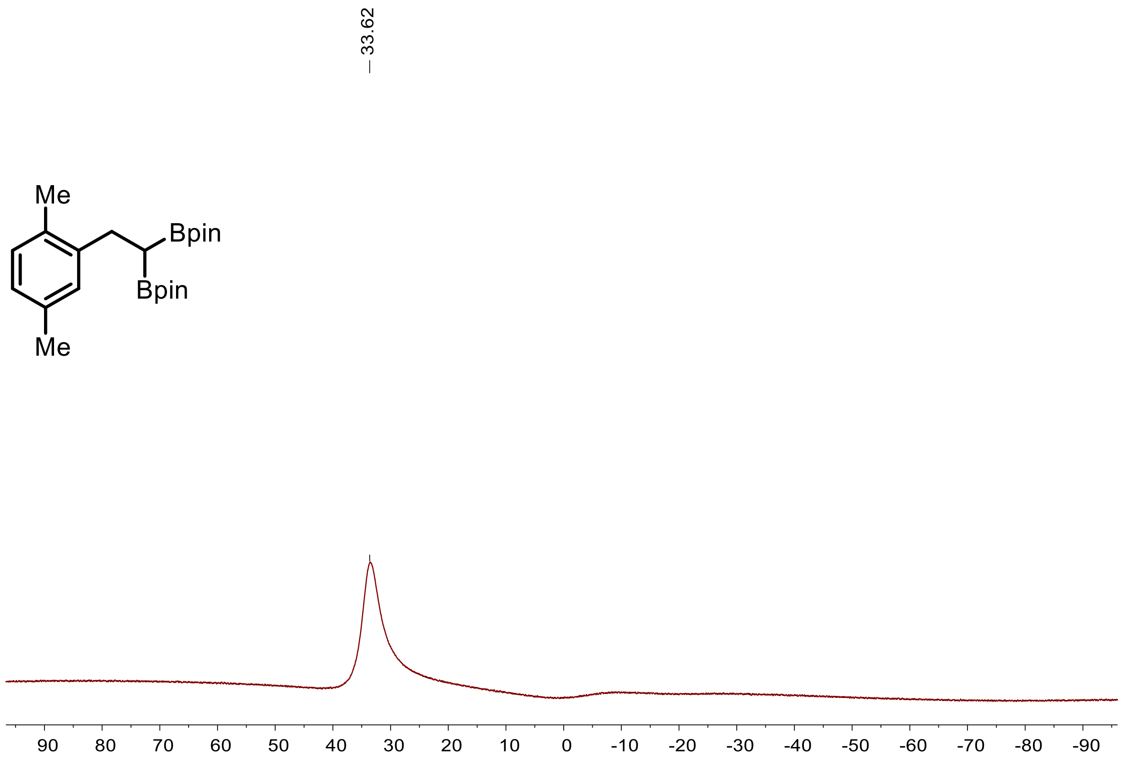

^{11}B NMR spectrum of **26** (Chloroform-*d*)

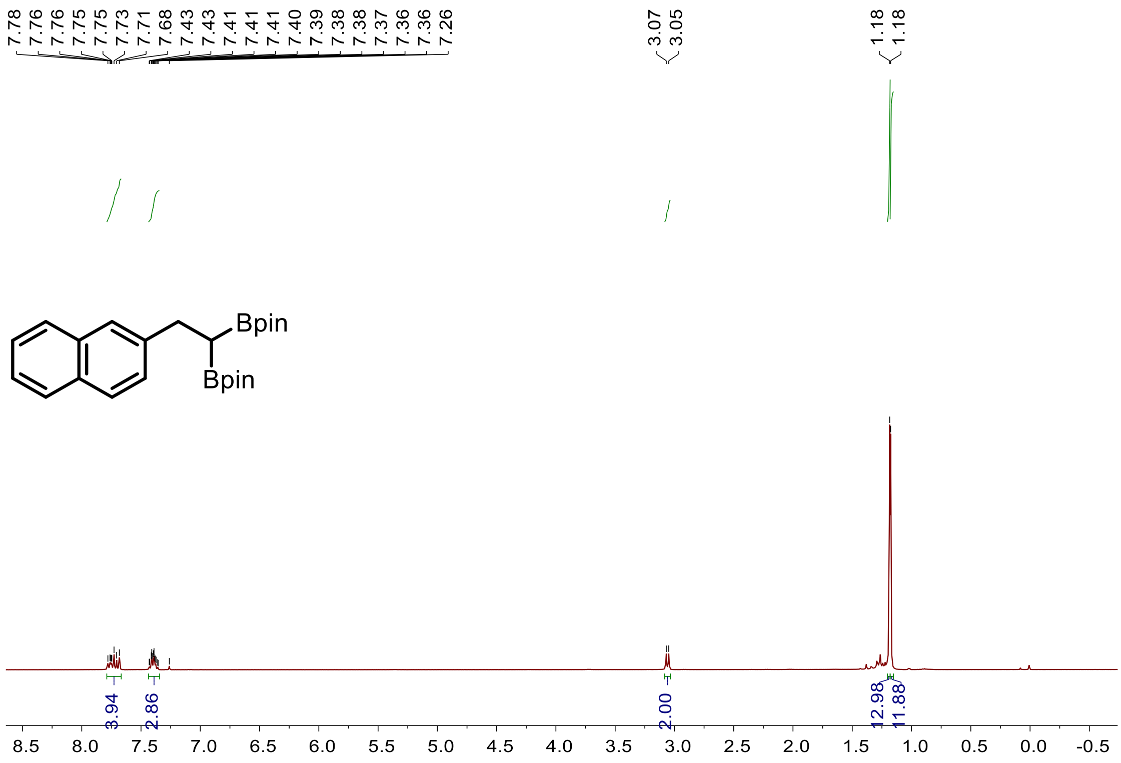

^{1}H NMR spectrum of **28** (Chloroform-*d*)

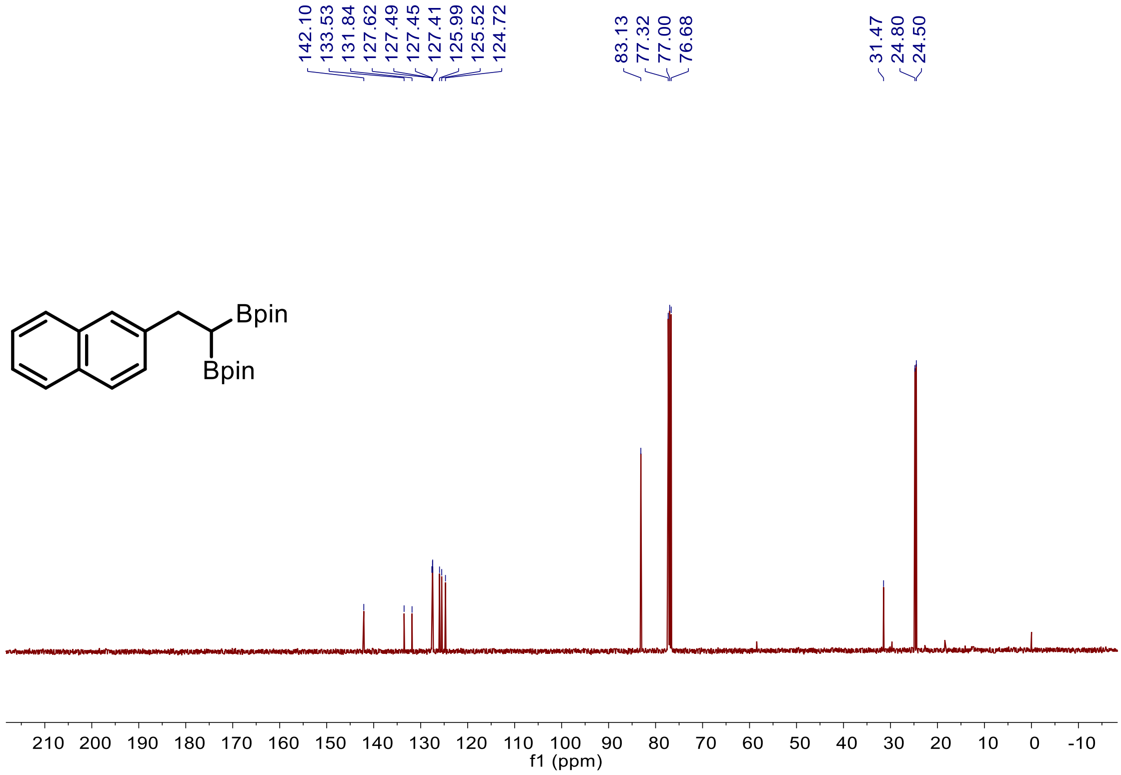

^{13}C NMR spectrum of **28** (Chloroform-*d*)

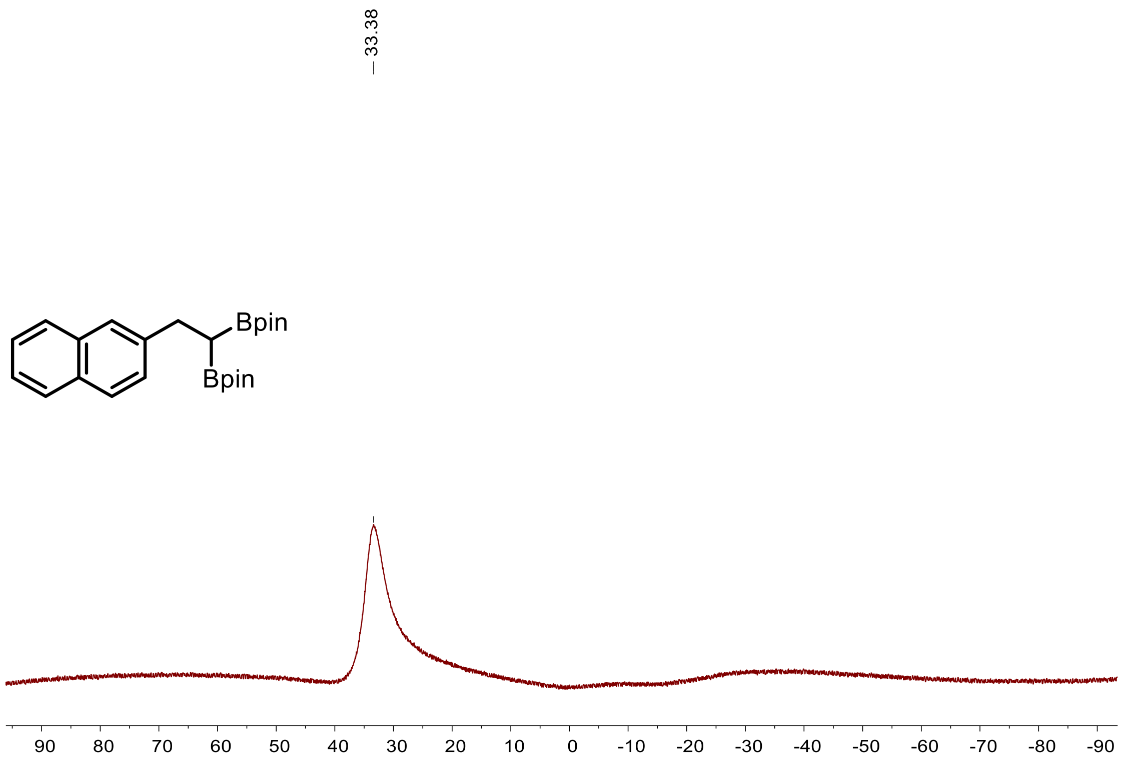

^{11}B NMR spectrum of **28** (Chloroform-*d*)

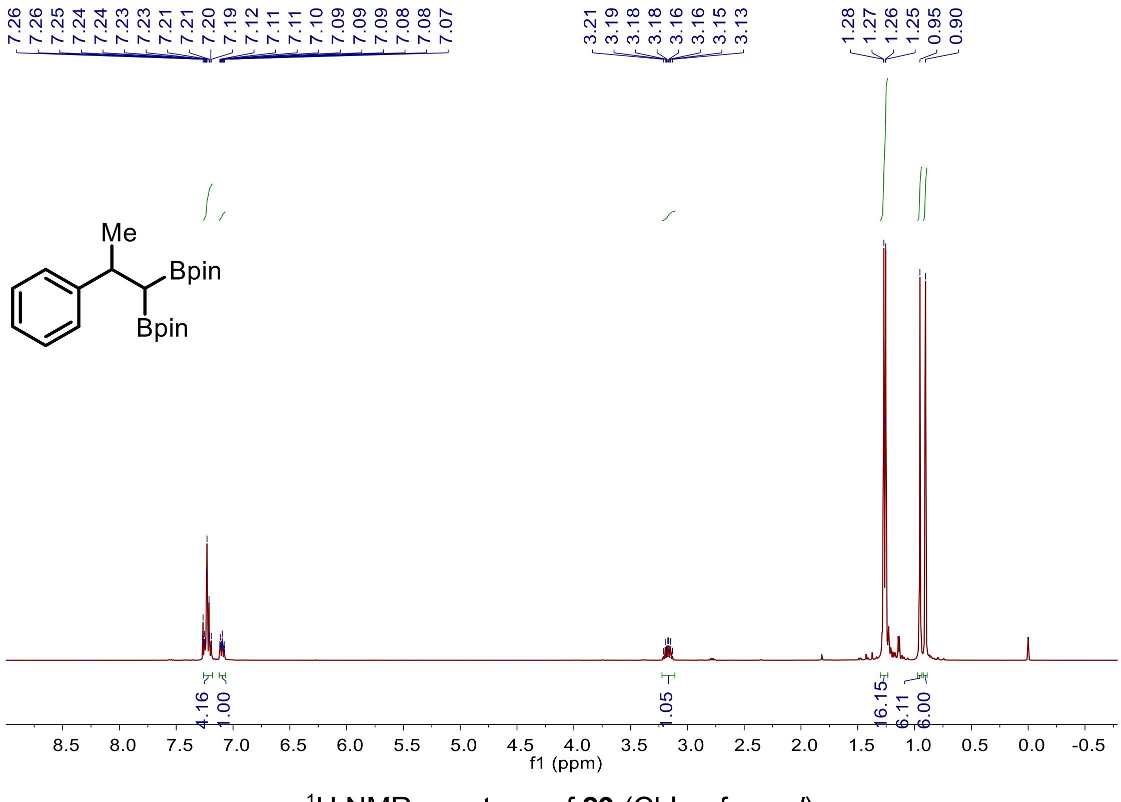

^{1}H NMR spectrum of **29** (Chloroform-*d*)

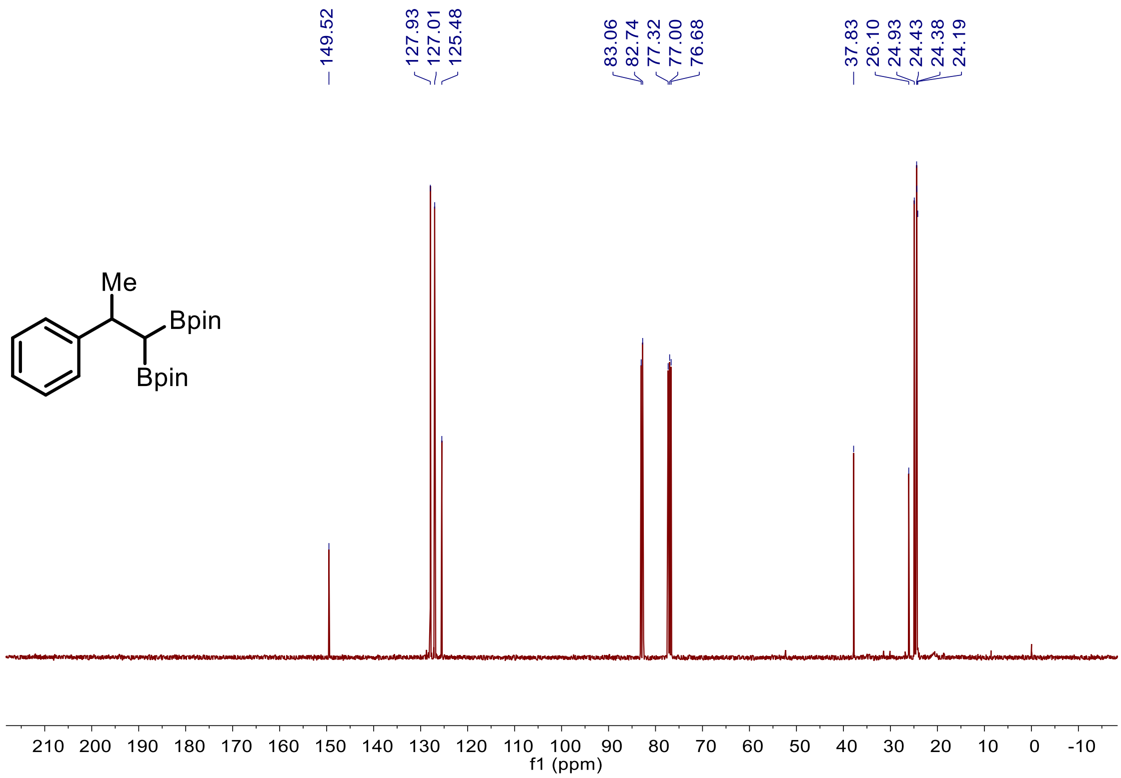

^{13}C NMR spectrum of **29** (Chloroform-*d*)

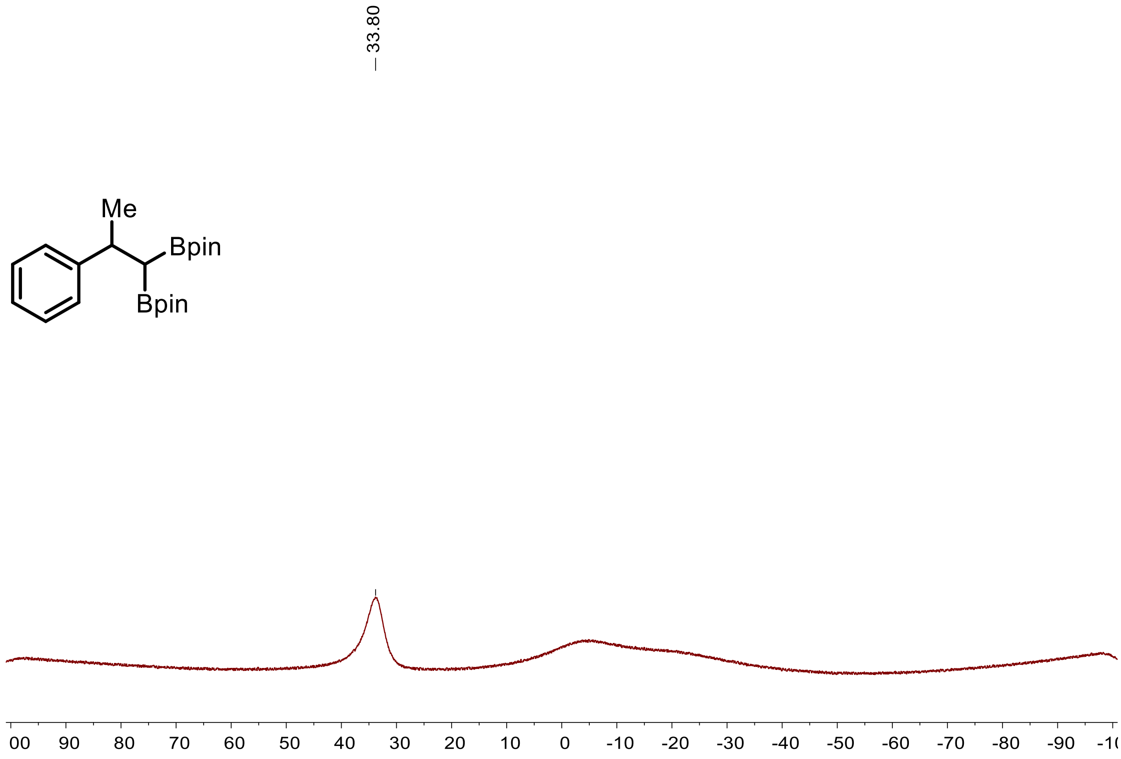

^{11}B NMR spectrum of **29** (Chloroform-*d*)

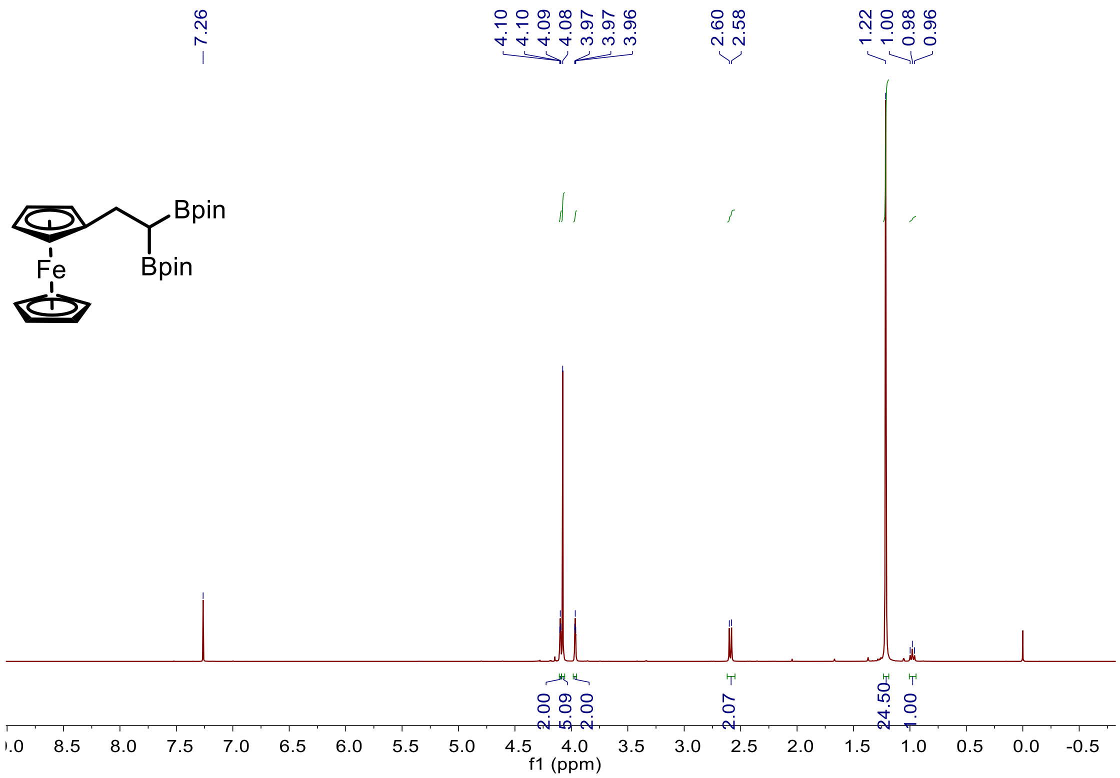

^{1}H NMR spectrum of **30** (Chloroform-*d*)

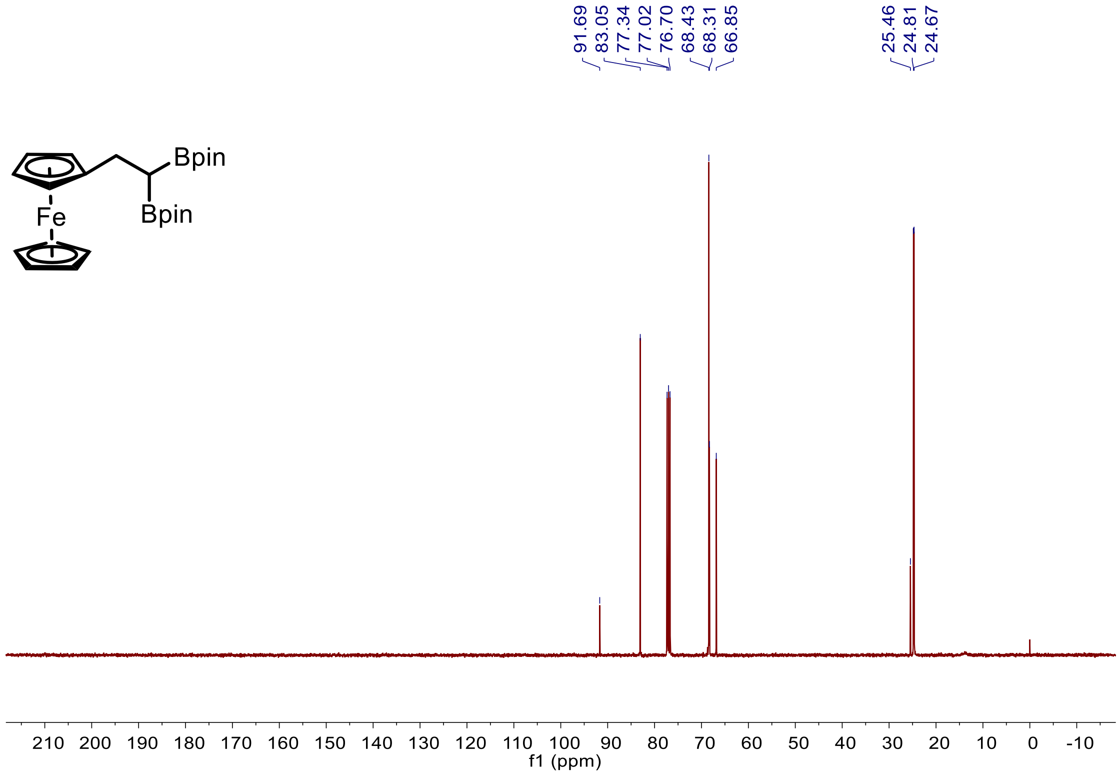

^{13}C NMR spectrum of **30** (Chloroform-*d*)

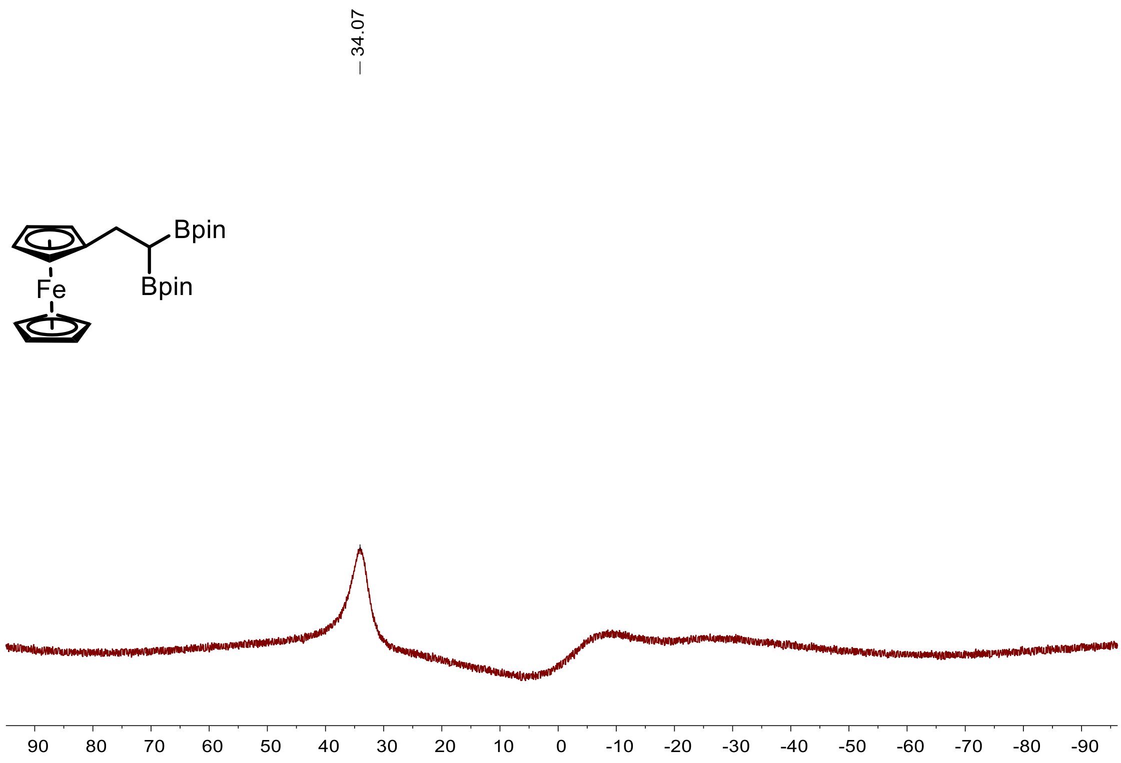

^{11}B NMR spectrum of **30** (Chloroform-*d*)

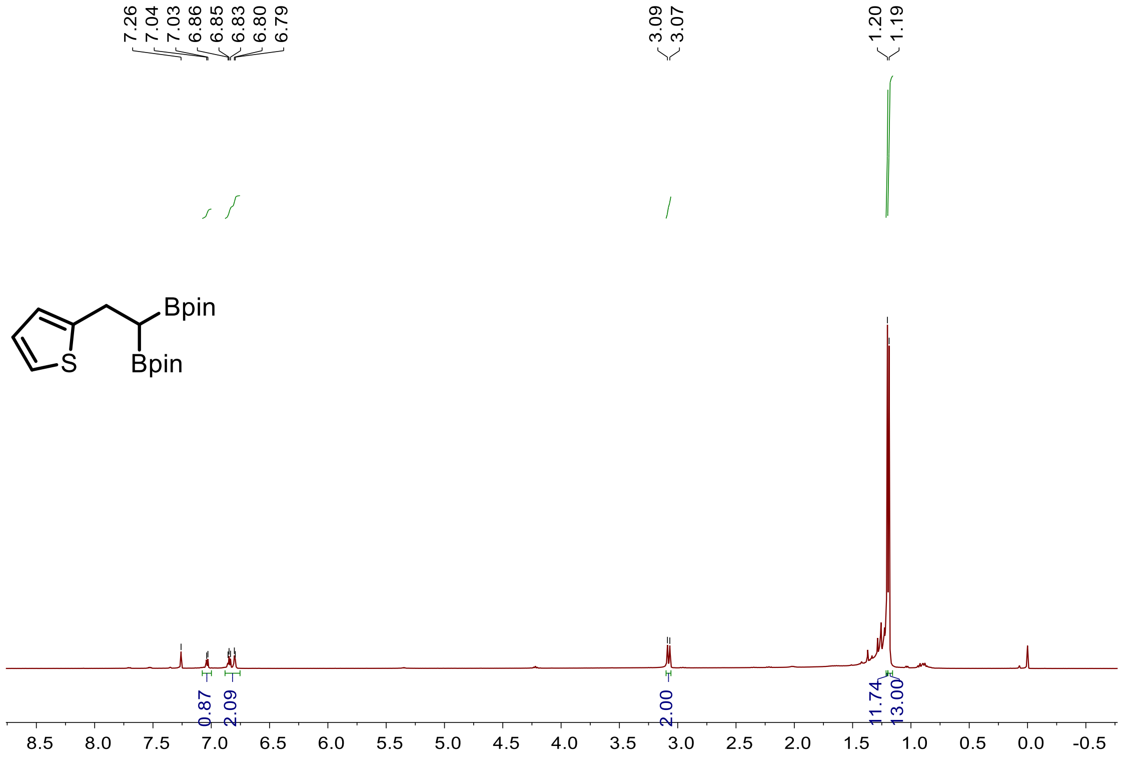

^{1}H NMR spectrum of **31** (Chloroform-*d*)

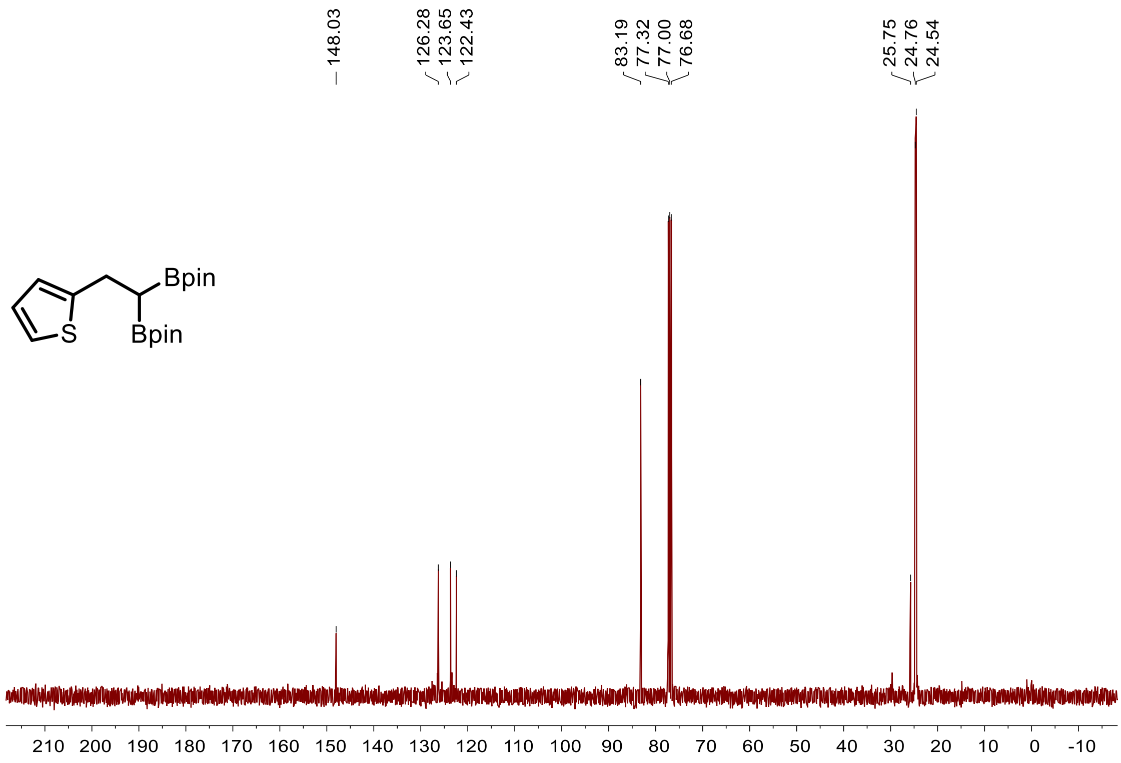

^{13}C NMR spectrum of **31** (Chloroform-*d*)

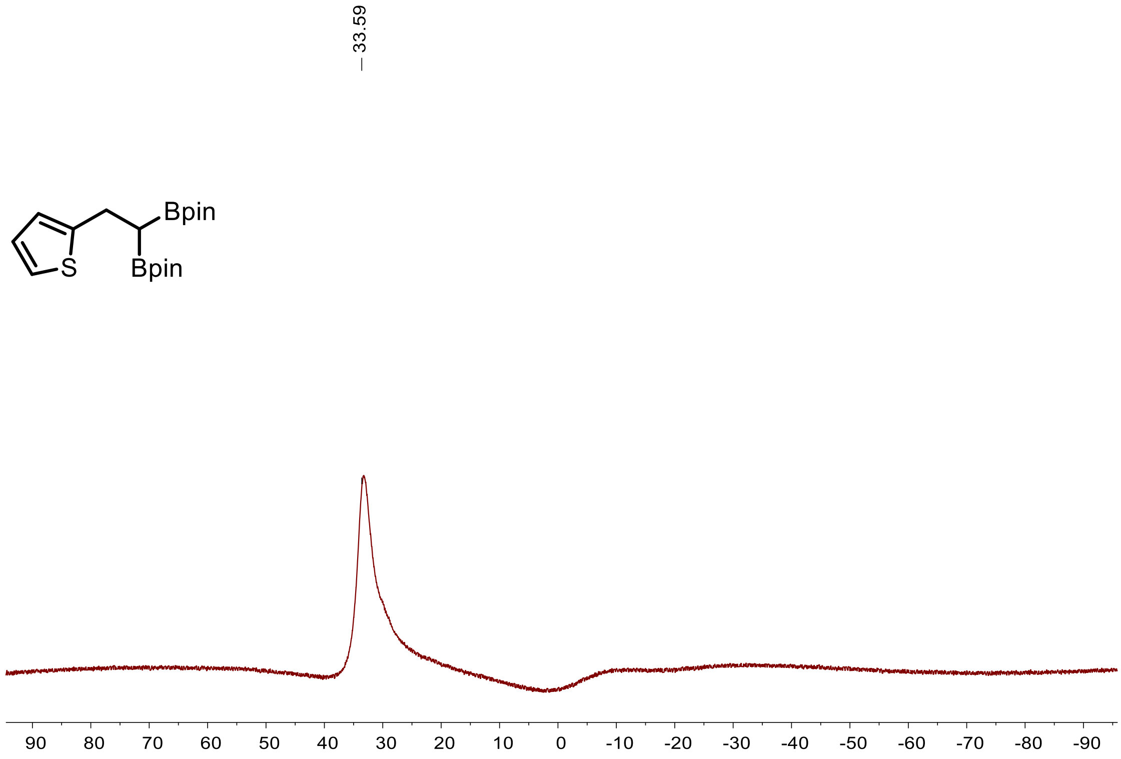

^{11}B NMR spectrum of **31** (Chloroform-*d*)

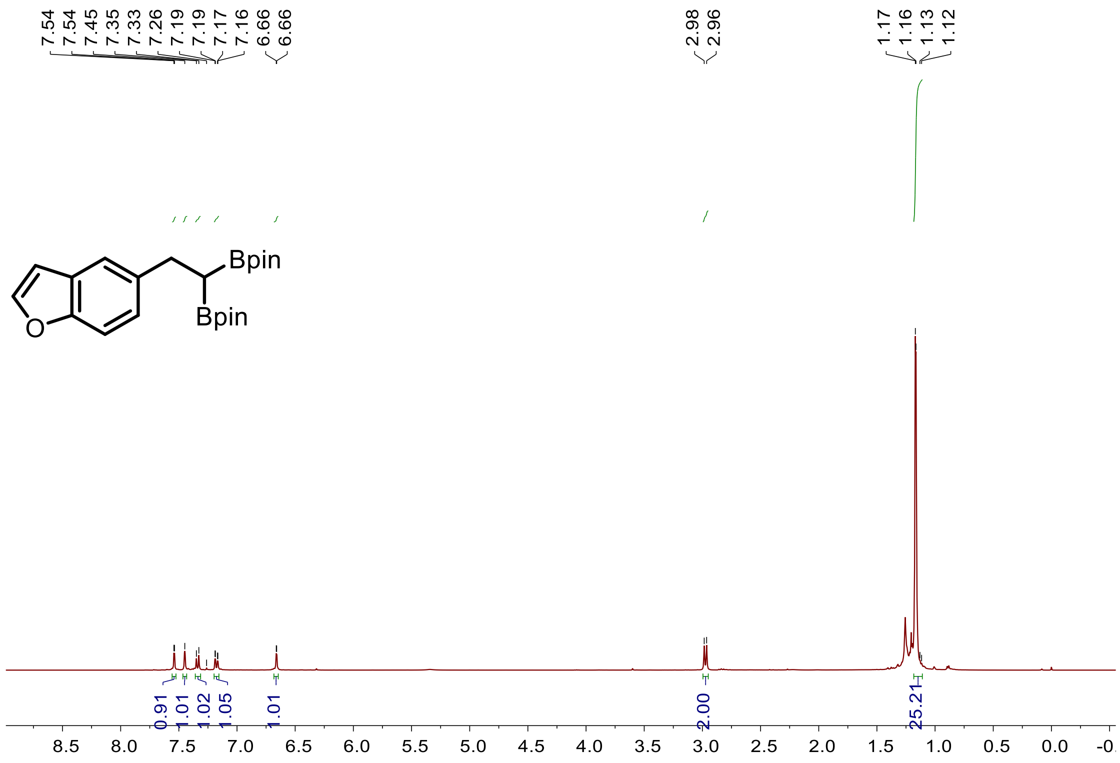

^{1}H NMR spectrum of **32** (Chloroform-*d*)

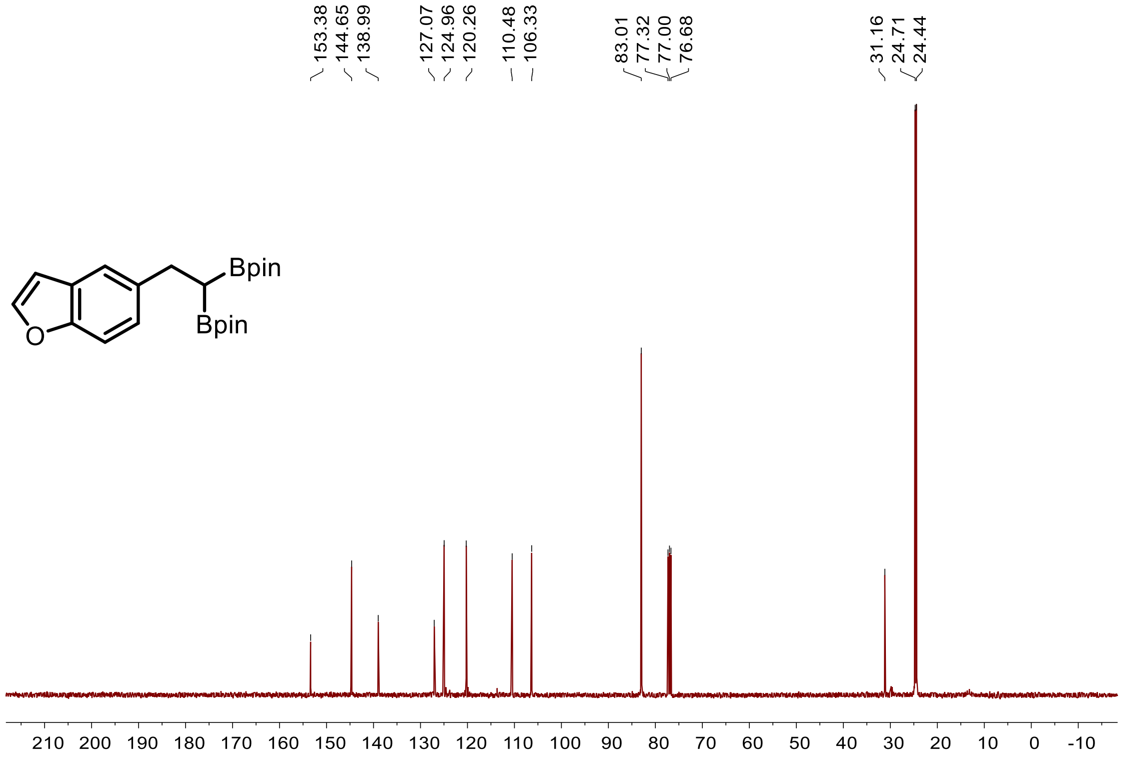

^{13}C NMR spectrum of **32** (Chloroform-*d*)

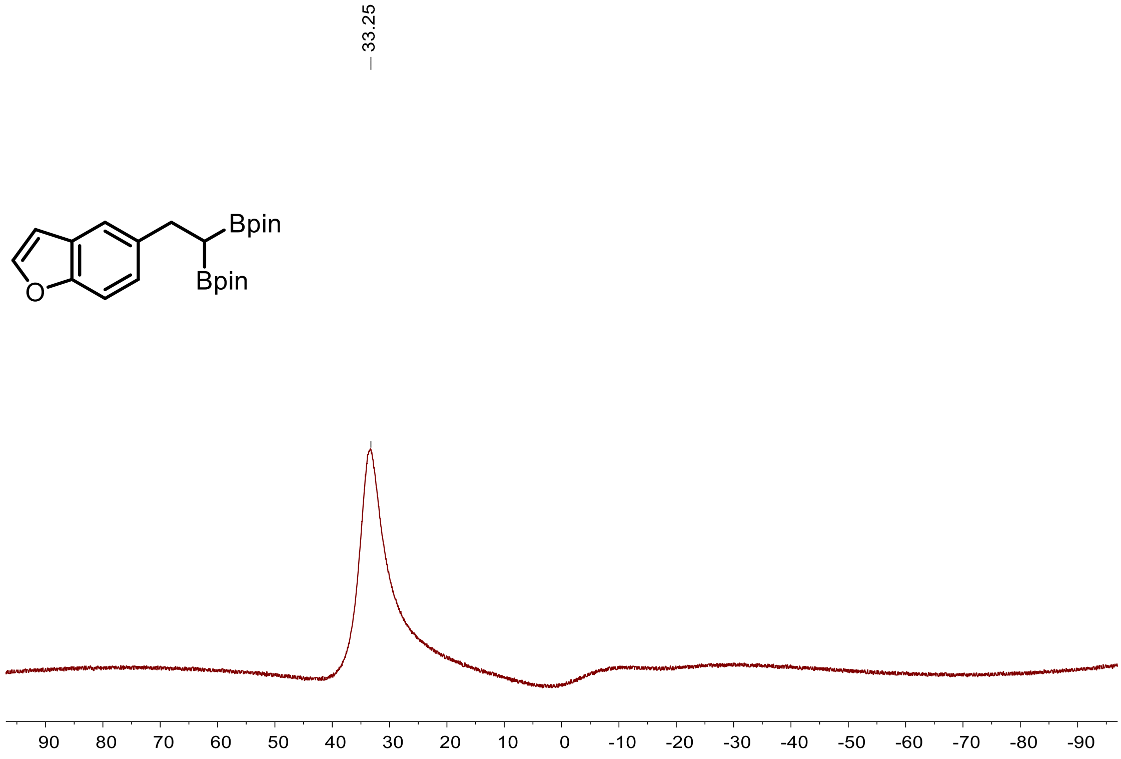

^{11}B NMR spectrum of **32** (Chloroform-*d*)

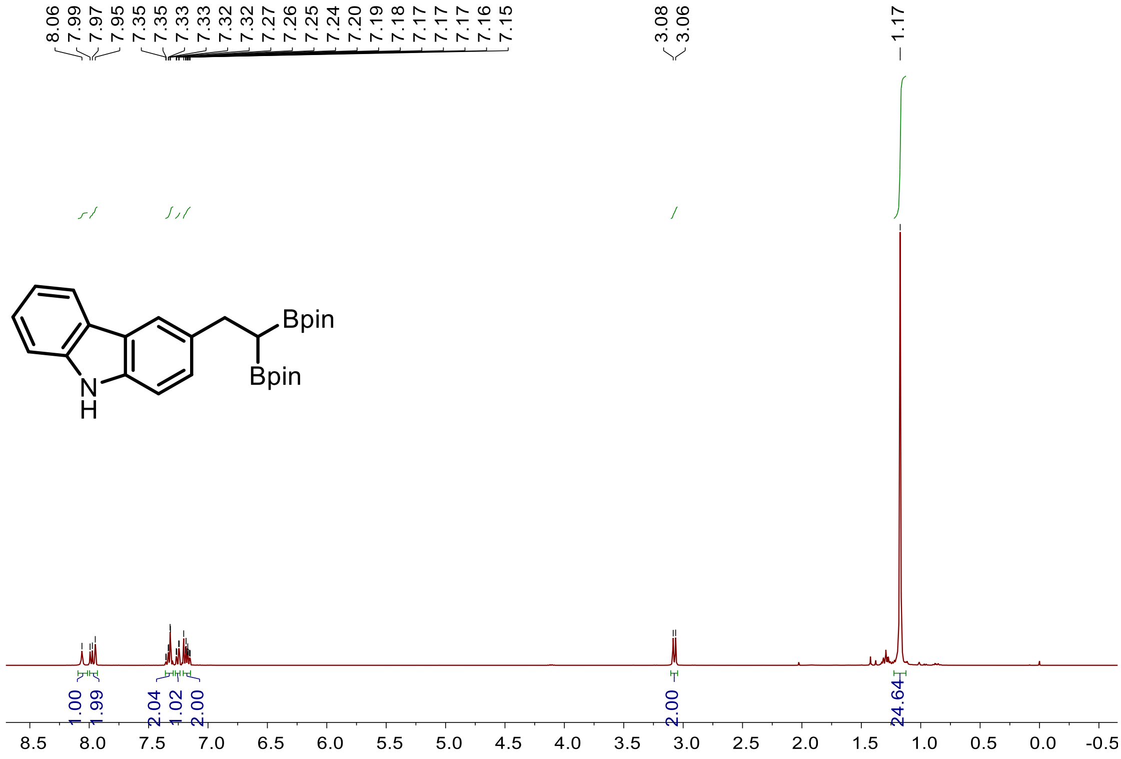

^{1}H NMR spectrum of **33** (Chloroform-*d*)

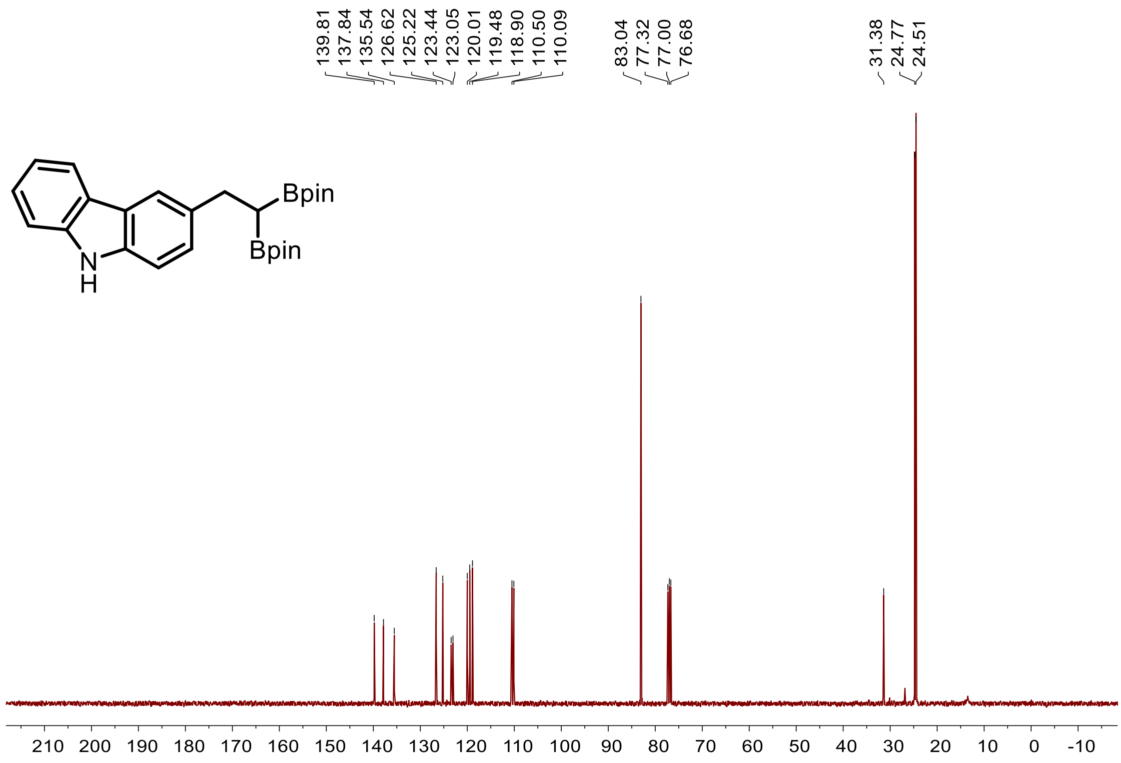

^{13}C NMR spectrum of **33** (Chloroform-*d*)

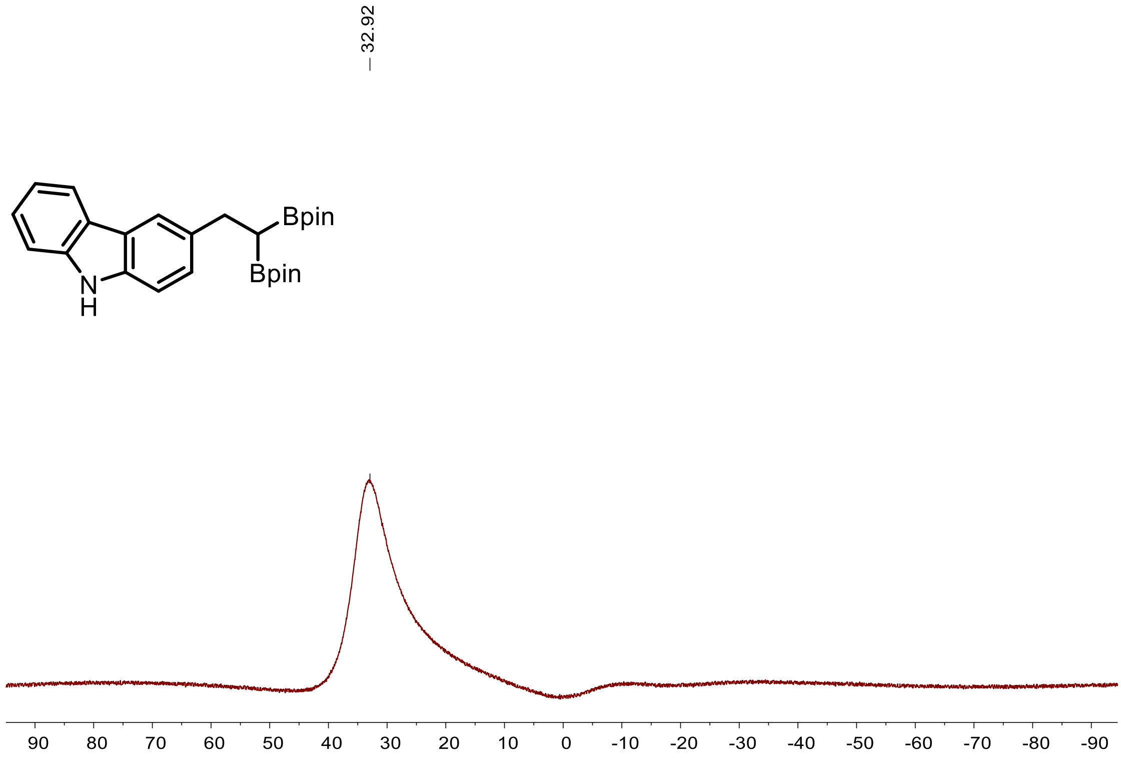

^{11}B NMR spectrum of **33** (Chloroform-*d*)

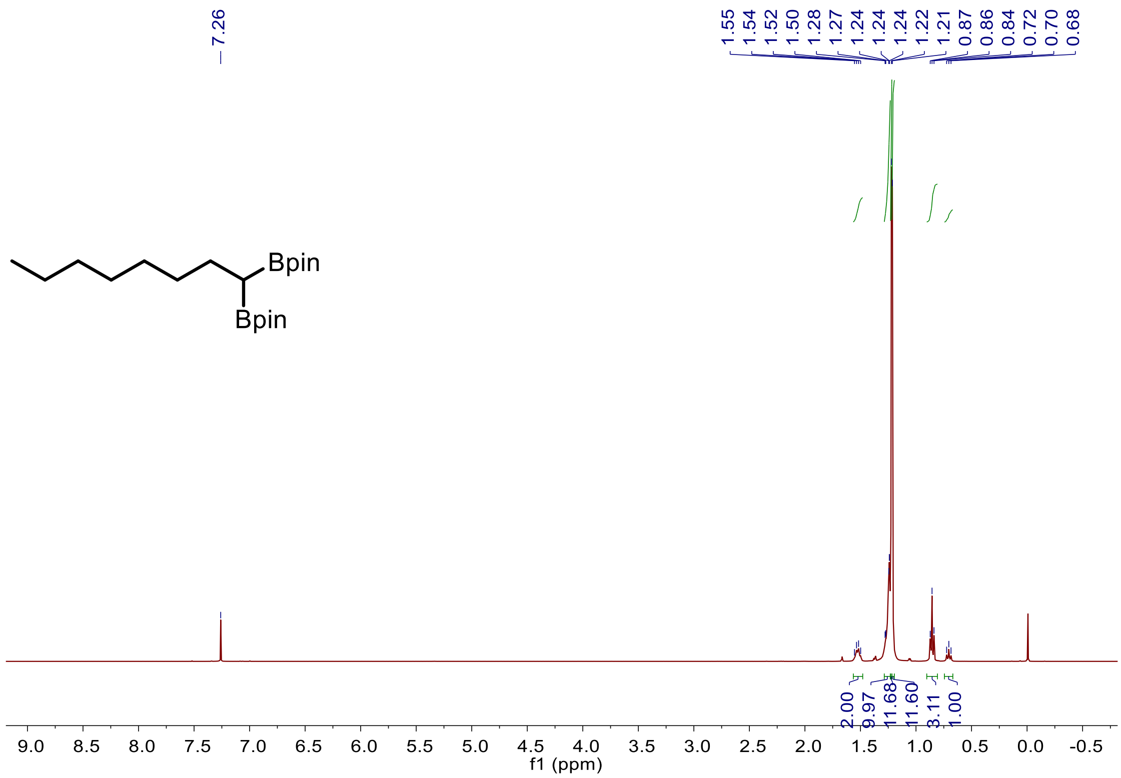

^{1}H NMR spectrum of **34** (Chloroform-*d*)

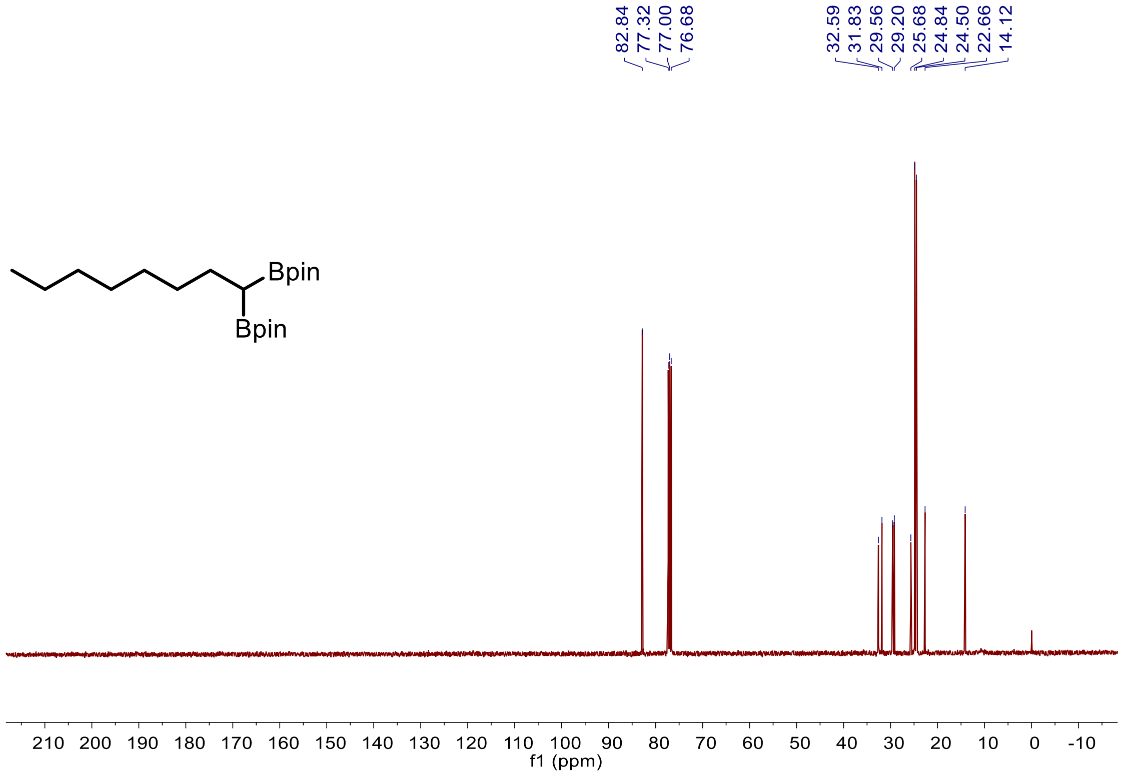

^{13}C NMR spectrum of **34** (Chloroform-*d*)

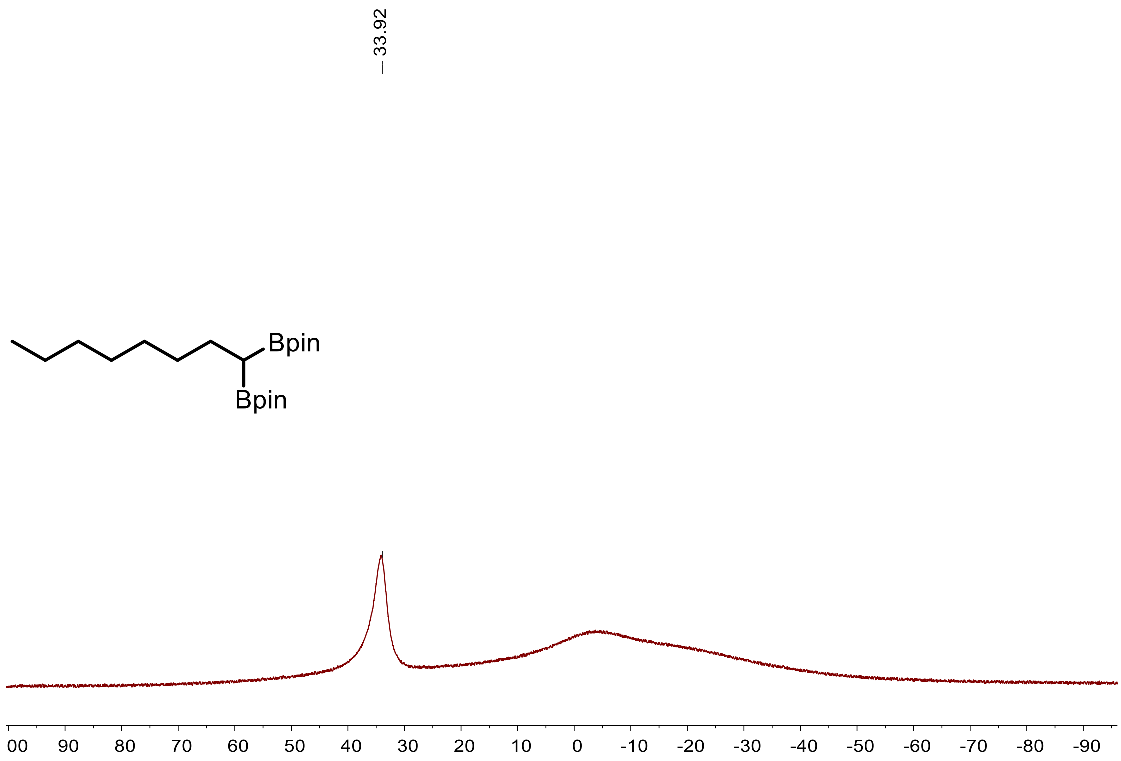

^{11}B NMR spectrum of **34** (Chloroform-*d*)

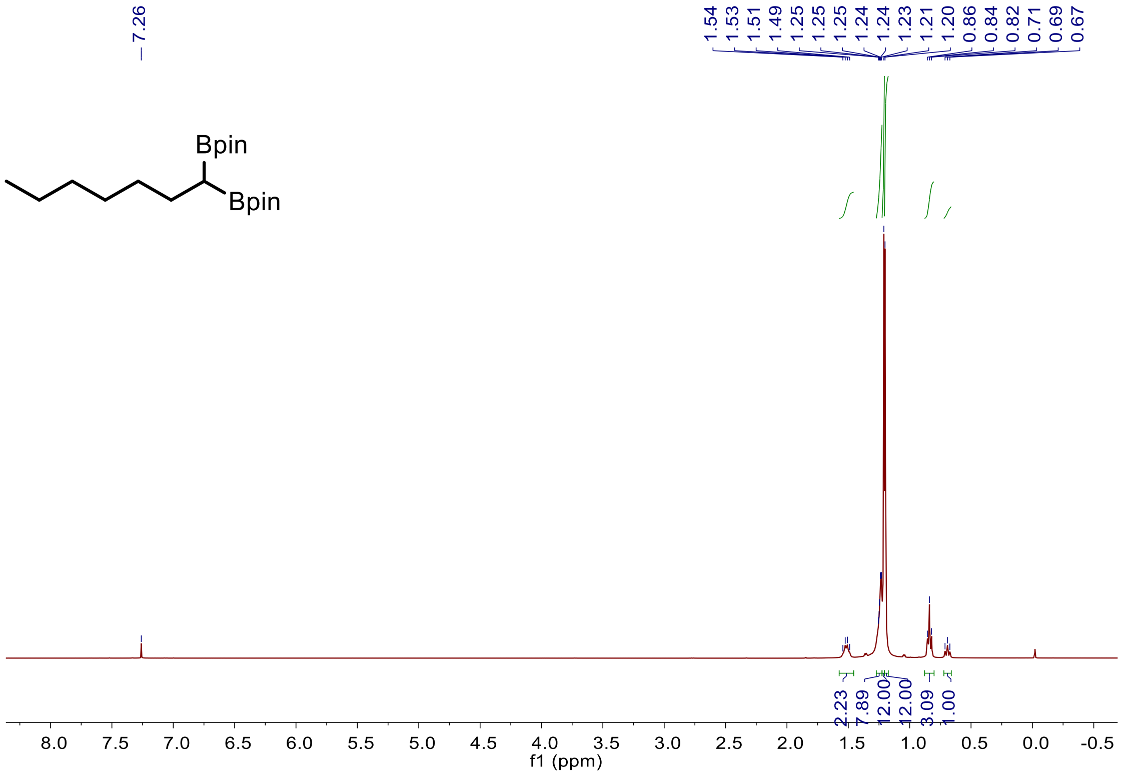

^{1}H NMR spectrum of **35** (Chloroform-*d*)

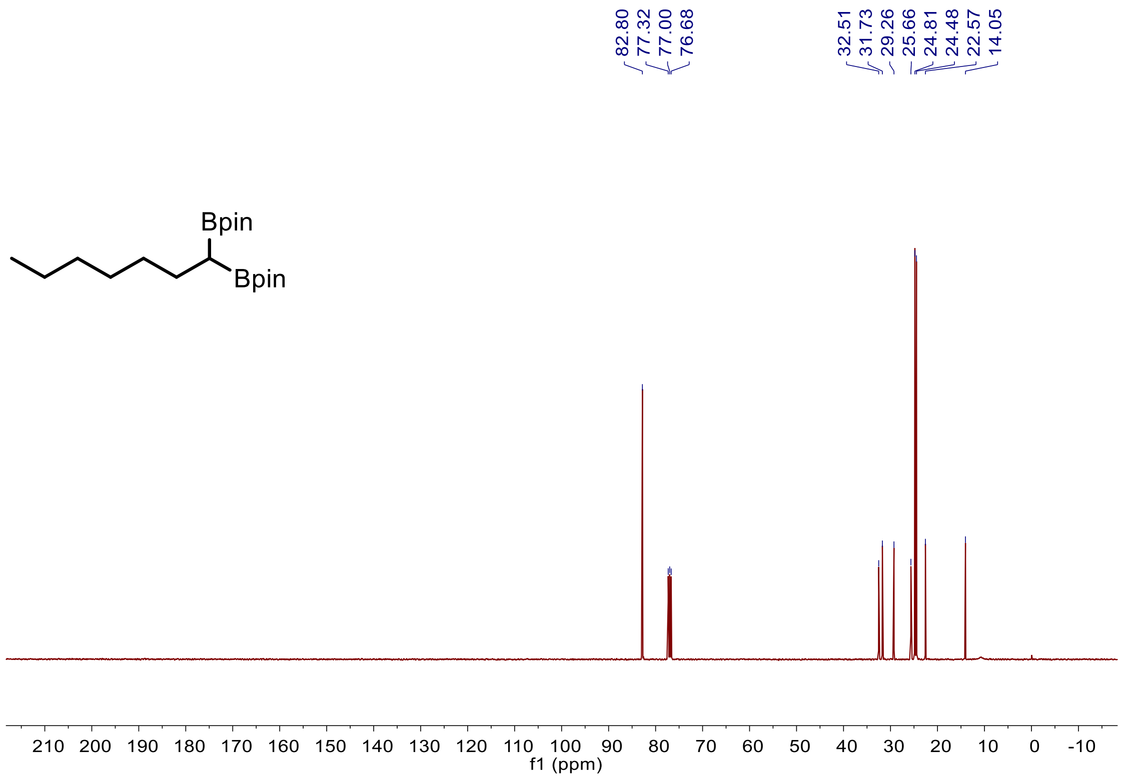

^{13}C NMR spectrum of **35** (Chloroform-*d*)

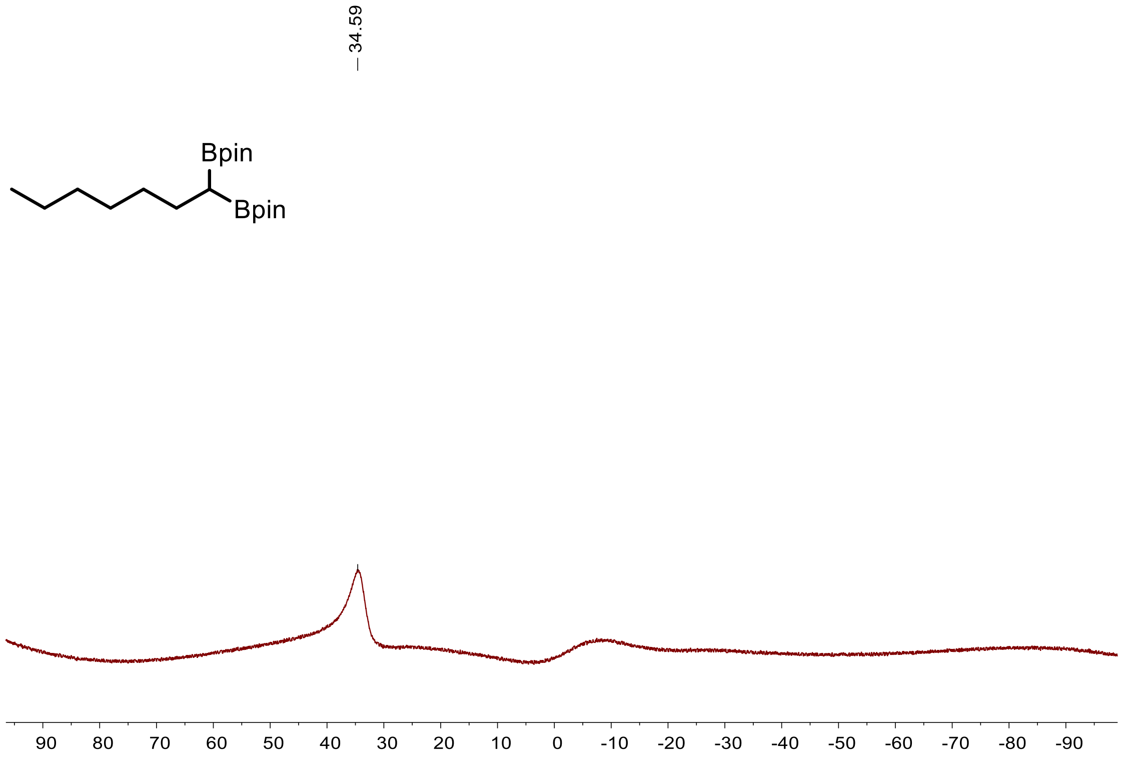

^{11}B NMR spectrum of **35** (Chloroform-*d*)

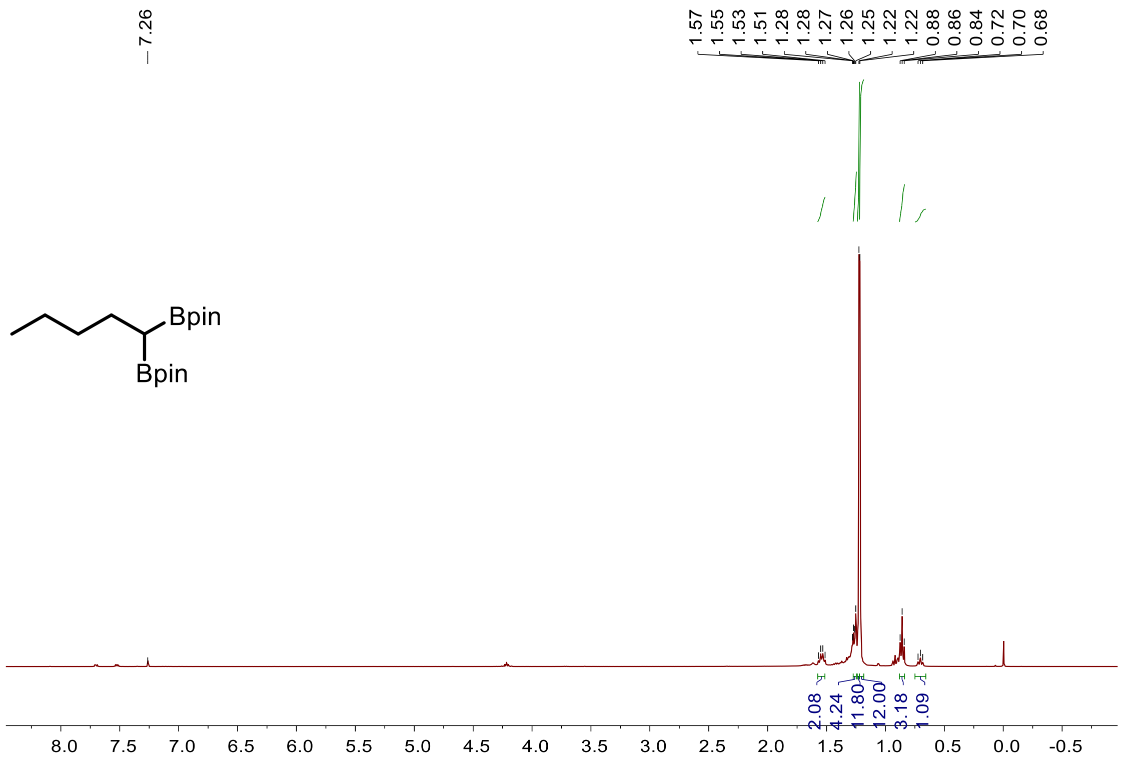

^{1}H NMR spectrum of **36** (Chloroform-*d*)

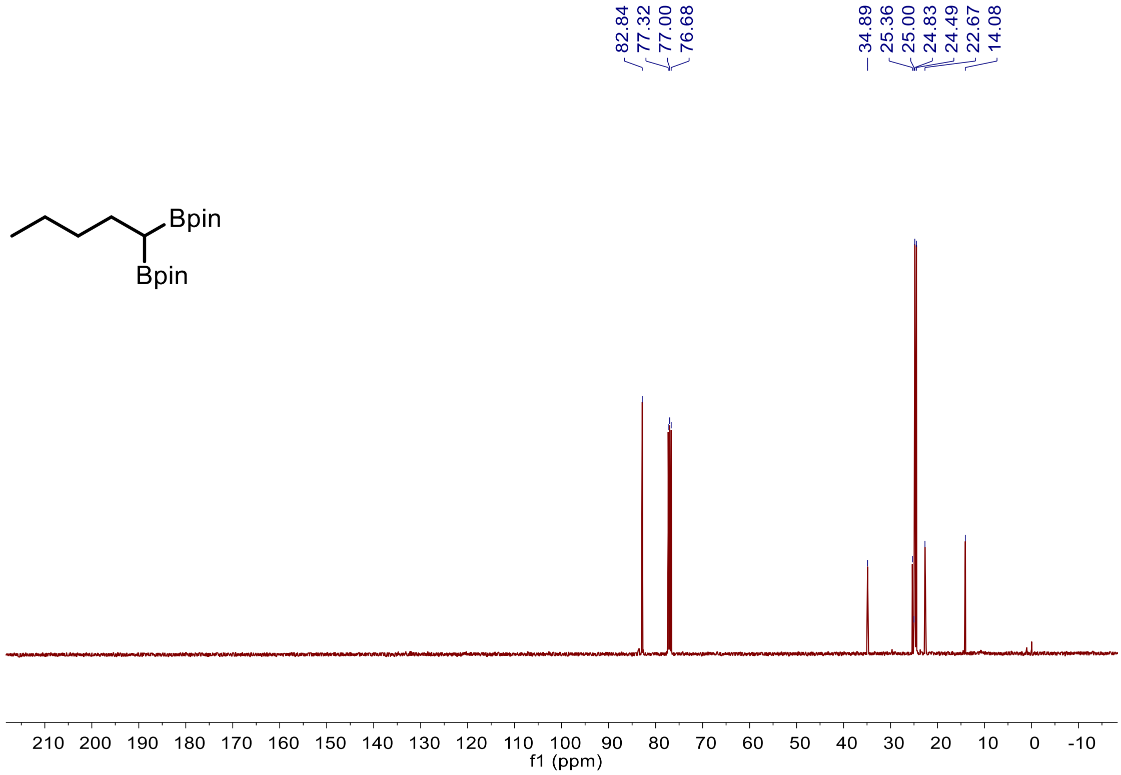

^{13}C NMR spectrum of **36** (Chloroform-*d*)

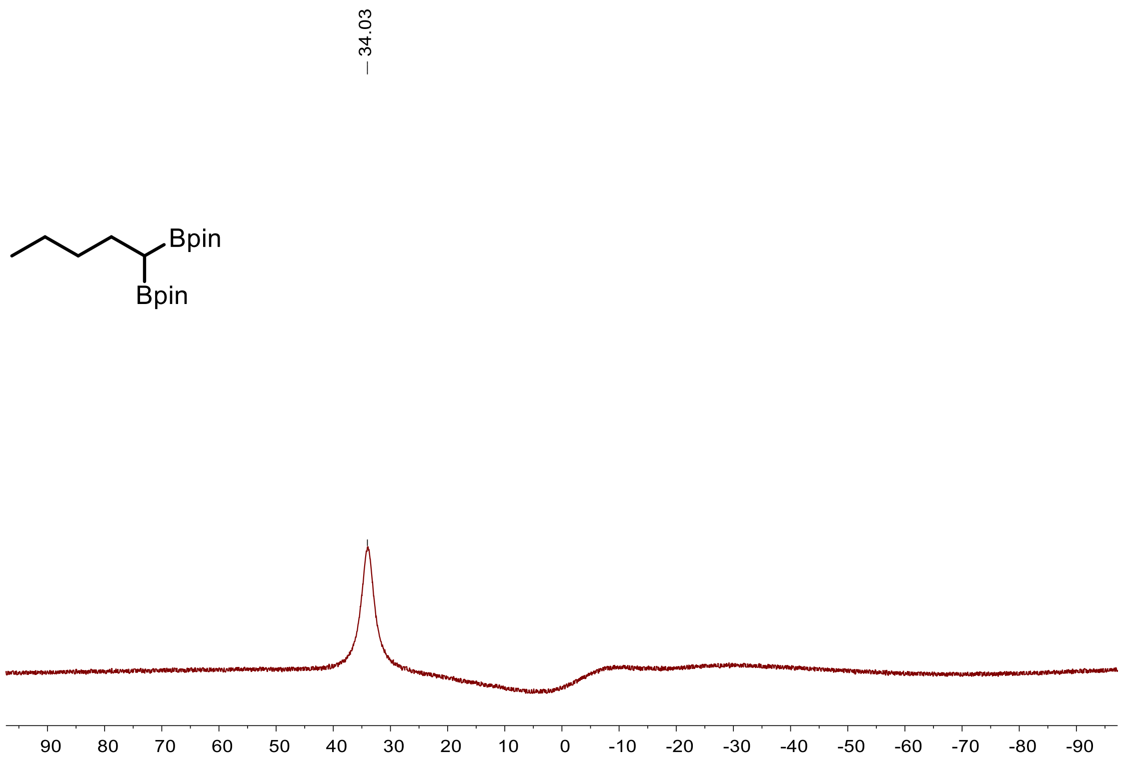

^{11}B NMR spectrum of **36** (Chloroform-*d*)

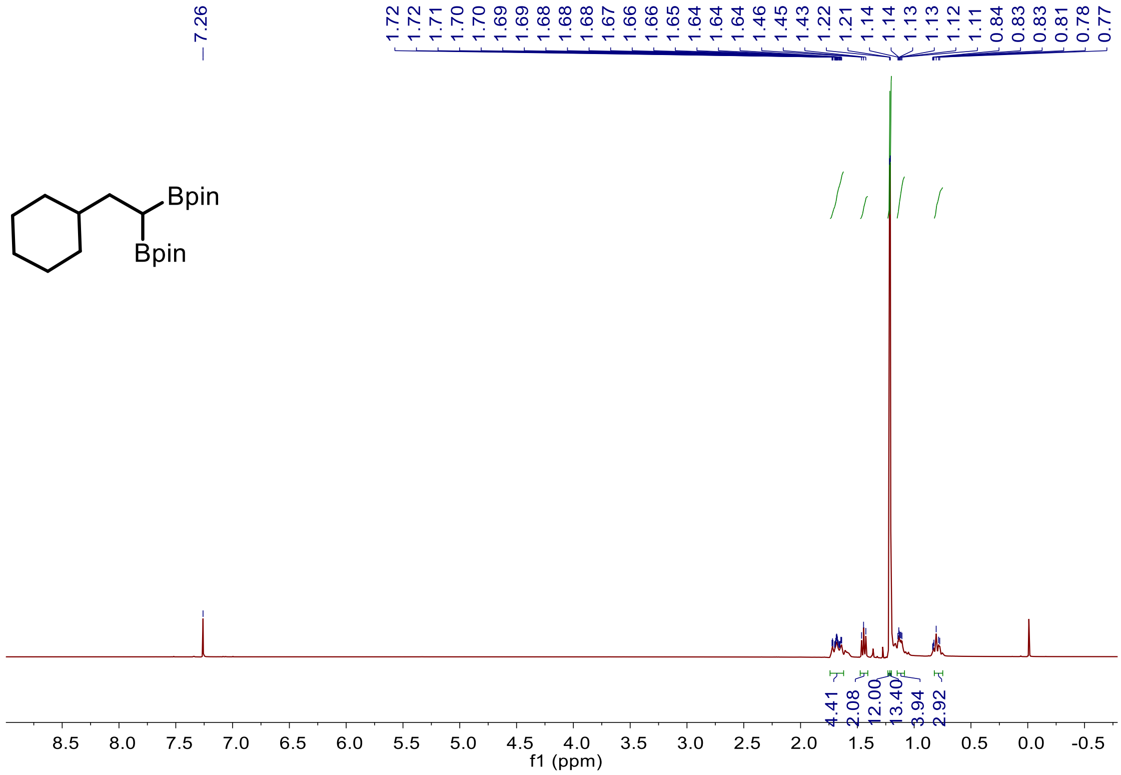

^{1}H NMR spectrum of **37** (Chloroform-*d*)

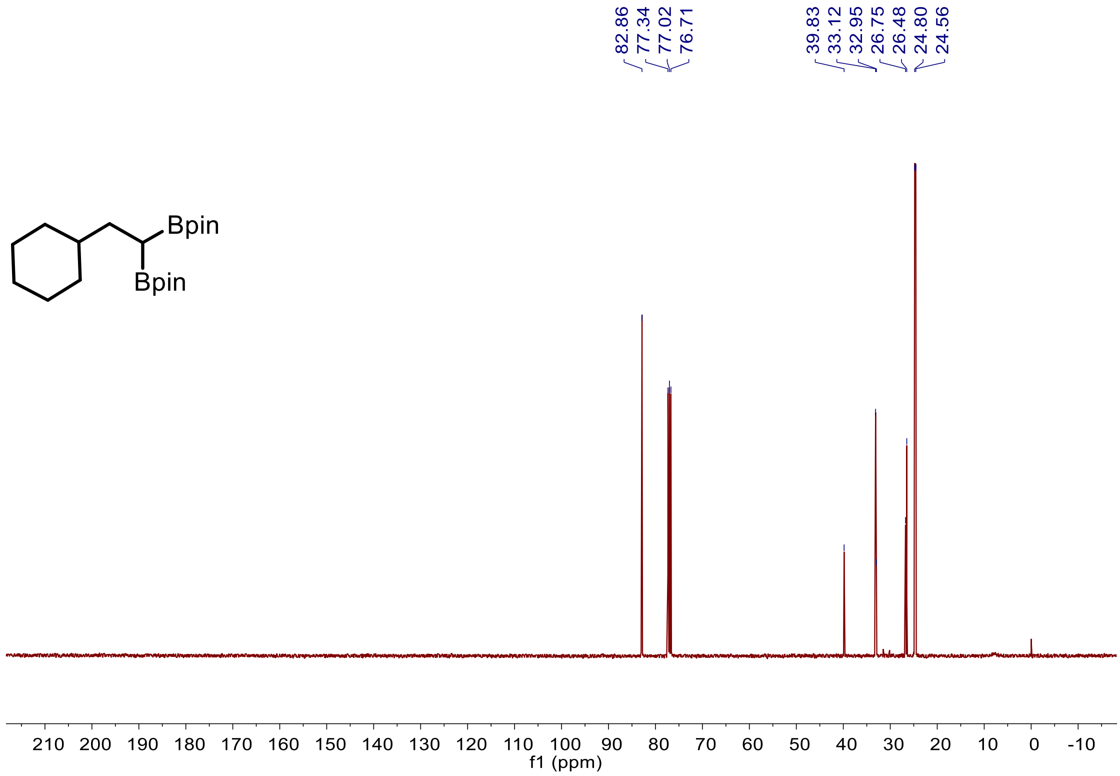

^{13}C NMR spectrum of **37** (Chloroform-*d*)

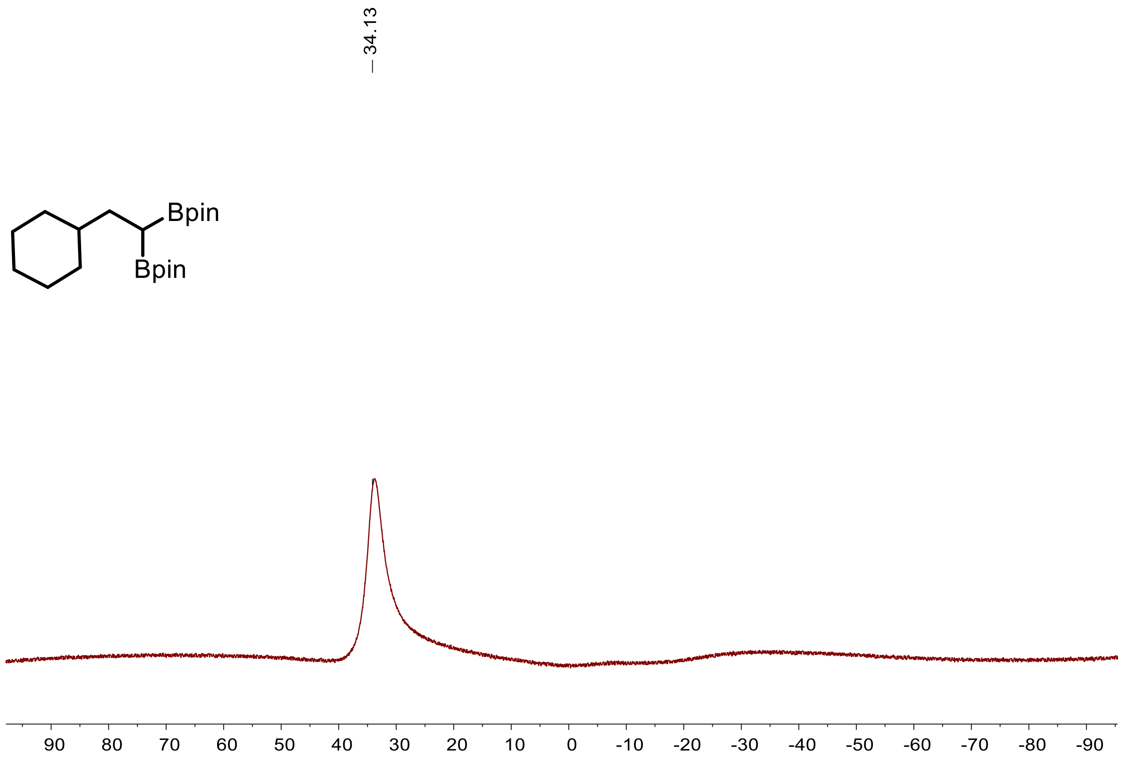

^{11}B NMR spectrum of **37** (Chloroform-*d*)

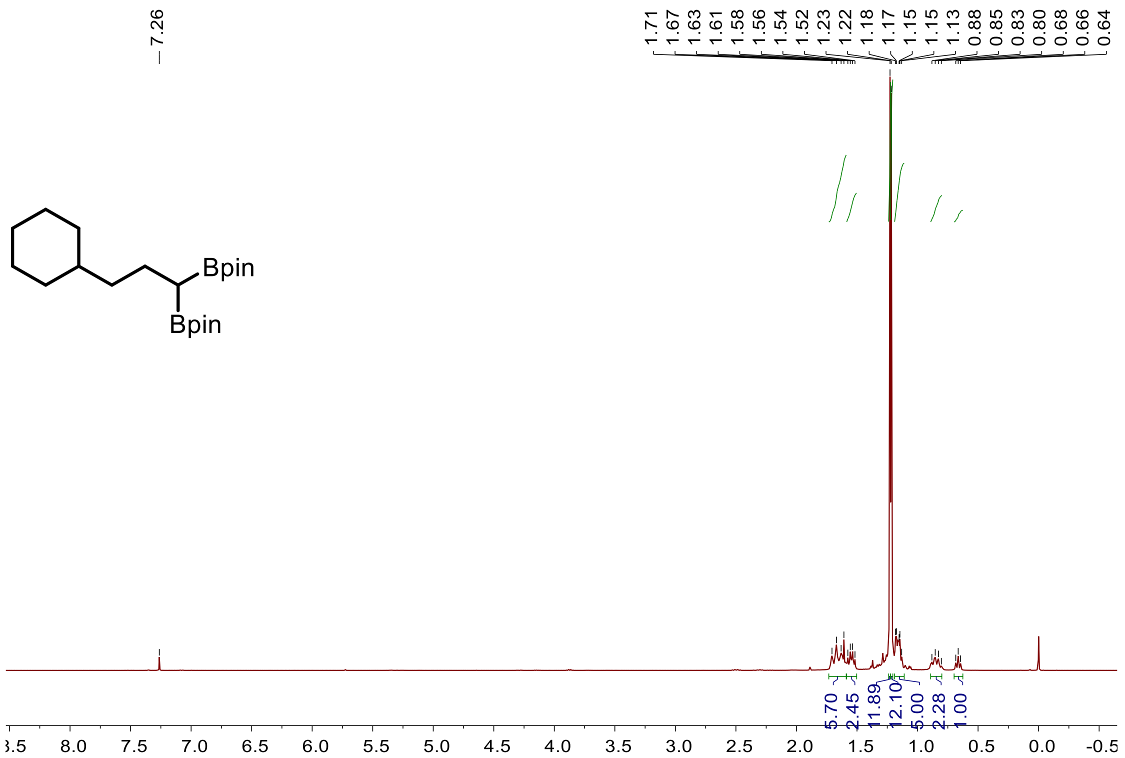

^{1}H NMR spectrum of **38** (Chloroform-*d*)

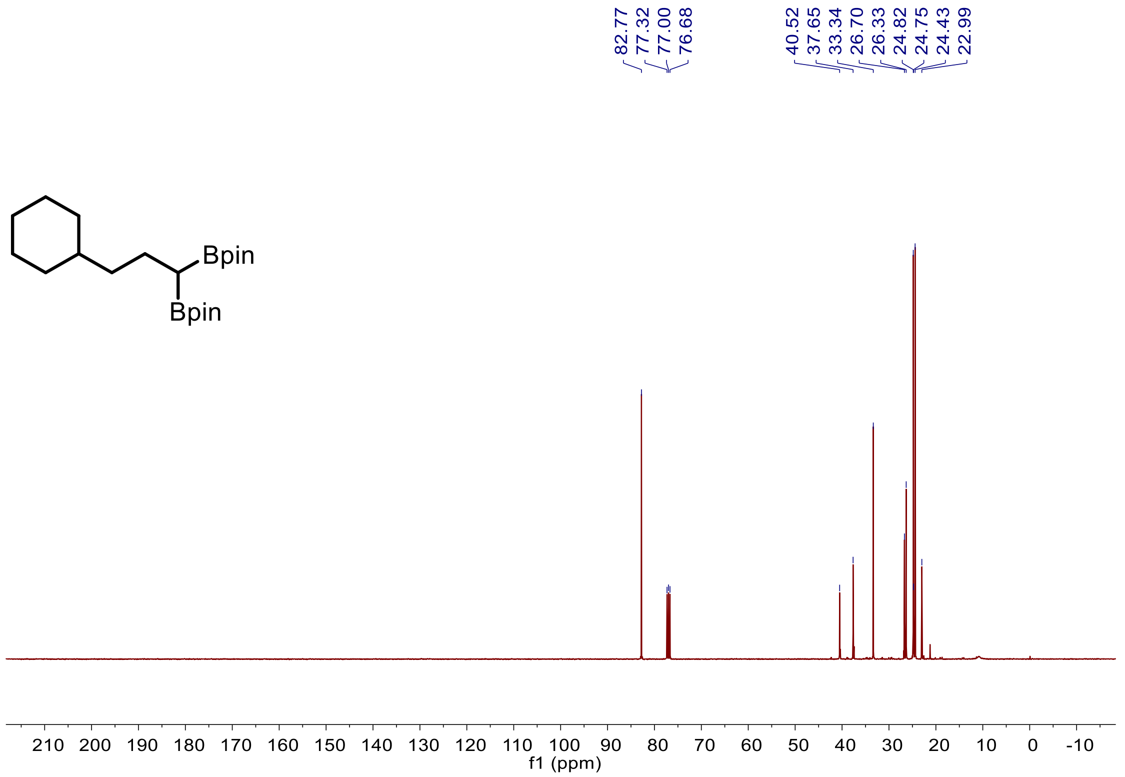

^{13}C NMR spectrum of **38** (Chloroform-*d*)

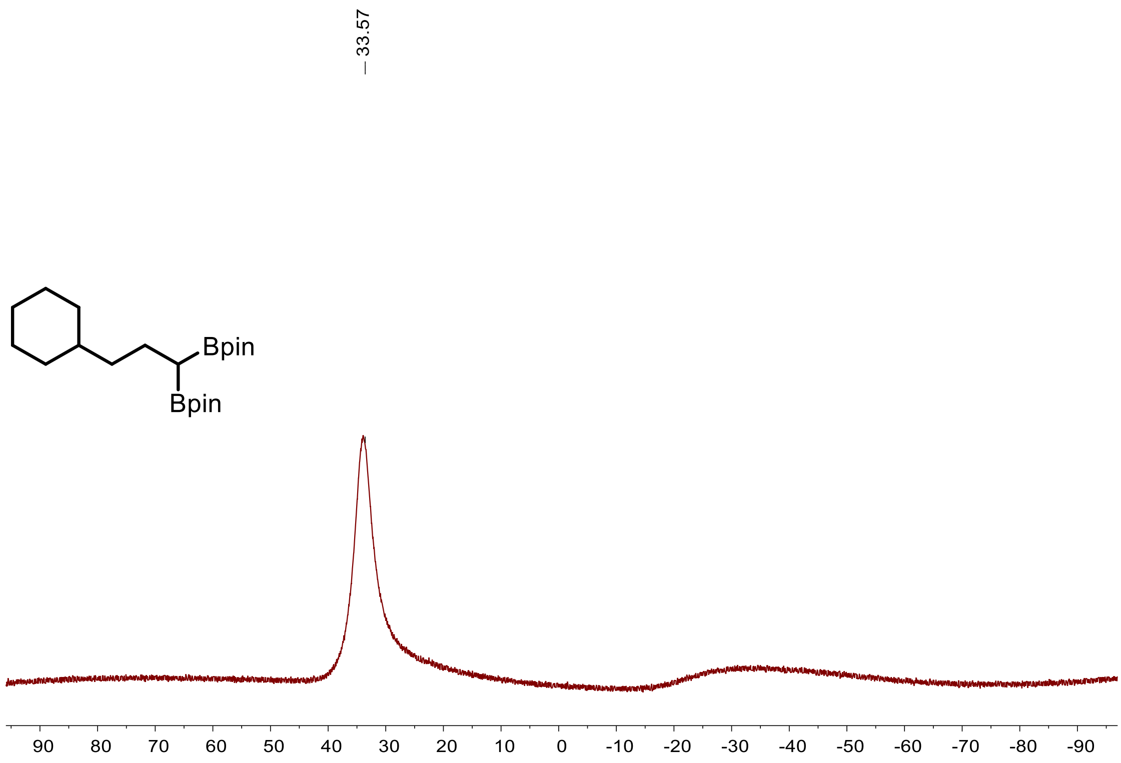

^{11}B NMR spectrum of **38** (Chloroform-*d*)

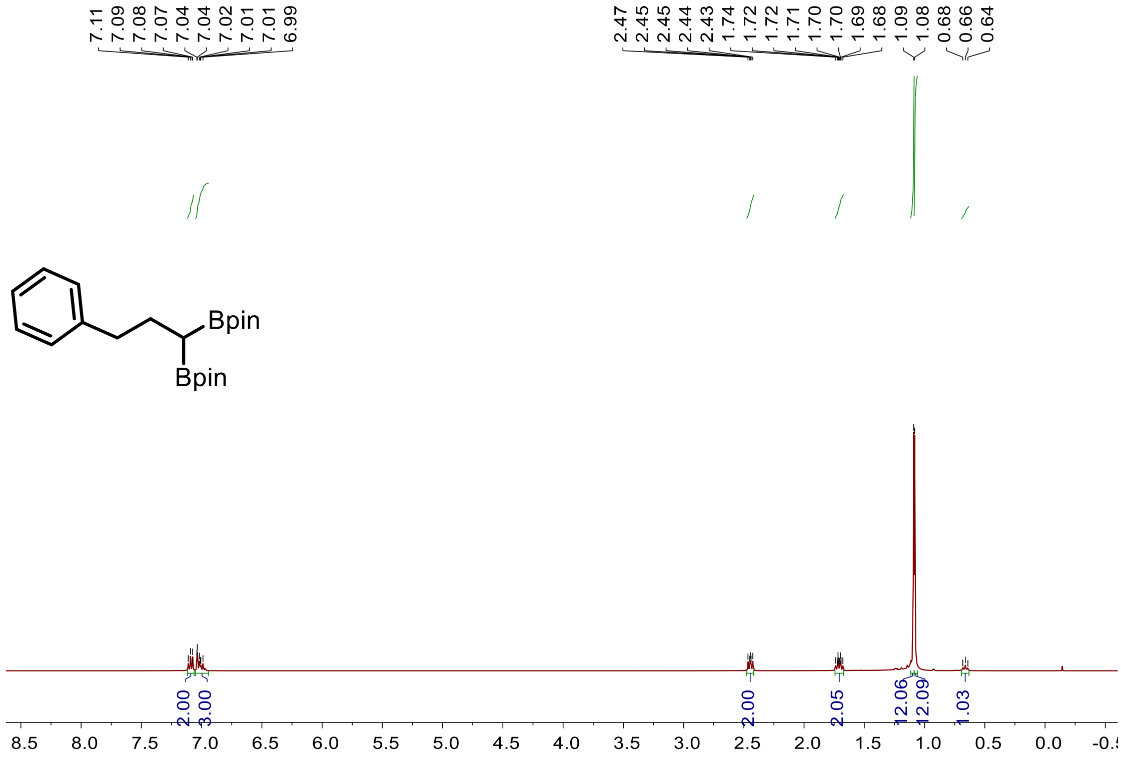

^{1}H NMR spectrum of **41** (Chloroform-*d*)

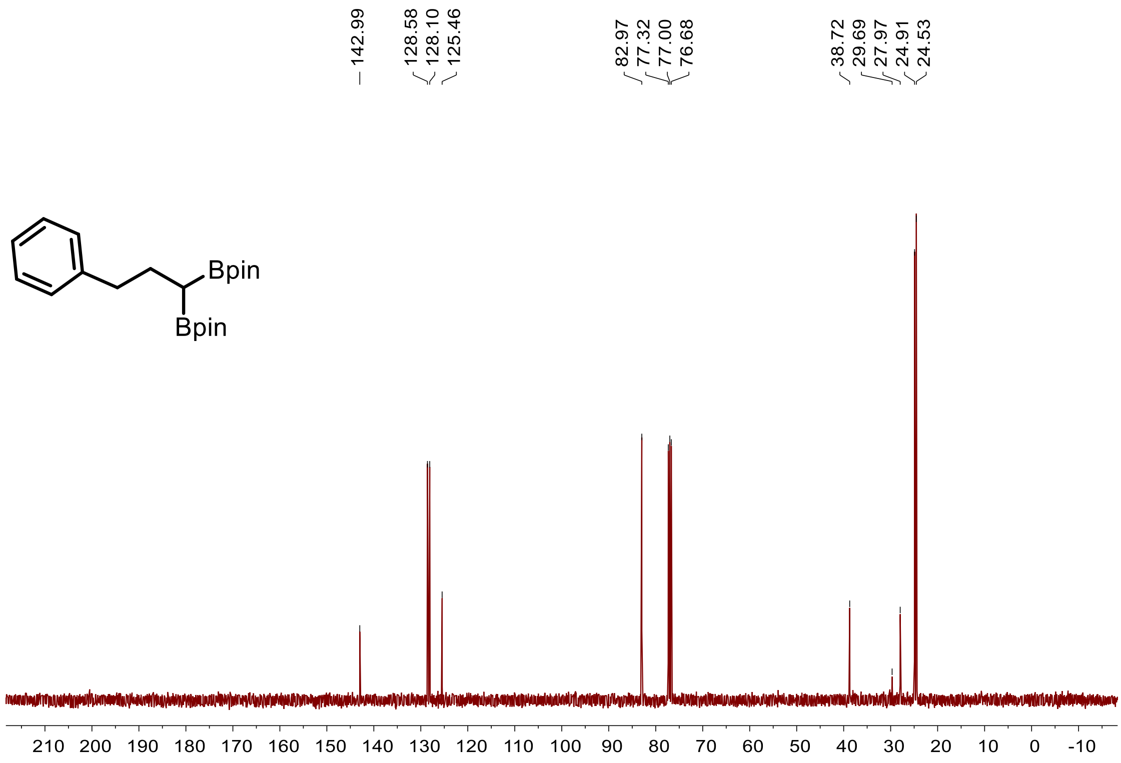

^{13}C NMR spectrum of **41** (Chloroform-*d*)

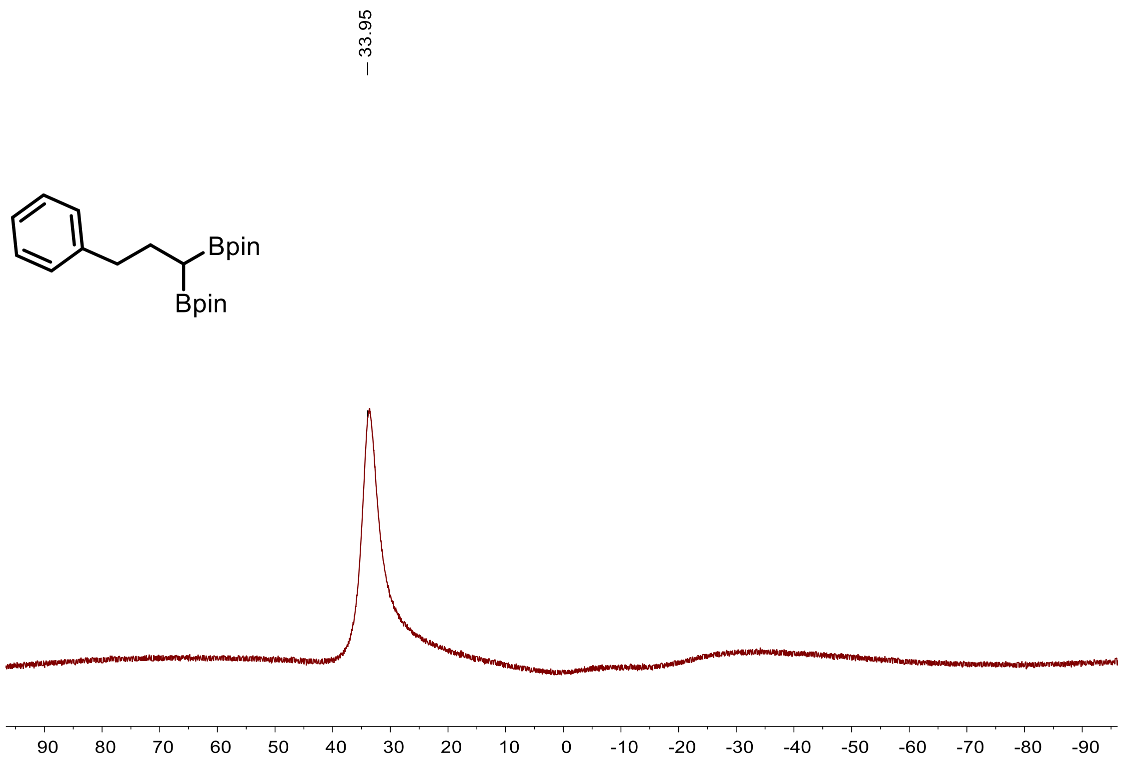

^{11}B NMR spectrum of **41** (Chloroform-*d*)

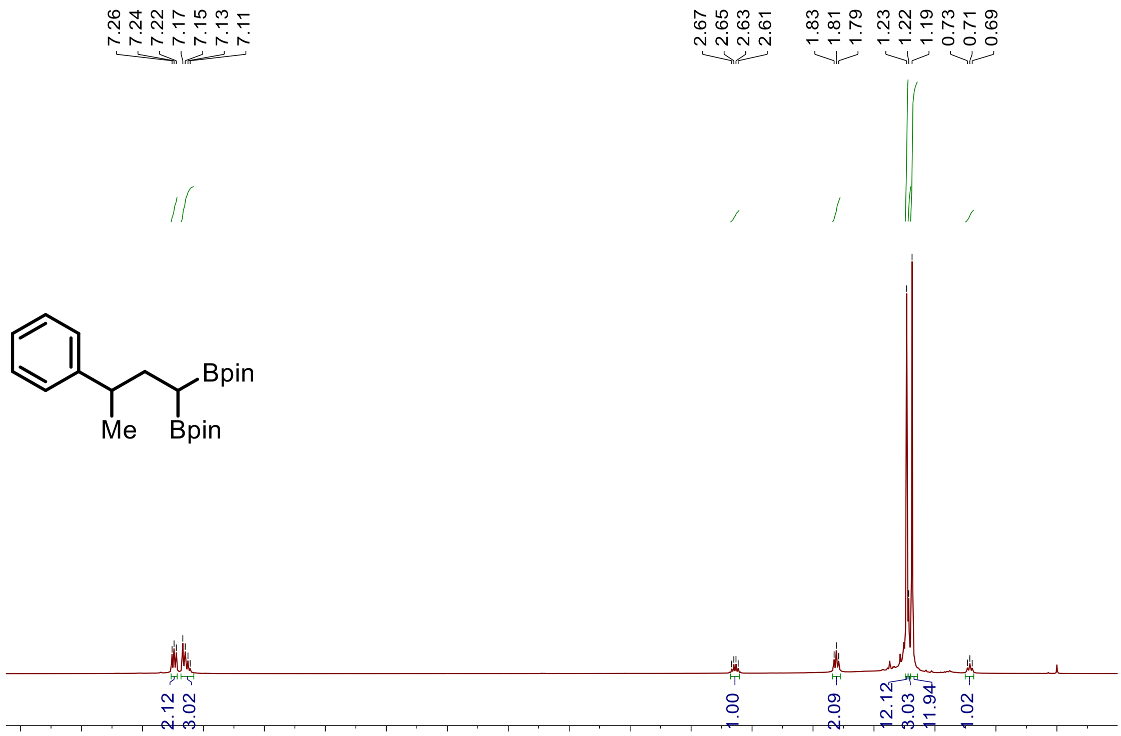

^{1}H NMR spectrum of **43** (Chloroform-*d*)

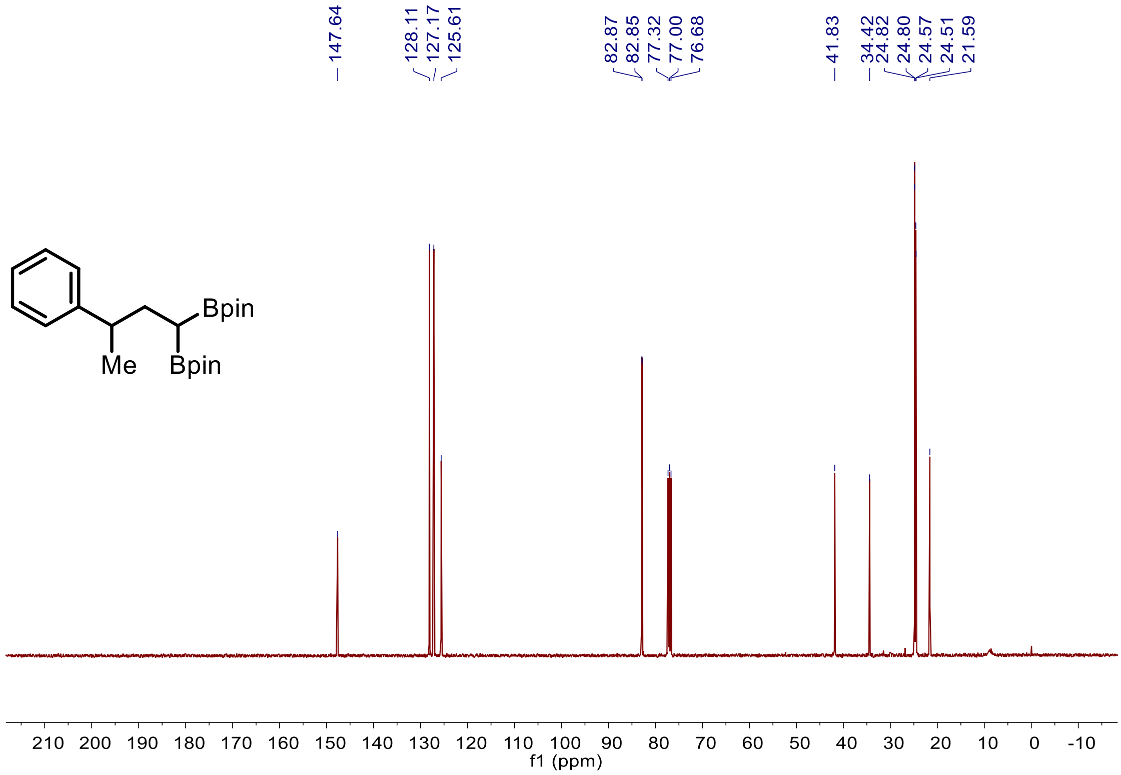

^{13}C NMR spectrum of **43** (Chloroform-*d*)

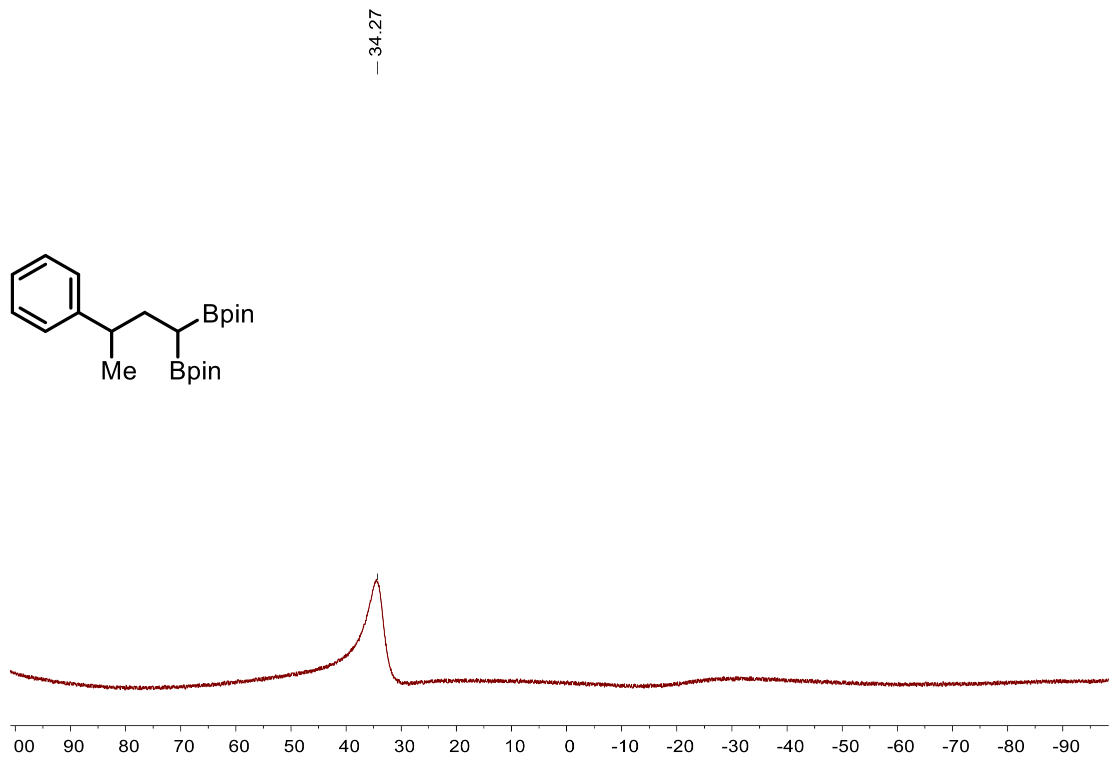

^{11}B NMR spectrum of **43** (Chloroform-*d*)

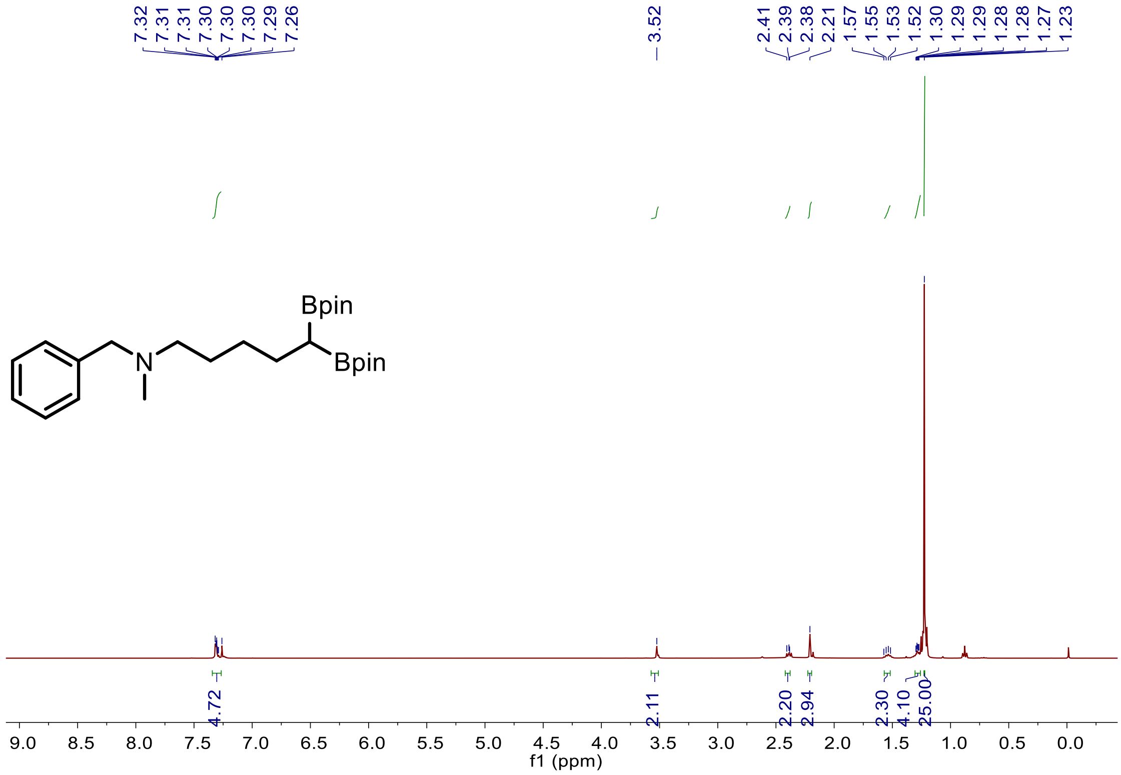

^{1}H NMR spectrum of **45** (Chloroform-*d*)

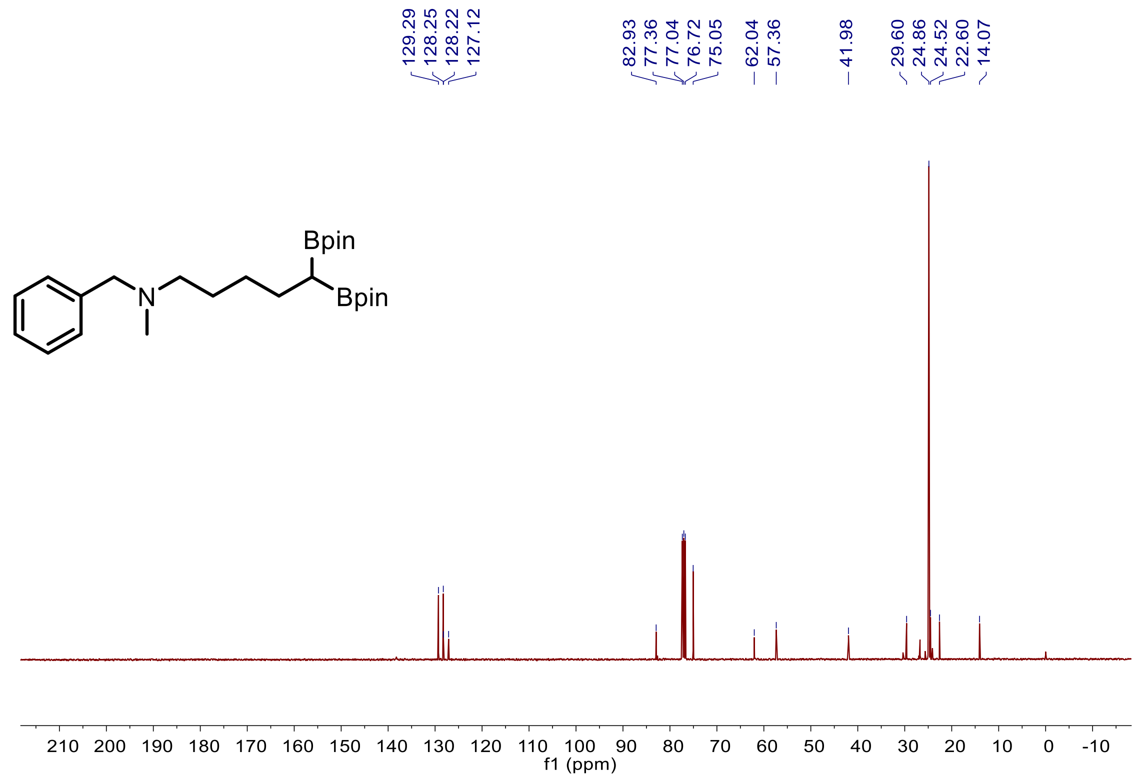

^{13}C NMR spectrum of **45** (Chloroform-*d*)

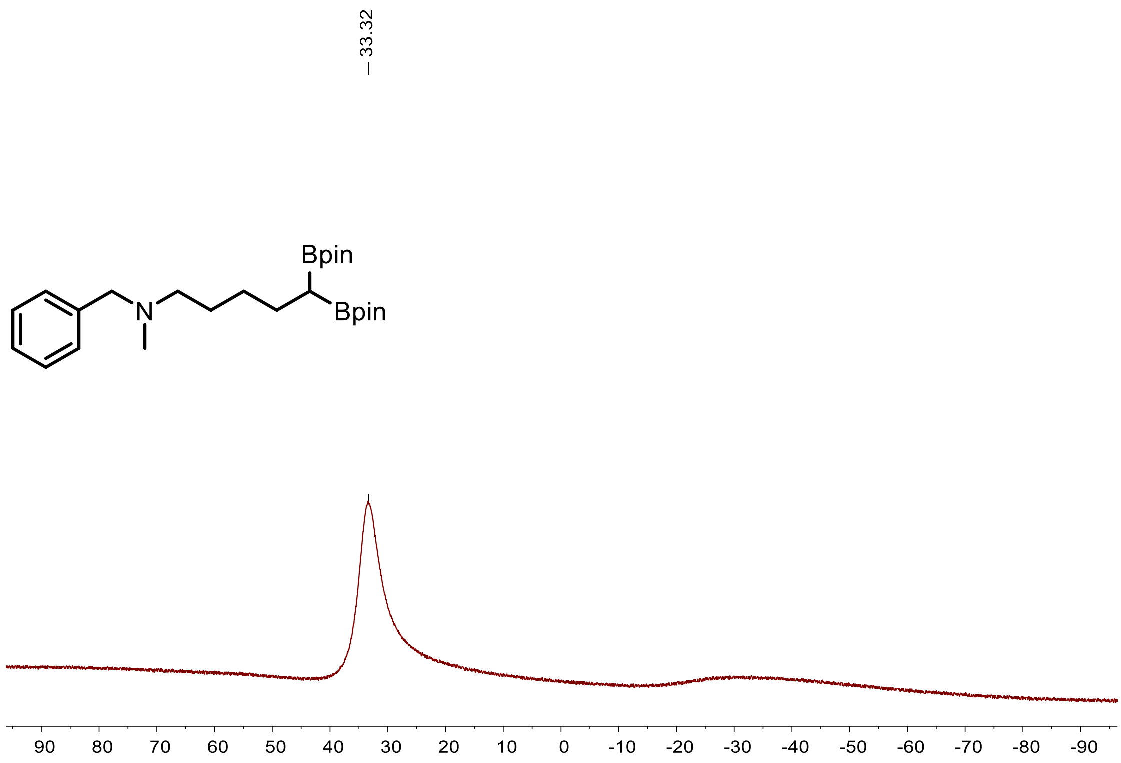

^{11}B NMR spectrum of **45** (Chloroform-*d*)

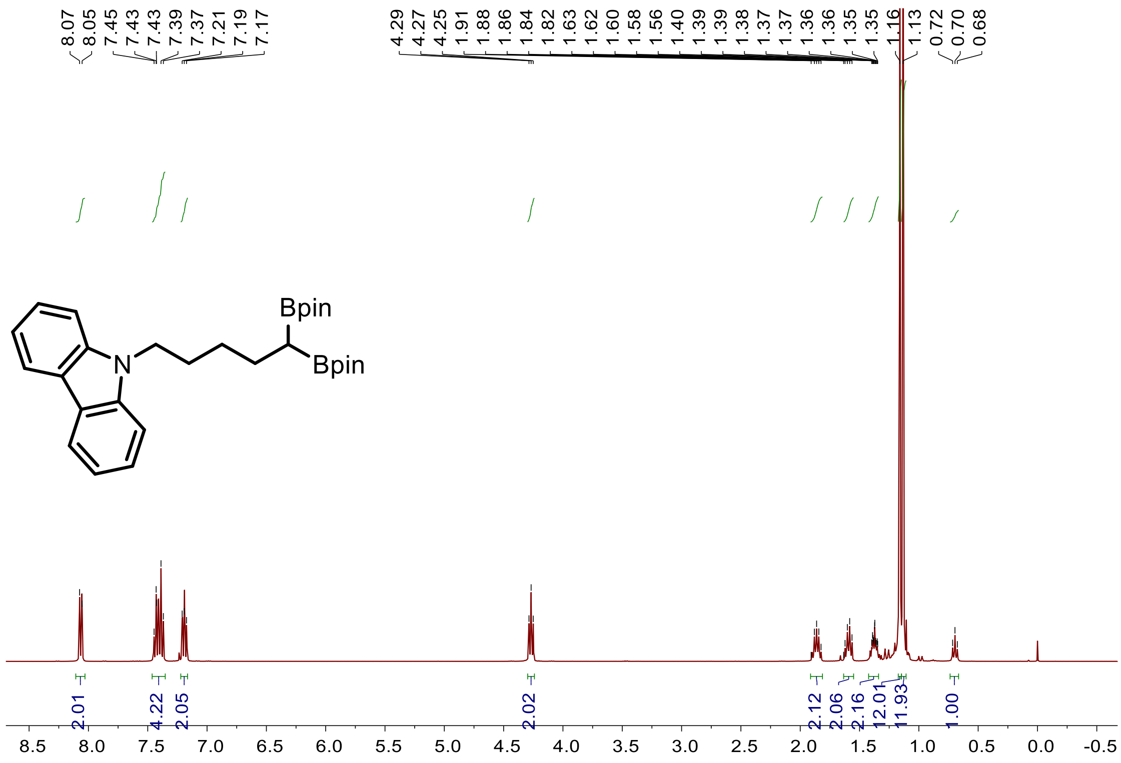

^{1}H NMR spectrum of **46** (Chloroform-*d*)

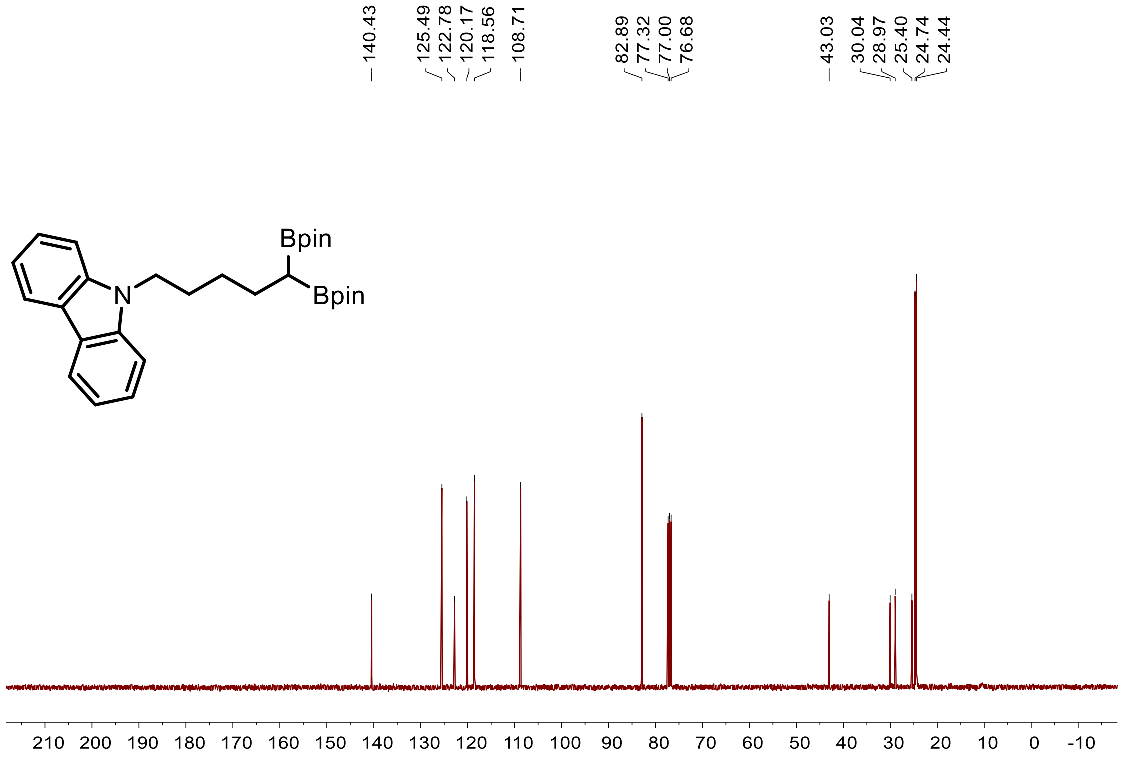

^{13}C NMR spectrum of **46** (Chloroform-*d*)

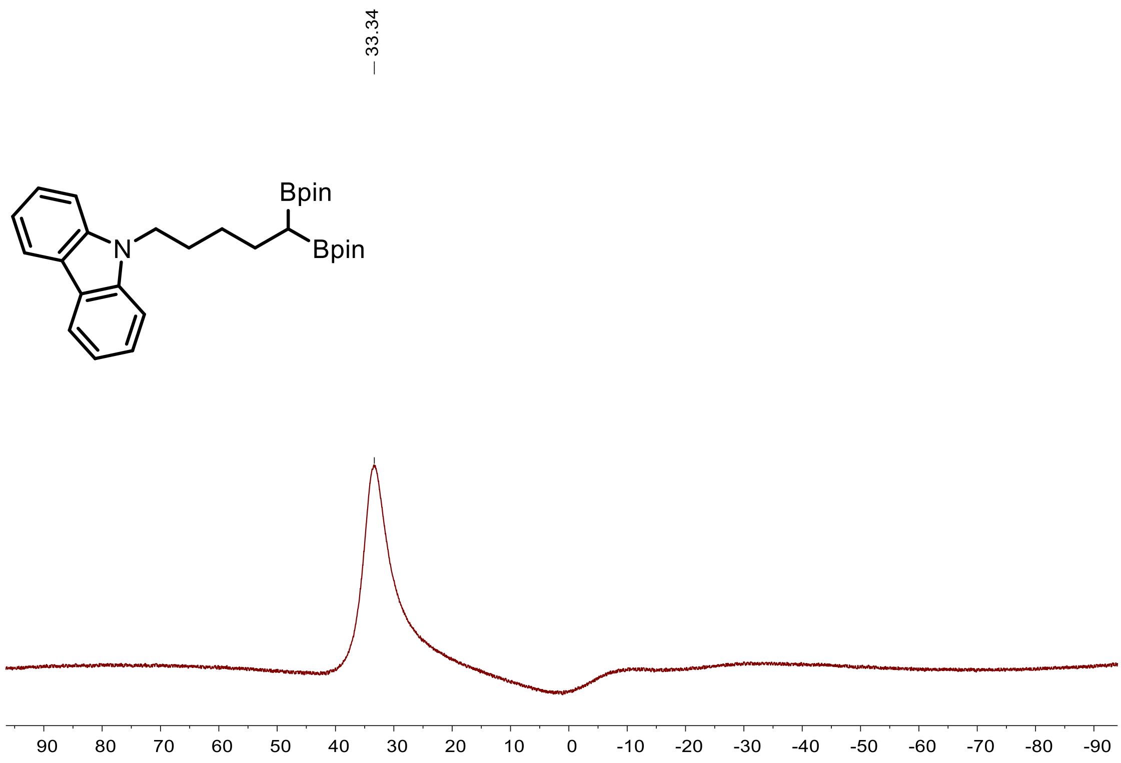

^{11}B NMR spectrum of **46** (Chloroform-*d*)

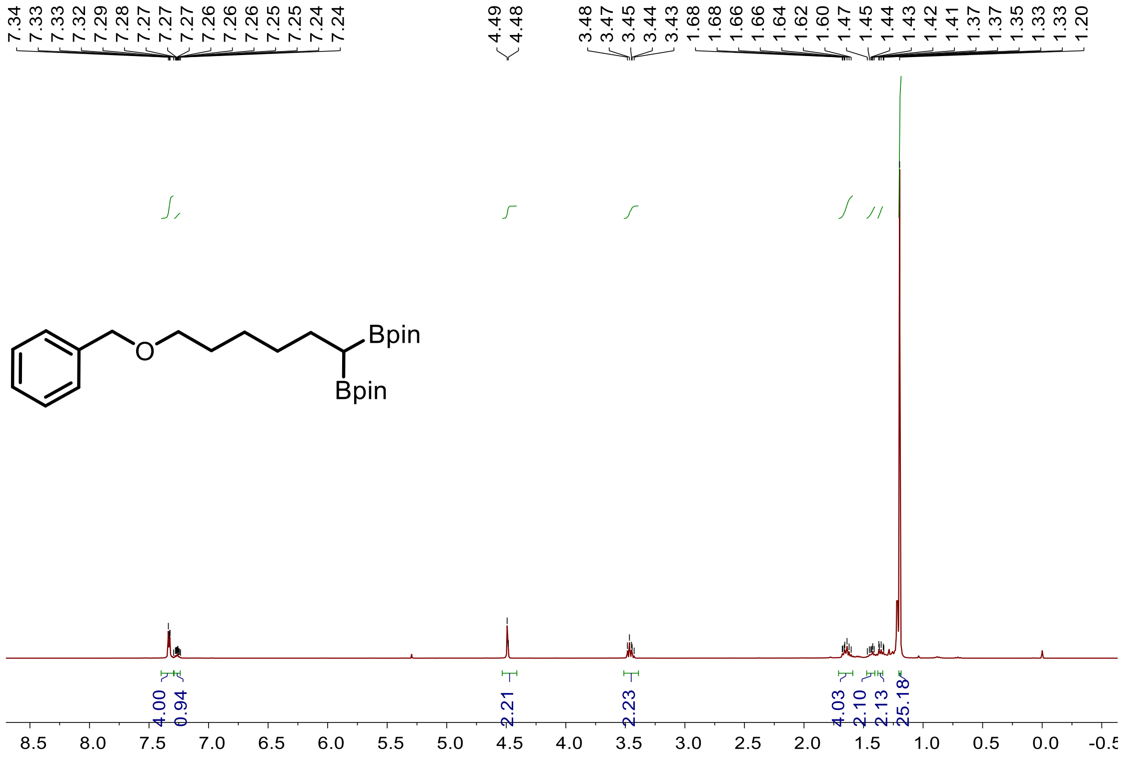

^{1}H NMR spectrum of **47** (Chloroform-*d*)

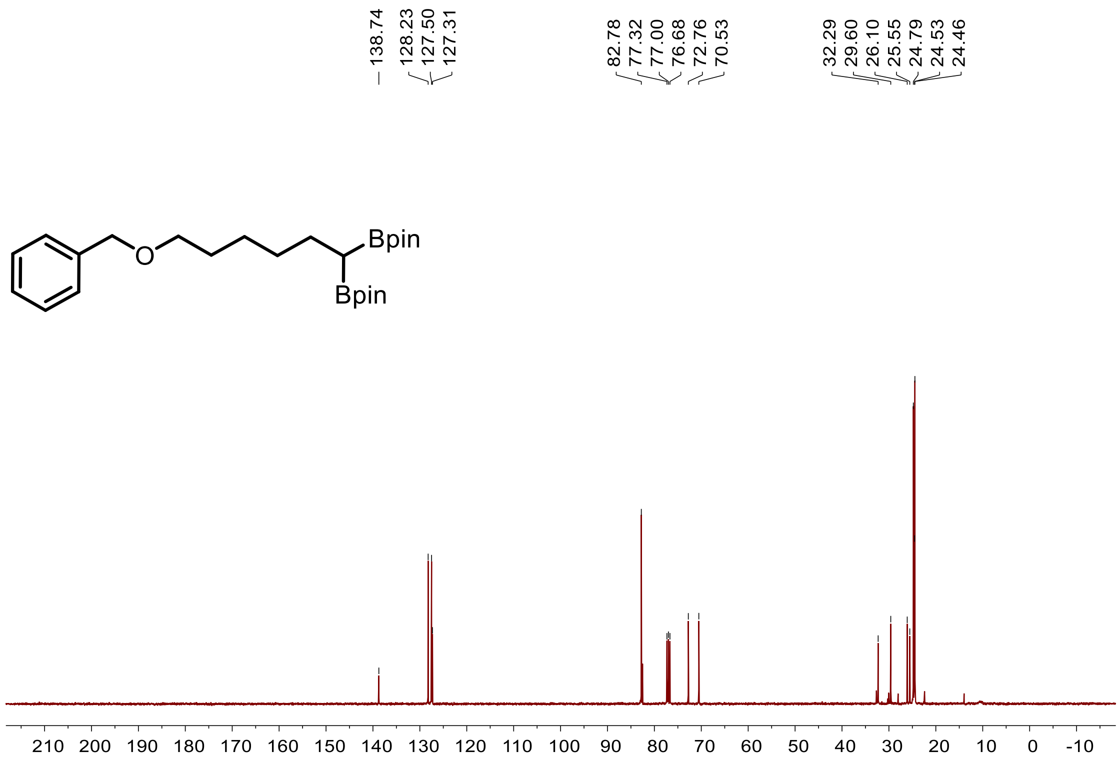

^{13}C NMR spectrum of **47** (Chloroform-*d*)

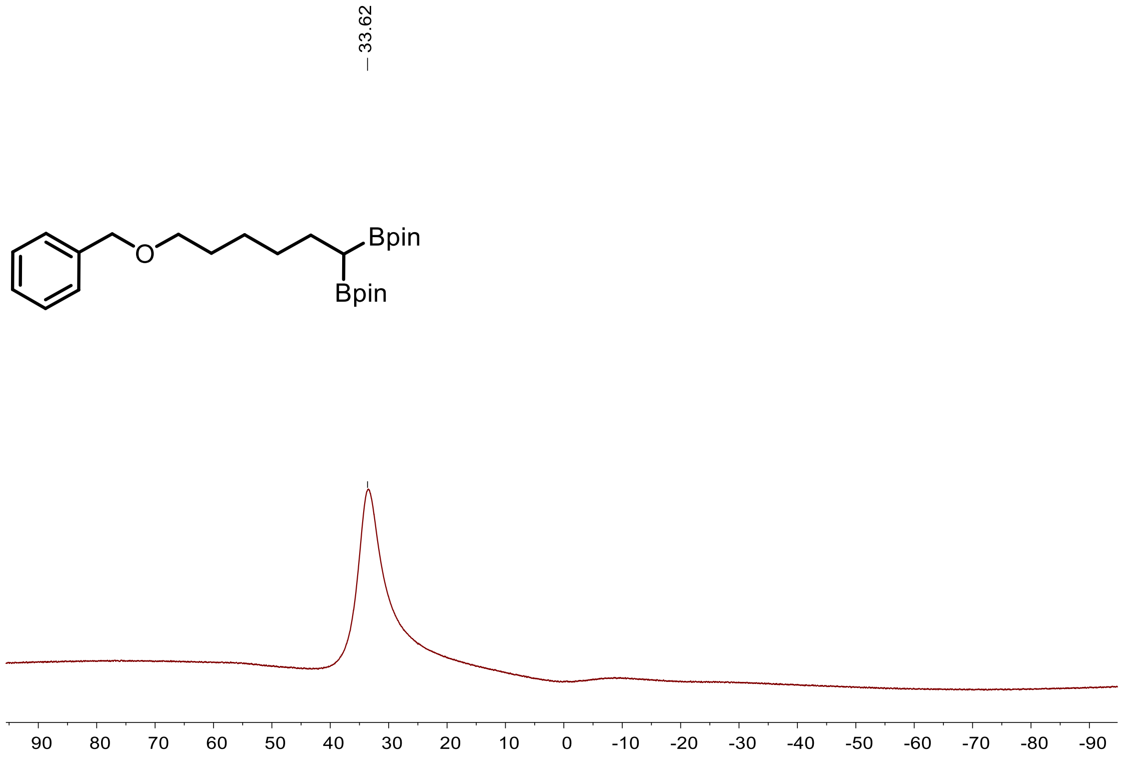

^{11}B NMR spectrum of **47** (Chloroform-*d*)

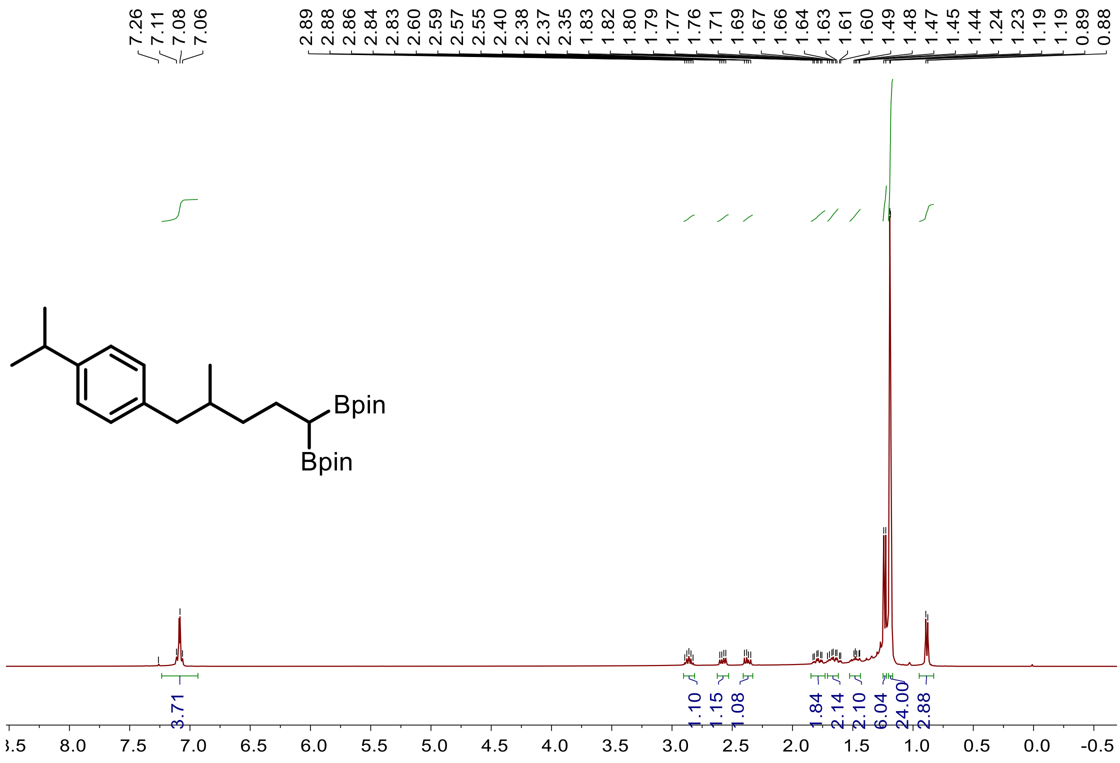

^{1}H NMR spectrum of **48** (Chloroform-*d*)

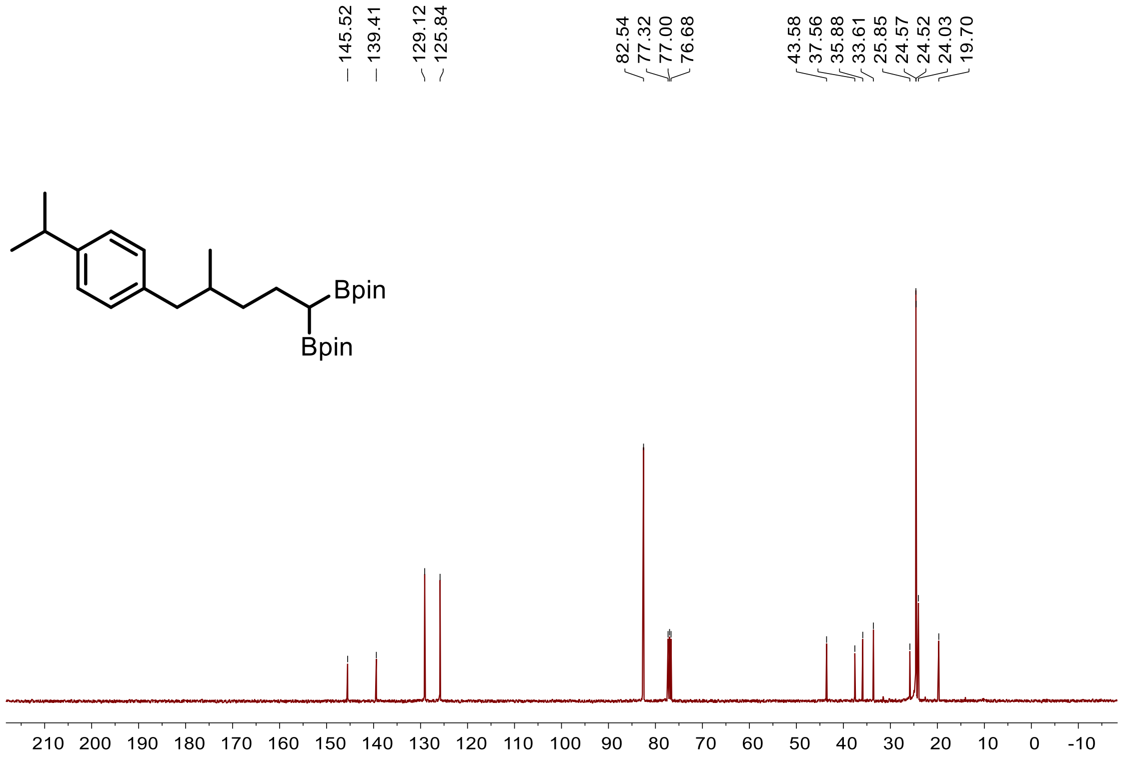

^{13}C NMR spectrum of **48** (Chloroform-*d*)

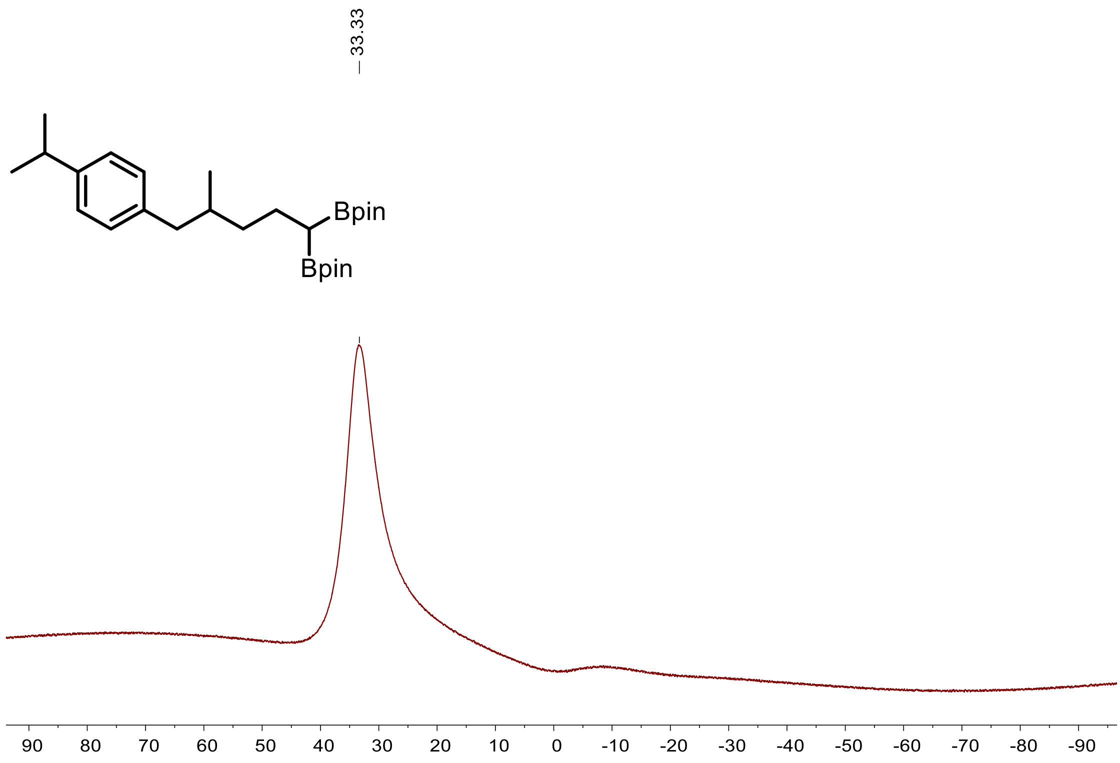

^{11}B NMR spectrum of **48** (Chloroform-*d*)

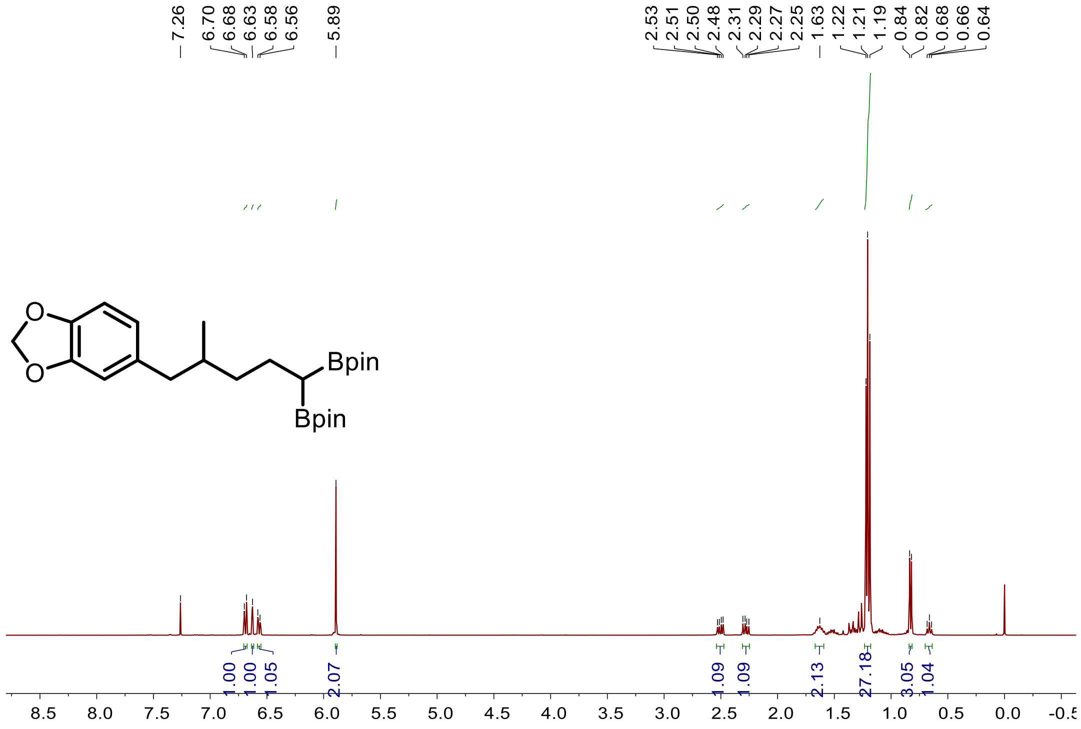

^{1}H NMR spectrum of **49** (Chloroform-*d*)

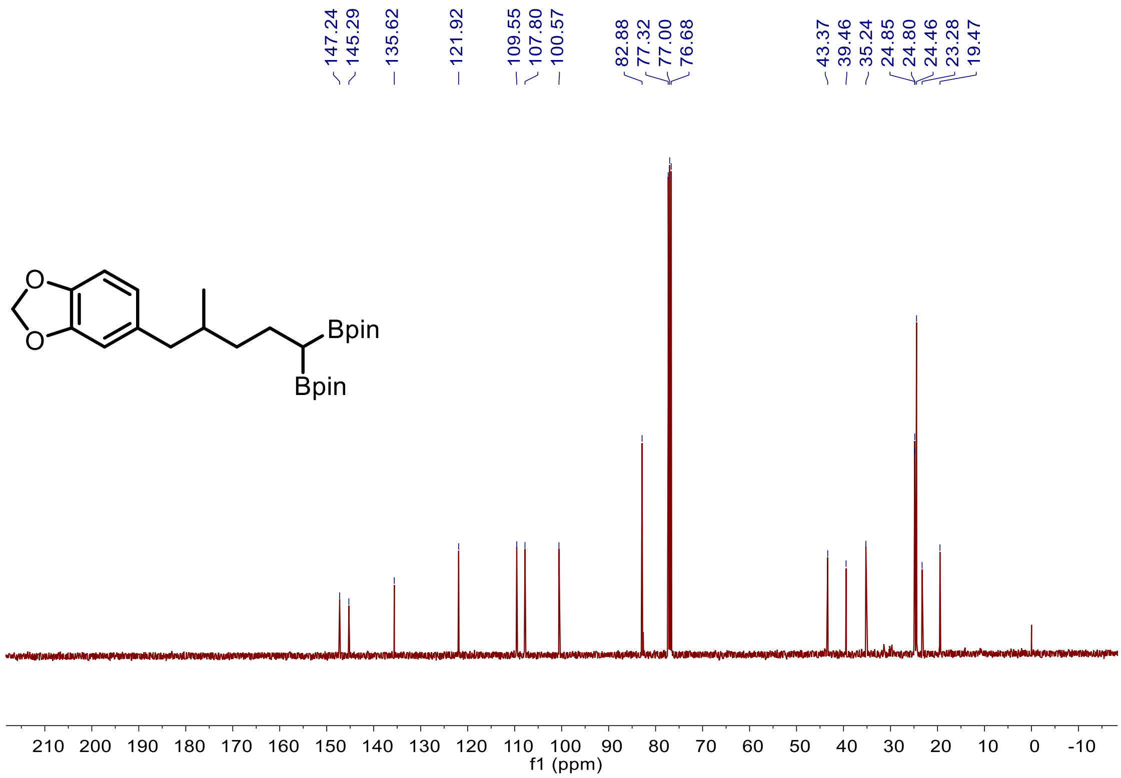

^{13}C NMR spectrum of **49** (Chloroform-*d*)

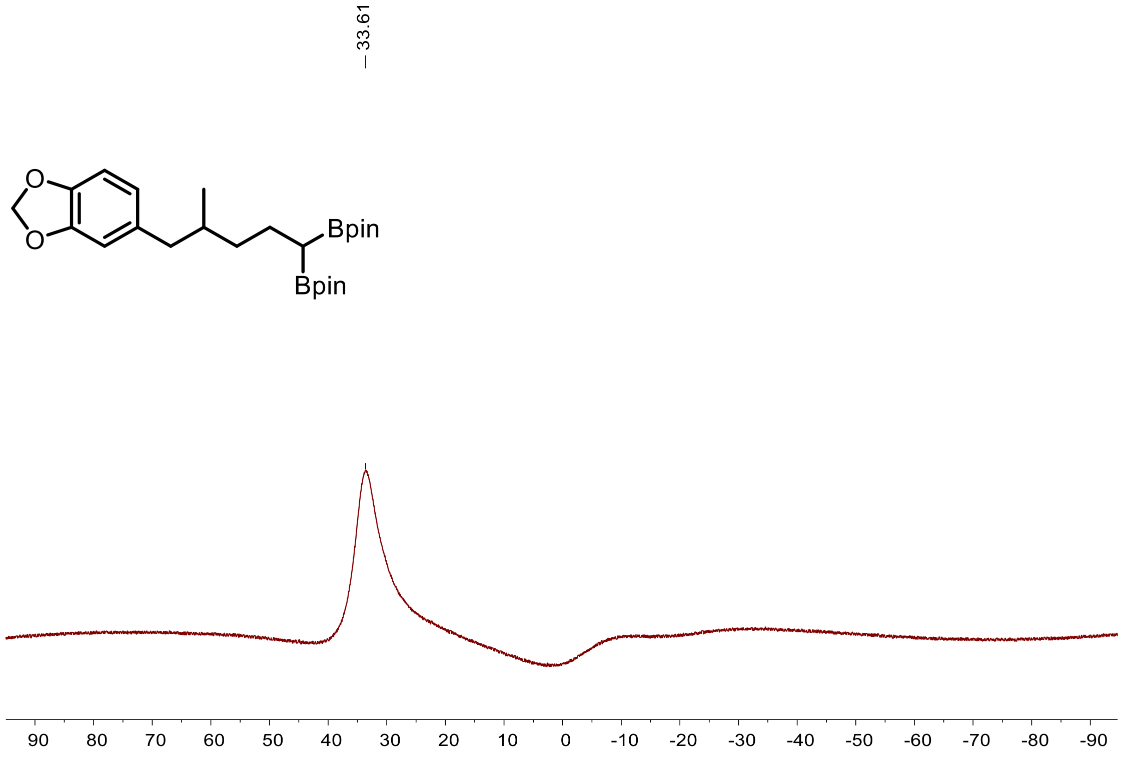

^{11}B NMR spectrum of **49** (Chloroform-*d*)

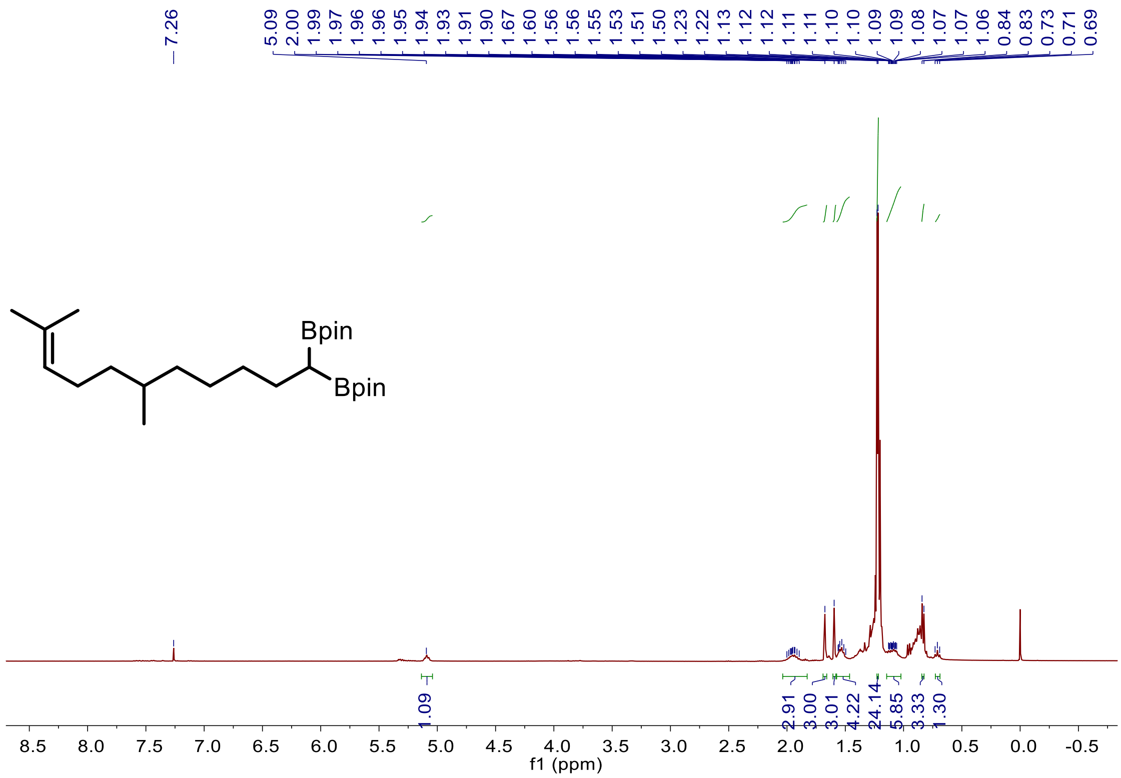

^{1}H NMR spectrum of **50** (Chloroform-*d*)

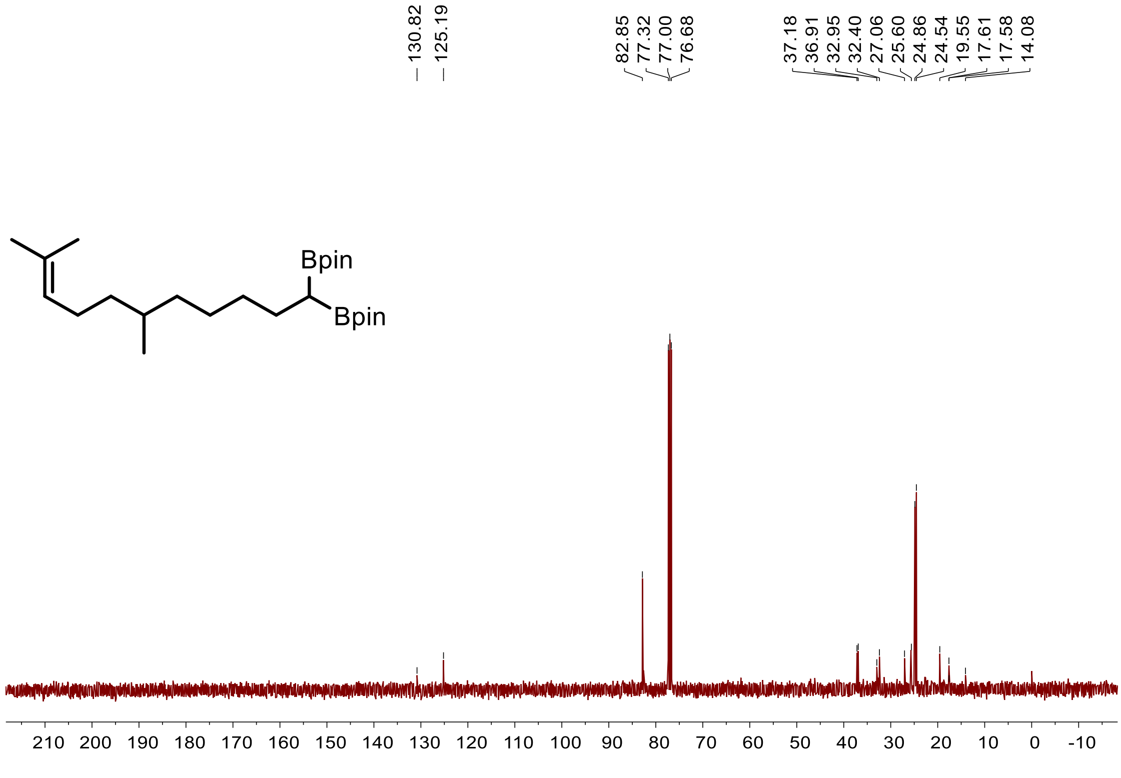

^{13}C NMR spectrum of **50** (Chloroform-*d*)

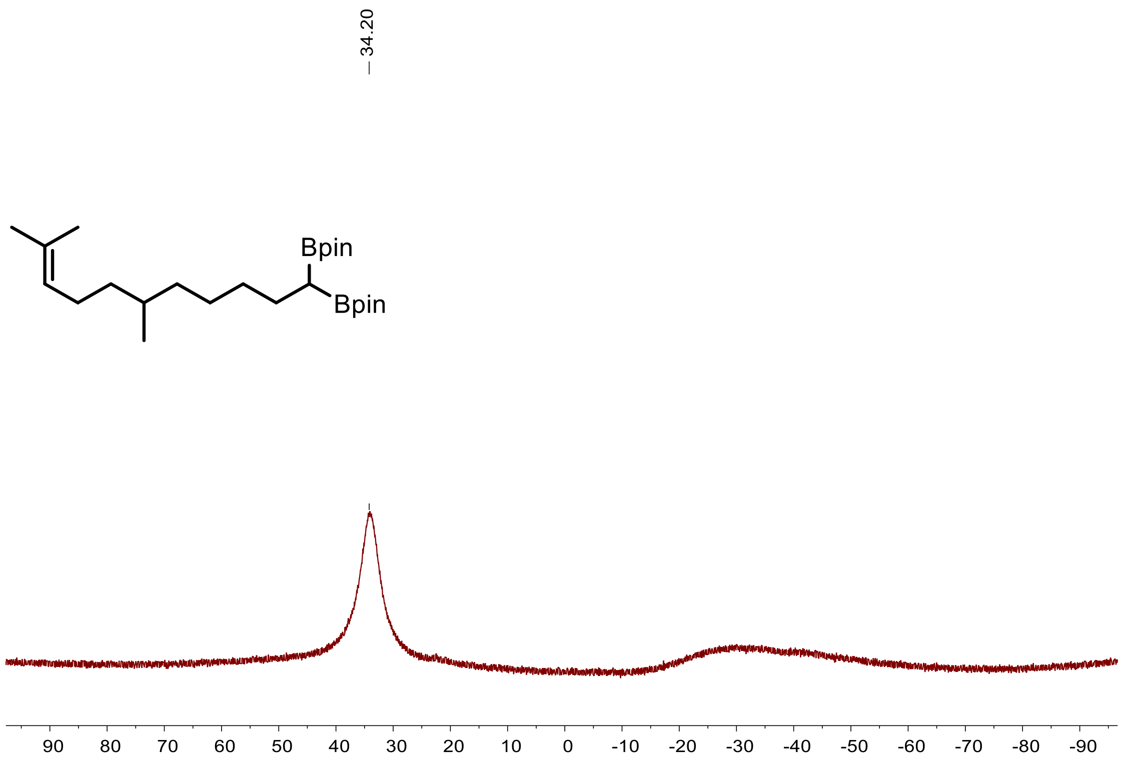

^{11}B NMR spectrum of **50** (Chloroform-*d*)

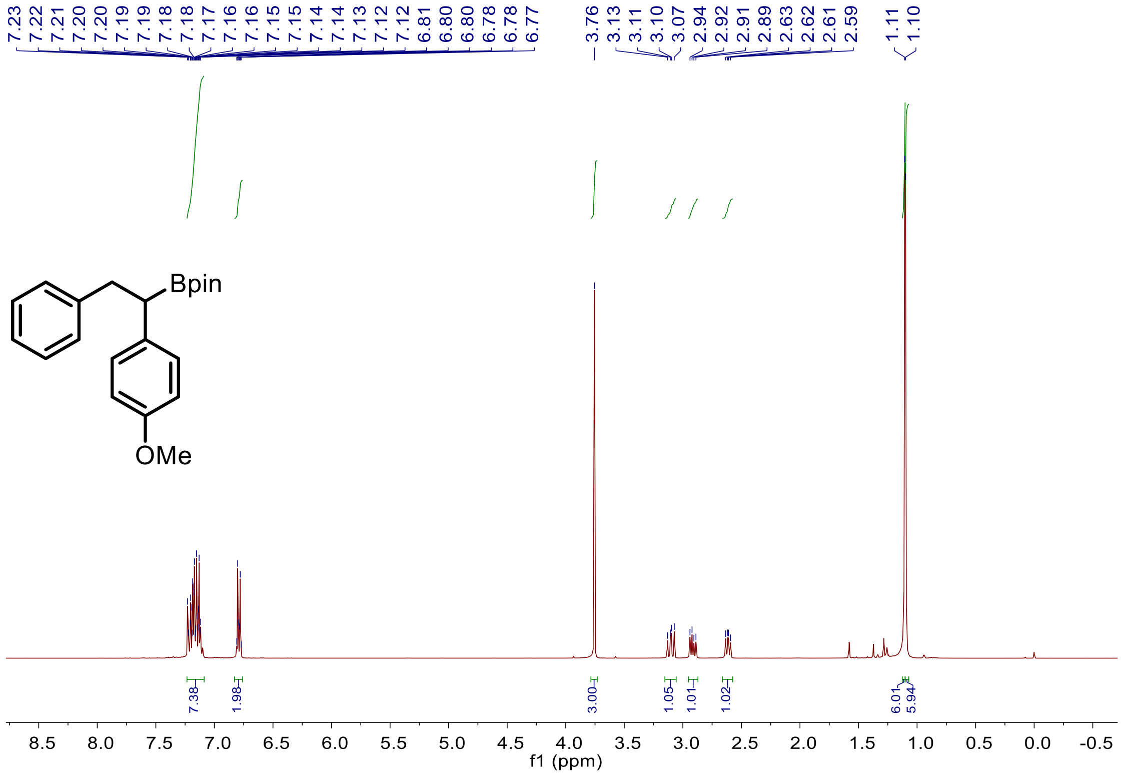

^{1}H NMR spectrum of **51** (Chloroform-*d*)

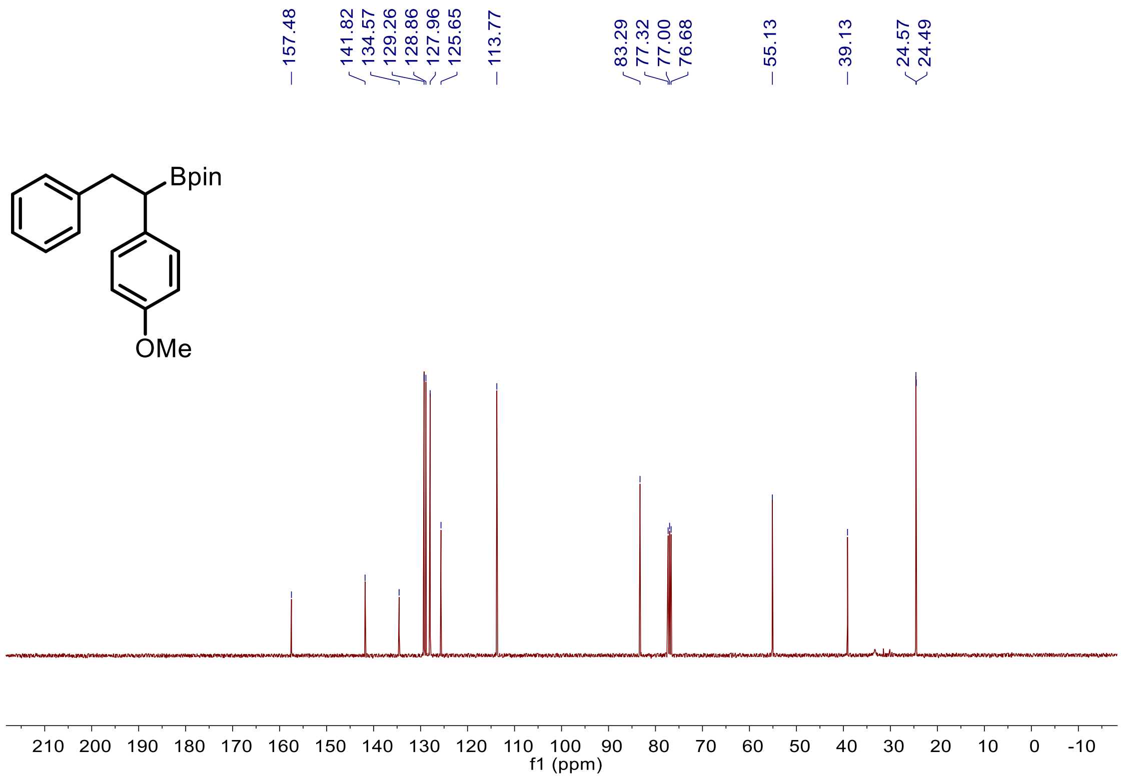

^{13}C NMR spectrum of **51** (Chloroform-*d*)

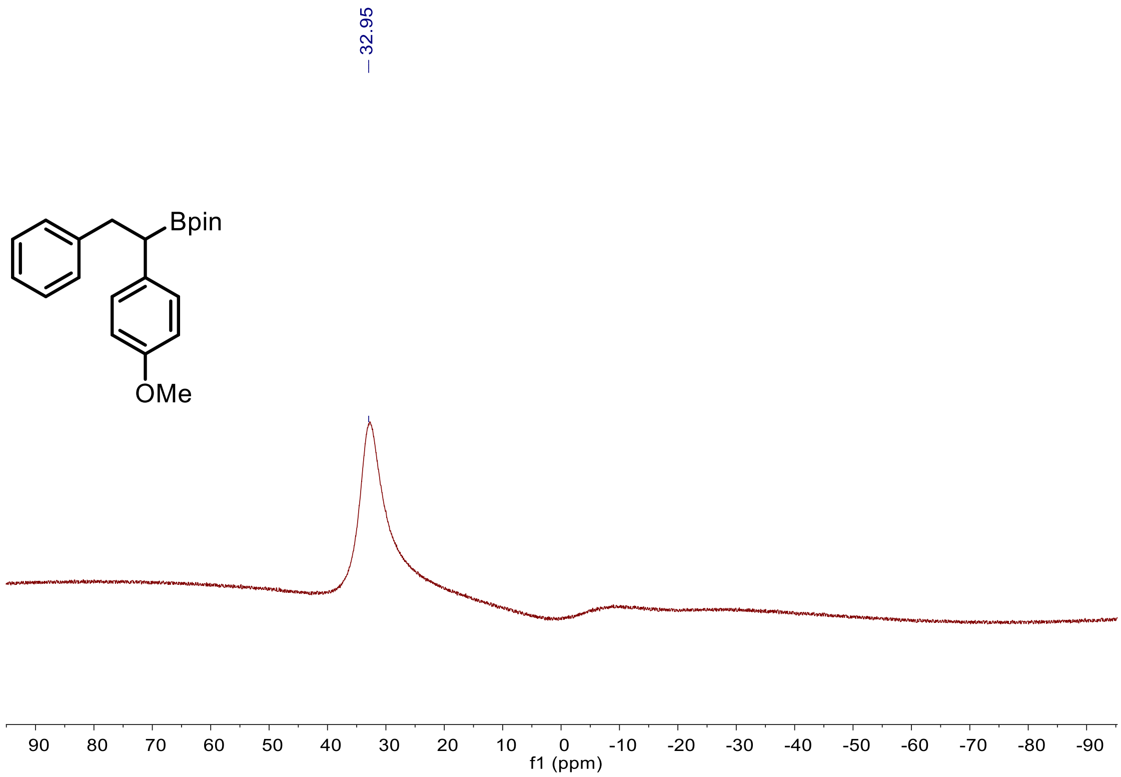

^{11}B NMR spectrum of **51** (Chloroform-*d*)

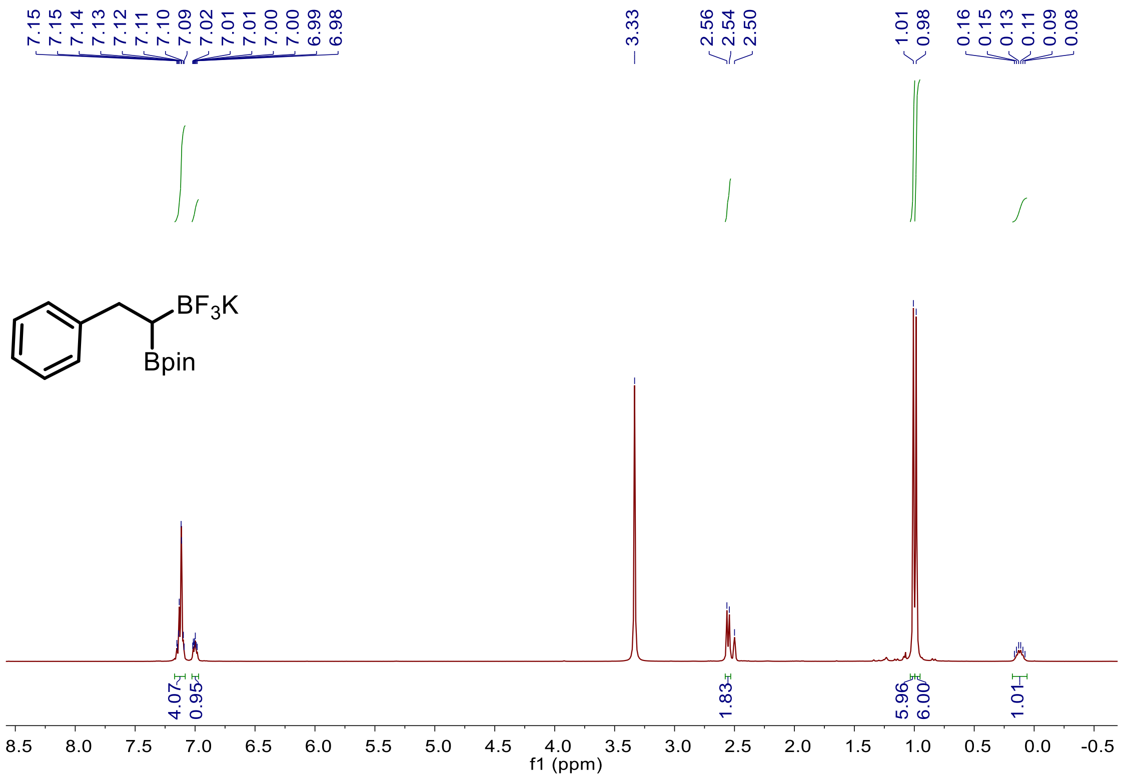

^{1}H NMR spectrum of **52** (Dimethyl sulfoxide-d_6)

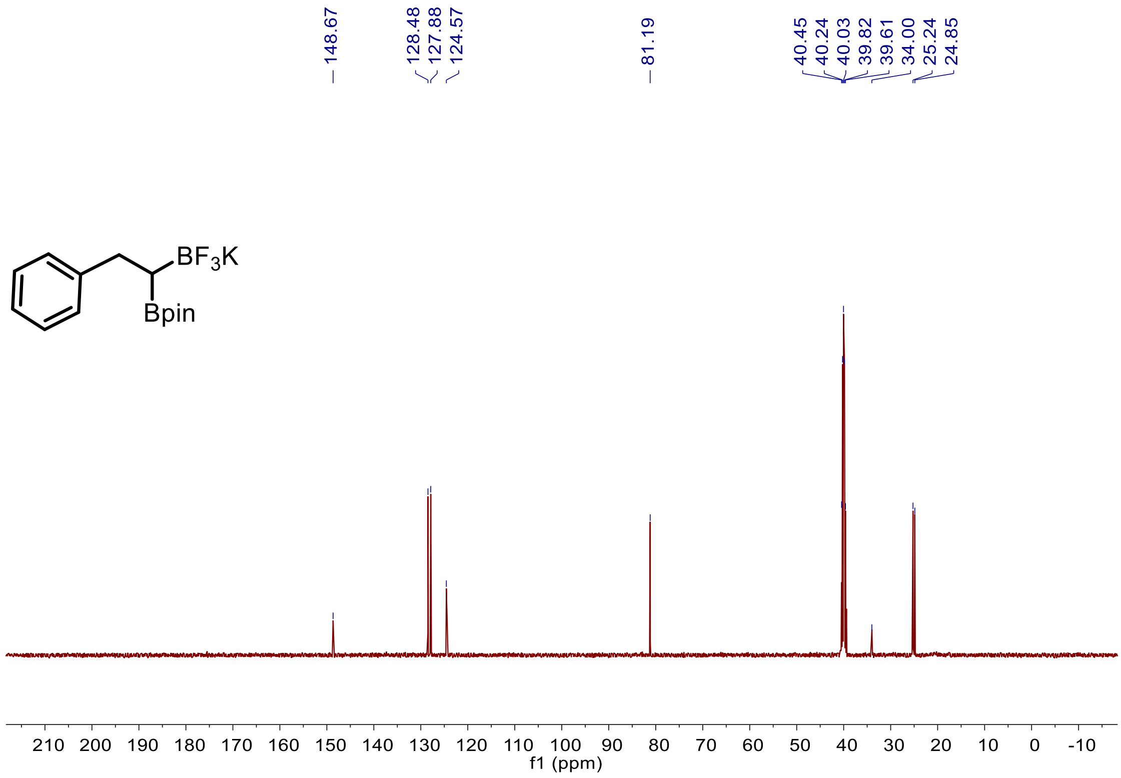

^{13}C NMR spectrum of **52** (Dimethyl sulfoxide-d_6)

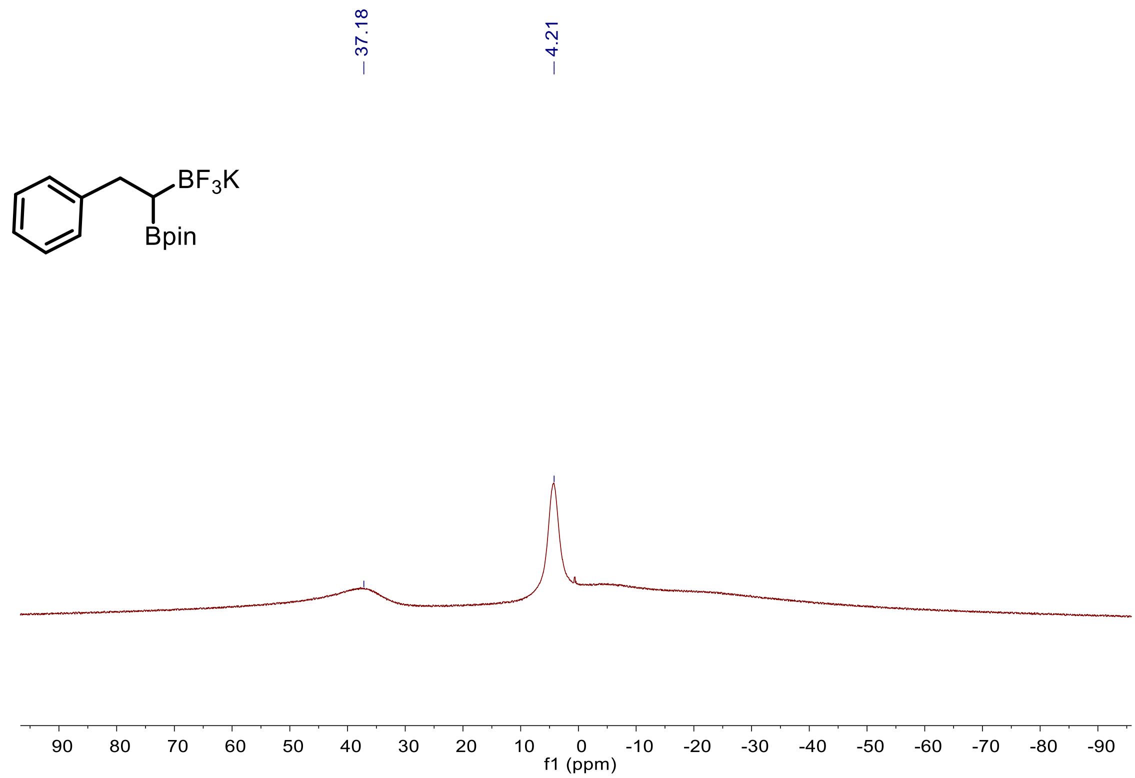

^{11}B NMR spectrum of **52** (Dimethyl sulfoxide-d_6)

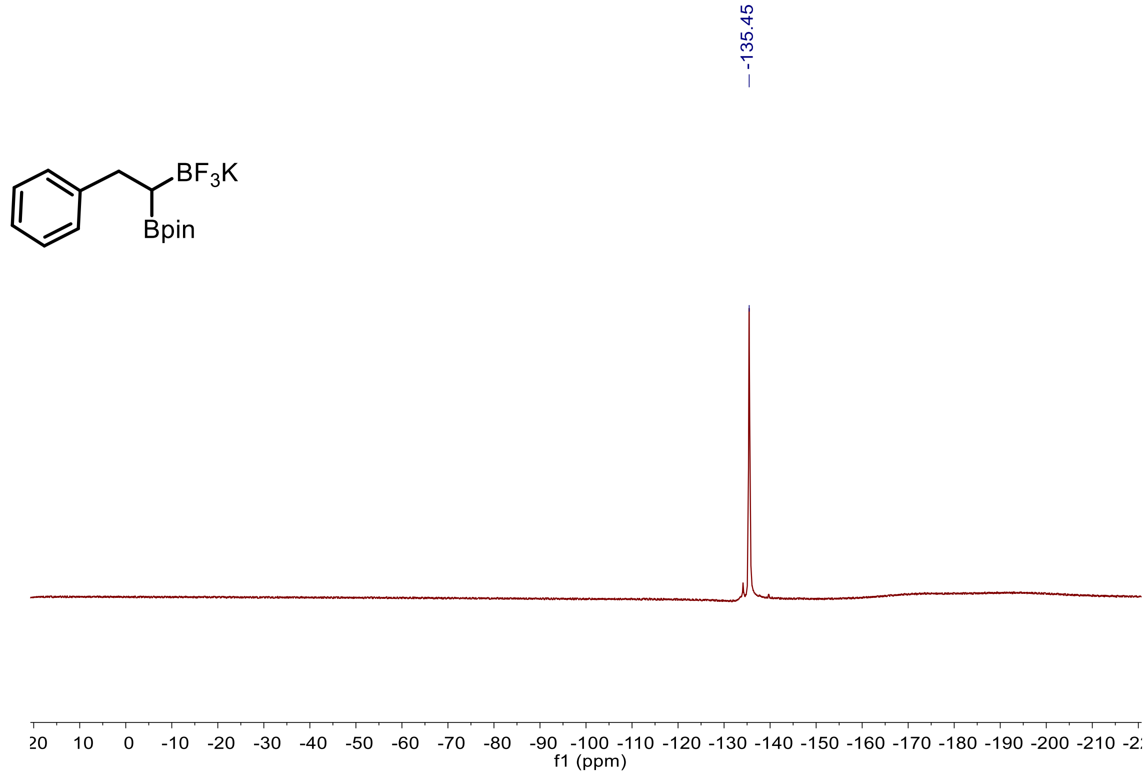

^{19}F NMR spectrum of **52** (Dimethyl sulfoxide-d_6)

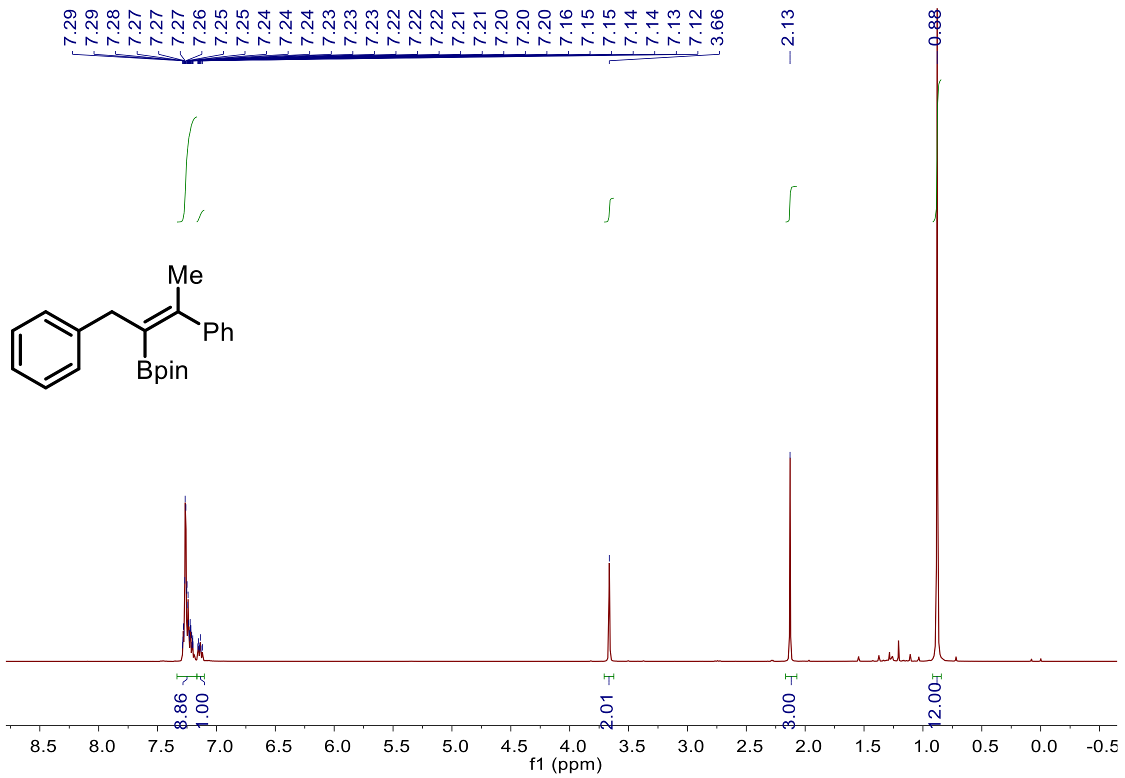

^{1}H NMR spectrum of **53** (Chloroform-*d*)

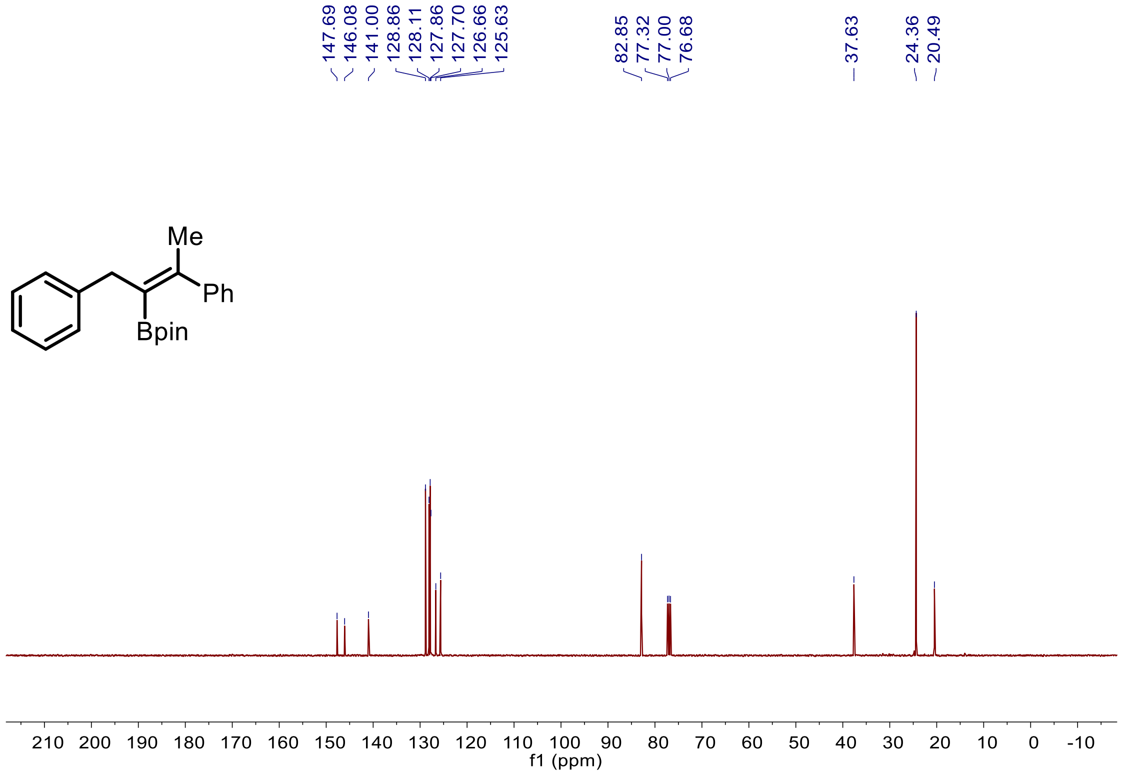

^{13}C NMR spectrum of **53** (Chloroform-*d*)

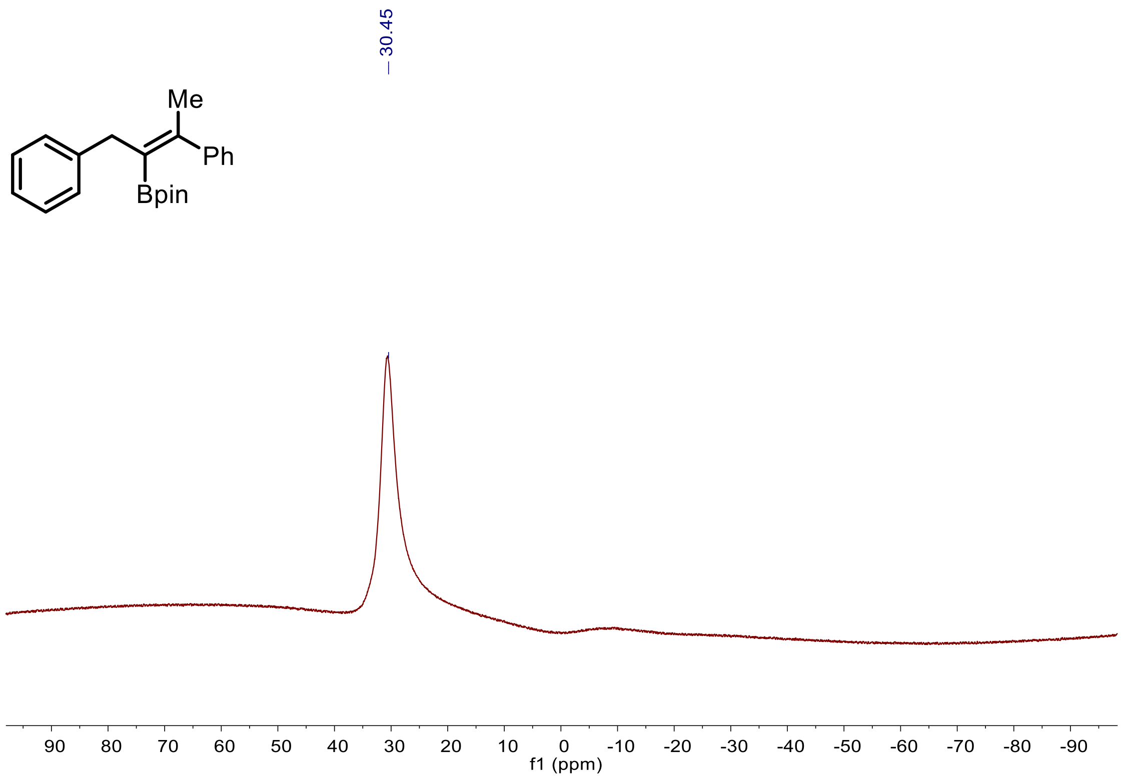

^{11}B NMR spectrum of **53** (Chloroform-*d*)

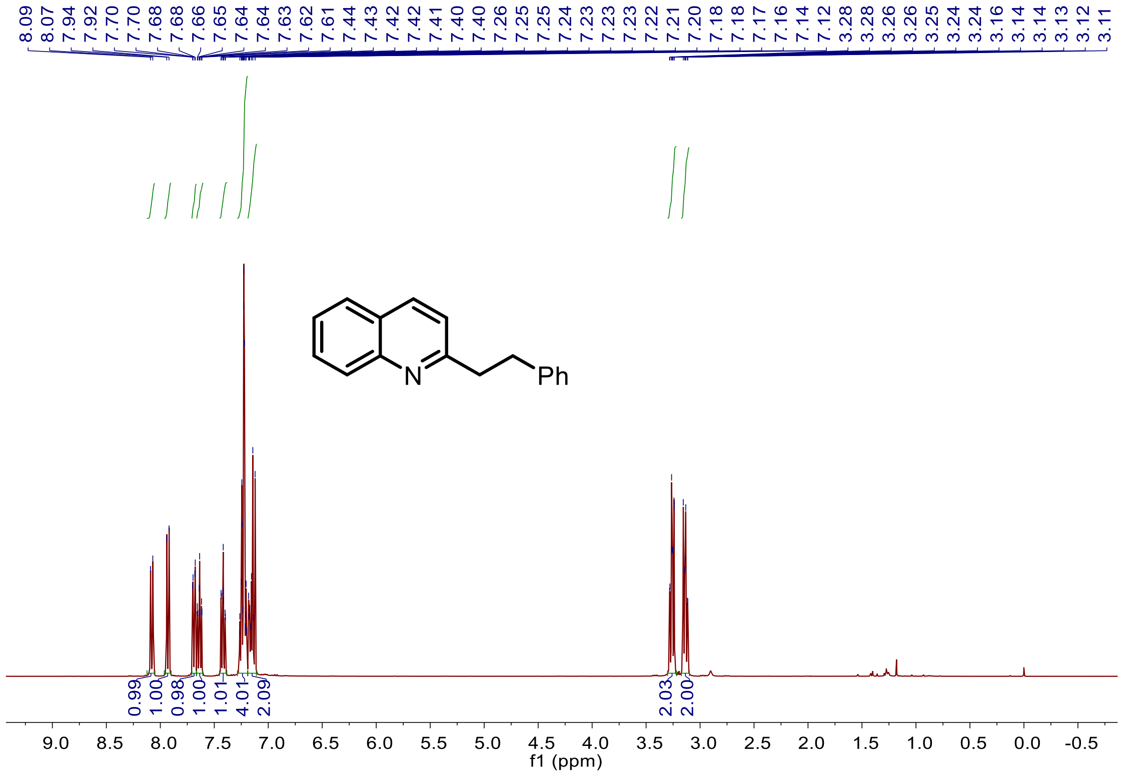

^{1}H NMR spectrum of **54** (Chloroform-*d*)

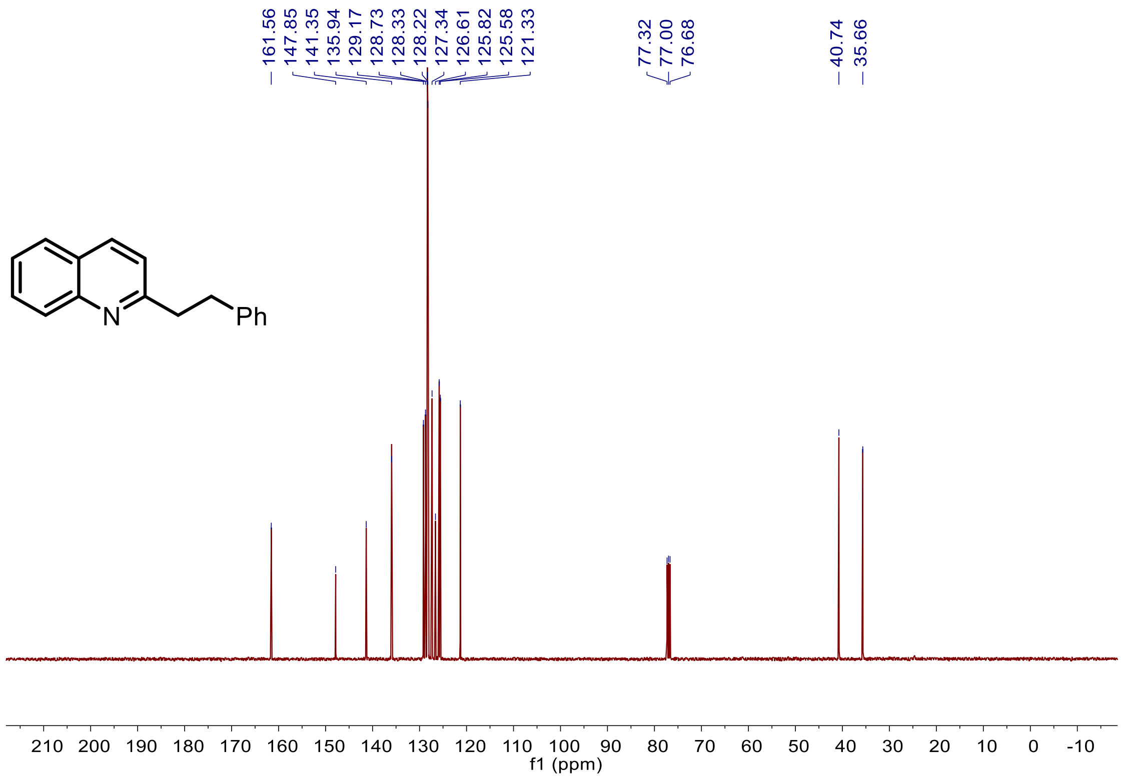

^{13}C NMR spectrum of **54** (Chloroform-*d*)

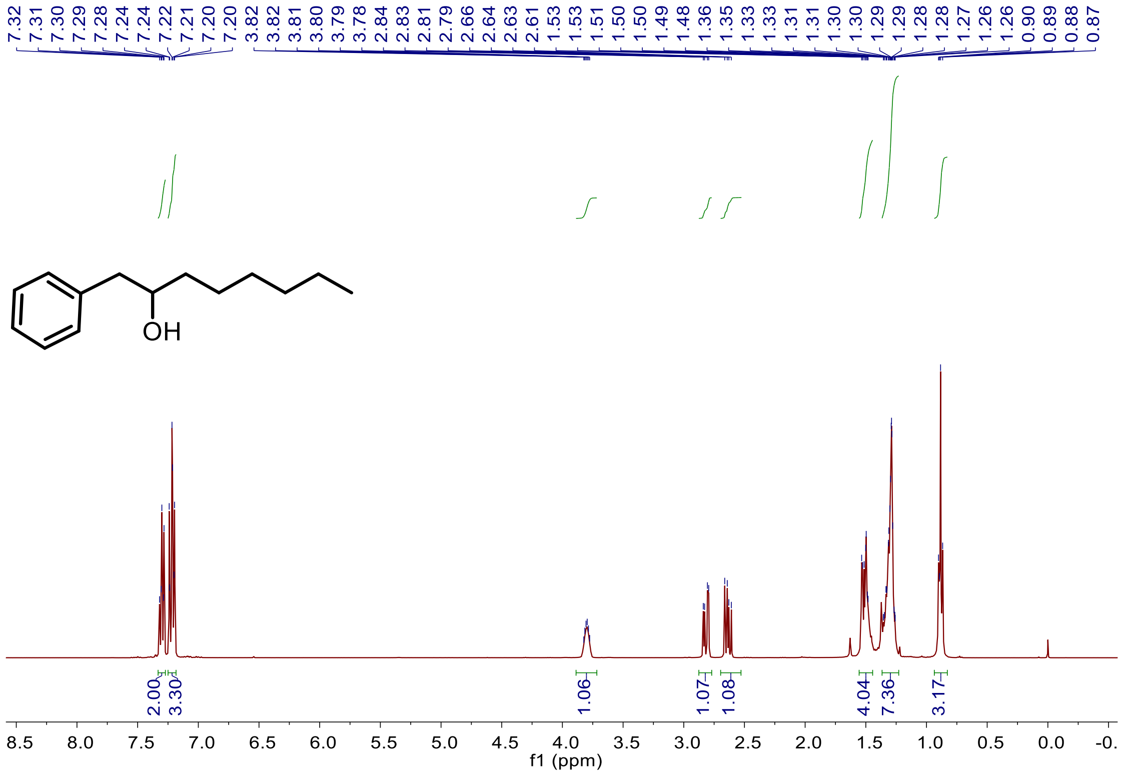

^{1}H NMR spectrum of **55** (Chloroform-*d*)

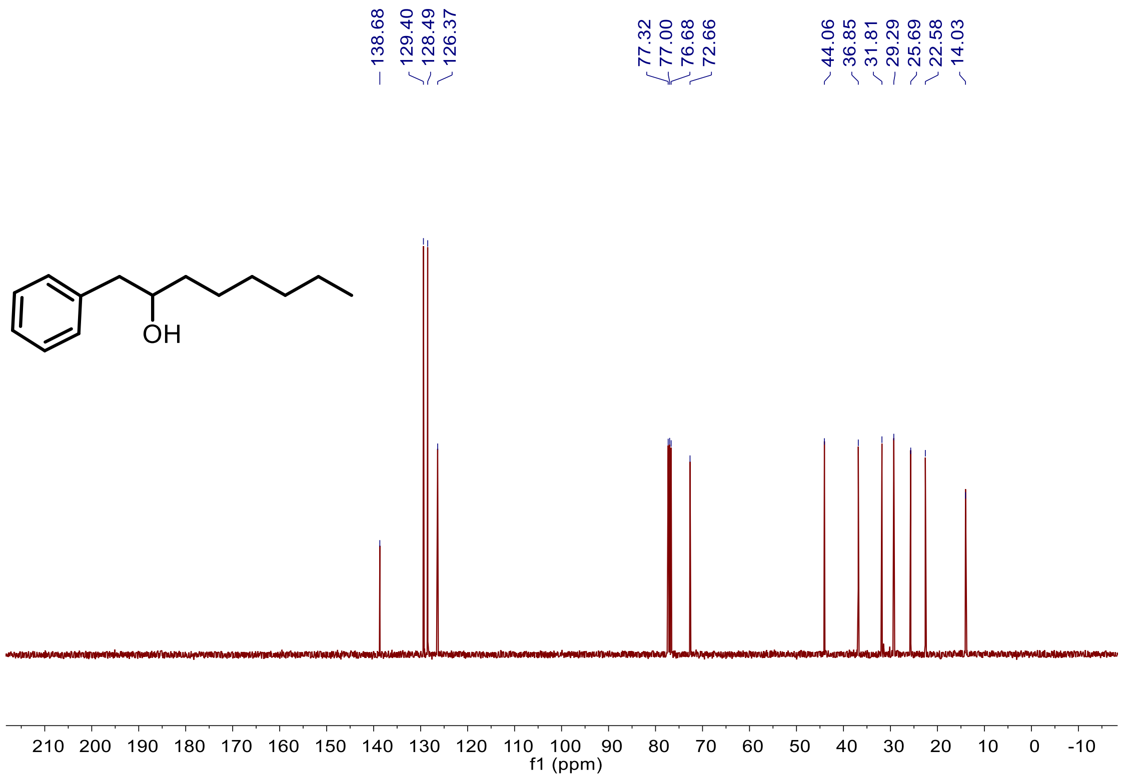

^{13}C NMR spectrum of **55** (Chloroform-*d*)

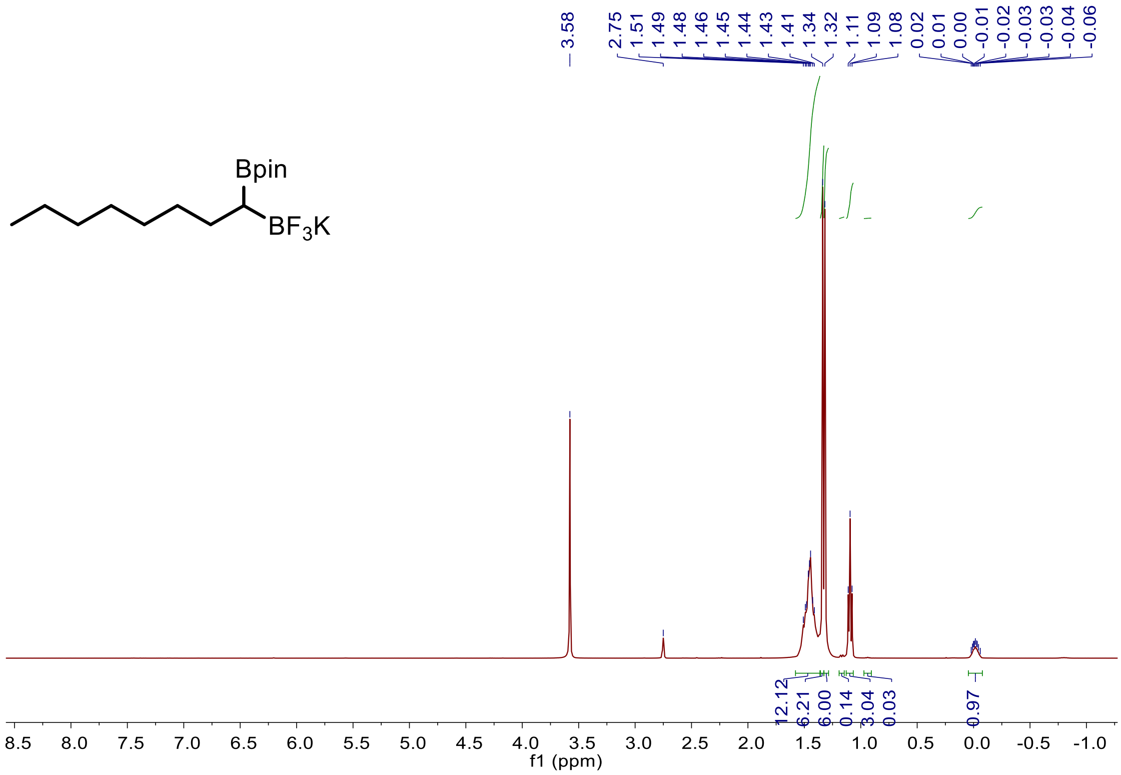

^{1}H NMR spectrum of **56** (Dimethyl sulfoxide-*d*$_6$)

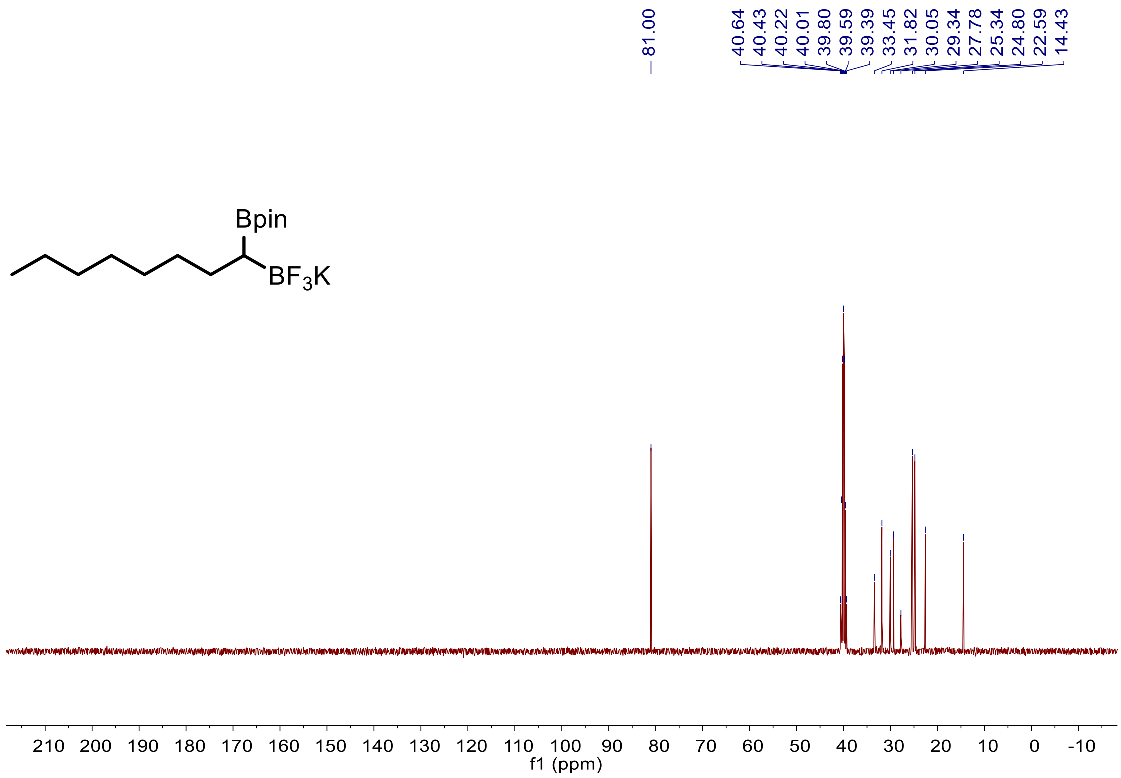

^{13}C NMR spectrum of **56** (Dimethyl sulfoxide-*d*$_6$)

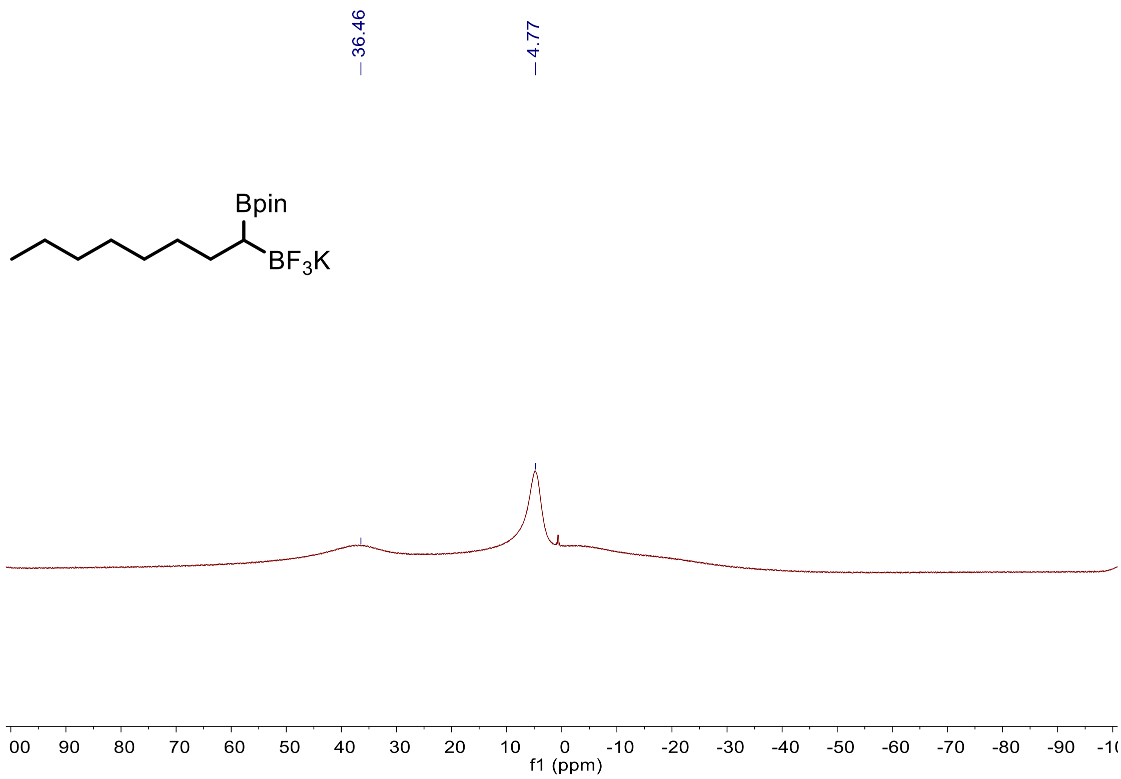

^{11}B NMR spectrum of **56** (Dimethyl sulfoxide-*d*$_6$)

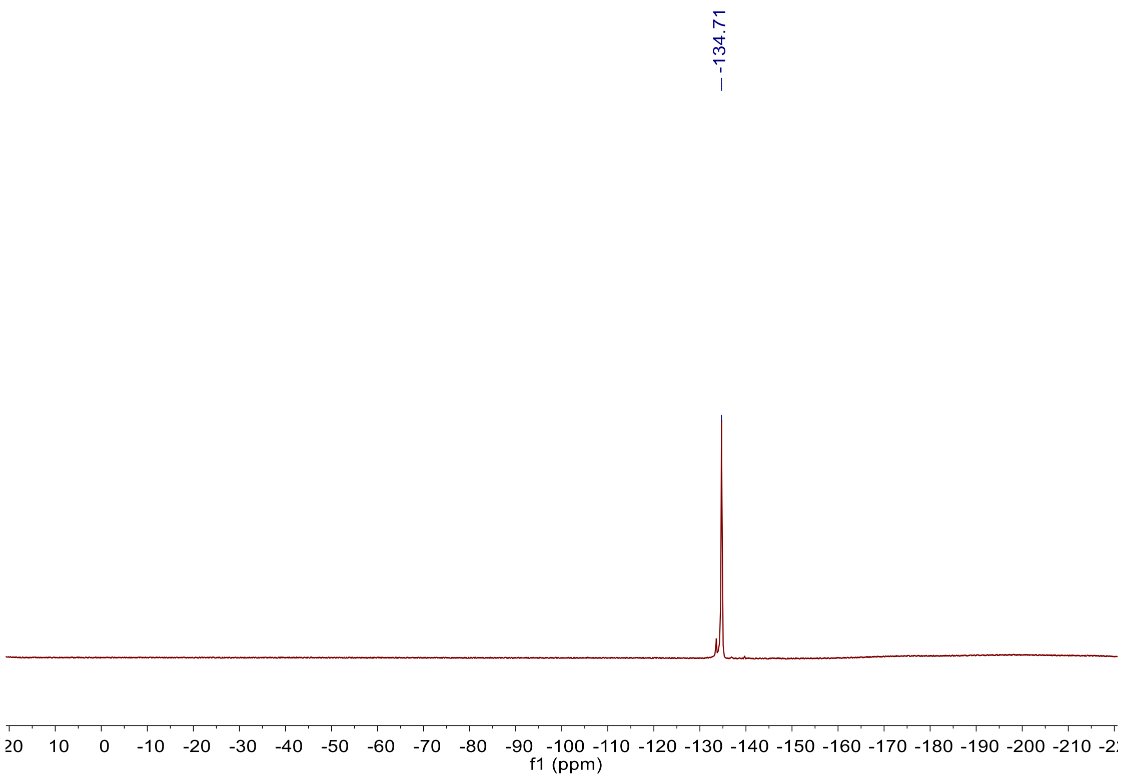

^{19}F NMR spectrum of **56** (Dimethyl sulfoxide-d_6)

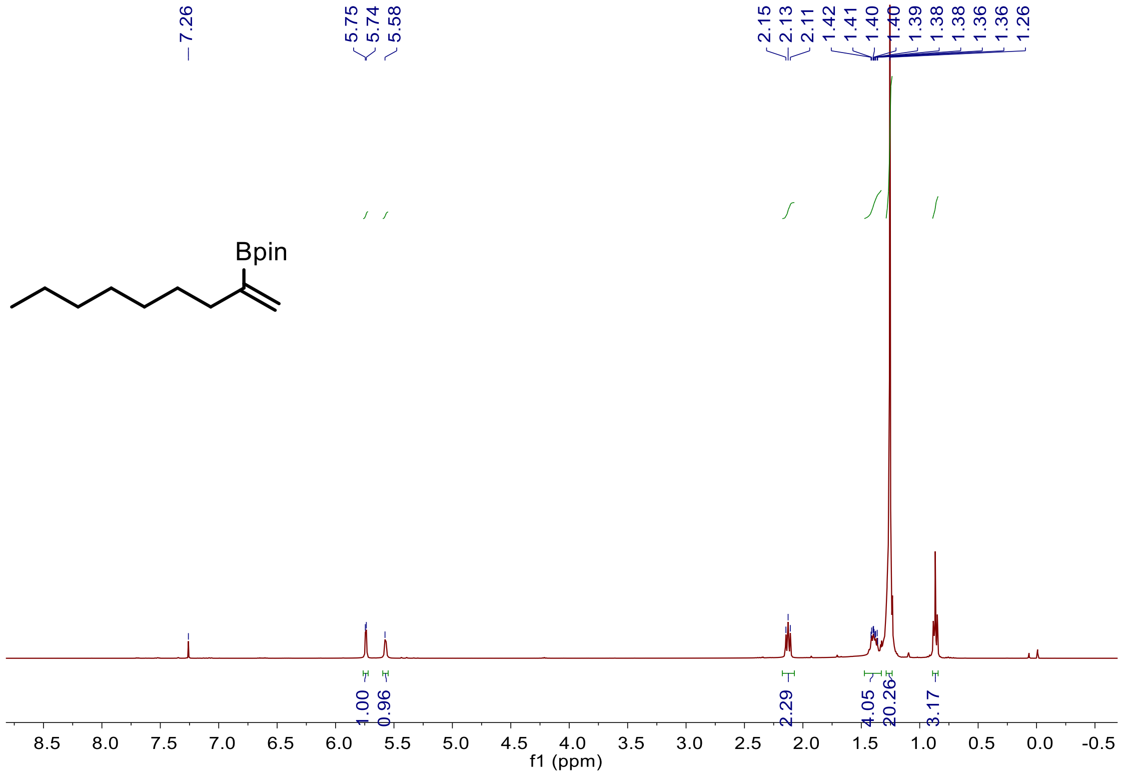

^{1}H NMR spectrum of **57** (Chloroform-d)

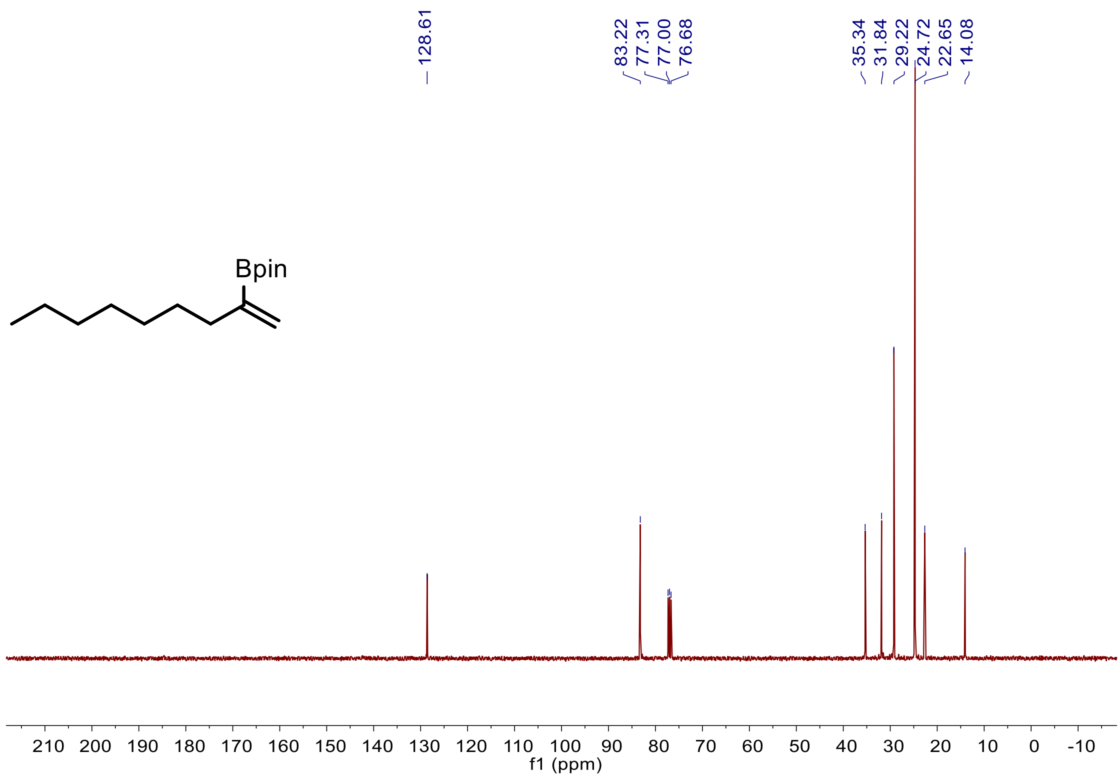

^{13}C NMR spectrum of **57** (Chloroform-*d*)

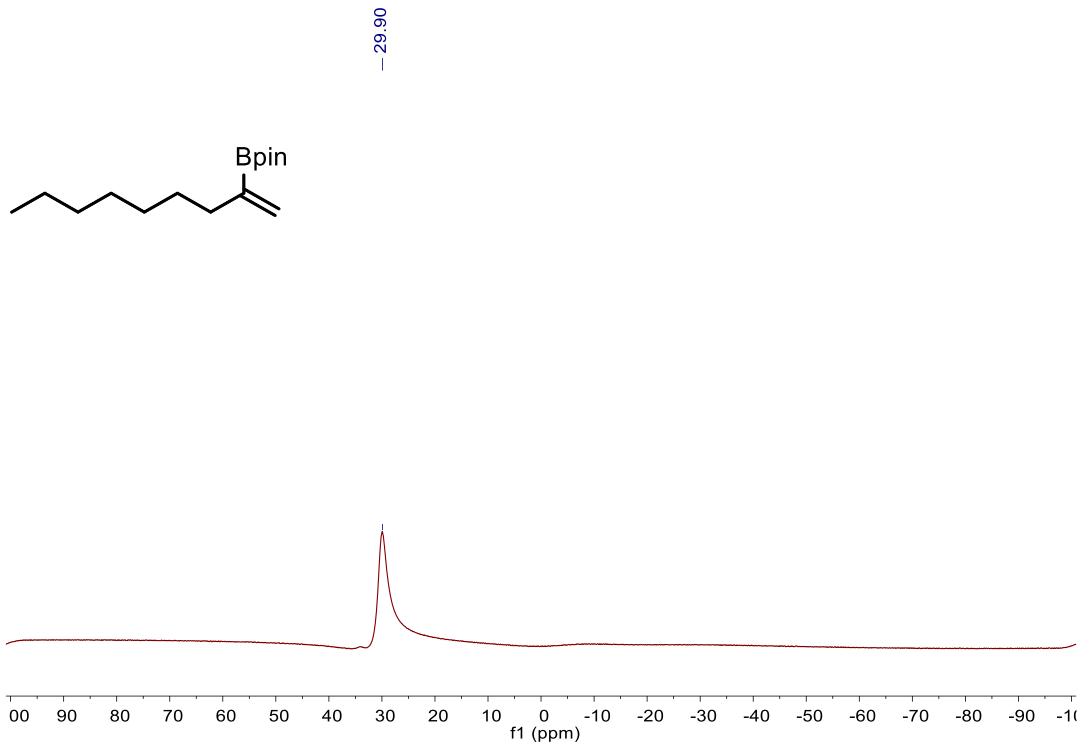

^{11}B NMR spectrum of **57** (Chloroform-*d*)

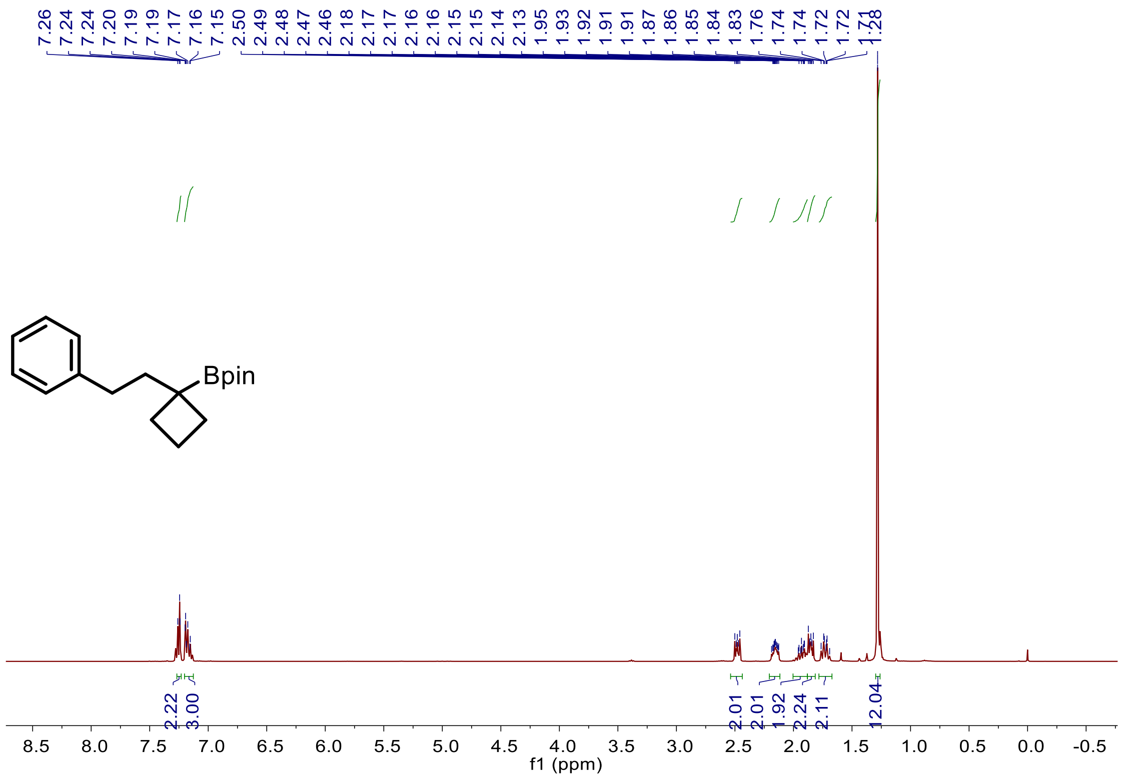

^{1}H NMR spectrum of **58** (Chloroform-*d*)

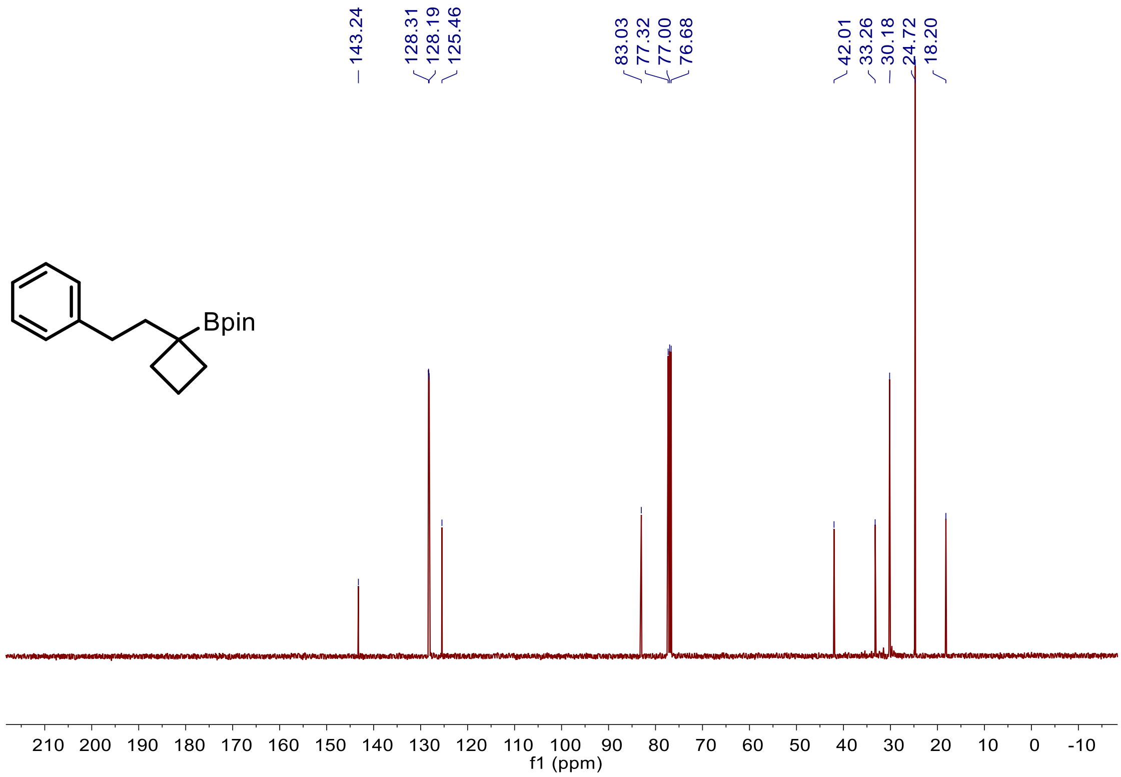

^{13}C NMR spectrum of **58** (Chloroform-*d*)

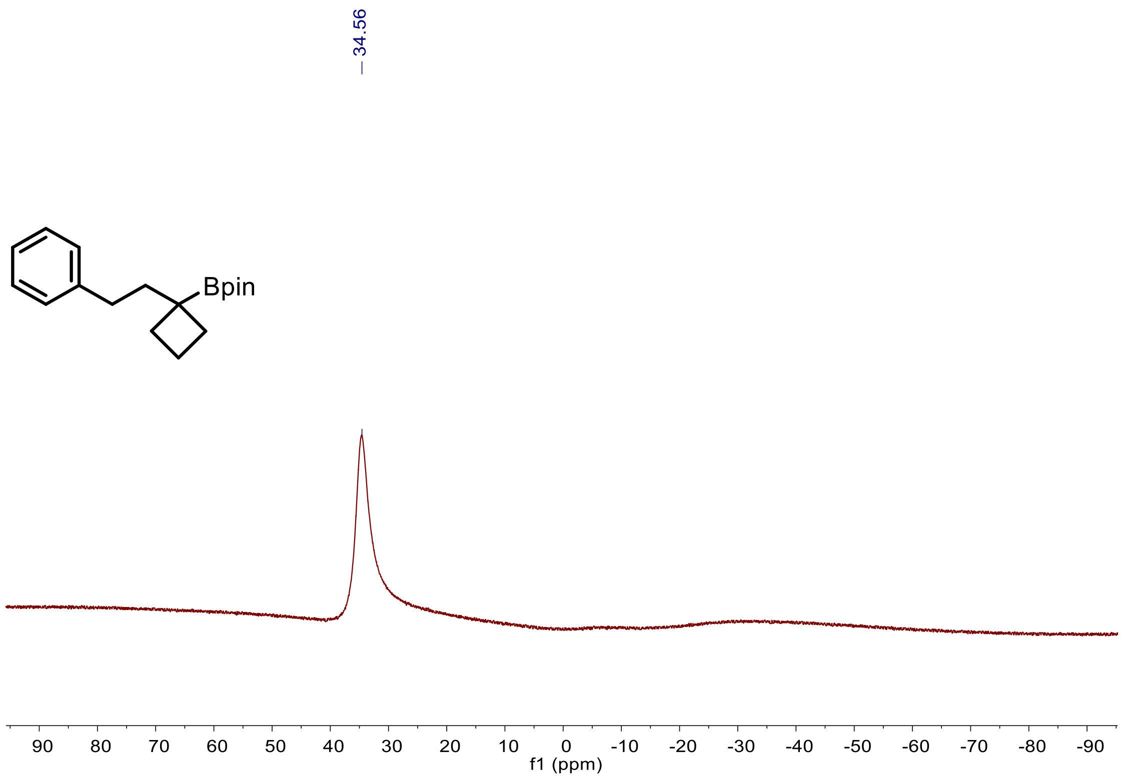

^{11}B NMR spectrum of **58** (Chloroform-*d*)

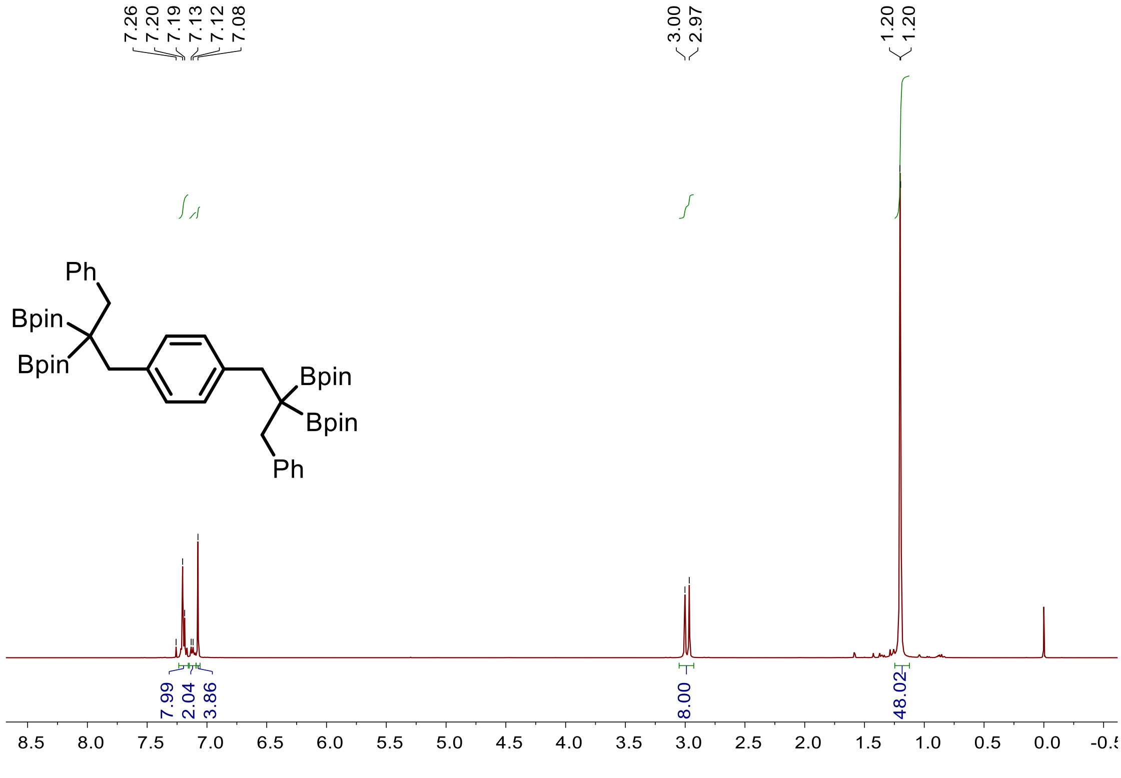

^{1}H NMR spectrum of **59** (Chloroform-*d*)

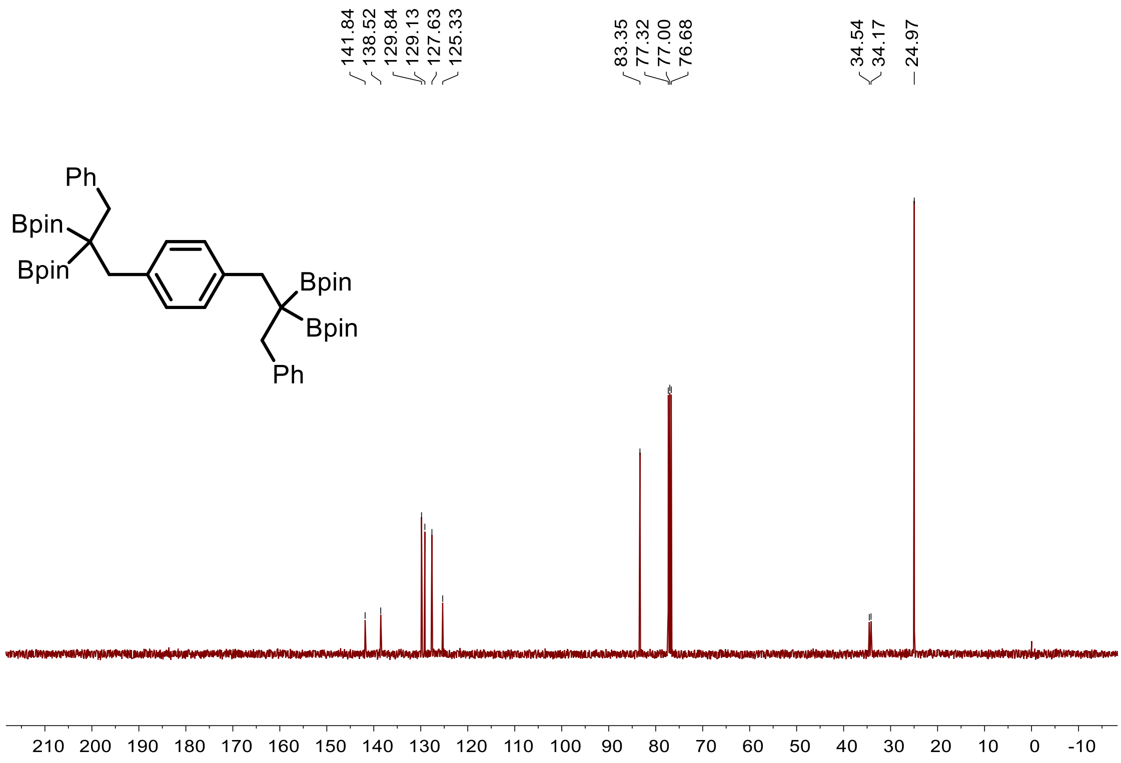

^{13}C NMR spectrum of **59** (Chloroform-*d*)

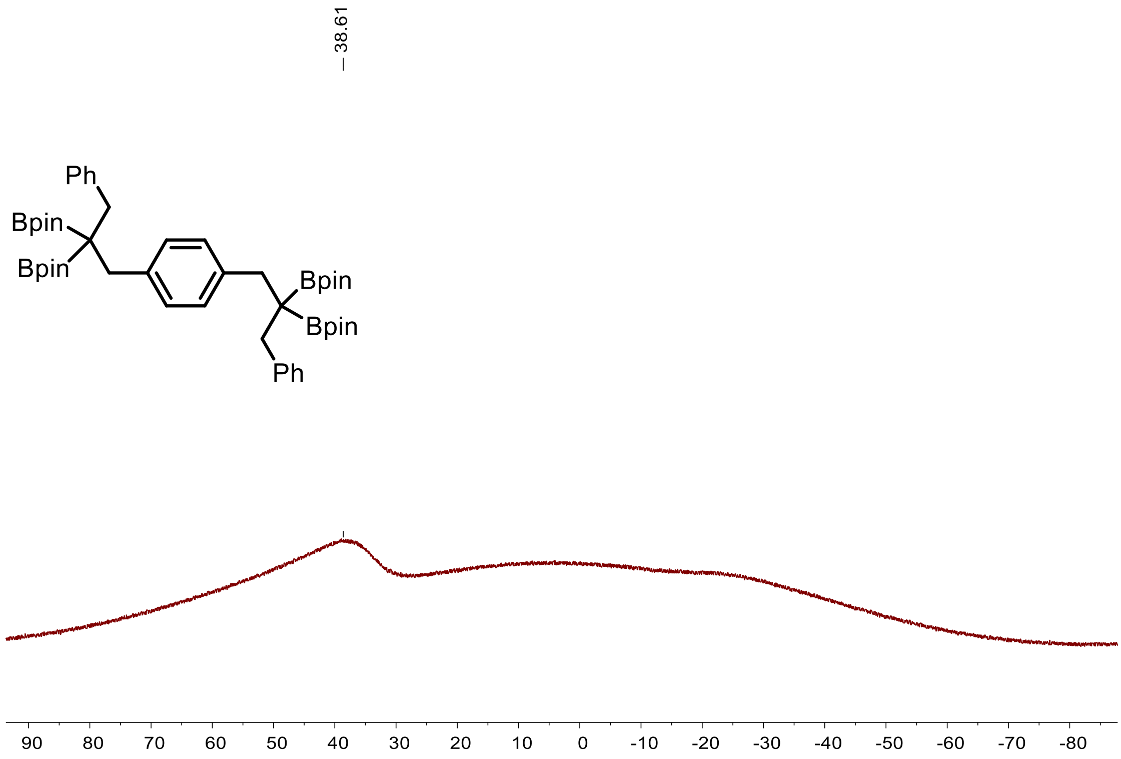

^{11}B NMR spectrum of **59** (Chloroform-*d*)

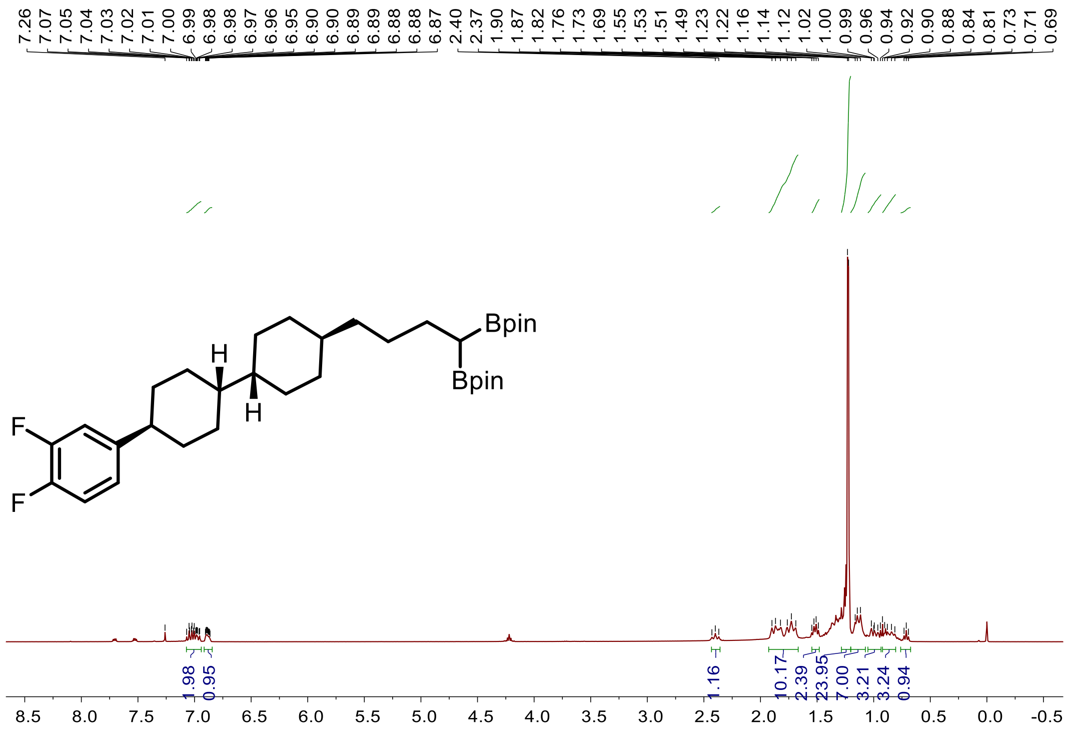

^{1}H NMR spectrum of **60** (Chloroform-*d*)

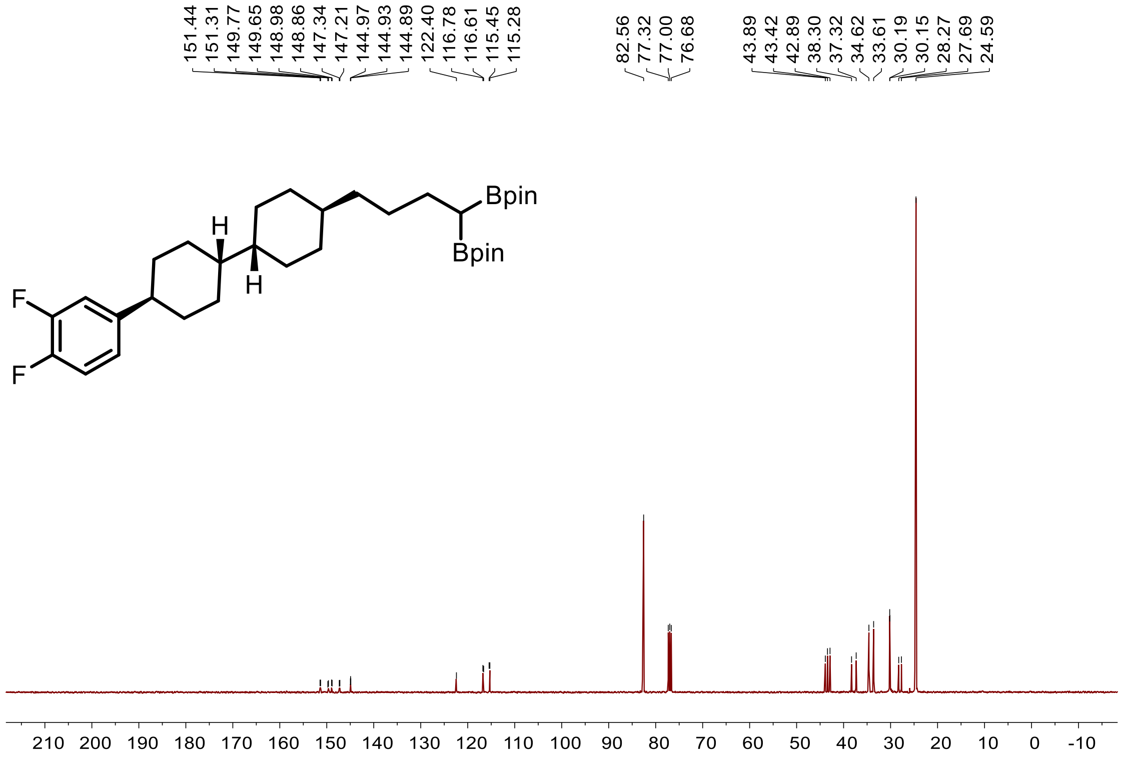

^{13}C NMR spectrum of **60** (Chloroform-*d*)

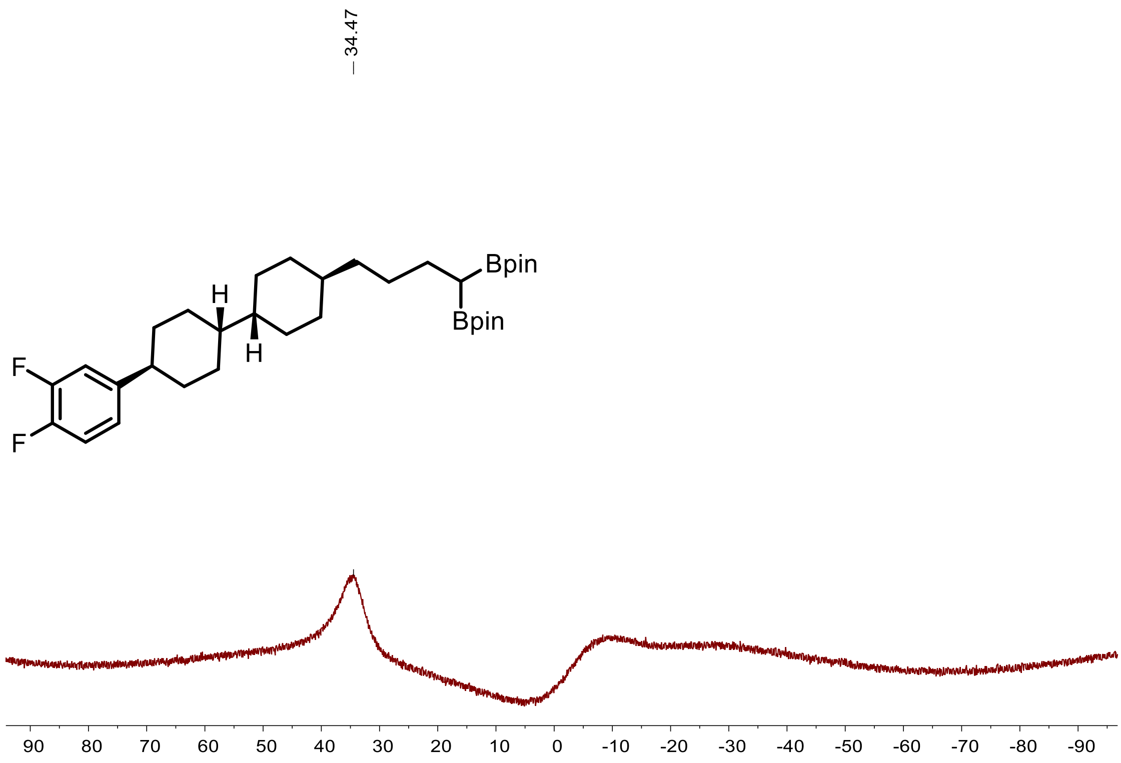

^{11}B NMR spectrum of **60** (Chloroform-*d*)

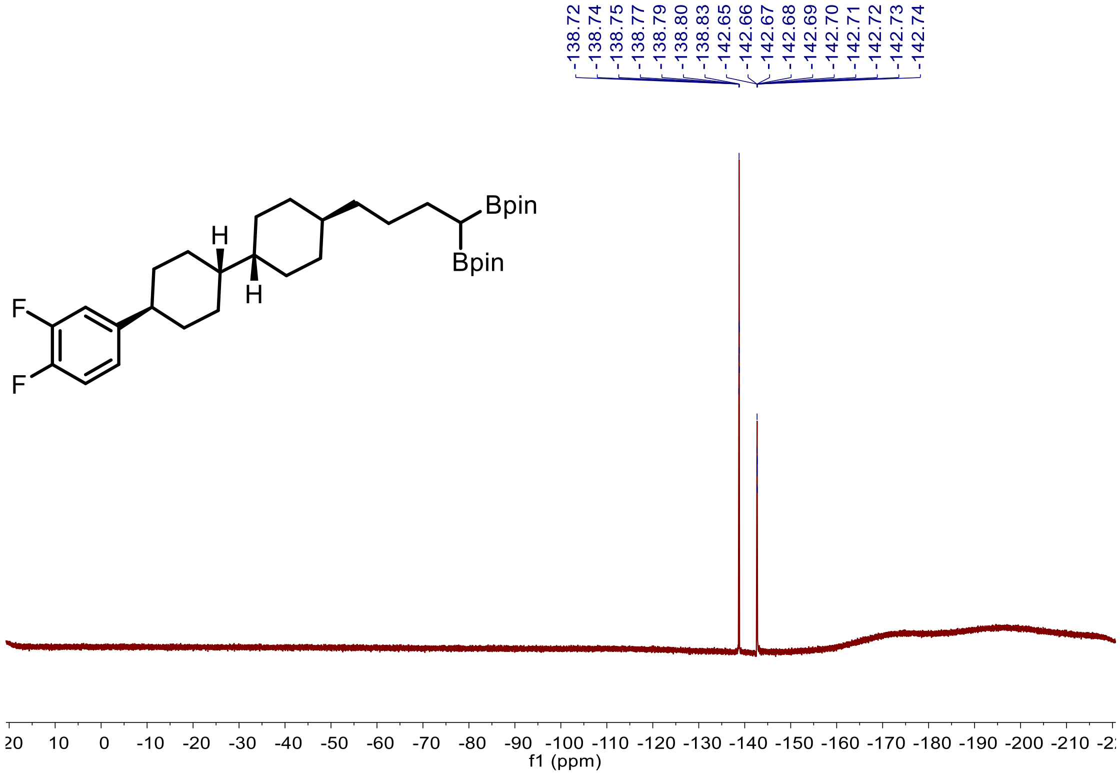

^{19}F NMR spectrum of **60** (Chloroform-*d*)

References

[1] G. A. Molander, A. R. Brown, *J. Org. Chem.* **2006**, *71*, 9681-9686.

[2] T. Kawasaki, H. Tanaka, T. Tsutsumi, T. Kasahara, I. Sato, K. Soai, *J. Am. Chem. Soc.* **2006**, *128*, 6032-6033.

[3] a) K. Endo, T. Ohkubo, M. Hirokami, T. Shibata, *J. Am. Chem. Soc.* **2010**, *132*, 11033-11035; b) C. Sun, B. Potter, J. P. Morken, *J. Am. Chem. Soc.* **2014**, *136*, 6534-6537; c) H. H. Rau, N. S. Werner, *Bioorg. Med. Chem. Lett.* **2018**, *28*, 2693-2696.

[4] N. Kumar, R. R. Reddy, A. Masarwa, *Chem. Eur. J.* **2019**, *25*, 8008-8012.

[5] K. Endo, M. Hirokami, T. Shibata, *J. Org. Chem.* **2010**, *75*, 3469-3472.

[6] W. Jo, J. Kim, S. Choi, S. H. Cho, *Angew. Chem. Int. Ed.* **2016**, *55*, 9690-9694.

[7] X. Liu, W. Ming, Y. Zhang, A. Friedrich, T. B. Marder, *Angew. Chem. Int. Ed.* **2019**, *58*, 18923-18927.

[8] J. R. Coombs, L. Zhang, J. P. Morken, *Org. Lett.* **2015**, *17*, 1708-1711.

[9] K. Hong, X. Liu, J. P. Morken, *J. Am. Chem. Soc.* **2014**, *136*, 10581-10584.

[10] L. Li, T. Gong, X. Lu, B. Xiao, Y. Fu, *Nat. Commun.* **2017**, *8*, 345.

[11] Z. Q. Zuo, Z. Huang, *Org. Chem. Front.* **2016**, *3*, 434-438.

[12] C. E. Iacono, T. C. Stephens, T. S. Rajan, G. Pattison, *J. Am. Chem. Soc.* **2018**, *140*, 2036-2040.

[13] K. Endo, M. Hirokami, T. Shibata, *Synlett* **2009**, *2009*, 1331-1335.

[14] W. J. Teo, S. Ge, *Angew. Chem. Int. Ed.* **2018**, *57*, 1654-1658.

[15] C. Hwang, W. Jo, S. H. Cho, *Chem. Commun.* **2017**, *53*, 7573-7576.

[16] S. Lee, D. Li, J. Yun, *Chem. Asian J.* **2014**, *9*, 2440-2443.

[17] X. Liu, T. M. Deaton, F. Haeffner, J. P. Morken, *Angew. Chem. Int. Ed.* **2017**, *56*, 11485-11489.

[18] M. Maji, K. Chakrabarti, D. Panja, S. Kundu, *J. Catal.* **2019**, *373*, 93-102.

[19] L. T. Ball, M. Green, G. C. Lloyd-Jones, C. A. Russell, *Org. Lett.* **2010**, *12*, 4724-4727.

[20] W. M. Yuan, S. M. Ma, *Adv. Synth. Catal.* **2012**, *354*, 1867-1872.
